# Substitution, Elimination, and Integration of Methyl Groups in Terpenes Initiated by C–H Bond Functionalization

**DOI:** 10.1021/acscentsci.4c01108

**Published:** 2024-08-16

**Authors:** Yi Cheng Kang, Richard T. Wetterer, Rashad R. Karimov, Masahiro Kojima, Max Surke, Inmaculada Martín-Torres, Jeremy Nicolai, Masha Elkin, John F. Hartwig

**Affiliations:** † Department of Chemistry, University of California, Berkeley, California 94720, United States

## Abstract

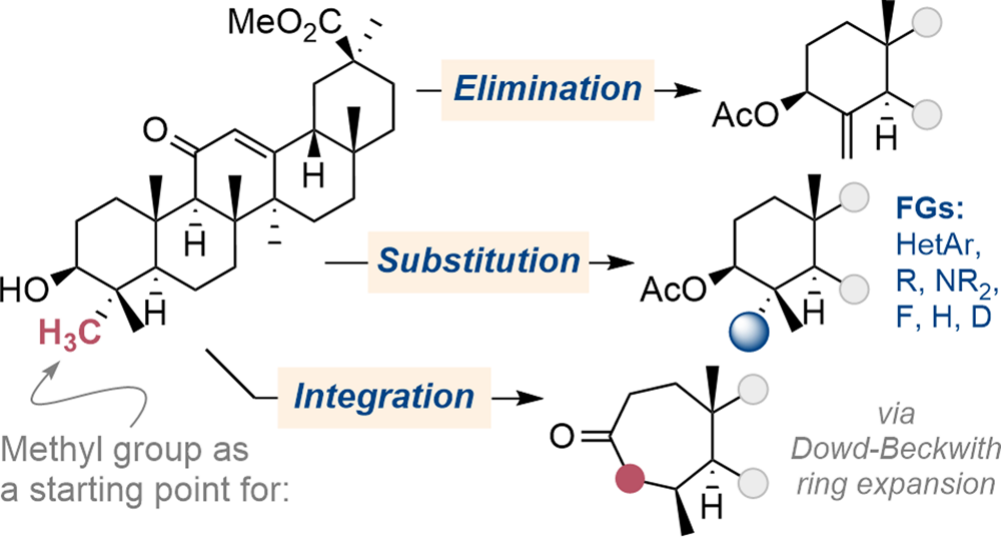

Methyl groups are ubiquitous in natural products and biologically active compounds, but methods for their selective transformation in such structures are limited. For example, terpenoids contain many methyl groups, due to their biosynthetic pathways, but few reactions of these groups in such structures have been reported. We demonstrate that the combination of methyl C–H silylation and oxidation proximal to native hydroxyl or carbonyl groups occurs in a range of terpenoids and show that the installed hydroxyl group serves as a toehold to enable substitution, elimination, or integration of the methyl carbon into the terpenoid skeleton by the cleavage of C–C bonds. In one case, substitution of the entire methyl group occurs by further oxidation and decarboxylative coupling. In a second, substitution of the methyl group with hydrogen occurs by photochemical hydrodecarboxylation or epimerization by retro-Claisen condensation. In a third, photocatalytic decarboxyolefination formally eliminates methane from the starting structure to generate a terminal olefin for further transformations. Finally, a Dowd–Beckwith-type rearrangement cleaves a nearby C–C bond and integrates the methyl group into a ring, forming derivatives with unusual and difficult-to-access expanded rings. This strategy to transform a methyl group into a synthon marks a distinct approach to restructuring the skeletons of complex architectures and adding functional groups relevant to medicinal chemistry.

## Abstract 2

The C−H silylation of methyl groups in complex terpenoids unlocks diverse transformations, including elimination, substitution with other functional groups, and integration into an expanded ring.

## Introduction

Methyl groups are ubiquitous in natural products and biologically active compounds, and in many cases the presence or absence of the group is known to affect the solubility, conformation, or binding of these compounds to biological targets.^1^ Despite their prevalence and function, methods for the modification of methyl groups in complex molecules to increase their value further are limited because the selective cleavage of a C–H or C–C bond connected to the methyl carbon atom is challenging to achieve. The existing C–C bond is nonpolar and inaccessible to catalysts and reagents, and cleavage of the C–H bond is challenging because the primary C(*sp*^3^)–H bonds of the methyl group are stronger (101 kcal/mol)^2^ than secondary or tertiary C(*sp*^3^)–H bonds and electronically unactivated (p*K*_a_ ∼ 50).^3^ Thus, functional groups and weaker C–H bonds are typically the sites of reaction.

Reactions at the methyl groups in complex structures could be valuable to initiate sequences that modify nearby C–C bonds and install new functional groups or alter the molecule’s framework. It is well established that catalytic reactions at C–H bonds can add functional groups in place of hydrogen and alter the periphery of a molecule;^4^ less established are studies showing how the functionalization of C–H bonds can lead to the removal or change in connectivity of C–C bonds within complex molecules in concert with the replacement of the methyl group with functional groups that are important to medicinal chemistry.

**Scheme 1:**
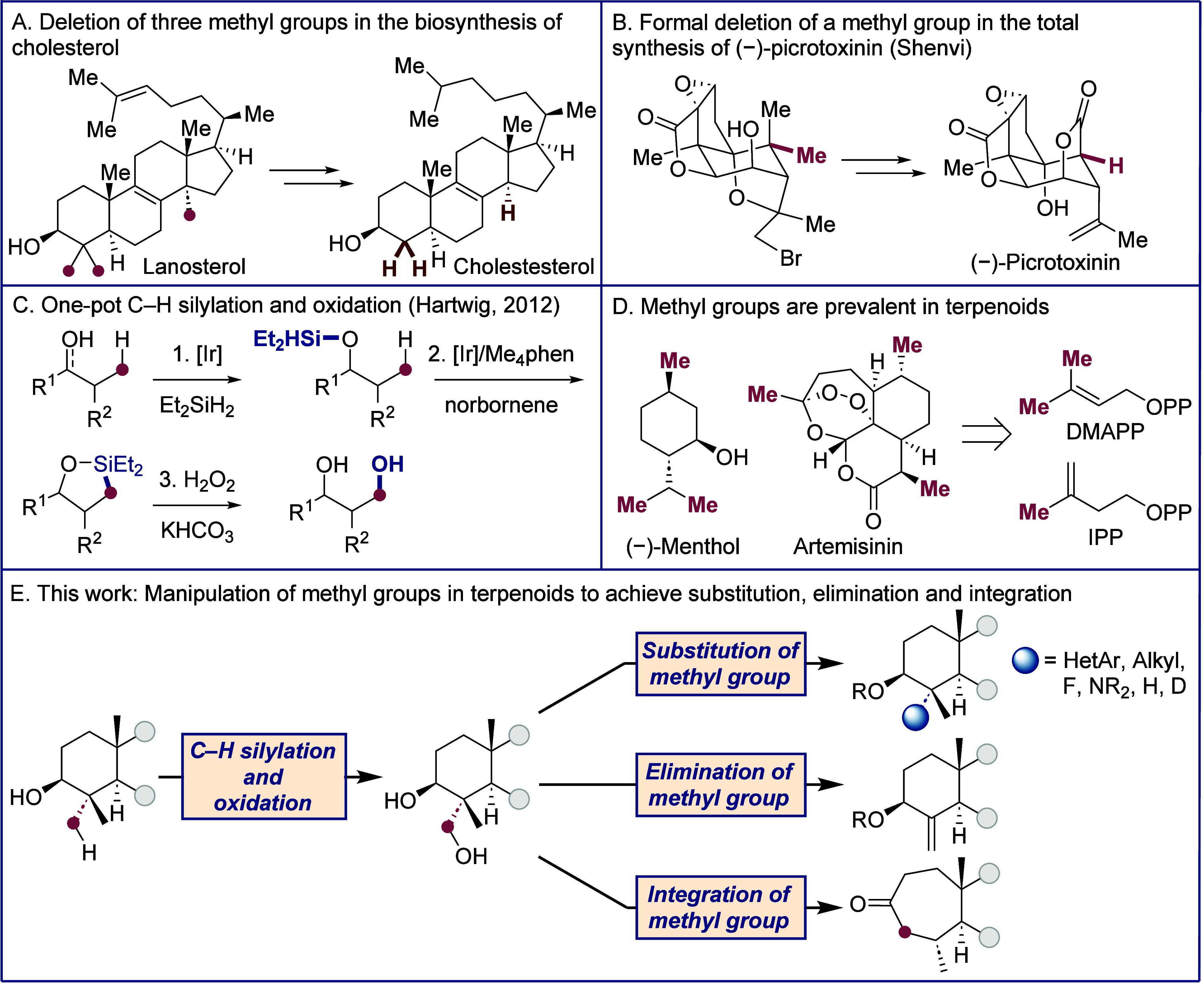
(A) Deletion of Three Methyl Groups in the Biosynthesis of Cholesterol; (B) Formal Deletion of a Methyl Group in the Total Synthesis of (−)-Picrotoxinin (Shenvi); (C) One-Pot C–H Silylation and Oxidation Directed by Alcohols or Ketones; (D) Methyl Groups Are Prevalent in Terpenoids Because of Their Biosynthetic Pathway; (E) This Work: Manipulation of Methyl Groups in Terpenoid Skeletons to Achieve Substitution, Elimination, and Integration

The functionalization of C–H bonds to initiate the cleavage of C–C bonds is well-known in biosynthetic pathways. For example, the “deletion” (i.e., substitution with hydrogen) of three methyl groups initiated by site-selective 1° C(*sp*^3^)–H oxidation occurs during the biosynthesis of cholesterol from squalene in all animals (Scheme 1A).^5^ Although less common, the elimination of methyl groups also has been used in the total synthesis of terpenoids. For example, Shenvi and co-workers formally deleted a methyl group in their synthesis of the sesquiterpenoid (−)-picrotoxinin (Scheme 1B)^6^ by a four-step sequence involving a Suárez oxidation of a C–H bond directed by a proximal alcohol. These structural changes to the carbon skeleton by reactions that ultimately cleave the C–C bond can strongly affect the biological activity of the compound. For example, deletion of the C19-methyl group of progesterone forms a derivative with 4–8 times greater binding affinity to progesterone receptors as progesterone itself;^7^ this discovery eventually led to the development of norethisterone, the first oral contraceptive.^8^

Methods that do exist to cleave a primary C–H bond and modify a methyl group typically rely on C(*sp*^3^)–H bond activation catalyzed by a transition-metal complex coordinated by a directing group.^9^ Catalytic processes based on the insertion of extremely sterically hindered rhodium carbenoids into primary C–H bonds in the absence of a directing group have been developed, but hindered methyl groups do not react, and the scope of functional groups that can be installed is limited by the requirement of a donor−acceptor carbene.^10^ C(*sp*^3^)–H bond activation is selective for methyl C(*sp*^3^)–H bonds because the formation of a primary alkyl-metal bond is kinetically^11−13^ and thermodynamically^14,15^ favorable over that of secondary or tertiary alkyl-metal bonds, and the directing group controls the site of functionalization. To alleviate the required preinstallation and subsequent removal of specific directing groups for such transformations^16−18^ and recognizing that alcohols are the most common functional group present in natural products,^19^ our group developed approaches to use alcohols and ketones as native directing groups for an iridium-catalyzed C(*sp*^3^)–H silylation and oxidation sequence (Scheme 1C).^20^ Dehydrogenative silylation of the alcohol or hydrosilylation of the ketone with diethylsilane, intramolecular C(*sp*^3^)–H silylation, and Tamao–Fleming oxidation of the 5-membered oxasilacycle in one pot afforded 1,3-diol products.^21^

Terpenoids are one class of natural products that typically contain multiple methyl groups. They contain at least one methyl group per five carbon atoms because they trace their biosynthetic origin to dimethylallyl and isopentenyl pyrophosphate units from the mevalonate pathway (Scheme 1D).^22^ Thus, methods for the replacement of methyl groups in terpenoids with other functional groups or methods in which functionalization of a methyl group can induce modifications to the terpenoid skeleton would be highly valuable for late-stage diversification. Many methods for the undirected functionalization of secondary, tertiary, or allylic C–H bonds in terpenes are known,^23,24^ but methods that modify the methyl groups in these structures are less common. Costas and co-workers have reported the manganese-catalyzed C–H lactonization of methyl groups γ to carboxylic acids,^25,26^ but the scope of the reaction is limited because carboxylic acids are less common in natural products than alcohols or ketones.^19^

Here, we report a strategy that combines our ability to convert methyl groups proximal to native alcohol or carbonyl moieties into hydroxymethyl groups within a range of terpenoid structures with an ability to use the installed functionality for sequences that lead to substitution, elimination, or integration of the methyl group (Scheme 1E). In some cases, these sequences occur with concomitant installation of new groups or atoms, including heteroaryl groups, amino groups, functionalized alkyl groups, fluorine or deuterium, and, in other cases, with structural rearrangement of the terpenoid core. We anticipate that this approach will widen the chemical space that can be accessed from natural terpenoids and synthetic structures with methyl groups proximal to an oxygen-based functionality and demonstrate, most generally, a synthetic strategy for deep-seated changes initiated by transformations at a methyl group.

## Results and Discussion

### 1 C(*sp*^3^)–H Silylation of Terpenoids

**Scheme 2:**
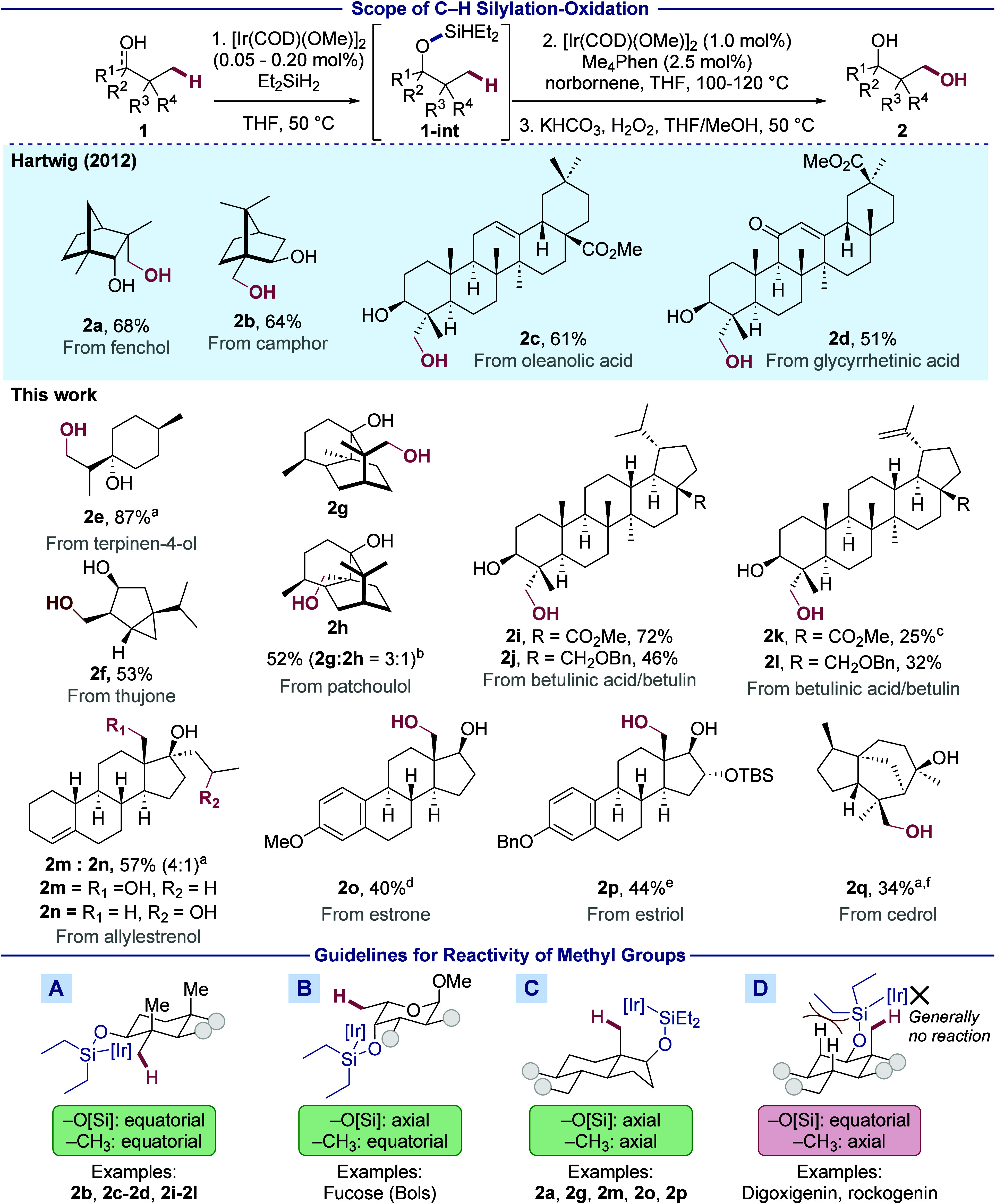
Silylation and Oxidation of Terpenoids Step 3 performed with CsOH·H_2_O (12 equiv), tBuOOH (14 equiv), TBAF (5 equiv), DMF. Step 2 performed with [Rh(COD)_2_Cl]_2_ (2 mol %) and Xantphos (4.4 mol %) instead of [Ir(COD)(OMe)]_2_ and Me_4_phen. Step 3 performed with added KF (2.5 equiv) in DMF instead of THF. Step 3 performed with KF (10 equiv) and *m*CPBA (10 equiv) in DMF. Step 3 performed with KHF_2_ (2.5 equiv) and *m*CPBA (3 equiv) in DMF. Step 1 performed with RuCl_2_(PPh_3_)_3_ (0.2 mol %) instead of [Ir(COD)(OMe)]_2_. See Supporting Information for detailed reaction conditions. Isolated yields are given. Me_4_Phen = 3,4,7,8-tetramethyl-1,10-phenanthroline.

To begin our studies on the restructuring of complex molecules initiated by the silylation of C–H bonds, we first surveyed the silylation and oxidation of methyl groups in a series of terpenoids. Various monoterpenoids, sesquiterpenoids, triterpenoids, and steroids underwent the silylation of methyl C(*sp*^3^)–H bonds to set the stage for subsequent manipulation of the erstwhile methyl group (Scheme 2). We had previously reported the silylation and oxidation of (+)-fenchol, (+)-camphor, methyl oleanolate, and methyl glycyrrhetinate (**2a**–**d**),^20^ but to access a wider range of structures and to establish the scope and limitations of this approach to functionalize methyl groups in terpenoids, we conducted this reaction sequence with a range of additional terpenoids. The monoterpenoids (−)-dihydroterpinen-4-ol (**2e**) and (−)-thujone (**2f**) were hydroxylated with complete selectivity for the γ-methyl group. (−)-Patchoulol, an important fragrance compound,^27^ was hydroxylated to form 14-hydroxypatchoulol **2g** and 13-hydroxypatchoulol **2h** in a 3:1 ratio and 52% overall yield. Sarpong and co-workers recently reported a *de novo* synthesis of 14-hydroxypatchoulol,^28^ itself a natural product isolated from *V. stenoptera*.^29^ We accessed this compound in a one-pot synthesis from (−)-patchoulol in 39% yield. Methyl dihydrobetulinate underwent silylation and oxidation to form diol **2i** in 72% yield on a 0.4 g scale and 65% yield on a 1.1 g scale. A dihydrobetulin derivative underwent the same process to form diol **2j** in 46% yield. Betulin and betulinic acid derivatives bearing a 1,1-disubstituted olefin, similarly, underwent this functionalization to form diols **2k** and **2l**, albeit in lower yields. In an effort to increase the water solubility of betulinic acid, which has anticancer and anti-HIV properties, Baran reported the hydroxylation of the benzyl ester analog of **2k** in 24% yield.^30^ A derivative of allylestrenol formed a 4:1 mixture of products **2m** and **2n** from functionalization at methyl and methylene positions, respectively. 18-Hydroxy derivatives of the steroids estrone and estriol formed in moderate yields (**2o, 2p**). The sesquiterpenoid cedrol, which does not contain any 1 °C–H bonds γ to the alcohol, formed the product **2q** from 1,4-silylation and oxidation in 34% yield.

Based on the observed reactivity of the terpenoids we examined, we have devised a set of guidelines for the iridium-catalyzed C–H silylation of cyclic and polycyclic structures summarized at the bottom of Scheme 2. These rules are predominantly based on the equatorial or axial disposition of the diethyl(hydrido)silyl ether group and the proximal potentially reactive methyl group. Three relationships between the silyl ether and the potentially reactive methyl group tend to form products from silylation of the methyl C–H bonds. First, compounds bearing an equatorial silyl ether and an equatorial methyl group generally undergo silylation of a methyl C–H bond in good yield (Case A). Examples include the diterpenoid pleuromutilin, reported by Herzon,^31^ camphor (**2b**), and the plant-derived triterpenoids (**2c**, **2d**, and **2i**-**2l**). Second, substrates containing an axial silyl ether and an equatorial methyl group, such as the fucose derivatives reported by Bols,^32,33^ undergo the reaction in good yield (Case B). Third, compounds containing axial or pseudoaxial silyl ether and methyl groups can undergo the silylation process (Case C). The axial hydroxyl groups in the bicyclo[2.2.1]heptane and bicyclo[2.2.2]octane skeletons of fenchol and patchoulol are close in space to the axial methyl groups, and both substrates undergo the C–H silylation process at this methyl group to lead to 1,3-diols (**2a**, **2g**). The pseudoaxial silyl ether moiety in the five-membered D-ring in steroid skeletons, such as that in **2m**, **2o**, and **2p**, direct functionalization to the axial C-18 methyl group.

The disposition of silyl ether and methyl groups that typically did not react contain an equatorial silyl ether and a proximal axial methyl group, especially a methyl group at a ring junction (Case D). Examples of structures containing alcohols and methyl groups that generate such silyl ethers include digoxigenin, rockogenin, and 5β-hydroxycholestane, shown in Section 3 of the Supporting Information and summarized in Scheme 2. In molecules containing an equatorial silyl ether and proximal geminal dimethyl substituents, the silylation usually occurred exclusively at the equatorial methyl group in this unit (**2c**, **2d**, and **2i**-**2l**).

The origin of this selectivity can be rationalized by consideration of the stability of *cis-* versus *trans*-fused rings and steric environment of a methyl group in an axial or equatorial position. *Trans*-decalin rings are more stable than *cis*-decalins, and *trans*-5,6-fused ring systems are more stable than the analogous *cis-*fused rings;^34^ these relative stabilities are linked to the greater steric hindrance of the axial positions versus the equatorial positions of cyclohexane rings. Thus, the preference for reaction of the equatorial methyl group of the *gem*-dimethyl unit vicinal to the silyl ether (see Case A) results from the formation of an oxasilametallacycle with a *trans*-decalin-type structure and a *trans*-5,6-fused ring structure in the oxasilacyclopentane product. Reaction of the axial methyl group would require the formation of a higher-energy *cis*-decalin-type iridacycle wherein the ethyl substituents of the silane experience severe *syn*-pentane interactions with the axial substituents of the cyclohexane ring (see Case D). Thus, the barrier to C–H oxidative addition of an axial methyl group from an iridium bound to an equatorial silyl ether is prohibitive, and no examples of oxasilolane products with this relative geometry were observed in the substrates we surveyed. The reaction with an equatorial methyl group directed by an axial silyl ether (Case B) also requires the formation of an iridacycle with *cis*-decalin geometry, but because the methyl group is oriented away from the ring, *syn*-pentane interactions with the diethylsilyl group and iridium center are avoided.

Finally, the scenario where both the methyl group and the silyl ether are axial typically occurs in bicyclic or 5,6-fused ring systems (Case C). For such compounds, we propose that ground state destabilization and the greater proximity of the two reacting groups favor the C–H oxidative addition. The methyl group and the silyl ether are locked in relatively high-energy eclipsed conformations in the bicyclo[2.2.1]heptane or bicyclo[2.2.2]octane ring systems of **1a** and **1g**, which decreases the energetic penalty for forming the iridacycle. The pseudoaxial silyl ether in the steroids (**1m, 1o, 1p**) is not fully eclipsed with the methyl group but is nonetheless situated in close proximity to that group. Moreover, the bulky ethyl substituents of the silane are pointed away from the ring system and, thus, do not experience steric interactions in the transition state that prohibit oxidative addition of the methyl C–H bond. Overall, we have demonstrated that this sequence of C–H silylation and oxidation occurs with a range of monoterpenoids, sesquiterpenoids, steroids, and triterpenoids and that a set of simple guidelines can predict the outcomes of the reaction based on the geometries of the directing group and the proximal methyl group.

### 2 Substitution of Methyl Groups

**Scheme 3:**
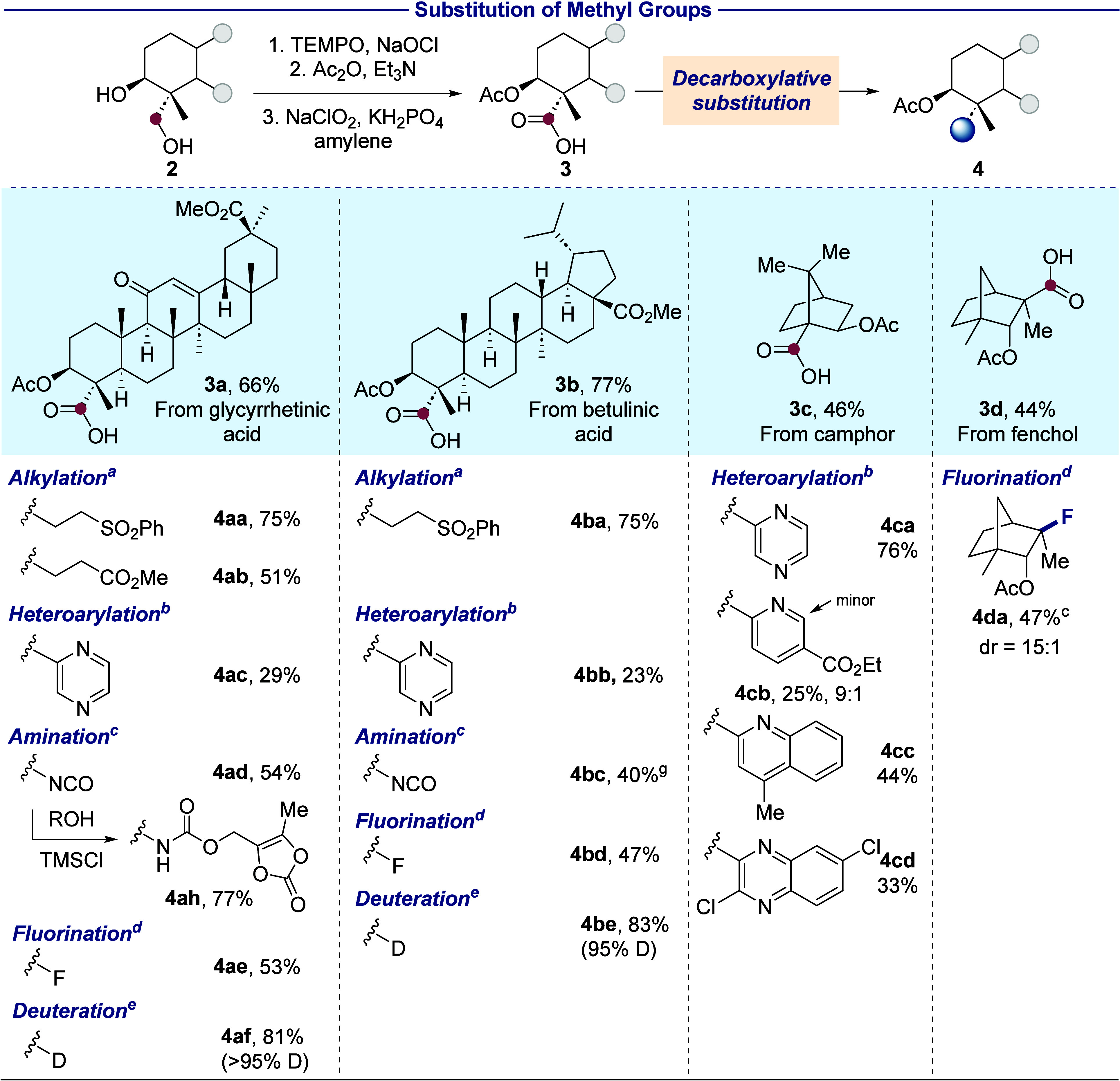
Substitution of Methyl Groups in Terpenoids Olefin (1.5 equiv), Ir[dF(CF_3_)ppy]_2_(dtbpy)PF_6_ (1.0 mol %), K_2_HPO_4_ (1.2 equiv), DMF, blue LEDs, 30 °C, 16 h. 1. PhthN–OH, DIC, DMAP, CH_2_Cl_2_, rt, 16 h. 2. Heterocycle (2 equiv), CF_3_COOH, 4-CzIPN (2–4 mol %), DMSO, blue LEDs, 45 °C, 16 h. (PhO)_2_P(O)N_3_ (1.1 equiv), Et_3_N (1.5 equiv), anisole, 85 °C, 20 h. Selectfluor (2.1 equiv), 2,6-lutidine (1.8 equiv), Fe(OAc)_2_ (10 mol %), 4,4′-(MeO)-2,2′-bipy (10 mol %), blue LEDs, MeCN/H_2_O, 30 °C. 1. CsOH, MeOH, 25 °C, 1 h. 2. Ir[dF(CF_3_)ppy]_2_(dtbpy)PF_6_ (1.0 mol %), TRIP-SH (10 mol %), CH_2_Cl_2_/D_2_O, blue LEDs, 30 °C, 16 h. See Supporting Information for detailed reaction conditions. Isolated yields are given.

With the hydroxylated products **2a**–**2q** in hand, we examined various methods for substitution, elimination, and integration of the hydroxymethyl group. A formal substitution would enable the late-stage diversification of terpenoid skeletons by conversion of the typically unreactive methyl group into a variety of functional groups.^35^ To achieve such substitutions, we developed conditions to oxidize the newly installed primary alcohol to a carboxylic acid (see Section 4A of the Supporting Information) and to protect the pre-existing secondary alcohol as an acetate (Scheme 3). β-Acetoxyacids **3a**–**3d** were synthesized in moderate to good yields from diols **2a**–**2d** by this sequence. The carboxylic acid moiety then underwent substitution by various photocatalytic decarboxylations,^36^ leading to the formal, overall replacement of the methyl group with a series of alkyl groups, aryl groups, and functional groups.

Substitution of a methyl group with an alkyl chain can improve the binding affinity of organic structures to a biological target by increasing van der Waals interactions within a hydrophobic binding site, and this binding can be further modulated by the functional groups appended to the attached alkyl groups.^37,38^ To show the potential to use the alcohol from silylation and oxidation for the installation of alkyl and functionalized alkyl groups, we prepared β-acetoxyacid **3a** from glycyrrhetinic acid. Glycyrrhetinic acid derivative **3a** underwent decarboxylative Giese reactions with methyl acrylate and phenyl vinyl sulfone to form alkyl derivatives **4aa** and **4ab** in 75% and 51% yield.^39^ Carboxylic acid **3b** derived from betulinic acid also underwent alkylation to form **4ba**. The sulfone in **4ba** can be cleaved under reductive conditions to reveal an ethyl group,^40^ resulting in formal homologation of the methyl group.

Nitrogen-containing heteroarenes are contained in hundreds of FDA-approved drugs because they participate in strong binding interactions and confer favorable physicochemical properties.^41^ However, terpenoids usually do not contain such heteroarenes. Therefore, we examined decarboxylative Minisci reactions to install heteroaryl fragments in place of the methyl group of terpenoid structures. To do so, the carboxylic acid in glycyrrhetinic acid derivative **3a** was substituted with pyrazine to form **4ac** in 29% yield.^42^ Formal substitution of the methyl group in betulinic acid derivative **3b** with pyrazine formed **4bb** in 23% yield. Finally, a similar sequence with camphor-derived acid **3c** converted the original methyl group with pyrazinyl, pyridinyl, quinolinyl, and quinoxalinyl units to form **4ca-4cd** in 25–76% yields.

In addition to the nitrogen atoms in heteroarenes, nitrogen atoms in amino groups or amide derivatives are present in a large fraction of FDA-approved drugs,^41^ but they are notably absent in most plant-derived terpenoids. Therefore, we sought to install such nitrogen-containing groups from carboxylic acid units by Curtius rearrangement of an *in situ*-generated acyl azide.^43,44^ Indeed, treatment of acids **3a** and **3b** with diphenylphosphoryl azide led to isocyanates **4ad** and **4bc** in moderate yields (Scheme 3). Treatment of isocyanate **4ad** with an alcohol bearing a dioxolone moiety formed carbamate **4ah**, a derivative of glycyrrhetinic acid that was investigated for the treatment of hyperkalemia.^44^

The substitution of H or Me with fluorine in a biologically active compound typically improves its metabolic stability.^45^ Thus, we sought to convert the methyl group to a fluoride by C(*sp*^3^)–H silylation, oxidation, and decarboxylative fluorination. By this sequence, fluorinated derivatives of glycyrrhetinic acid (**4ae**), betulinic acid (**4bd**), and fenchol (**4da**) were prepared. The decarboxylative fluorination step occurred in 47%–53% yields with an iron catalyst.^46^

Deuterated compounds are increasingly prominent in pharmaceuticals because the kinetic isotope effect can retard metabolism at positions of a drug prone to oxidation with essentially no change in the drug’s physicochemical properties.^47^ Photocatalytic deuterodecarboxylation of glycyrrhetinic and betulinic acid derivatives with D_2_O,^48^ the cheapest source of deuterium,^49^ generated products **4af** and **4be** in 81% and 83% yields, respectively, with 95% deuterium incorporation. In all cases, the new group was installed with retention of configuration, likely due to preferential trapping of the radical from the less hindered pseudoequatorial face. We emphasize that the same β-acetoxyacid intermediate **3** derived from 1,3-diol **2** was used for all five decarboxylative substitution reactions, thereby illustrating the versatility of our strategy for the late-stage diversification of a biologically active compound.^50^

### 3 Deletion of Methyl Groups

**Scheme 4:**
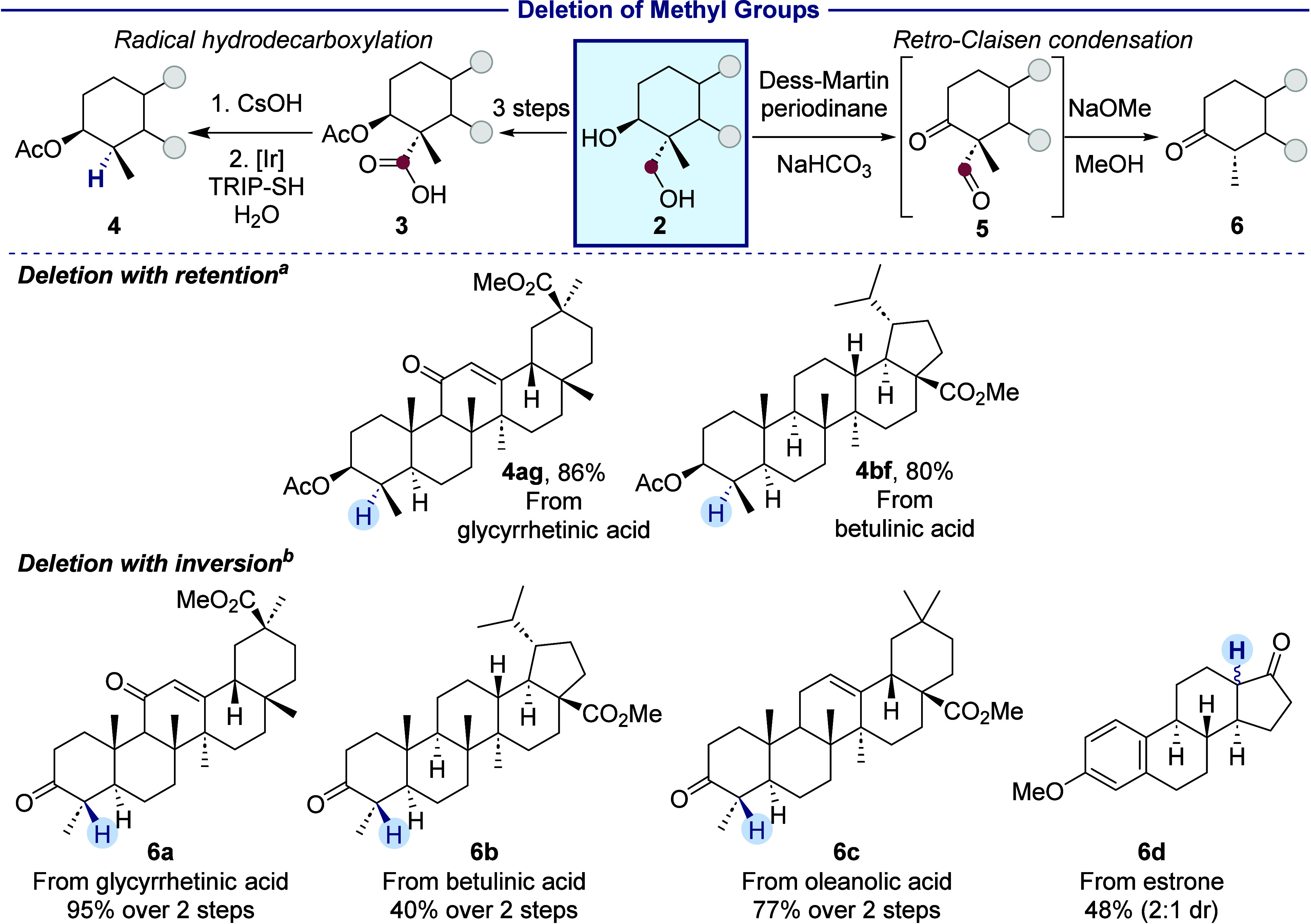
Deletion of Methyl Groups in Terpenoids with Retention and Inversion of Configuration 1. CsOH, MeOH, 25 °C, 1 h. 2. Ir[dF(CF_3_)ppy]_2_(dtbpy)PF_6_ (1.0 mol %), TRIP-SH (10 mol %), CH_2_Cl_2_/H_2_O, blue LEDs, 30 °C, 16 h. 1. Dess-Martin periodinane (2.4 equiv), NaHCO_3_ (10 equiv), CH_2_Cl_2_, rt, 4 h. 2. NaOMe (1.5 equiv), MeOH, rt, 16 h. Isolated yields are given.

The deletion of a methyl group (a formal substitution of hydrogen for a CH_3_ group) from terpenes is shown in Scheme 4. This deletion is crucial to the biosynthesis of cholesterol, as well as plant and fungal sterols,^51^ and it can strongly affect the binding affinities of drugs (*vide supra*).^7,52^ We performed a direct decarboxylation of acids **3a** and **3b** under photocatalytic conditions without the preinstallation of a thiohydroxamate ester, as required in a more conventional Barton decarboxylation.^49^ The demethylated terpenoids **4ag** and **4bf** formed in high yields with complete retention of configuration.

We hypothesized that the demethylation also could proceed with net inversion of configuration if the demethylation was conducted by a process during which the two stereoisomeric products were allowed to equilibrate. The 4α-methyl isomer should be more stable because the methyl group is oriented equatorially and syn-pentane interactions are avoided.^53^ While the trapping of the tertiary alkyl radical is kinetically controlled and irreversible, the protonation of a putative enolate intermediate would be reversible. To generate this enolate, we oxidized diol **2** to a β-ketoaldehyde **5** and treated it with sodium methoxide to trigger a retro-Claisen condensation. This retro-Claisen condensation leads to loss of the methyl group as methyl formate. Demethylated triterpenoid derivatives of glycyrrhetinic acid (**6a**), betulinic acid (**6b**), and oleanolic acid (**6c**)^54^ were synthesized by this sequence. Although it is the functionalized methyl group that is excised, *in situ* epimerization under the alkaline conditions led to the product from formal deletion of the *unfunctionalized* methyl group, as designed. An estrone derivative also underwent demethylation to form **6d**, although a mixture of diastereomeric ketones was generated because the stabilities of *cis*- and *trans*-5,6-fused rings only differ by ∼0.5 kcal/mol.^34^

**Scheme 5:**
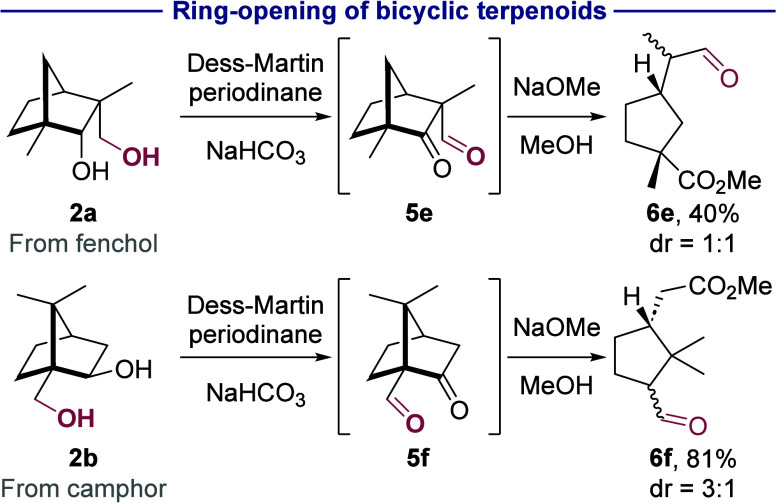
Ring-Opening of Bicyclic Terpenoids Isolated yields are given. Conditions: 1. Dess-Martin periodinane (2.4 equiv), NaHCO_3_ (10 equiv), CH_2_Cl_2_, rt, 4 h. 2. NaOMe (1.5 equiv), MeOH, rt, 16 h.

A retro-Claisen process with the 1,3-dioxygenated intermediates also can lead to the cleavage of C–C bonds to form ring-opened products (Scheme 5). For example, oxidation of the 1,3-diol derived from the bicyclic monoterpenoid fenchol (**2a**) by Dess-Martin periodinane and treatment with sodium methoxide led to ketoester **6e** in 40% yield (dr = 1:1). A similar sequence with the diol derived from camphor (**2b**) formed ketoester **6f** as a 3:1 mixture of diastereomers in 81% yield. The retro-Claisen condensation can occur via nucleophilic addition of methoxide to either the aldehyde or ketone of the intermediate, leading to demethylation or ring opening. In the triterpenoids and steroid derivatives (**6a**–**6d**), the more electrophilic aldehyde is attacked, leading to elimination of methyl formate and overall demethylation (*vide supra*). However, in these bicyclic monoterpenoids, nucleophilic addition to the aldehyde likely occurs reversibly because elimination of methyl formate would generate a strained enolate. Nucleophilic addition of methoxide to the ketone, instead, leads to irreversible ring-opening to form the monocyclic ketoesters **6e** and **6f**. The release of ring strain in the norbornane scaffold (∼15 kcal/mol)^55^ provides a strong driving force for the formation of the observed products.

### 4 Formal Elimination of Methane

**Scheme 6:**
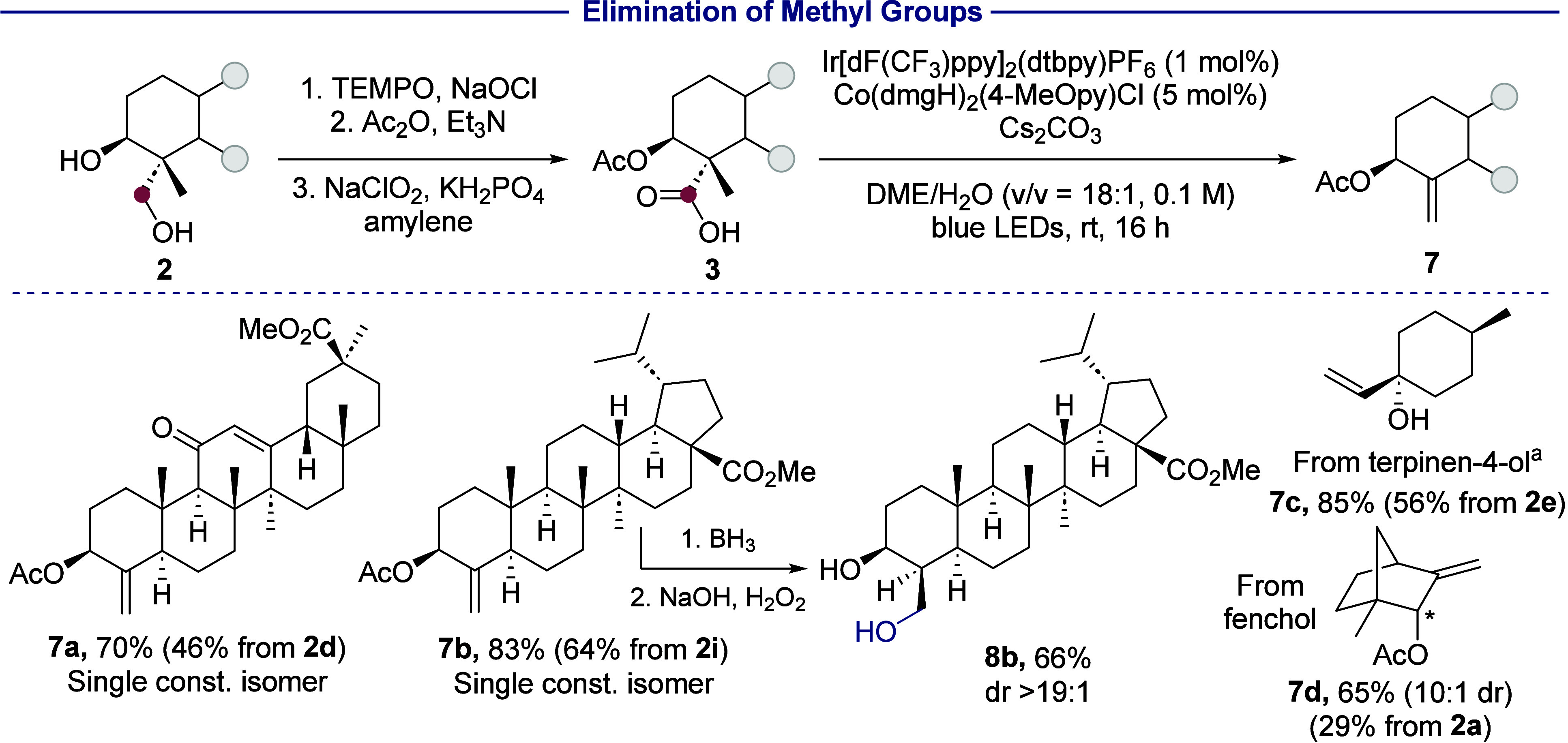
Elimination of Methyl Groups in Terpenoids The tertiary alcohol was not acetylated for this compound. Isolated yields are given. Yields in parentheses are the overall yields over 3–4 steps from the diols.

The formal, overall dehydromethylation of a terpenoid was achieved by excising the functionalized methyl group and concomitantly forming an olefin, which serves as a useful functional handle for further modification of the terpenoid skeleton (Scheme 6).^56,57^ To this end, we followed a photochemical decarboxyolefination protocol reported by Ritter.^58^ In this scenario, oxidation of the methyl group to the carboxylic acid and decarboxyolefination would form an alkene in place of a *gem*-dimethyl unit. Thus, subjection of the β-acetoxyacid intermediates derived from glycyrrhetinic acid (**3a**) and betulinic acid (**3b**) to the reported conditions generated terminal alkenes **7a** and **7b** in 70% and 83% yields respectively. Olefin **7c** formed similarly from carboxylic acid **3e** derived from terpinen-4-ol. The decarboxyolefination of acid **3d** derived from fenchol generated alkene **7d** as a 10:1 mixture of diastereomers, due to a minor amount of epimerization at the carbon bearing the acetate moiety. Complete regioselectivity for the terminal olefin was observed in all cases in which internal isomers could form. Hydroboration and oxidation of alkene **7b** led to anti-Markovnikov hydration to form diol **8b** in 66% yield.^59^ The acetyl protecting group was hydrolyzed under the alkaline conditions of the oxidation step. The hydroboration is highly stereoselective for *syn*-addition to the pseudoequatorial α-face of the olefin, likely because the α-face is more sterically accessible. Therefore, this sequence involving formal dehydromethylation enables the functionalization of methyl groups that cannot be hydroxylated directly by the iridium-catalyzed C(*sp*^*3*^)–H silylation and oxidation sequence, such as the previously unmodified 4β-methyl group (C-24) in **3a** and **3b** and the equatorial methyl group in fenchol derivative **3d**.

### 5 Integration of Methyl Groups

The hydroxylated methyl group also can be integrated into the ring systems of terpenoids. The modified carbon skeletons thus obtained are rare or unknown in naturally occurring terpenoids and could have distinct physicochemical or biological properties.^60,61^ Terpenoids with expanded rings are typically accessed by a three-step sequence including a Tiffeneau-Demjanov rearrangement; however, the ring expansion is often unselective, forming a mixture of constitutional isomers from migration of either of the alkyl substituents of the ketone.^62^ For example, analogs of the neuroactive steroid allopregnanolone with expanded A or D rings have been synthesized and tested as potential drug candidates for the treatment of postpartum depression.^63,64^

**Scheme 7:**
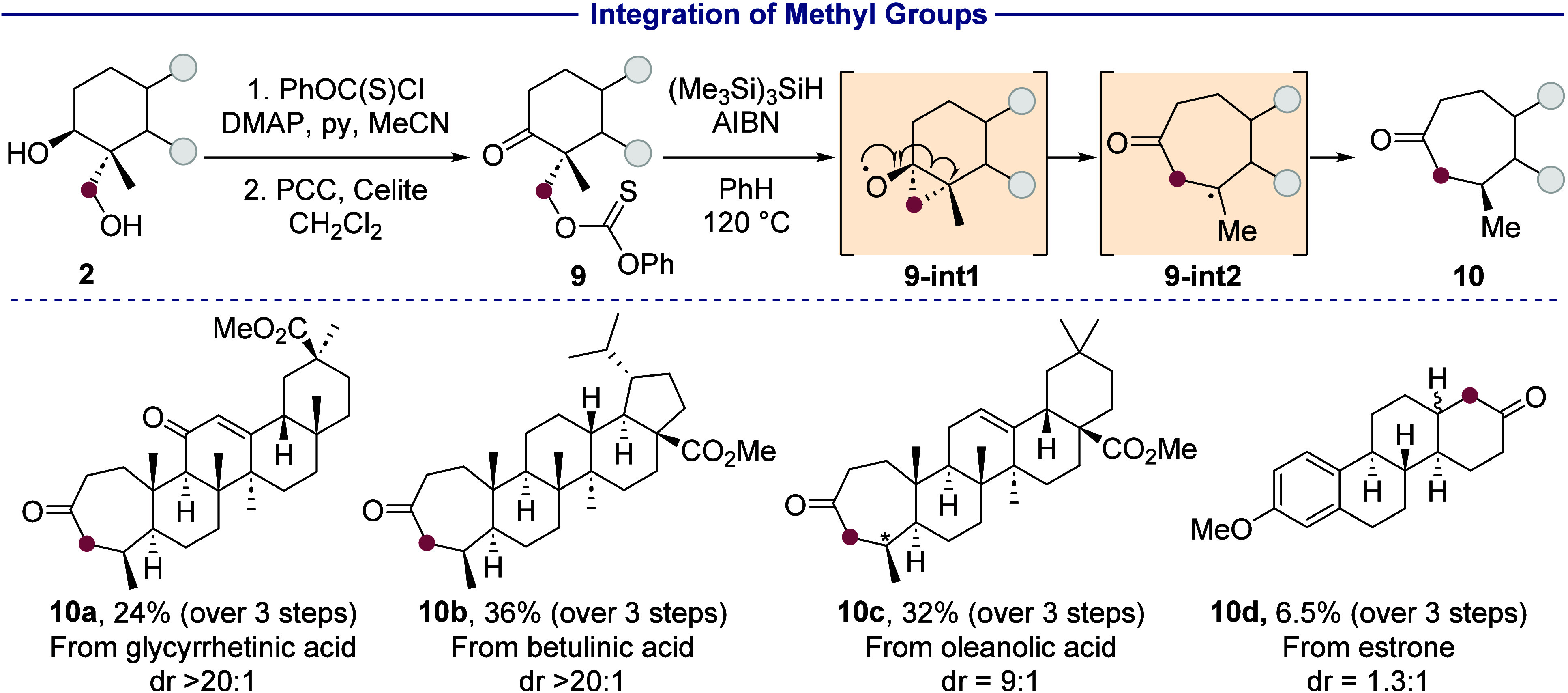
Integration of Methyl Groups in Terpenoids 1. PhOC(S)Cl, pyridine, DMAP (0.1 equiv), MeCN, 0 °C to rt, 16 h. 2. PCC, Celite, CH_2_Cl_2_, rt, 3 h. 3. (Me_3_Si)_3_SiH, AIBN, PhH, 120 °C, 3 h. Isolated yields after the three-step sequence from the diol are given.

Our synthetic strategy for integration of the methyl group was based on the Dowd-Beckwith ring expansion (Scheme 7).^65^ While the classical Dowd–Beckwith reaction is performed on alkyl halides, Chen^66^ and Ding^67^ have reported Dowd–Beckwith-type ring expansions starting from thioxanthates that would more easily be formed from the sterically hindered alcohols in our substrates than the corresponding halides. Selective functionalization of the neopentyl primary alcohol was achieved with *O*-phenyl chlorothionoformate, after which the secondary alcohol was oxidized to give ketone **9**. Among various conditions tested (see Section 6 of the Supporting Information), those with pyridinium chlorochromate formed the ketone in the highest yields; other conditions led to decomposition of the thionocarbonate or to little conversion.

We then intercepted the Barton–McCombie deoxygenation of the thionocarbonate unit in **9** to form the ring expanded product. Rearrangement of the initially generated primary alkyl radical by addition to the ketone formed a cyclopropane containing an alkoxy radical (**9-int-1**). This cyclopropane opened to form a tertiary alkyl radical (**9-int-2**), which was trapped by a hydrogen atom source to form the ring-expanded product. We found that the use of tris(trimethylsilyl)silane, rather than the more commonly used tributylstannane, in this process was crucial to obtaining reproducibly high yields of the product. By this procedure, the hydroxylated methyl group in glycyrrhetinic acid, betulinic acid, and oleanolic acid were incorporated into the A ring to generate analogs containing a seven-membered ring (**10a**–**c**). We hypothesize that the tertiary alkyl radical from the rearrangement is selectively reduced by tris(trimethylsilyl)silane by approach from the more sterically accessible pseudoequatorial face (*vide supra*), leading to the observed major or exclusive diastereomer.

The integration of methyl groups into steroids and monoterpenoids was also investigated. The five-membered D ring in estrone was similarly expanded with this sequence (**10d**). However, attempts to perform the Dowd-Beckwith ring expansion with the corresponding (+)-fenchol derivative led to Barton-McCombie deoxygenation without rearrangement, yielding exclusively (−)-fenchone (see Section 6 of the Supporting Information). The lack of rearrangement in this case is likely due to a greater barrier to formation of the cyclopropyloxy radical intermediate because of ring strain in the norbornane scaffold.

### 6 Application to the Synthesis of Medicinally Relevant Compounds

**Scheme 8:**
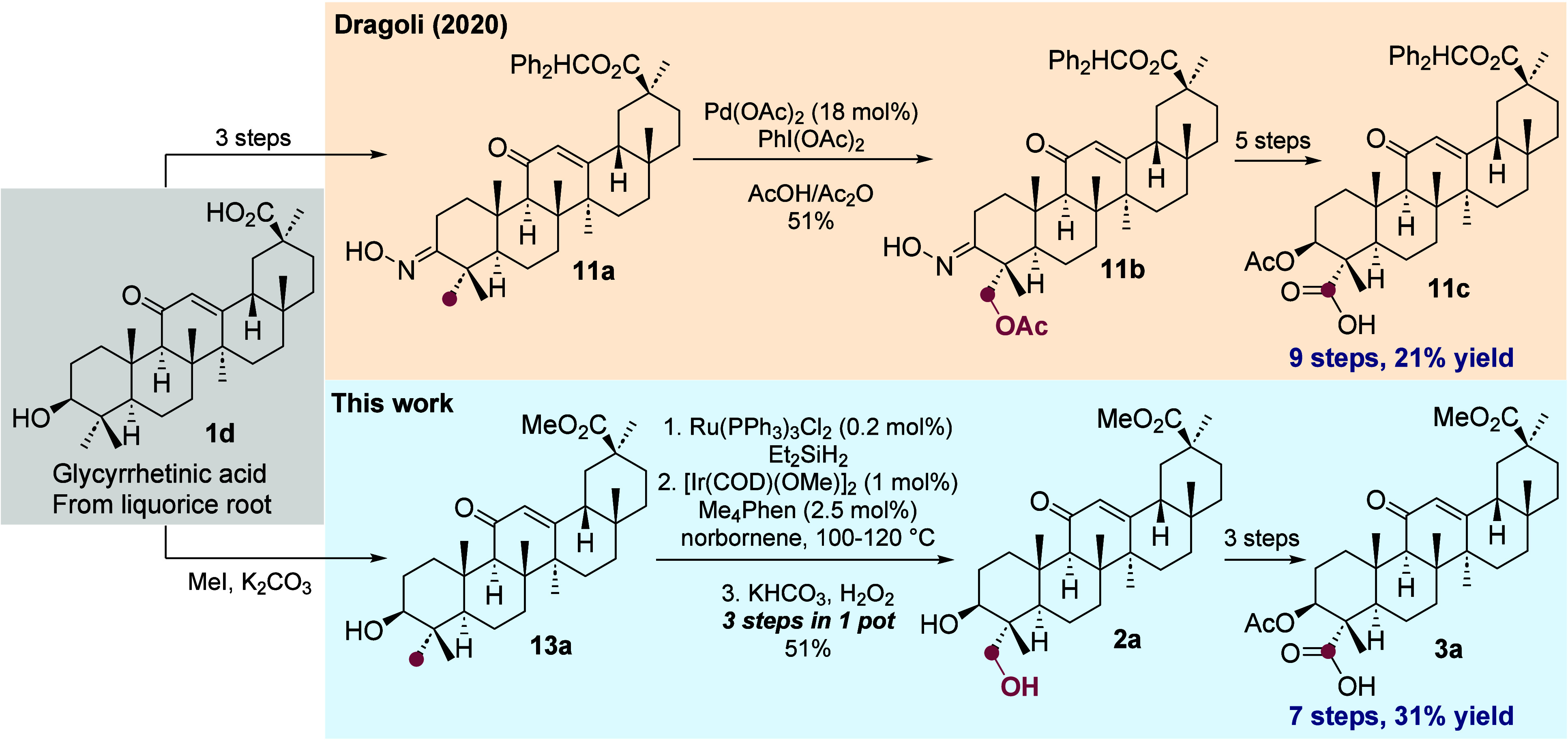
Comparison of Dragoli’s and Our Route to Glycyrrhetinic Acid Derivatives That Are Primed for Reactions That Substitute the Functionalized Methyl Group

The substitution of methyl groups in biologically active terpenoids has been exploited for studies of structure–activity relationships.^68^ For example, Dragoli and co-workers used palladium-catalyzed C(*sp*^*3*^)–H acetoxylation of **11a** to synthesize intermediate **11c** (Scheme 8). Subsequent Curtius rearrangement and nucleophilic addition of an alcohol generated functionalized carbamate derivatives that were drug candidates for the treatment of hyperkalemia.^44^ The C(*sp*^*3*^)–H activation of the methyl group in their synthesis required the installation of an oxime as a directing group, necessitating four functional group interconversions,^69^ as well as a high loading (18 mol %) of palladium catalyst. In our sequence, the C(*sp*^*3*^)–H silylation and oxidation of the methyl group was performed in one pot using only 2 mol % of iridium and 0.2 mol % of ruthenium. Thus, we were able to synthesize the key carboxylic acid-containing glycyrrhetinic acid derivative **3a** (analogous to **11c**), which is primed for the Curtius rearrangement or other decarboxylative substitution reactions, in 31% yield over 7 steps in 5 separate vessels compared to the prior 21% yield over 9 separate steps in 9 vessels.

## Conclusions

We have shown how the site-selective hydroxylation of methyl groups proximal to ketone or alcohol units in various terpenoids by the combination of C–H silylation and oxidation directed by native hydroxyl groups enables the initial juxtaposed methyl group to serve as a synthetic handle for transformations that substitute, eliminate, or integrate this group by cleavage or formation of carbon-carbon bonds, or both. Substitution of the methyl group installs a series of medicinally relevant functional groups, including heteroarenes, fluorine, and deuterium. Substitution of the methyl group with hydrogen occurs with retention or inversion of configuration, depending on the class of reaction used for C–C bond cleavage. Elimination of the methyl group leads to selective formation of a terminal olefin, thereby activating one of the original geminal methyl units for further functionalizations. Integration of the methyl group into the carbon skeleton of the terpenoid leads to modified structures with expanded rings that are rare in nature and are otherwise difficult to access synthetically. We anticipate that this strategy will find applications in the synthesis and pharmacology of terpenoid derivatives, as well as other natural products or synthetic intermediates bearing methyl groups.

## Supporting Information Available

Experimental procedures, characterization of new compounds, and spectroscopic data (PDF)
