# Supplementary material for: Substitution, Elimination, and Integration of Methyl Groups in Terpenes Initiated by C–H Bond Functionalization
Source: ACS Cent Sci. 2024 Aug 16;10(11):2016–27. doi: 10.1021/acscentsci.4c01108 (PMC11613304; doi:10.1021/acscentsci.4c01108)
Supplement: Supplementary file 1 — oc4c01108_si_001.pdf [file oc4c01108_si_001.pdf]

Supporting Information

Substitution, elimination, and integration of methyl groups in terpenes initiated by C–H bond functionalization

Yi Cheng Kang, Richard T. Wetterer, Rashad Karimov, Masahiro Kojima, Max Surke, Inmaculada Martín-Torres, Jeremy Nicolai, Masha Elkin, and John F. Hartwig*

*Corresponding author – John F. Hartwig – *Department of Chemistry, University of California, Berkeley, California 94720, United States*; Email: jhartwig@berkeley.edu

Table of Contents

1. General Considerations 2
2. Synthesis of starting materials 3
3. Silylation-oxidation of terpenoids 8
4. Substitution of methyl groups 19
- ***4a. Synthesis of carboxylic acid intermediates*** 19
- ***4b. Decarboxylative Giese reactions*** 22
- ***4c. Decarboxylative Minisci reactions*** 26
- ***4d. Decarboxylative fluorination*** 30
- ***4e. Decarboxylative amination*** 30
- ***4f. Decarboxylative deuteration*** 36
- ***4g. Decarboxylative protonation*** 38
- ***4h. Retro-Claisen condensation sequence*** 40

5. Elimination of methyl groups 45
6. Integration of methyl groups 49
7. References 49
8. NMR spectra 59

1. General Considerations

i) Experimental procedures

Unless otherwise noted, all reactions were performed in oven-dried (165°C) or flame-dried glassware containing a Teflon-coated stirring bar under a nitrogen atmosphere using standard Schlenk techniques. Reactions involving moisture and air sensitive solids were assembled in a nitrogen-filled glovebox. Reaction temperatures above 23 °C refer to temperatures of an aluminum heating block or an oil bath, which were controlled by an electronic temperature modulator. Reactions were monitored by NMR, GC-FID or GC-MS analysis.

ii) Solvents and reagents

Commercial substrates were used as received, unless otherwise stated. Solvents were purchased from commercial suppliers and dried by standard procedures or obtained from the solvent-drying system (Innovative Technologies, Newburyport, MA) and stored over 3Å or 4Å molecular sieves. $[Ir(COD)OMe]_2$, norbornene, estrone, betulinic acid, (-)-α-thujone, (+)-fenchol and (+)-camphor were purchased from Sigma-Aldrich, Me_4phen was purchased from ChemScene, diethylsilane was purchased from Alfa Aesar and stored in a nitrogen-filled glovebox, terpinen-4-ol was purchased from Frontier Science, estriol was purchased from AK Scientific, betulin was purchased from Adipogen, patchouli alcohol was purchased from TRC Canada, allylestrenol and (+)-cedrol were purchased from TCI. Molecular sieves were activated by heating under high vacuum or in a microwave.

iii) Analytical Instrumentation

^{1}H, ^{13}C{^{1}H} and ^{19}F{^{1}H} NMR spectra were recorded on 400, 500, 600 and 700 MHz Bruker instruments at the College of Chemistry's Pines NMR facility at the University of California, Berkeley at 25°C if not otherwise stated. All NMR data are reported in parts per million (ppm) relative to the residual solvent signal (δ 7.26 for ^{1}H NMR, δ 77.16 for ^{13}C NMR in $CDCl_3$). Coupling constants are reported in Hertz (Hz). Signal forms and multiplicities were displayed using standard abbreviations: s = singlet, d = doublet, t = triplet, q = quartet, quint = quintet, sex = sextet, hept = heptet, m = multiplet, br = broad, dd = doublet of doublets, ddd = doublet of doublets of doublets, dddd = doublet of doublets of doublets of doublets, dq = doublet of quartets, dt = doublet of triplets, td = triplet of doublets, tdd = triplet of doublets of doublets. Signal assignment was supported by DEPT, COSY, HSQC, HMBC and NOE experiments. Quantitative GC analyses were carried out on an HP 6890 GC and an FID detector. Column chromatography was performed on a ISCO Combiflash Teledyne Flash Chromatography-System using RediSept Gold packed columns. Mass spectrometric data were acquired on a GC-MS Agilent 5977B MSD. HRMS analyses and mass spectral data were acquired on a Waters GCT Premier CAB163 with a TOF mass analyzer. The MS ionization was achieved by ESI. Acetonitrile was typically used as the solvent. Thin-layer chromatography (TLC) was performed on F_{254} silica gel plates (60 µm) and typically visualized with UV light or ceric ammonium molybdate stain. In some cases, an iodine, alkaline $KMnO_4$, or *p*-anisaldehyde stain was used.

2. Synthesis of Starting Materials

Alcohol S1:

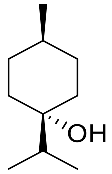

A solution of terpinen-4-ol (1.02 mL, 6.20 mmol, 1.00 equiv) and Crabtree's catalyst (12.5 mg, 15.5 µmol, 0.25 mol%) in dry dichloromethane (1.25 ml) was stirred under a hydrogen atmosphere (balloon) for 22 h at rt. After completion, the mixture was filtered through silica, and the silica pad was washed with dichloromethane (10 mL). The combined filtrate was concentrated under reduced pressure to give **S1** as a yellowish solid (814 mg, 5.21 mmol, 84%). **S1** was used without further purification.

The ^{13}C NMR spectra of **S1** matched those reported by Platzer.[2] Specifically, the resonance of C-7 at 20.7 ppm matched the literature value of 20.5 ppm for this isomer, rather than the 22.5 ppm value for the other isomer.

^{1}H NMR (500 MHz, $CDCl_3$) δ 1.92-1.86 (m, 1H), 1.85-1.74 (m, 2H), 1.72-1.60 (m, 2H), 1.60-1.51 (m, 1H), 1.30 (ddd, *J* = 13.7, 10.8, 3.8 Hz, 2H), 1.20-1.14 (br, 1H), 1.14-1.03 (m, 2H), 0.90 (dd, *J* = 6.7, 5.1 Hz, 9H) ppm.

^{13}C{^{1}H} NMR (126 MHz, $CDCl_3$) δ 73.4, 34.4, 31.5, 30.7, 30.6, 20.7, 16.3 ppm.

Ketone S2:

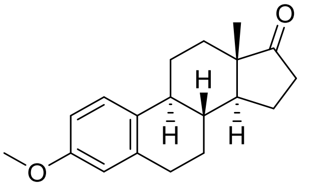

A suspension of estrone (500 mg, 1.85 mmol, 1.00 equiv), cesium carbonate (663 mg, 2.03 mmol, 1.10 equiv) and methyl iodide (598 µL, 9.25 mmol, 5.00 equiv) in 4.50 mL of dry acetonitrile was refluxed for 4 hours in a 20 mL vial. The residual methyl iodide was then removed under reduced pressure. The reaction mixture was cooled to room temperature, poured into 6 mL of cold water, and stirred for 30 minutes. The suspension was filtered on a fritted funnel and washed several times with water. The wet powder was dried *in vacuo* to give **S2** as a colorless solid (432 mg, 1.85 mmol, 82%).

^{1}H NMR (500 MHz, $CDCl_3$) δ 7.21 (d, *J* = 8.6 Hz, 1H), 6.72 (dd, *J* = 8.7, 2.8 Hz, 1H), 6.65 (d, *J* = 2.8 Hz, 1H), 3.78 (s, 3H), 2.98-2.81 (m, 2H), 2.50 (dd, *J* = 19.0, 8.7 Hz, 1H), 2.39 (dd, *J* = 9.1, 5.4 Hz, 1H), 2.29-2.22 (m, 1H), 2.21-1.88 (m, 4H), 1.72-1.36 (m, 6H), 0.91 (s, 3H) ppm.

^{13}C{^{1}H} NMR (126 MHz, $CDCl_3$) δ 221.1, 157.7, 137.9, 132.2, 126.5, 114.0, 111.7, 55.4, 50.6, 48.2, 44.1, 38.5, 36.0, 31.7, 29.8, 26.7, 26.1, 21.7, 14.0 ppm.

HRMS (ESI) calculated for $C_{19}H_{28}NO_2$ $[M+NH_4]^+$: 302.2120, Found: 302.2116

Alcohol S3:

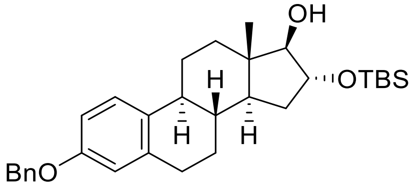

To a stirred solution of benzyl estriol (379 mg, 1.00 mmol, 1.00 equiv) and imidazole (102 mg, 1.50 mmol, 1.50 equiv) in DMF (1.00 mL) was added TBSCl (166 mg, 1.10 mmol, 1.1 equiv). After 16 hours, the solution was diluted with diethyl ether (50 mL) and water (50 mL). The aqueous layer was extracted with diethyl ether (3 x 50 mL), and the combined organic layers were dried over Na_2SO_4, filtered and concentrated under reduced pressure. The crude was purified by flash column chromatography (SiO_2, hexanes/EtOAc 1:0 to 4:1) to give **S3** as colorless solid (304 mg, 0.62 mmol, 62%).

^{1}H NMR (400 MHz, $CDCl_3$) δ 7.43 – 7.23 (m, 5H), 7.16 (d, *J* = 8.6 Hz, 1H), 6.75 (dd, *J* = 8.6, 2.8 Hz, 1H), 6.69 (d, *J* = 2.8 Hz, 1H), 4.99 (s, 2H), 4.12 – 4.03 (m, 1H), 3.54 (d, *J* = 5.3 Hz, 1H), 2.81 (t, *J* = 4.8 Hz, 2H), 2.31 – 2.15 (m, 2H), 1.90 – 1.71 (m, 4H), 1.56 (t, *J* = 7.5 Hz, 2H), 1.52 – 1.26 (m, 4H), 0.91 (s, 9H), 0.76 (s, 3H), 0.08 (s, 6H) ppm.

^{13}C{^{1}H} NMR (101 MHz, $CDCl_3$) δ 156.8, 138.0, 137.4, 132.9, 128.6, 127.9, 127.5, 126.3, 114.9, 112.4, 90.0, 79.3, 70.0, 47.7, 44.0, 43.4, 38.4, 36.6, 34.6, 29.8, 27.3, 26.1, 25.9, 18.3, 12.4, -4.4, -4.5 ppm.

HRMS (ESI): *m/z* for $C_{31}H_{45}O_3Si$ $[M+H]^+$ calcd.: 493.3132, found: 493.3111 (residual: 4.26 ppm).

Alcohol S4:

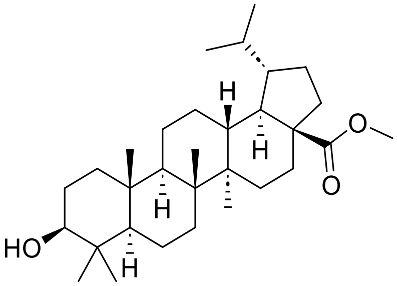

To a suspension of betulinic acid (500 mg, 1.09 mmol, 1.00 equiv) in MeOH (2.5 mL) and toluene (2.50 mL) was added trimethylsilyldiazomethane (820 µL, 2.0 M in Et_2O, 1.64 mmol, 1.50 equiv) at 0 °C. The reaction mixture was warmed to room temperature and stirred for 3 hours. Subsequently, all volatile materials were removed under reduced pressure and half of the residue (the other half was used for **S6**) was dissolved in ethyl acetate (8.0 mL) and MeOH (8.0 mL). Palladium on carbon (117 mg, 5 wt%, 0.055 mmol, 5 mol%) was added, and the mixture was stirred under a hydrogen atmosphere (balloon) for 17 hours. The suspension was filtered through Celite, and the Celite pad was washed with ethyl acetate. The filtrate was concentrated under reduced pressure and purified by flash column chromatography (SiO_2, hexanes/EtOAc 1:0 to 4:1) to give **S4** as a colorless solid (242 mg, 0.512 mmol, 47%).

^{1}H NMR (400 MHz, $CDCl_3$) δ 3.64 (s, 3H), 3.18 (dd, *J* = 11.2, 5.0 Hz, 1H), 2.28 – 2.13 (m, 3H), 1.83 – 1.73 (m, 2H), 1.71 – 1.55 (m, 4H), 1.47 (dddd, *J* = 13.1, 10.4, 7.3, 3.8 Hz, 4H), 1.40 – 1.31 (m, 5H), 1.30 – 1.23 (m, 2H), 1.22 – 1.09 (m, 3H), 0.95 (s, 3H), 0.93 (s, 3H), 0.89 (s, 3H), 0.84 (d, *J* = 6.8 Hz, 3H), 0.82 (s, 3H), 0.76 – 0.72 (m, 6H), 0.67 (d, *J* = 8.7 Hz, 1H) ppm.

$^{13}C\{^1H\}$ NMR (101 MHz, $CDCl_3$) δ 177.0, 79.1, 57.1, 55.5, 51.3, 50.4, 49.1, 44.3, 42.7, 40.8, 39.0, 38.9, 38.3, 37.5, 37.3, 34.5, 32.2, 29.9, 29.8, 28.1, 27.5, 27.1, 23.1, 22.9, 21.1, 18.4, 16.2, 16.1, 15.5, 14.8, 14.8 ppm.

HRMS (ESI): *m/z* for $C_{31}H_{52}O_3Na$ $[M+Na]^+$ calcd.: 495.3809, found: 495.3784.

Alcohol S5:

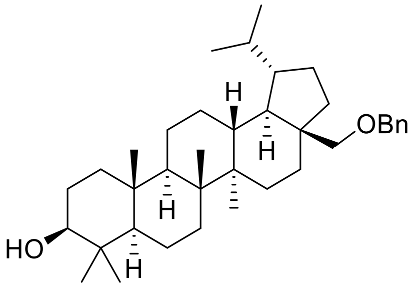

A suspension of betulin (200 mg, 0.452 mmol, 1.00 equiv) and palladium on carbon (96.2 mg, 5 wt%, 0.045 mmol, 10 mol%) in ethyl acetate (15.0 mL) and MeOH (15.0 mL) was stirred under a hydrogen atmosphere (balloon) for 21 hours. The mixture was filtered through Celite, and the Celite pad was washed with ethyl acetate. The combined filtrate was concentrated under reduced pressure. The residue was dissolved in a 2:1 hexanes/EtOAc mixture and filtered through a short plug. The filtrate was concentrated under reduced pressure to afford the crude dihydrobetulin as a colorless solid.

The crude dihydrobetulin and tetrabutylammonium iodide (1.7 mg, 4.5 µmol, 1.00 mol%) were dissolved in THF (4.5 mL). NaH (20 mg, 60% in mineral oil, 0.50 mmol, 1.10 equiv) was added, and the mixture was stirred for 30 minutes. The mixture was cooled to 0 °C, and benzyl bromide (53 µL, 0.45 mmol, 1.00 equiv) was added. The mixture was stirred for 14 hours at room temperature and 2 hours at 50 °C. Tetrabutylammonium iodide (6.8 mg, 18 µmol, 4.00 mol%) was added, and the mixture was stirred at 50 °C for 46 hours. The reaction was cooled to room temperature, and MeOH (3 mL) and water (50 mL) were added. The aqueous layer was extracted with ethyl acetate (3 x 50 mL), and the combined organic layers were dried over Na_2SO_4, filtered and concentrated under reduced pressure. The crude material was purified by flash column chromatography (SiO_2, hexanes/EtOAc 1:0 to 3:2) to give **S5** as a colorless solid (76.1 mg, 0.14 mmol, 31%).

^1H NMR (400 MHz, $CDCl_3$) δ 7.39-7.32 (m, 4H), 7.30 – 7.27 (m, 1H), 4.57 (d, J = 12.4 Hz, 1H), 4.46 (d, *J* = 12.4 Hz, 1H), 3.49 (dd, *J* = 9.0, 1.5 Hz, 1H), 3.19 (dd, *J* = 11.2, 4.9 Hz, 1H), 3.07 (d, *J* = 9.1 Hz, 1H), 2.06 – 1.91 (m, 2H), 1.86 – 1.79 (m, 2H), 1.75 – 1.62 (m, 3H), 1.57 – 1.50 (m, 5H), 1.46 – 1.29 (m, 8H), 1.28 – 1.22 (m, 3H), 1.21 – 1.10 (m, 3H), 0.97 (s, 3H), 0.92 (s, 3H), 0.86 – 0.82 (m, 6H), 0.81 (s, 3H), 0.77 – 0.72 (m, 6H), 0.67 (d, *J* = 9.3 Hz, 1H) ppm.

$^{13}C\{^1H\}$ NMR (101 MHz, $CDCl_3$) δ 139.3, 128.4, 127.6, 127.5, 79.1, 73.5, 68.2, 55.4, 50.2, 48.3, 47.5, 44.8, 42.9, 41.0, 39.0, 38.8, 37.2, 37.1, 35.1, 34.4, 30.3, 29.7, 28.1, 27.5, 27.1, 27.0, 23.1, 22.0, 20.9, 18.5, 16.2, 15.9, 15.5, 15.1, 14.8 ppm.

HRMS (EI): *m/z* for $C_{37}H_{58}O_2$ $[M]^+$ calcd.: 534.4437, found: 534.4410.

Alcohol S6:

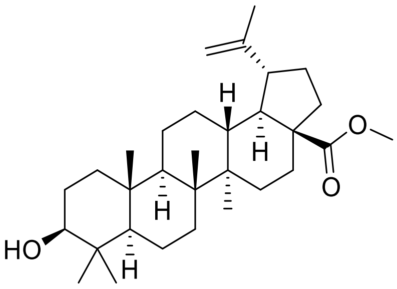

To a stirred suspension of betulinic acid (500 mg, 1.09 mmol, 1.00 equiv) in MeOH (2.50 mL) and toluene (2.50 mL) was added trimethylsilyldiazomethane (820 µL, 2.0 M in Et_2O, 1.64 mmol, 1.50 equiv) at 0 °C. The reaction mixture was warmed to room temperature and stirred for 3 hours. The volatile materials were removed under reduced pressure, and half of the residue (the other half was used for **S4**) was purified by flash column chromatography (SiO_2, hexanes/EtOAc 1:0 to 4:1) to give **S6** as colorless solid (156 mg, 0.32 mmol, 59%).

^{1}H NMR (400 MHz, $CDCl_3$) δ 4.71 (s, 1H), 4.57 (s, 1H), 3.64 (s, 3H), 3.16 (dd, *J* = 11.1, 5.1 Hz, 1H), 3.03 – 2.92 (m, 1H), 2.27 – 2.12 (m, 2H), 1.94 – 1.80 (m, 2H), 1.69 (s, 2H), 1.66 (s, 3H), 1.63 (s, 1H), 1.61 – 1.42 (m, 5H), 1.42 – 1.30 (m, 8H), 1.25 – 1.20 (m, 2H), 1.18 – 1.08 (m, 1H), 1.00 (d, *J* = 12.4 Hz, 1H), 0.94 (s, 6H), 0.89 (s, 3H), 0.80 (s, 3H), 0.73 (s, 3H), 0.65 (d, *J* = 8.7 Hz, 1H) ppm.

^{13}C{^{1}H} NMR (101 MHz, $CDCl_3$) δ 176.7, 150.6, 109.7, 79.0, 56.7, 55.5, 51.4, 50.7, 49.6, 47.1, 42.5, 40.8, 39.0, 38.8, 38.4, 37.3, 37.1, 34.4, 32.3, 30.7, 29.8, 28.1, 27.5, 25.6, 21.0, 19.5, 18.4, 16.2, 16.1, 15.5, 14.8 ppm.

HRMS (ESI): *m/z* for $C_{31}H_{51}O_3$ $[M+H]^+$ calcd.: 471.3833, found: 471.3813.

Alcohol S7:

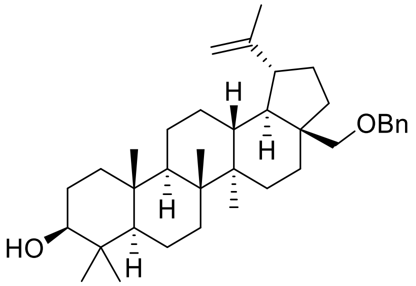

A suspension of betulin (443 mg, 1.00 mmol, 1.00 equiv) in THF (5.00 mL) was cooled to 0 °C. Then, NaH (48 mg, 60% in mineral oil, 1.20 mmol, 1.20 equiv) and benzyl bromide (131 µL, 1.10 mmol, 1.10 equiv) were added successively. The reaction mixture was warmed to room temperature and stirred for 36 hours. The mixture was cooled to 0 °C and MeOH (2 mL) and water (25 mL) were added. The aqueous layer was extracted with ethyl acetate (3 x 25 mL) and the combined organic layer was dried with Na_2SO_4, filtered, and concentrated under reduced pressure. Purification by flash column chromatography (SiO_2, hexanes/EtOAc 6:1 to 2:1) afforded **S7** as a colorless solid (139 mg, 0.261 mmol, 26%).

^{1}H NMR (600 MHz, $CDCl_3$) δ 7.39-7.32 (m, 4H), 7.31 – 7.26 (m, 1H), 4.70 – 4.45 (m, 4H), 3.51 (dd, *J* = 9.0, 1.7 Hz, 1H), 3.18 (dd, *J* = 11.5, 4.7 Hz, 1H), 3.10 (d, *J* = 8.9 Hz, 1H), 2.36 (td, *J* = 11.0, 5.8 Hz, 1H), 2.10 – 1.95 (m, 2H), 1.96 – 1.85 (m, 1H), 1.64 – 1.47 (m, 8H), 1.45 – 1.30 (m,

6H), 1.29-1.22 (m, 2H), 1.17 (qd, J = 13.3, 4.5 Hz, 2H), 1.09 – 1.02 (m, 3H), 1.02 – 0.93 (m, 7H), 0.93 – 0.74 (m, 11H), 0.71 – 0.64 (m, 1H) ppm.

^{13}C{^{1}H} NMR (151 MHz, $CDCl_3$) δ 150.9, 139.1, 128.4, 127.7, 127.6, 109.6, 79.1, 73.5, 68.2, 55.4, 50.5, 49.0, 48.1, 47.4, 42.8, 41.0, 39.0, 38.8, 37.6, 37.3, 35.0, 34.3, 30.2, 30.1, 28.1, 27.5, 27.3, 25.3, 21.0, 19.2, 18.5, 16.2, 15.9, 15.5, 14.9 ppm.

HRMS (ESI): *m/z* for $C_{37}H_{57}O_2$ $[M+H]^+$ calcd.: 533.4353, found: 533.4370.

Alcohol S8:

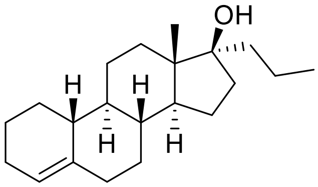

Allylestrenol (150 mg, 0.500 mmol, 1.00 equiv) was dissolved in CH_2Cl_2 (5.00 mL) and $Rh(PPh_3)_3Cl$ (46.3 mg, 0.050 mmol, 10 mol%) was added. The mixture was stirred under a hydrogen atmosphere (balloon) for 29 hours at room temperature and after completion, the suspension was filtered through Celite, and the Celite pad was washed with CH_2Cl_2. The filtrate was concentrated under reduced pressure and purified by flash column chromatography (SiO_2, hexanes/EtOAc 1:0 to 8:2) to afford **S8** as a colorless solid (131 mg, 0.433 mmol, 87%).

^{1}H NMR (500 MHz, $CDCl_3$) δ 5.38 (br s, 1H), 2.19 (ddd, J = 13.6, 4.1, 2.6 Hz, 1H), 2.07 – 1.86 (m, 5H), 1.83 (dtd, J = 13.5, 4.1, 2.8 Hz, 1H), 1.78 – 1.72 (m, 1H), 1.68 (dtq, J = 12.4, 4.7, 2.8 Hz, 2H), 1.58 – 1.13 (m, 14H), 1.08 (tdd, J = 12.7, 9.4, 2.9 Hz, 1H), 0.94 (t, J = 7.0 Hz, 3H), 0.89 (s, 3H), 0.88 – 0.78 (m, 1H), 0.62 (dtd, J = 11.7, 10.1, 4.2 Hz, 1H) ppm.

^{13}C{^{1}H} NMR (126 MHz, $CDCl_3$) δ 140.6, 120.0, 83.6, 50.4, 49.6, 46.7, 42.2, 42.1, 39.3, 35.7, 34.5, 32.1, 31.7, 28.9, 26.2, 25.6, 23.7, 22.2, 16.9, 15.1, 14.5 ppm.

HRMS (ESI) calculated for $C_{42}H_{68}NaO_2$ $[2M+Na]^+$: 627.5117, Found: 627.5110

3. Silylation and Oxidation of Terpenoids

General procedure for Ir-catalyzed silylation of alcohols and ketones (GP-1)

In a nitrogen-filled glovebox, the alcohol or ketone (1.0 equiv) and $[Ir(COD)OMe]_2$ (0.05–0.20 mol%, added as a stock solution in THF) were weighed into a one-dram screw-capped vial. A magnetic stir bar was added, and the solids were dissolved in THF (0.5 M). The resulting solution was treated with neat Et_2SiH_2 (1.2 equiv). Effervescence was often observed in large-scale reactions with alcohols from the formation of dihydrogen. The vial was capped with a Teflon-lined screw cap, sealed with electrical tape, and removed from the glovebox. The vial was placed in a pre-heated aluminum heating block at room temperature or 50 °C and stirred until complete conversion to the corresponding diethyl(hydrido)silyl ether was observed, as determined by GC or GC-MS analysis. TLC analysis was used in the case of some triterpenoids with very high boiling points.

General procedure for Ru-catalyzed silylation of alcohols (GP-2)

In a nitrogen-filled glovebox, the alcohol or ketone (1.0 equiv) and $Ru(PPh_3)_3Cl_2$ (0.2 mol%) were weighed into a one-dram screw-capped vial. A magnetic stir bar was added, and the solids were dissolved in benzene (0.5 M). The resulting solution was treated with neat Et_2SiH_2 (1.2 equiv). The vial was capped with a Teflon-lined screw cap, sealed with electrical tape, and removed from the glovebox. The vial was placed in a pre-heated aluminum heating block at 50 °C and stirred until complete conversion to the corresponding diethyl(hydrido)silyl ether was observed, as determined by GC or GC-MS analysis. TLC analysis was used to assess conversion for some triterpenoids with high boiling points.

General procedure for Ir-catalyzed intramolecular aliphatic silylation (GP-3)

The crude reaction mixture containing the diethyl(hydrido)silyl ether and solvent was evaporated on a rotary evaporator (the magnetic stir bar was temporarily removed during this operation to prevent bumping) and then transferred to a nitrogen-filled glovebox. The stir bar was replaced, and the concentrated diethyl(hydrido)silyl ether was then sequentially treated with $[Ir(COD)OMe]_2$ (1.0 mol%, added as a stock solution in THF for reactions run on a small scale), Me_4phen (2.5 mol%), norbornene (1.2 equiv), and THF (0.2 M). The Teflon-lined screw cap was replaced, the vial was sealed with electrical tape, and the resulting solution was stirred in the glovebox for 10 min to ensure complete formation of the active Ir species (a dark green solution is typically observed). The vial was then removed from the glovebox, placed in a pre-heated aluminum block at 120 °C, and stirred for 21 h.

General procedure A for Tamao-Fleming oxidation of oxasilolanes (GP-4)

On the bench top, the crude reaction mixture containing the oxasilolane in THF was transferred to a round-bottomed flask and sequentially treated with MeOH (0.25 M), $KHCO_3$ (2.5 equiv) and H_2O_2 (50% solution in H_2O, 10 equiv). The flask was sealed with a septum, and a balloon was attached (the reaction generates carbon dioxide). The resulting mixture was stirred overnight at 50 °C. The reaction was carefully quenched with sat. aq. $Na_2S_2O_3$ and sat. aq. $NaHCO_3$ (1:1 v/v). and the resulting mixture was extracted with EtOAc. The combined organic layers were washed with 1 M HCl (3 times) and sat. aq. $NaHCO_3$, dried over $MgSO_4$, and concentrated by rotary evaporation. The crude diol was purified by column chromatography on silica gel to give the desired products.

General procedure B for Tamao-Fleming oxidation of oxasilolanes (GP-5)

On the bench top, the crude reaction mixture containing the oxasilolane in THF (2 mL) was concentrated by rotary evaporation. The crude product was sequentially treated with DMF (0.1 M), $CsOH{\cdot}H_2O$ (12 equiv), *t*-butyl hydroperoxide (70% in H_2O, 14 equiv) and TBAF (1.0 M in THF, 5 equiv). The vial was sealed with a Teflon-lined screw cap, and the resulting mixture was stirred overnight at 80 °C. The reaction was carefully quenched with sat. Na_2SO_3, and the resulting mixture was extracted with EtOAc. The combined organic layers were washed with sat. Na_2SO_3, 5% HCl, and brine, dried over $MgSO_4$, and concentrated by rotary evaporation. The crude diol was purified by column chromatography on silica gel to give the desired products.

Compound 2a:

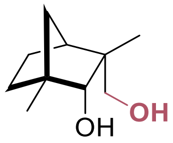

The synthesis of the corresponding diacetate (66% yield) had previously been reported by Simmons and Hartwig.[1]

Following **GP-1**, (+)-fenchol (321 mg, 2.00 mmol, 1.00 equiv) was converted to the corresponding diethyl(hydrido)silyl ether with $[Ir(COD)OMe]_2$ (1.00 mL of a 1.00 mM stock solution in THF, 1.00 µmol, 0.05 mol%) as catalyst and Et_2SiH_2 (311 µL, 2.40 mmol, 1.20 equiv) at room temperature for 23 h. The subsequent C-H silylation (**GP-3**) was conducted with $[Ir(COD)OMe]_2$ (13.3 mg, 0.020 mmol, 1.0 mol%) and Me_4phen (11.8 mg, 0.050 mmol, 2.5 mol%) at 100 °C for 19 h to provide the intermediate oxasilolane. Tamao-Fleming oxidation (**GP-4**) and purification by flash column chromatography (SiO_2, hexanes/EtOAc 9:1 to 3:2) gave **2a** as a colorless solid (233 mg, 1.37 mmol, 68%).

^{1}H NMR (500 MHz, $CDCl_3$) δ 3.93 (d, *J* = 10.7 Hz, 1H), 3.42 (d, *J* = 1.8 Hz, 1H), 3.35 (d, *J* = 10.8 Hz, 1H), 2.06 (broad singlet, 2H).1.80 (dddd, *J* = 11.8, 8.6, 6.1, 2.1 Hz, 1H), 1.73 (d, *J* = 3.8 Hz, 1H), 1.62 (ddd, *J* = 12.2, 9.1, 3.1 Hz, 1H), 1.50 (dt, *J* = 10.3, 2.1 Hz, 1H), 1.41 (tdd, *J* = 12.6, 6.2, 4.0 Hz, 1H), 1.16 (dd, *J* = 10.3, 1.6 Hz, 1H), 1.10 (d, 6H), 1.08 – 1.02 (m, 1H).

^{13}C{^{1}H} NMR (126 MHz, $CDCl_3$) δ 85.9, 66.8, 49.5, 46.2, 43.5, 42.0, 25.5, 25.3, 25.3, 19.2.

HRMS (ESI) *m/z* for $C_{10}H_{19}O_2$ $[M+H]^+$ calcd.: 171.1385, found: 171.1379.

Compound 2b:

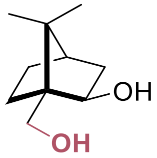

The synthesis of the corresponding diacetate (57% yield) had previously been reported by Simmons and Hartwig.[1]

Following **GP-1**, (+)-camphor (311 mg, 2.00 mmol, 1.00 equiv) was converted to the corresponding diethyl(hydrido)silyl ether with $[Ir(COD)OMe]_2$ 2 (1.00 mL of a 1.00 mM stock solution in THF, 1.00 µmol, 0.05 mol%) as catalyst and Et_2SiH_2 (311 µL, 2.40 mmol, 1.20 equiv) at room temperature for 21 h. The subsequent C-H silylation (**GP-3**) was conducted with

[Ir(COD)OMe]$_2$ (13.3 mg, 0.020 mmol, 1.0 mol%) and Me$_4$phen (11.8 mg, 0.050 mmol, 2.5 mol%) at 120 °C for 21 h to provide the intermediate oxasilolane. Tamao-Fleming oxidation (**GP-4**) and purification by flash column chromatography (SiO_2, hexanes/EtOAc 9:1 to 1:1) gave **2b** as a colorless solid (218 mg, 1.28 mmol, 64%).

^{1}H NMR (500 MHz, $CDCl_3$) δ 4.01 (dd, *J* = 7.9, 3.7 Hz, 1H), 3.93 (d, *J* = 11.1 Hz, 1H), 3.78 (d, *J* = 11.1 Hz, 1H), 2.00 (broad singlet, 2H), 1.88 – 1.79 (m, 1H), 1.78 – 1.66 (m, 3H), 1.53 – 1.43 (m, 1H), 1.20 (s, 3H), 1.12 (ddd, *J* = 12.7, 9.7, 3.7 Hz, 1H), 1.08 – 1.01 (m, 1H), 0.90 (s, 3H).

^{13}C{^{1}H} NMR (126 MHz, $CDCl_3$) δ 79.0, 63.6, 53.1, 46.6, 46.2, 40.7, 30.2, 27.0, 21.3, 20.8.

HRMS (ESI) *m/z* for $C_{20}H_{37}O_4$ $[2M+H]^+$ calcd.: 341.2692, found: 341.2685.

Compound 2c:

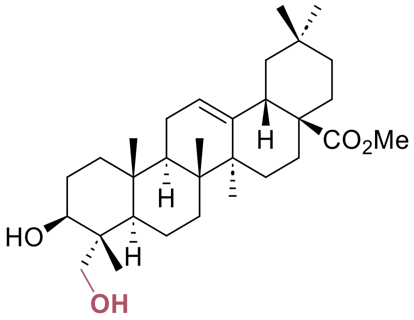

The synthesis of this compound (61% yield) had previously been reported by Simmons and Hartwig.[1] It was successfully synthesized according to their procedure (following **GP-1**, **GP-3**, and **GP-4**).

Compound 2d:

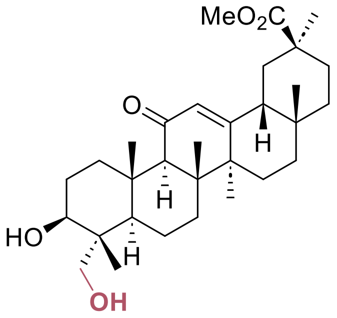

The synthesis of this compound (51% yield) had previously been reported by Simmons and Hartwig.[1] It was successfully synthesized according to their procedure (following **GP-2**, **GP-3**, and **GP-4**).

Compound 2e:

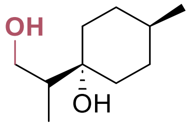

Following **GP-1**, dihydroterpinen-4-ol **S1** (156 mg, 1.00 mmol, 1.00 equiv) was converted to the corresponding diethyl(hydrido)silyl ether with $[Ir(COD)OMe]_2$ (0.500 mL of a 1.00 mM stock solution in THF, 0.50 μmol, 0.05 mol%) as catalyst and Et_2SiH_2 (155 μL, 1.20 mmol, 1.20 equiv) at room temperature for 23 h. The subsequent C-H silylation (**GP-3**) was conducted with $[Ir(COD)OMe]_2$ (3.31 mg, 5.0 μmol, 0.50 mol%) and Me_4phen (3.0 mg, 12.5 μmol, 1.25 mol%) at 100 °C for 19 h to provide the intermediate oxasilolane. Tamao-Fleming oxidation (**GP-5**) and purification by flash column chromatography (SiO_2, hexanes/EtOAc 4:1 to 3:1) gave **2e** as a colorless oil (150 mg, 0.871 mmol, 87%).

^{1}H NMR (500 MHz, $CDCl_3$) δ 4.01 (dd, *J* = 11.0, 3.2 Hz, 1H), 3.65 (dd, *J* = 10.9, 3.7 Hz, 1H), 2.25 (broad singlet, 2H), 2.03 (ddd, *J* = 13.1, 5.9, 3.0 Hz, 1H), 1.94 – 1.80 (m, 2H), 1.79 – 1.62 (m, 2H), 1.62 – 1.51 (m, 1H), 1.43 (ddd, *J* = 13.3, 11.1, 3.8 Hz, 1H), 1.30 (ddd, *J* = 13.1, 11.0, 3.8 Hz, 1H), 1.07 (d, *J* = 7.2 Hz, 5H), 0.91 (d, *J* = 6.7 Hz, 3H) ppm.

^{13}C{^{1}H} NMR (126 MHz, $CDCl_3$) δ 75.3, 65.5, 36.8, 35.8, 34.7, 30.9, 30.8, 30.7, 20.8, 11.6 ppm.

HRMS (ESI): *m/z* for $C_{10}H_{20}O_2Na$ $[M+Na]^+$ calcd.: 195.1356, found: 195.1356.

Compound 2f:

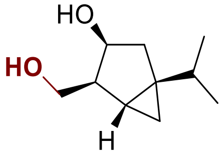

Following **GP-1**, (-)-α-thujone (83 μL, 0.500 mmol, 1.00 equiv) was converted to the corresponding diethyl(hydrido)silyl ether with $[Ir(COD)OMe]_2$ (1.00 mL of a 1.00 mM stock solution in THF, 1.00 μmol, 0.20 mol%) as catalyst and Et_2SiH_2 (78 μL, 0.60 mmol, 1.20 equiv) at room temperature for 24 h. The subsequent C-H silylation (**GP-3**) was conducted with $[Ir(COD)OMe]_2$ (3.3 mg, 5.0 μmol, 1.0 mol%) and Me_4phen (3.0 mg, 12.5 μmol, 2.5 mol%) at 120 °C for 20 h to provide the intermediate oxasilolane. Tamao-Fleming oxidation (**GP-4**) and purification by flash column chromatography (SiO_2, hexanes/EtOAc 9:1 to 1:1) gave **2f** as a colorless solid (45.0 mg, 0.264 mmol, 53%).

^{1}H NMR (500 MHz, $CDCl_3$) δ 4.16 (q, *J* = 7.6 Hz, 1H), 3.82 (dd, *J* = 11.1, 9.0 Hz, 1H), 3.65 (dd, *J* = 11.1, 3.7 Hz, 1H), 2.33 (ddd, *J* = 9.6, 6.9, 3.6 Hz, 1H), 2.02 (dd, *J* = 12.3, 7.4 Hz, 1H), 1.72 (dd, *J* = 12.3, 8.7 Hz, 1H), 1.25 (quint, *J* = 6.8 Hz, 1H), 0.95 (d, *J* = 6.8 Hz, 3H), 0.89 (d, *J* = 6.9 Hz, 3H), 0.75 (dd, *J* = 8.2, 4.0 Hz, 1H), 0.34 – 0.26 (m, 2H) ppm.

^{13}C{^{1}H} NMR (126 MHz, $CDCl_3$) δ 74.2, 64.7, 44.7, 34.8, 33.3, 32.2, 23.2, 20.2, 19.7, 14.5.

HRMS (ESI) *m/z* for $C_{10}H_{18}NaO_2$ $[M+Na]^+$ calcd.: 193.1204, found: 193.1210.

Compound 2g and 2h:

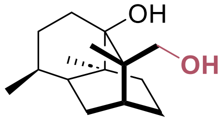

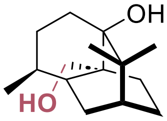

2g **2h**

Following **GP-1**, patchouli alcohol (111 mg, 0.500 mmol, 1.00 equiv) was converted to the corresponding diethyl(hydrido)silyl ether with $[Ir(COD)OMe]_2$ (1.00 mL of a 1.00 mM stock solution in THF, 1.00 μmol, 0.20 mol%) as catalyst and Et_2SiH_2 (78 μL, 0.600 mmol, 1.20 equiv) at room temperature for 24 h. The subsequent C-H silylation was conducted with $[Rh(COD)_2Cl]_2$ (4.9 mg, 10.0 μmol, 2.0 mol%) and Xantphos (12.7 mg, 22.0 μmol, 4.4 mol%) at 120 °C for 24 h to provide the intermediate oxasilolane. Tamao-Fleming oxidation (**GP-4**) and purification by flash column chromatography (SiO_2, hexanes/EtOAc 9:1 to 1:1) gave **2g** (46.7 mg, 0.196 mmol, 39%) and **2h** (15.5 mg, 0.065 mmol, 13%) as colorless solids (52% total, 3:1).

2g:

The ^{1}H and ^{13}C NMR spectra of this compound were identical to those reported by Sarpong.[3]

^{1}H NMR (700 MHz, $CDCl_3$) δ 4.25 (d, *J* = 10.7 Hz, 1H), 3.10 (d, *J* = 10.7 Hz, 1H), 2.01 – 1.92 (m, 2H), 1.82 – 1.78 (m, 1H), 1.77 – 1.70 (m, 1H), 1.55 – 1.43 (m, 3H), 1.42 – 1.34 (m, 2H), 1.32 – 1.19 (m, 6H), 1.06 (ddd, *J* = 13.8, 11.8, 2.2 Hz, 1H), 0.87 (s, 3H), 0.80 (d, *J* = 6.7 Hz, 3H) ppm.

^{13}C{^{1}H} NMR (151 MHz, $CDCl_3$) δ 77.7, 71.6, 44.0, 43.3, 38.2, 36.6, 34.5, 29.0, 28.8, 28.2, 25.0, 23.3, 20.1, 18.7, 18.6 ppm.

2h:

^{1}H NMR (700 MHz, $CDCl_3$) δ 4.34 (d, *J* = 11.3 Hz, 1H), 3.19 (d, *J* = 11.3 Hz, 1H), 2.73 (broad singlet, 1H), 2.29 (ddd, *J* = 14.0, 11.6, 7.6 Hz, 1H), 2.07 – 1.91 (m, broad singlet overlapping with triplet, 2H), 1.83 – 1.69 (m, 3H), 1.61 – 1.46 (m, 3H), 1.44 – 1.35 (m, 1H), 1.34 – 1.23 (m, 3H), 1.18 (ddd, *J* = 13.9, 11.8, 2.0 Hz, 1H), 1.08 (s, 3H), 1.08 (s, 3H), 0.77 (d, *J* = 6.7 Hz, 3H).

^{13}C{^{1}H} NMR (126 MHz, $CDCl_3$) δ 78.6, 68.7, 41.2, 40.7, 40.4, 38.9, 32.9, 28.7, 27.6, 26.9, 24.5, 24.3, 23.7, 23.0, 18.6 ppm.

HRMS (ESI) *m/z* for $C_{16}H_{31}O_3$ $[M+MeOH+H]^+$ calcd.: 271.2273, Found: 271.2267

Compound 2i:

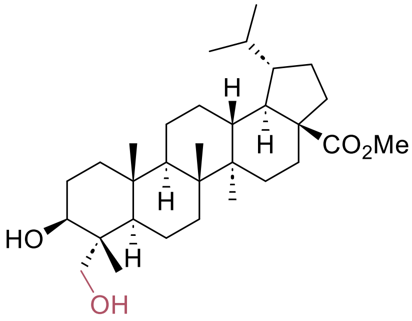

Following **GP-1**, **S4** (398 mg, 0.868 mmol, 1.00 equiv) was converted to the corresponding diethyl(hydrido)silyl ether with $[Ir(COD)OMe]_2$ (0.870 mL of a 1.00 mM stock solution in THF, 0.870 μmol, 0.10 mol%) as catalyst and Et_2SiH_2 (131 μL, 1.04 mmol, 1.20 equiv) at 50 °C for 16 h. The

subsequent C-H silylation (**GP-3**) was conducted with $[Ir(COD)OMe]_2$ (5.6 mg, 8.70 µmol, 1.0 mol%) and Me_4phen (5.1 mg, 21.6 µmol, 2.5 mol%) at 120 °C for 24 h to provide the intermediate oxasilolane. Subsequently, volatile materials were removed under reduced pressure and the crude product was used in the next step without further purification. Tamao-Fleming oxidation (**GP-4**) and purification by flash column chromatography (SiO_2, hexanes/EtOAc 1:0 to 2:3) to give **2i** as a colorless solid (26.8 mg, 0.055 mmol, 72%).

^{1}H NMR (400 MHz, $CDCl_3$) δ 3.68 (d, *J* = 10.4 Hz, 1H), 3.63 (s, 3H), 3.59 (d, *J* = 7.9 Hz, 1H), 3.39 (d, *J* = 10.3 Hz, 1H), 2.31 – 2.11 (m, 3H), 1.81 – 1.73 (m, 2H), 1.71 – 1.56 (m, 4H), 1.52 – 1.40 (m, 3H), 1.40 – 1.35 (m, 2H), 1.35 – 1.26 (m, 7H), 1.24 (s, 2H), 1.22 – 1.06 (m, 3H), 0.92 (s, 3H), 0.89 (s, 3H), 0.87 – 0.81 (m, 9H), 0.73 (d, *J* = 6.7 Hz, 3H) ppm.

^{13}C{^{1}H} NMR (101 MHz, $CDCl_3$) δ 177.0, 76.8, 72.0, 57.1, 51.3, 50.4, 50.0, 49.0, 44.3, 42.7, 42.0, 40.8, 38.5, 38.2, 37.4, 37.2, 34.3, 29.9, 29.8, 27.0, 23.1, 22.9, 21.0, 18.6, 16.6, 16.1, 14.8, 14.8 ppm.

HRMS (ESI): *m/z* for $C_{31}H_{52}O_4$ $[M]^+$ calcd.: 488.3866, found: 488.3857.

Compound 2j:

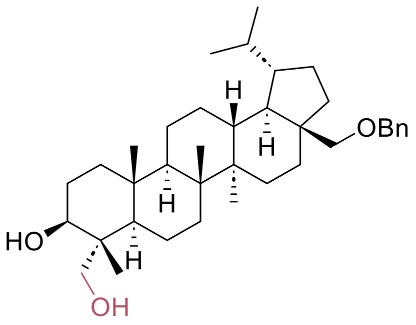

Following **GP-1**, **S5** (53.5 mg, 0.100 mmol, 1.00 equiv) was converted to the corresponding diethyl(hydrido)silyl ether with $[Ir(COD)OMe]_2$ (0.200 mL of a 1.00 mM stock solution in THF, 0.200 µmol, 0.20 mol%) as catalyst and Et_2SiH_2 (15.5 µL, 0.120 mmol, 1.20 equiv) at room temperature for 16 h. The subsequent C-H silylation (**GP-3**) was conducted with $[Ir(COD)OMe]_2$ (1.00 mL of a 1.00 mM stock solution in THF, 1.00 µmol, 1.0 mol%) and Me_4phen (0.6 mg, 2.50 µmol, 2.5 mol%) at 120 °C for 25 h to provide the intermediate oxasilolane. Tamao-Fleming oxidation (**GP-4**) and purification by flash column chromatography (SiO_2, hexanes/EtOAc 1:0 to 1:1) gave **2j** as a colorless solid (25.5 mg, 0.0463 mmol, 46%).

^{1}H NMR (600 MHz, $CDCl_3$) δ 7.37 – 7.30 (m, 4H), 7.29 – 7.26 (m, 1H), 4.56 (d, *J* = 12.4 Hz, 1H), 4.45 (d, *J* = 12.3 Hz, 1H), 3.72 (d, *J* = 10.3 Hz, 1H), 3.62 (t, *J* = 8.2 Hz, 1H), 3.48 (d, *J* = 9.2 Hz, 1H), 3.42 (d, *J* = 10.3 Hz, 1H), 3.06 (d, *J* = 9.0 Hz, 1H), 2.04 – 1.91 (m, 2H), 1.86 – 1.76 (m, 1H), 1.67 (d, *J* = 13.8 Hz, 3H), 1.61 (d, *J* = 9.7 Hz, 2H), 1.57 – 1.48 (m, 5H), 1.46 – 1.34 (m, 5H), 1.32 – 1.23 (m, 6H), 1.19 – 1.09 (m, 2H), 0.91 (s, 3H), 0.87 (s, 3H), 0.85 (s, 3H), 0.84 (s, 3H), 0.82 (d, *J* = 6.7 Hz, 3H), 0.74 (d, *J* = 6.7 Hz, 3H) ppm.

^{13}C{^{1}H} NMR (151 MHz, $CDCl_3$) δ 139.4, 128.4, 127.7, 127.5, 76.8, 73.6, 72.1, 68.4, 50.3, 50.0, 48.4, 47.6, 45.0, 43.0, 42.2, 41.1, 38.6, 37.2, 35.1, 34.3, 30.3, 29.9, 29.7, 27.3, 27.0, 23.1, 22.1, 21.0, 18.7, 16.5, 16.0, 15.1, 14.9, 11.4 ppm.

HRMS (EI): *m/z* for $C_{37}H_{58}O_3$ $[M]^+$ calcd.: 550.4386, found: 550.4360.

Compound 2k:

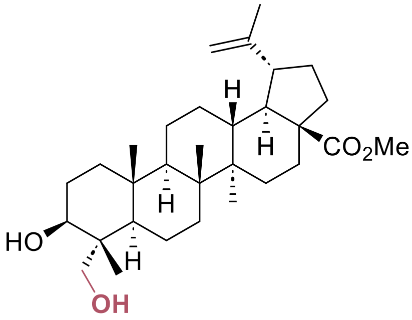

Following **GP-1**, **S6** (47.0 mg, 0.100 mmol, 1.00 equiv) was converted to the corresponding diethyl(hydrido)silyl ether with [Ir(COD)OMe]$_2$ (0.200 mL of a 1.00 mM stock solution in THF, 0.200 µmol, 0.20 mol%) as catalyst and Et_2SiH_2 (15.5 µL, 0.120 mmol, 1.20 equiv) at room temperature for 12 h. The subsequent C-H silylation (**GP-3**) was conducted with [Ir(COD)OMe]$_2$ (1.00 mL of a 1.00 mM stock solution in THF, 1.00 µmol, 1.00 mol%) and Me$_4$phen (0.6 mg, 2.5 µmol, 2.5 mol%) at 120 °C for 24 h to provide the intermediate oxasilolane. Subsequently, volatile materials were removed under reduced pressure and the crude product was used in the next step without further purification. The crude product was dissolved in DMF (0.800 mL). Subsequently, to the solution was added KF (14.5 mg, 0.250 mmol, 2.50 equiv), $KHCO_3$ (25.0 mg, 0.250 mmol, 2.50 equiv) and H_2O_2 (57 µL, 50 wt% in H_2O, 1.00 mmol, 10.0 equiv) and the solution was stirred at room temperature overnight. The reaction was quenched by carefully adding 0.6 mL sat. $Na_2S_2O_3$/$NaHCO_3$ (1:1) solution and extracted with EtOAc (3 x 10 mL), the organic layers were combined and dried over Na_2SO_4 and concentrated under reduced pressure. The crude mixture was purified by flash column chromatography (SiO_2, hexanes/EtOAc 1:0 to 2:3) to give **2k** as a colorless solid. (12.0 mg, 0.025 mmol, 25%).

^{1}H NMR (400 MHz, $CDCl_3$) δ 4.73 (s, 1H), 4.59 (s, 1H), 3.68 (d, *J* = 10.4 Hz, 1H), 3.66 (s, 3H), 3.65 – 3.56 (m, 1H), 3.39 – 3.34 (m, 3H), 3.01 – 2.96 (m, 1H), 2.25 – 2.13 (m, 2H), 1.94 – 1.82 (m, 2H), 1.68 (s, 3H), 1.64 – 1.55 (m, 2H), 1.40 – 1.35 (m, 4H),), 1.34 – 1.31 (m, 9H), 1.25 (s, 5H), 0.95 (s, 3H), 0.91 (s, 3H), 0.86 (s, 3H), 0.85 (s, 3H) ppm.

^{13}C{^{1}H} NMR (101 MHz, $CDCl_3$) δ 176.8, 150.7, 109.7, 71.8, 58.8, 56.7, 51.4, 50.7, 50.0, 49.6, 47.1, 42.6, 42.1, 40.8, 38.6, 38.4, 37.3, 34.2, 32.3, 30.8, 29.8, 27.1, 25.6, 21.0, 19.5, 18.6, 16.6, 16.1, 14.9, 11.5, 8.5 ppm.

HRMS (ESI): *m/z* for $C_{31}H_{50}O_4$ $[M]^+$ calcd.: 486.3709, found: 486.3704.

Compound 2l:

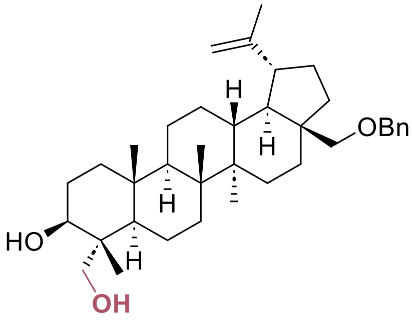

Following **GP-1**, **S7** (53.2 mg, 0.100 mmol, 1.00 equiv) was converted to the corresponding diethyl(hydrido)silyl ether with [Ir(COD)OMe]$_2$ (0.200 mL of a 1.00 mM stock solution in THF, 0.200 µmol, 0.20 mol%) as catalyst and Et_2SiH_2 (15.5 µL, 0.120 mmol, 1.20 equiv) at room temperature

for 16 h. The subsequent C-H silylation (**GP-3**) was conducted with $[Ir(COD)OMe]_2$ (1.00 mL of a 1.00 mM stock solution in THF, 1.00 µmol, 1.00 mol%) and Me_4phen (0.6 mg, 2.5 µmol, 2.5 mol%) at 120 °C for 24 h to provide the intermediate oxasilolane. Tamao-Fleming oxidation (**GP-4**) and purification by flash column chromatography (SiO_2, hexanes/EtOAc 9:1 to 3:2) gave **2l** as a colorless solid (17.3 mg, 0.0315 mmol, 32%).

^{1}H NMR (400 MHz, $CDCl_3$) δ 7.38 – 7.32 (m, 4H), 7.31 – 7.27 (m, 1H), 4.67 – 4.52 (m, 3H), 4.46 (d, *J* = 12.4 Hz, 1H), 3.71 (d, *J* = 10.3 Hz, 1H), 3.61 (t, *J* = 8.1 Hz, 1H), 3.50 (d, *J* = 8.9 Hz, 1H), 3.43 – 3.34 (m, 1H), 3.09 (d, *J* = 8.9 Hz, 1H), 2.41 – 2.29 (m, 1H), 2.07 – 1.94 (m, 2H), 1.92 – 1.80 (m, 1H), 1.64 – 1.45 (m, 8H), 1.42 – 1.32 (m, 4H), 1.31 – 1.21 (m, 10H), 1.18 – 1.11 (m, 2H), 1.02 (t, *J* = 10.7 Hz, 2H), 0.93 (s, 3H), 0.87 (s, 3H), 0.84 (s, 3H), 0.83 (s, 3H) ppm.

^{13}C{^{1}H} NMR (151 MHz, $CDCl_3$) δ 150.9, 139.1, 128.5, 127.7, 127.6, 109.6, 73.5, 72.2, 68.2, 50.5, 50.0, 49.0, 48.1, 47.4, 42.8, 42.0, 41.0, 38.5, 37.5, 37.2, 35.0, 34.1, 30.1, 30.1, 29.9, 27.3, 27.2, 25.3, 21.0, 19.2, 18.6, 16.6, 15.9, 15.0, 11.4 ppm.

HRMS (ESI): *m/z* for $C_{37}H_{56}O_3Na$ $[M+Na]^+$ calcd.: 571.4122, found: 571.4151.

Compound 2m and 2n:

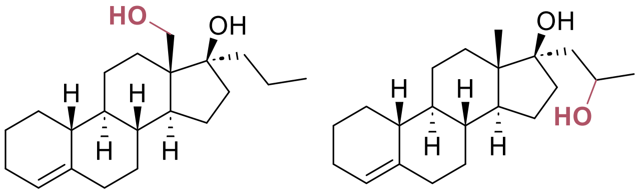

2m **2n**

Inseparable mixture (4:1)

Following **GP-1**, **S8** (103 mg, 0.340 mmol, 1.00 equiv) was converted to the corresponding diethyl(hydrido)silyl ether with $[Ir(COD)OMe]_2$ (0.340 mL of a 1.00 mM stock solution in THF, 0.340 µmol, 0.10 mol%) as catalyst and Et_2SiH_2 (15.5 µL, 0.120 mmol, 1.20 equiv) at room temperature for 16 h. The subsequent C-H silylation (**GP-3**) was conducted with $[Ir(COD)OMe]_2$ (2.3 mg, 3.40 µmol, 1.0 mol%) and Me_4phen (2.0 mg, 8.50 µmol, 2.5 mol%) at 120 °C for 21 h to provide the intermediate oxasilolane. Tamao-Fleming oxidation (**GP-5**) and purification by flash column chromatography (SiO_2, hexanes/EtOAc 9:1 to 7:3) gave an inseparable mixture of **2m** and **2n** as a colorless solid (62.1 mg, 0.195 mmol, 57%, 4:1).

15a:

^{1}H NMR (500 MHz, $CDCl_3$) δ 5.38 (s, 1H, overlapping), 3.90 (d, *J* = 11.5 Hz, 1H), 3.83 (d, *J* = 11.5 Hz, 1H), 2.77 (br s, 2H, overlapping), 2.32 – 1.02 (m, 24H, overlapping), 0.97 (t, *J* = 6.9 Hz, 3H), 0.86 – 0.73 (m, 1H, overlapping), 0.72 – 0.55 (m, 1H, overlapping) ppm.

^{13}C{^{1}H} NMR (126 MHz, $CDCl_3$) δ 140.4, 120.2, 86.4, 62.1, 50.4, 49.9 (overlapping), 49.8 (overlapping), 42.1 (overlapping), 41.5, 39.9, 35.6 (overlapping), 35.5 (overlapping), 32.3, 28.9, 26.4, 25.7, 25.6, 24.1, 22.2, 16.3, 14.9 ppm.

15a´:

^{1}H NMR (500 MHz, $CDCl_3$) δ 5.38 (s, 1H, overlapping), 4.26 – 4.19 (m, 1H), 2.77 (br s, 2H, overlapping), 2.32 – 1.02 (m, 24H, overlapping), 0.89 (s, 3H), 0.86 – 0.73 (m, 1H, overlapping), 0.72 – 0.55 (m, 1H, overlapping).

^{13}**C{^{1}H} NMR** (126 MHz, $CDCl_3$) δ 140.5, 120.1, 85.1, 65.6, 49.9 (overlapping), 49.8 (overlapping), 49.4, 46.9, 43.1, 42.2, 42.1 (overlapping), 35.6, 35.6 (overlapping), 35.5 (overlapping), 34.8, 31.9, 31.4, 26.1, 24.6, 23.8, 13.8 ppm.

HRMS (ESI) calculated for $C_{42}H_{72}NO_4$ $[2M+NH_4]^+$calcd.: 654.5461, Found: 654.5400

Compound 2o:

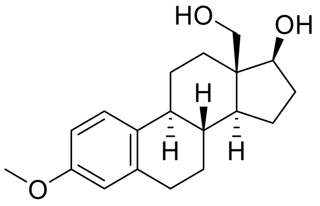

Following **GP-1**, **S2** (143 mg, 0.500 mmol, 1.00 equiv) was converted to the corresponding diethyl(hydrido)silyl ether with $[Ir(COD)OMe]_2$ (1.00 mL of a 1.00 mM stock solution in THF, 1.00 µmol, 0.20 mol%) as catalyst and Et_2SiH_2 (78 µL, 0.600 mmol, 1.20 equiv) at room temperature for 45 h. The subsequent C-H silylation (**GP-3**) was conducted with $[Ir(COD)OMe]_2$ (3.3 mg, 5.00 µmol, 1.0 mol%) and Me_4phen (3.0 mg, 12.5 µmol, 2.5 mol%) at 120 °C for 48 h to provide the intermediate oxasilolane. Subsequently, volatile materials were removed under reduced pressure and the crude product was used in the next step without further purification. The crude product was dissolved in DMF (20.0 mL) and sequentially treated with KF (291 mg, 5.00 mmol, 10.0 equiv) and *m*CPBA (1120 mg, 77%, 5.00 mmol, 10.0 equiv) and stirred at room temperature for 20 h. The reaction was quenched by carefully adding 10.0 mL of a sat. $Na_2S_2O_3/NaHCO_3$ (1:1 v/v) solution and extracted with EtOAc (3 x 15 mL), the organic layers were combined, washed with $NaHCO_3$ (20 mL), 5% HCl (20 mL), brine (20 mL), dried over $MgSO_4$ and concentrated. The crude mixture was purified by flash column chromatography (SiO_2, hexanes/EtOAc 9:1 to 3:2) to give **2o** as a colorless solid (60.6 mg, 0.200 mmol, 40%).

^{1}**H NMR** (500 MHz, $CDCl_3$) δ 7.21 (d, *J* = 8.6 Hz, 1H), 6.72 (dd, *J* = 8.6, 2.8 Hz, 1H), 6.62 (d, *J* = 2.9 Hz, 1H), 4.00 (t, *J* = 8.6 Hz, 1H), 3.90 (d, *J* = 11.5 Hz, 1H), 3.77 (s, 4H), 2.91 – 2.77 (m, 2H), 2.66 (dt, *J* = 13.0, 3.4 Hz, 1H), 2.40 – 2.31 (m, 1H), 2.30 – 2.15 (m, 2H), 1.91 – 1.68 (m, 3H), 1.58 (qd, *J* = 13.4, 4.0 Hz, 1H), 1.47 – 1.22 (m, 5H), 1.16 (td, *J* = 13.0, 4.1 Hz, 1H) ppm.

^{13}**C{^{1}H} NMR** (126 MHz, $CDCl_3$) δ 157.7, 137.9, 132.5, 126.4, 114.0, 111.6, 83.9, 60.8, 55.4, 49.8, 46.1, 44.0, 38.8, 31.4, 31.1, 29.7, 27.6, 25.8, 23.4 ppm.

HRMS (ESI) *m/z* for $C_{19}H_{26}NaO_3$ $[M+Na]^+$ calcd.: 325.1780, found: 325.1777.

Compound 2p:

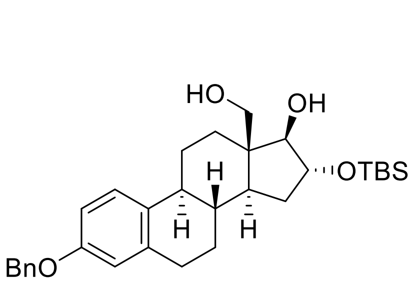

Following **GP-1**, **S3** (49.3 mg, 0.100 mmol, 1.00 equiv) was converted to the corresponding diethyl(hydrido)silyl ether with $[Ir(COD)OMe]_2$ (0.200 mL of a 1.00 mM stock solution in THF, 0.200 µmol, 0.20 mol%) as catalyst and Et_2SiH_2 (15.5 µL, 0.120 mmol, 1.20 equiv) at room temperature for 16 h. The subsequent C-H silylation was conducted with $[Ir(COD)OMe]_2$ (1.00 mL of a 1.00

mM stock solution in THF, 1.00 μmol, 1.0 mol%) and Me_4phen (0.6 mg, 2.5 μmol, 2.5 mol%) at 120 °C for 24 h to provide the intermediate oxasilolane. Subsequently, volatile materials were removed under reduced pressure and the crude product was used in the next step without further purification. The crude product was dissolved in DMF (2.00 mL) and cooled to 0°C. Subsequently, to the solution was added KHF_2 (19.5 mg, 0.250 mmol, 2.50 equiv) and *m*CPBA (67 mg, 77%, 0.300 mmol, 3.00 equiv), and the solution was allowed to warm to room temperature and stirred overnight. The reaction was quenched by carefully adding 0.6 mL of a sat. $Na_2S_2O_3/NaHCO_3$ (1:1 v/v) solution and extracted with EtOAc (3 x 10 mL), the organic layers were combined and dried over Na_2SO_4 and concentrated under reduced pressure. The crude mixture was purified by flash column chromatography (SiO_2, hexanes/EtOAc 1:0 to 3:2) to give **2p** as a colorless solid. (22.4 mg, 0.044 mmol, 44%).

^{1}H NMR (400 MHz, $CDCl_3$) δ 7.45 – 7.35 (m, 4H), 7.34 – 7.29 (m, 1H), 7.19 (d, *J* = 8.6 Hz, 1H), 6.78 (dd, *J* = 8.6, 2.8 Hz, 1H), 6.71 (d, *J* = 2.7 Hz, 1H), 5.03 (s, 2H), 4.34 (ddd, *J* = 8.8, 5.4, 2.1 Hz, 1H), 3.80 – 3.77 (m, 2H), 2.83 (dd, *J* = 8.6, 4.1 Hz, 2H), 2.66 – 2.49 (m, 1H), 2.41 – 2.19 (m, 2H), 1.96 – 1.78 (m, 2H), 1.75 – 1.66 (m, 1H), 1.64 – 1.51 (m, 4H), 1.37 – 1.24 (m, 3H), 1.03 – 0.93 (m, 1H), 0.92 (s, 9H), 0.10 (s, 3H), 0.07 (s, 3H) ppm.

^{13}C{^{1}H} NMR (101 MHz, $CDCl_3$) δ 157.0, 137.9, 137.4, 132.7, 128.7, 128.0, 127.6, 126.3, 115.0, 112.5, 92.2, 79.4, 77.5, 77.2, 76.8, 70.1, 62.1, 47.3, 46.4, 44.0, 38.4, 34.6, 31.5, 29.7, 27.6, 26.1, 25.4, 18.3, -4.4 ppm.

HRMS (ESI): m/z for $C_{31}H_{44}O_4SiNa$ $[M+Na]^+$ calcd.: 531.2901, found: 531.2894.

Compound 2q:

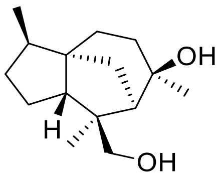

Following **GP-2**, (+)-cedrol (111 mg, 0.500 mmol, 1.00 equiv) was converted to the corresponding diethyl(hydrido)silyl ether with $RuCl_2(PPh_3)_3$ (1.0 mg, 1.00 μmol, 0.20 mol%) in benzene at 50°C for 18 h. The subsequent C-H silylation (**GP-3**) was conducted with $[Ir(COD)OMe]_2$ (3.3 mg, 5.00 μmol, 1.0 mol%) and Me_4phen (3.0 mg, 12.5 μmol, 2.5 mol%) at 120 °C for 48 h to provide the intermediate oxasilolane. Tamao-Fleming oxidation (**GP-5**) and purification by flash column chromatography (SiO_2, hexanes/EtOAc 9:1 to 1:1) gave **2q** as a colorless solid (40.5 mg, 0.170 mmol, 34%).

^{1}H NMR (500 MHz, $CDCl_3$) δ 3.57 (d, *J* = 10.8 Hz, 1H), 3.49 (d, *J* = 10.8 Hz, 1H), 1.93 – 1.77 (m, 3H), 1.77 – 1.66 (m, 4H), 1.63 (dt, *J* = 12.6, 4.0 Hz, 1H), 1.55 (ddd, *J* = 13.6, 8.3, 6.1 Hz, 1H), 1.49 – 1.34 (m, 3H), 1.33 (s, 3H), 1.32 – 1.24 (m, 3H), 1.02 (s, 3H), 0.84 (d, *J* = 7.1 Hz, 3H).

^{13}C{^{1}H} NMR (126 MHz, $CDCl_3$) δ 76.4, 68.6, 57.1, 56.5, 54.1, 43.7, 41.6, 40.7, 37.2, 31.4, 30.3, 28.9, 27.8, 25.6, 15.7 ppm.

HRMS (ESI) *m/z* for $C_{15}H_{26}NaO_2$ $[M+Na]^+$ calcd.: 261.1830, found: 261.1820.

Limitations in the scope of silylation and oxidation:

Cyclic terpenoids bearing a hydroxyl group in an equatorial position (or which underwent hydrosilylation to form a diethylhydridosilyl ether in an equatorial position) generally did not undergo C–H silylation. Examples of this trend include digoxigenin, rockogenin, and cholestenone.

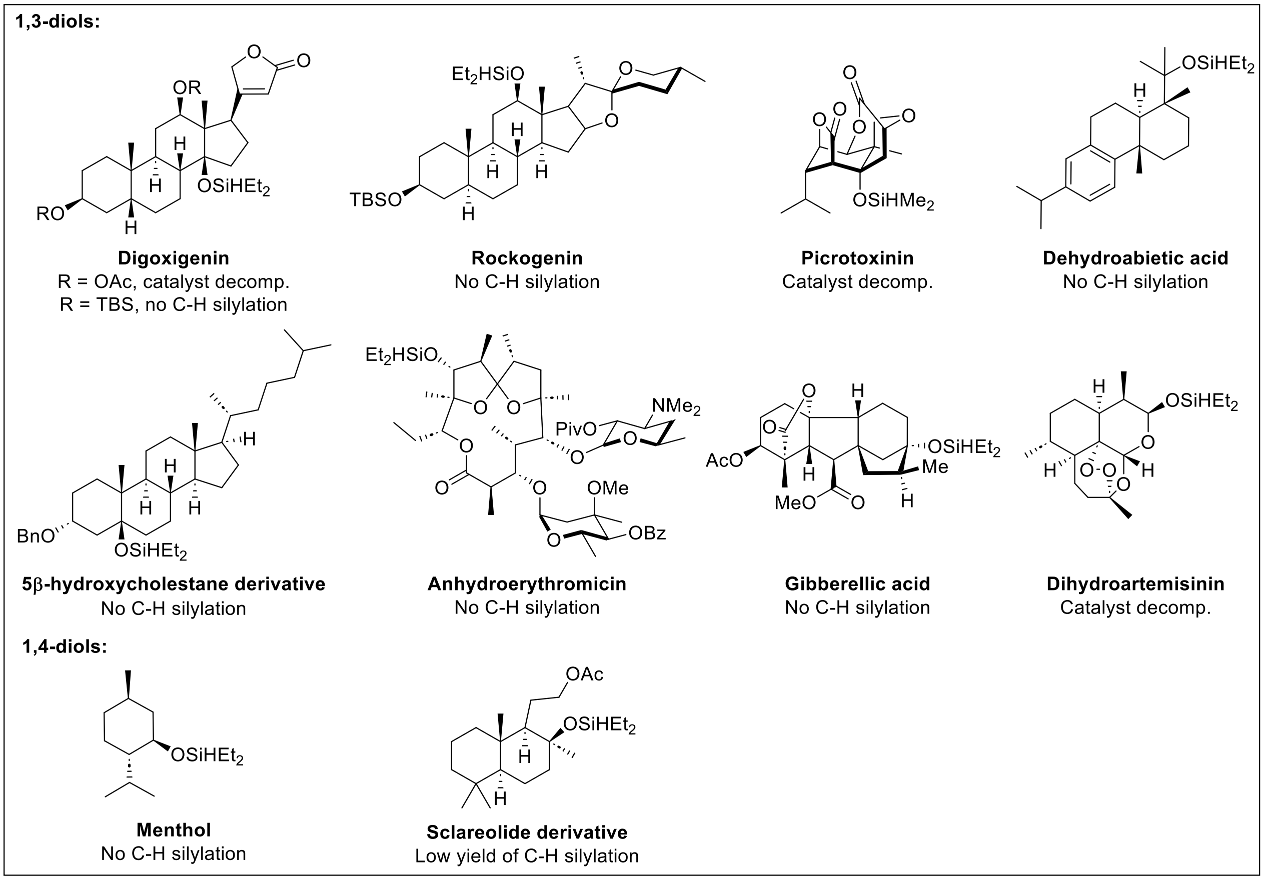

4. Substitution of Methyl Groups

4A. Synthesis of carboxylic acid intermediates

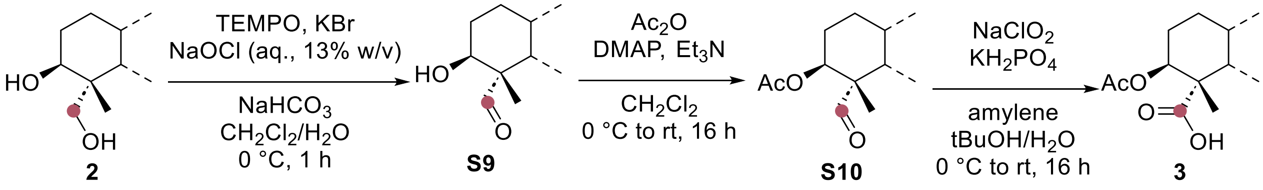

General procedure for selective oxidation of 1,3-diols (GP-6)

On the benchtop, the substrate (1.00 equiv), KBr (20 mol%), and TEMPO (20 mol%) were weighed into a round-bottomed flask. A magnetic stir bar was added, and the solids were dissolved in a mixture of sat. aq. $NaHCO_3$ and CH_2Cl_2 (1:1 v/v, 0.05 M). The resulting solution was cooled to 0 °C and treated with a fresh solution of aq. NaOCl (13% w/v, three additions of 1.15 equiv or until complete conversion to the corresponding aldol **S9**, as determined by TLC analysis). The reaction was quenched with a sat. $Na_2S_2O_3$ solution, and the resulting mixture was extracted three times with CH_2Cl_2. The combined organic layers were dried over $MgSO_4$, passed through a plug of silica gel (flushing with 50% EtOAc/hexanes), and concentrated by rotary evaporation. The product obtained was carried forward without further purification. If desired, analytically pure product can be obtained by flash column chromatography on silica gel.

General procedure for acetylation of aldols (GP-7)

On the bench top, the substrate (1.00 equiv) and DMAP (10 mol%) were weighed into a 20 mL screw-capped vial equipped with a magnetic stir bar and dissolved in CH_2Cl_2 (0.20 M). To the resulting mixture was sequentially added Et_3N (4.00 equiv) and Ac_2O (4.00 equiv). The vial was capped with a Teflon-lined screw cap, and the reaction was stirred for 16 h at room temperature or until complete consumption of starting material, as determined by TLC analysis. The crude reaction mixture was diluted with CH_2Cl_2 and washed with 1 M HCl (2 times) and sat. aq. $NaHCO_3$. The combined organic layers were dried over $MgSO_4$, passed through a plug of silica gel (flushing with 50% EtOAc/hexanes), and concentrated by rotary evaporation. The product obtained was carried forward without further purification. If desired, analytically pure product can be obtained by flash column chromatography on silica gel.

General procedure for Pinnick oxidation of aldols (GP-8)

To a 20 mL screw-capped vial equipped with stir bar, the aldol (1.00 equiv) and KH_2PO_4 (1.50 equiv) were added. ^{t}BuOH and deionized water (5:1 v/v, 0.10 M) were then added, followed by amylene (4.50 equiv). The reaction mixture was cooled to 0 °C and $NaClO_2$ (4.00 equiv) was added in one portion. ***(Caution: do not mix solid $NaClO_2$ directly with the substrate, as it is a strong oxidant)***. The vial was capped with a Teflon-lined screw cap, allowed to warm to room temperature, and stirred for 12 h or until complete conversion to the corresponding carboxylate was observed, as determined by TLC analysis. If necessary, additional $NaClO_2$, KH_2PO_4, and amylene were added. The reaction was acidified to pH 5 with acetic acid, washed with sat. $Na_2S_2O_3$ solution and extracted in EtOAc (3 times). The combined organic layers were dried over $MgSO_4$, filtered, and concentrated by rotary evaporation to obtain the carboxylic acid product **3**

(yields are usually quantitative). The acid can be used without further purification. Traces of acetic acid or ethyl acetate generally did not affect the following transformations. For characterization, the product was further purified by column chromatography on silica gel (DCM/EtOAc + 0.5% AcOH).

Investigation of conditions for selective oxidation of the primary alcohol

Several conditions were investigated for the selective oxidation of the primary alcohol in diol **3**. The results of the investigation are summarized in the table below.

Conditions	Yield of 9	Remarks
N-methylmorpholine oxide, $Cu(MeCN)_4PF_6$ (10 mol%), 4,4'-dimethoxybipyridine (10 mol%), TEMPO (5 mol%), N-methylimidazole (20 mol%), MeCN/THF , O_2 (1 atm), 65 °C, 3 h<br>[Stahl oxidation]	0%	No conversion
Dess-Martin periodinane, CH_2Cl_2, 0 °C, 1 h	29%	14% dioxidized product
TEMPO (20 mol%), $PhI(OAc)_2$ (1.2 equiv), CH_2Cl_2/MeCN, rt, 16 h	Not determined	Complex mixture
Pyridinium chlorochromate, Celite, CH_2Cl_2, rt, 16 h	0%	Overoxidation to dioxidized product
TEMPO (20 mol%), KBr (20 mol%), NaOCl, $NaHCO_3$, CH_2Cl_2/H_2O, 0 °C, 1 h	72%-74%	

Compound 3a:

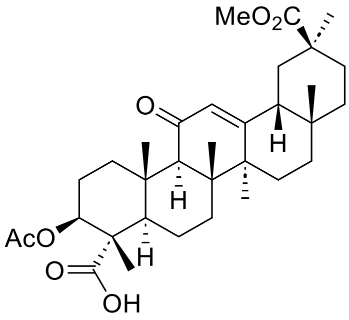

Diol **2d** was subject to **GP-6**, **GP-7**, and **GP-8** to give **3a** as a colorless solid (66% over 3 steps).

^{1}H NMR (500 MHz, $CDCl_3$) δ 5.68 (s, 1H), 5.17 (dd, *J* = 11.4, 5.2 Hz, 1H), 3.69 (s, 3H), 2.86 (dt, *J* = 13.7, 3.5 Hz, 1H), 2.43 (s, 1H), 2.11 – 2.06 (m, 1H), 2.00 (s, 3H), 1.37 (s, 3H), 1.22 (s, 3H), 1.18 (s, 3H), 1.15 (s, 3H), 1.12 (s, 3H), 1.01 (ddd, *J* = 13.7, 4.6, 2.2 Hz, 1H), 0.80 (s, 3H).

^{13}C{^{1}H} NMR (126 MHz, $CDCl_3$) δ 199.75, 181.21, 177.09, 170.49, 169.86, 128.47, 77.09 (overlaps with peak of $CDCl_3$), 61.83, 52.29, 51.95, 50.48, 48.56, 45.80, 44.20, 43.39, 41.22,

38.64, 37.87, 36.42, 32.37, 31.96, 31.26, 28.66, 28.46, 26.62, 26.52, 23.58, 23.19, 21.20, 20.49, 18.74, 17.00, 11.66.

HRMS (ESI): *m/z* for $C_{33}H_{49}O_7$ $[M+H]^+$ calcd.: 557.3473, found: 557.3476 (residual: 0.58 ppm).

Compound 3b:

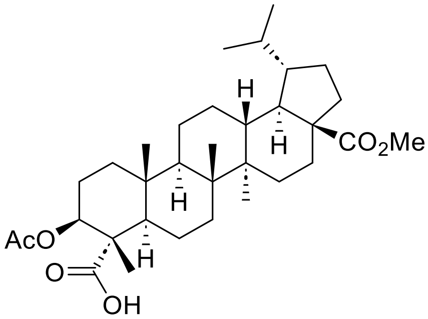

Diol **2i** was subject to **GP-6**, **GP-7**, and **GP-8** to give **3b** as a colorless solid (77% over 3 steps).

^{1}H NMR (500 MHz, $CDCl_3$) δ 5.14 (dd, *J* = 11.9, 4.6 Hz, 1H), 3.63 (s, 3H), 2.26 – 2.13 (m, 3H), 1.98 (s, 3H), 1.82 – 1.55 (m, 8H), 1.54 – 1.26 (m, 11H), 1.25 – 1.17 (m, 2H), 1.16 (s, 3H), 1.12 – 1.05 (m, 3H), 0.94 (s, 3H), 0.89 (s, 3H), 0.87 (s, 3H), 0.85 (d, *J* = 6.8 Hz, 3H), 0.75 (d, *J* = 6.7 Hz, 3H).

^{13}C{^{1}H} NMR (126 MHz, $CDCl_3$) δ 182.12, 177.01, 170.47, 77.50, 57.12, 52.16, 51.31, 51.14, 50.42, 49.04, 44.32, 42.73, 41.18, 38.27, 38.19, 37.42, 36.64, 34.01, 32.15, 29.89, 29.81, 26.92, 23.27, 23.12, 22.91, 21.21, 21.19, 21.12, 16.61, 16.02, 14.83, 14.78, 11.51.

HRMS (ESI): *m/z* for $C_{35}H_{55}NO_6Na$ $[M+ACN+Na]^+$ calcd.: 608.3922, found: 608.3897 (residual: 4.05 ppm).

Compound 3c:

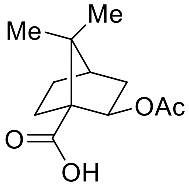

Diol **2b** was subject to **GP-6**, **GP-7**, and **GP-8** to give **3c** as a colorless solid (46% over 3 steps).

^{1}H NMR (500 MHz, $CDCl_3$) δ 4.99 (dd, *J* = 7.6, 4.0 Hz, 1H), 2.15 – 2.04 (m, 1H), 2.00 (s, 3H), 1.94 – 1.70 (m, 4H), 1.56 (ddd, *J* = 13.5, 9.4, 4.3 Hz, 1H), 1.24 (s, 3H), 1.20 – 1.12 (m, 1H), 1.09 (s, 3H).

^{13}C{^{1}H} NMR (126 MHz, $CDCl_3$) δ 178.96, 170.51, 79.58, 57.82, 48.36, 45.88, 38.94, 31.04, 26.59, 21.30, 20.98, 20.51.

HRMS (ESI): *m/z* for $C_{14}H_{21}NO_4Na$ $[M+ACN+Na]^+$ calcd.: 290.1363, found: 290.1361 (residual: 0.57 ppm).

Compound 3d:

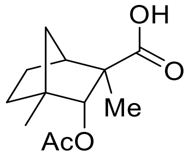

Diol **2a** was subject to **GP-6**, **GP-7**, and **GP-8** to give **3d** as a colorless solid (44% over 3 steps).

^{1}**H NMR** (500 MHz, $CDCl_3$) δ 4.48 (d, *J* = 2.1 Hz, 1H), 2.18 (dd, *J* = 3.9, 1.9 Hz, 1H), 2.06 (s, 3H), 1.85 (dddd, *J* = 11.8, 8.9, 5.2, 2.3 Hz, 1H), 1.74 (tdd, *J* = 9.0, 4.4, 2.6 Hz, 1H), 1.65 (tdd, *J* = 13.1, 5.3, 4.3 Hz, 1H), 1.57 (dq, *J* = 10.7, 2.1 Hz, 1H), 1.48 (s, 3H), 1.28 (dd, *J* = 10.7, 1.7 Hz, 1H), 1.14 (tdd, *J* = 12.0, 4.2, 1.9 Hz, 1H), 1.09 (s, 3H).

^{13}**C{^{1}H} NMR** (126 MHz, $CDCl_3$) δ 179.10, 172.10, 83.87, 51.23, 47.82, 46.80, 40.32, 27.15, 27.02, 26.85, 21.31, 18.83.

HRMS (ESI): *m/z* for $C_{14}H_{21}NO_4Na$ $[M+ACN+Na]^+$ calcd.: 290.1363, found: 290.1375 (residual: 4.26 ppm).

Compound 3e:

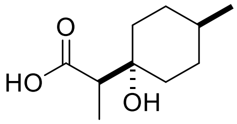

(±)

Diol **2e** (126.0 mg, 0.732 mmol, 1.00 equiv) was subjected to **GP-6** but not purified by column chromatography. We found that the aldehyde decomposes in contact with silica gel (probably by dehydration to the corresponding enal). As a result, the crude product obtained after extraction, consisting largely of the aldol and TEMPO, was directly subject to **GP-8** to give acetoxyacid **3e** as a colorless solid (89.3 mg, 66% over 2 steps). This substrate was not acetylated as the alcohol was tertiary.

^{1}**H NMR** (500 MHz, $CDCl_3$) δ 2.86 (q, *J* = 7.2 Hz, 1H), 1.95 – 1.76 (m, 2H), 1.75 – 1.59 (m, 2H), 1.59 – 1.48 (m, 2H), 1.41 (ddd, *J* = 13.4, 11.8, 3.8 Hz, 1H), 1.25 (d, *J* = 7.2 Hz, 3H), 1.13 (dddd, *J* = 13.8, 11.8, 10.0, 3.5 Hz, 1H), 0.99 (dddd, *J* = 13.6, 11.8, 10.0, 3.5 Hz, 1H), 0.91 (d, *J* = 6.7 Hz, 3H).

^{13}**C{^{1}H} NMR** (126 MHz, $CDCl_3$) δ 181.66, 72.23, 42.94, 37.25, 33.04, 31.17, 31.06, 30.46, 21.03, 11.59.

HRMS (ESI): *m/z* for $C_{12}H_{21}NO_3Na$ $[M+ACN+Na]^+$ calcd.: 250.1414, found: 250.1416 (residual: 0.94 ppm).

4B. Decarboxylative Giese reactions

Adapted from a procedure reported by MacMillan.[4]

On the bench top, to an oven-dried 1-dram screw-capped vial equipped with stir bar was added the carboxylic acid (1.00 equiv), Ir[dF(CF_3)ppy]$_2$(dtbbpy)PF_6 (1.0 mol%), the olefin (if solid)(2.00 equiv) and K_2HPO_4 (1.20 equiv). N,N-dimethylformamide (0.10 M) and the olefin (if liquid)(2.00 equiv) were then added. The vial was then capped with a Teflon-lined septum cap and sparged with N_2 for 1 minute. The reaction mixture was then irradiated by blue LEDs for 16 h at 30 °C, with cooling from an overhead fan. The reaction mixture was diluted with CH_2Cl_2 and washed with water (3 times). The combined organic layers were dried over $MgSO_4$, concentrated by rotary evaporation, and purified by column chromatography (ethyl acetate/hexanes) on silica gel to give the desired product.

Compound 4aa:

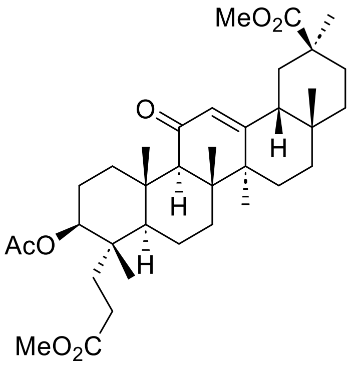

Following the procedure given above, acid **3a** (20.0 mg, 0.036 mmol, 1.00 equiv) was allowed to react with methyl acrylate (4.86 µL, 0.054 mmol, 1.50 equiv) to give product **4aa** as a colorless solid (10.9 mg, 51%).

^{1}H NMR (400 MHz, $CDCl_3$) δ 5.66 (s, 1H), 4.66 (dd, J = 11.6, 4.8 Hz, 1H), 3.68 (s, 3H), 3.66 (s, 3H), 2.80 (dt, J = 13.7, 3.6 Hz, 1H), 2.42 – 2.35 (m, 1H), 2.34 (s, 1H), 2.03 (s, 3H), 1.34 (s, 3H), 1.17 (s, 3H), 1.15 (s, 3H), 1.11 (s, 3H), 1.04 – 0.98 (m, 2H), 0.92 (s, 3H), 0.79 (m, 4H, methyl peak overlapping with a 1H peak).

^{13}C{^{1}H} NMR (126 MHz, $CDCl_3$) δ 199.94, 177.07, 174.37 (-CH_2CH_2**C**O_2Me), 170.92, 169.50, 128.60, 75.28, 62.06, 51.93, 51.83, 49.73, 48.53, 45.53, 44.19, 43.28, 41.22, 40.11, 38.91, 37.87, 37.00, 32.55, 32.32, 31.96, 31.26, 28.65, 28.46, 28.23, 26.61, 26.52, 23.58, 23.53, 21.38, 18.73, 18.02, 17.19, 17.05.

HRMS (ESI): *m/z* for $C_{36}H_{54}O_7Na$ $[M+Na]^+$ calcd.: 621.3762, found: 621.3741 (residual: 3.41 ppm).

Compound 4ab:

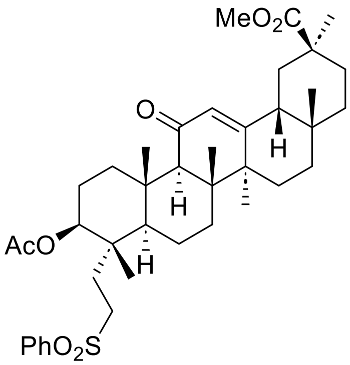

Following the procedure given above, acid **3a** (22.1 mg, 0.0397 mmol, 1.00 equiv) was allowed to react with phenyl vinyl sulfone (10.1 mg, 0.0596 mmol, 1.50 equiv) to give product **4ab** as a colorless solid (20.2 mg, 75%).

^{1}H NMR (500 MHz, $CDCl_3$) δ 7.93 – 7.82 (m, 2H), 7.66 (t, *J* = 7.4 Hz, 1H), 7.58 (t, *J* = 7.6 Hz, 2H), 5.66 (s, 1H), 4.49 (dd, *J* = 11.7, 4.7 Hz, 1H), 3.68 (s, 3H), 3.24 (td, *J* = 13.5, 3.8 Hz, 1H), 3.08 – 2.76 (m, 2H), 2.30 (s, 1H), 2.12 – 1.96 (m, 3H), 1.91 (dt, *J* = 13.6, 3.5 Hz, 1H), 1.80 (ddd, *J* = 25.1, 13.6, 4.2 Hz, 2H), 1.72 (s, 3H), 1.70 – 1.64 (m, 2H), 1.64 – 1.48 (m, 3H), 1.47 – 1.35 (m, 3H), 1.33 (s, 3H), 1.31 – 1.21 (m, 3H), 1.20 – 1.15 (m, 1H), 1.15 (s, 3H), 1.14 (s, 3H), 1.10 (s, 3H), 1.06 – 0.98 (m, 1H), 0.93 (td, *J* = 13.7, 3.7 Hz, 1H), 0.87 (s, 3H), 0.79 (s, 3H), 0.75 – 0.63 (m, 1H).

^{13}C{^{1}H} NMR (126 MHz, $CDCl_3$) δ 199.66, 177.04, 170.41, 169.69, 138.56, 133.71, 129.33, 128.51, 128.44, 74.80, 61.93, 51.91, 51.09, 50.01, 48.50, 45.46, 44.17, 43.27, 41.21, 40.53, 38.87, 37.85, 37.05, 32.55, 31.94, 31.22, 29.94, 28.64, 28.43, 26.59, 26.48, 23.55, 23.49, 20.86, 18.62, 18.02, 17.29, 16.92.

HRMS (ESI): *m/z* for $C_{40}H_{56}O_7SNa$ $[M+Na]^+$ calcd.: 703.3639, found: 703.3673 (residual: 4.81 ppm).

Compound 4ba:

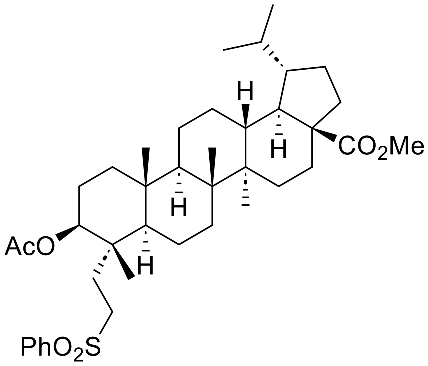

Following the procedure given above, acid **3b** (15.0 mg, 0.0275 mmol, 1.00 equiv) was allowed to react with phenyl vinyl sulfone (7.0 mg, 0.0413 mmol, 1.50 equiv) to give product **4ba** as a colorless solid (13.9 mg, 75%).

^{1}H NMR (500 MHz, $CDCl_3$) δ 7.96 – 7.85 (m, 2H), 7.69 – 7.61 (m, 1H), 7.57 (dd, *J* = 8.4, 7.0 Hz, 2H), 4.47 (dd, *J* = 11.3, 4.9 Hz, 1H), 3.64 (s, 3H), 3.20 (td, *J* = 13.5, 3.8 Hz, 1H), 2.87 (td, *J* = 13.5, 4.2 Hz, 1H), 2.28 – 2.10 (m, 3H), 1.83 – 1.76 (m, 2H), 1.75 (s, 3H), 1.73 – 1.60 (m, 3H), 1.56 – 1.39 (m, 4H), 1.37 – 1.08 (m, 14H), 0.92 (s, 3H), 0.88 (s, 3H), 0.86 – 0.82 (m, 8H), 0.75 (d, *J* = 6.7 Hz, 3H), 0.68 – 0.64 (m, 1H).

^{13}C{^{1}H} NMR (126 MHz, $CDCl_3$) δ 176.97, 170.50, 138.76, 133.65, 129.32, 128.44, 75.06, 57.12, 51.32, 51.08, 50.67, 50.38, 49.04, 44.32, 42.65, 40.85, 40.31, 38.47, 38.15, 37.40, 37.22, 34.24, 32.14, 29.90, 29.82, 29.75, 26.92, 23.62, 23.12, 22.90, 21.13, 20.93, 17.88, 17.75, 16.90, 15.98, 14.84.

HRMS (ESI): *m/z* for $C_{40}H_{60}O_6SNa$ $[M+Na]^+$ calcd.: 691.4003, found: 691.4036 (residual: 4.77 ppm).

4C. Decarboxylative Minisci reactions

Adapted from a procedure reported by Sherwood.[5]

On the bench top, the carboxylic acid (1.00 equiv), N-hydroxyphthalimide (1.00 equiv), and DMAP (10.0 mol%) were weighed sequentially into a 1-dram vial equipped with a magnetic stir bar and dissolved in CH_2Cl_2 (0.20 M). To the reaction mixture, N,N'-diisopropylcarbodiimide (1.00 equiv) was added. The vial was capped with a Teflon-lined screw cap and stirred for 4 h or until complete conversion to the corresponding coupled product was observed, as determined by TLC analysis. The solution was filtered through Celite and concentrated by rotary evaporation to give the corresponding redox active ester, which was typically used without further purification.

The redox active ester was transferred into a flame-dried one-dram screw-capped vial equipped with stir bar and combined with 4-CzIPN (2.0 mol%) and an aromatic heterocycle (2.00 equiv) before being dissolved in anhydrous DMSO (0.20 M). The mixture was treated with trifluoracetic acid (2.00 equiv) before being capped with a Teflon-lined septum cap and sparged with N_2 for 2 minutes. The vial was then capped with a Teflon-lined septum cap and sparged with N_2 for 1 minute. The reaction mixture was then irradiated by blue LEDs for 24 h at 45 °C (in a box with the overhead fan turned off). The reaction was diluted with Et_2O, quenched with sat. aq. $NaHCO_3$ and washed with water (3 times). The combined organic layers were dried over $MgSO_4$ and concentrated by rotary evaporation. The crude mixture was purified by column chromatography (ethyl acetate/hexanes) on silica gel to give the desired product.

Compound 4ac:

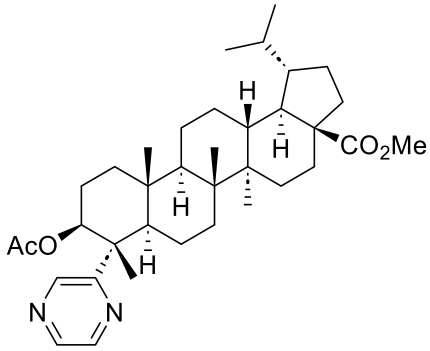

Following the procedure given above, acid **3a** (16.6 mg, 0.0298 mmol, 1.00 equiv) was converted to product **4bb** as a colorless solid (5.0 mg, 29%).

^{1}H NMR (400 MHz, $CDCl_3$) δ 8.61 (d, *J* = 1.5 Hz, 1H), 8.60 – 8.55 (m, 1H), 8.39 (d, *J* = 2.4 Hz, 1H), 5.69 (s, 1H), 5.44 (dd, *J* = 10.6, 6.1 Hz, 1H), 3.69 (s, 4H), 2.94 (d, *J* = 13.7 Hz, 1H), 2.50 (s, 1H), 1.75 (s, 4H), 1.51 (s, 3H), 1.34 (s, 4H), 1.29 (s, 3H), 1.15 (s, 4H), 1.10 (s, 3H), 0.78 (s, 3H).

^{13}C{^{1}H} NMR (126 MHz, $CDCl_3$) δ 200.01, 177.08, 169.69, 161.79, 143.88, 143.79, 142.20, 128.57, 78.83, 62.05, 52.73, 51.94, 48.55, 48.53, 45.82, 44.20, 43.35, 41.22, 38.73, 37.87, 37.06, 32.46, 31.95, 31.27, 29.85, 28.64, 28.47, 26.57, 26.50, 23.65, 23.55, 21.07, 18.94, 18.76, 17.41, 13.08.

HRMS (ESI): *m/z* for $C_{36}H_{51}N_2O_5$ $[M+H]^+$ calcd.: 591.3793, found: 591.3788 (residual: 0.80 ppm).

Compound 4bb:

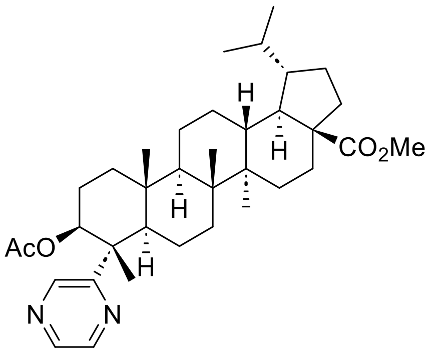

Following the procedure given above, acid **3b** (14.6 mg, 0.0268 mmol, 1.00 equiv) was converted to product **4bb** as a colorless solid (3.6 mg, 23%).

^{1}H NMR (500 MHz, $CDCl_3$) δ 8.59 (d, *J* = 2.2 Hz, 2H), 8.37 (d, *J* = 2.2 Hz, 1H), 5.45 (dd, *J* = 11.8, 4.5 Hz, 1H), 3.63 (s, 3H), 2.28 – 2.13 (m, 3H), 1.74 (s, 3H), 1.46 (s, 3H), 0.99 (s, 4H), 0.92 (s, 3H), 0.88 (s, 3H), 0.85 (d, *J* = 6.9 Hz, 4H), 0.75 (d, *J* = 6.7 Hz, 4H), 0.57 – 0.47 (m, 1H).

^{13}C{^{1}H} NMR (126 MHz, $CDCl_3$) δ 176.99, 170.07, 161.90, 143.90, 143.55, 141.73, 79.11, 57.12, 53.55, 51.30, 50.55, 49.06, 48.41, 44.35, 42.71, 41.19, 38.31, 38.22, 37.42, 37.28, 34.13, 32.14, 29.90, 29.77, 26.99, 23.62, 23.14, 22.91, 21.18, 21.10, 19.74, 16.92, 16.05, 14.86, 14.79, 12.99.

HRMS (ESI): *m/z* for $C_{36}H_{58}N_3O_4$ $[M+NH_4]^+$ calcd.: 596.4422, found: 596.4413 (residual: 4.05 ppm).

Compound 4ca:

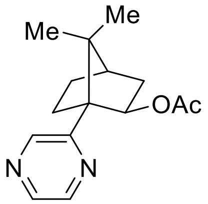

Following the procedure given above, acid **3c** (13.6 mg, 0.0600 mmol, 1.00 equiv) was converted to product **4ca** as a colorless solid (11.2 mg, 76%).

^{1}H NMR (600 MHz, $CDCl_3$) δ 8.64 – 8.59 (m, 1H), 8.55 – 8.48 (m, 1H), 8.37 (d, *J* = 2.5 Hz, 1H), 5.09 (dd, *J* = 8.0, 3.8 Hz, 1H), 2.29 (td, *J* = 11.1, 6.4 Hz, 1H), 2.09 – 1.96 (m, 2H), 1.91 – 1.86 (m, 2H), 1.84 (s, 3H), 1.79 (ddd, *J* = 13.6, 9.9, 4.3 Hz, 1H), 1.44 (s, 3H), 1.31 – 1.26 (m, 1H), 1.04 (s, 3H).

^{13}C{^{1}H} NMR (126 MHz, $CDCl_3$) δ 170.18, 156.91, 144.94, 143.70, 141.92, 80.96, 57.03, 49.19, 47.30, 39.56, 32.88, 26.62, 21.81, 21.27, 21.13.

HRMS (ESI): *m/z* for $C_{15}H_{21}N_2O_2$ $[M+H]^+$ calcd.: 261.1598, found: 261.1605 (residual: 2.77 ppm).

Compound 4cb:

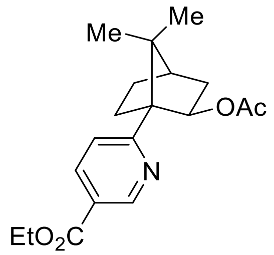

Major

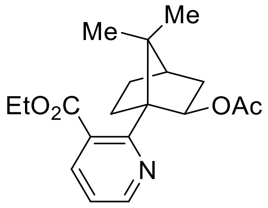

Minor

Following the procedure given above, acid **3a** (20.8 mg, 0.0600 mmol, 1.00 equiv) was converted to product **4cb** as an inseparable mixture of two isomers (**major:minor** = 9:1). Colorless solid (4.6 mg, 25%).

Major diastereomer:

^{1}H NMR (500 MHz, $CDCl_3$) δ 9.15 (dd, *J* = 2.3, 0.9 Hz, 1H), 8.16 (dd, *J* = 8.3, 2.3 Hz, 1H), 7.33 (dd, *J* = 8.2, 0.9 Hz, 1H), 5.16 (dd, *J* = 7.8, 3.9 Hz, 1H), 4.39 (q, *J* = 7.1 Hz, 2H), 2.26 – 2.14 (m, 1H), 2.05 – 1.92 (m, 3H), 1.90 – 1.80 (m, 3H), 1.78 (s, 3H), 1.43 (s, 3H), 1.39 (t, *J* = 7.1 Hz, 3H), 1.09 (s, 3H).

^{13}C{^{1}H} NMR (126 MHz, $CDCl_3$) δ 170.26, 165.88, 149.93, 136.26, 123.62, 122.58, 81.20, 61.28, 58.47, 48.90, 47.46, 39.38, 33.72, 26.72, 21.74, 21.30, 21.27, 14.44.

HRMS (ESI): *m/z* for $C_{19}H_{26}NO_4$ $[M+H]^+$ calcd.: 332.1857, found: 332.1868 (residual: 3.38 ppm).

Minor diastereomer (only distinct peaks included):

^{1}H NMR (500 MHz, $CDCl_3$) δ 8.62 (dd, *J* = 4.7, 1.9 Hz, 1H), 7.67 (dd, *J* = 7.7, 1.9 Hz, 1H), 7.13 (dd, *J* = 7.7, 4.7 Hz, 1H), 5.16 (1H, overlapping), 4.39 (2H, overlapping).

Compound 4cc:

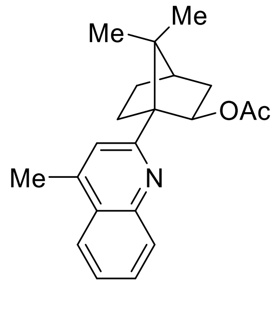

Following the procedure given above, acid **3a** (26 mg, 0.0700 mmol, 1.00 equiv) was converted to product **4cc** as a colorless solid (9.8 mg, 44%).

^{1}H NMR (500 MHz, $CDCl_3$) δ 8.04 (dd, *J* = 8.4, 1.2 Hz, 1H), 7.93 (dd, *J* = 8.3, 1.5 Hz, 1H), 7.64 (ddd, *J* = 8.4, 6.8, 1.4 Hz, 1H), 7.49 (ddd, *J* = 8.2, 6.8, 1.3 Hz, 1H), 7.19 – 7.14 (m, 1H), 5.27 (dd, *J* = 7.8, 3.9 Hz, 1H), 2.65 (s, 3H), 2.27 – 2.19 (m, 1H), 2.09 – 1.95 (m, 2H), 1.92 – 1.75 (m, 3H), 1.72 (s, 3H), 1.54 (s, 3H), 1.30 – 1.25 (m, 1H), 1.24 (s, 3H).

^{13}C{^{1}H} NMR (126 MHz, $CDCl_3$) δ 170.55, 161.27, 147.58, 142.63, 130.32, 128.57, 126.73, 125.57, 123.58, 121.86, 81.08, 58.18, 49.06, 47.22, 39.43, 33.88, 27.01, 21.68, 21.64, 21.30, 18.92.

HRMS (ESI): *m/z* for $C_{21}H_{26}NO_2$ $[M+H]^+$ calcd.: 324.1958, found: 324.1958 (residual: 0.07 ppm).

Compound 4cd:

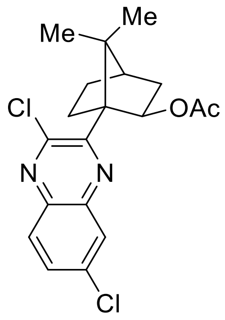

Following the procedure given above, acid **3a** (20.8 mg, 0.0600 mmol, 1.00 equiv) was converted to product **4cd** as a colorless solid (7.0 mg, 33%).

^{1}H NMR (500 MHz, $CDCl_3$) δ 8.09 (d, *J* = 2.3 Hz, 1H), 7.91 (d, *J* = 8.9 Hz, 1H), 7.68 (dd, *J* = 8.9, 2.3 Hz, 1H), 5.75 (dd, *J* = 8.1, 3.7 Hz, 1H), 2.43 – 2.36 (m, 1H), 2.17 (dd, *J* = 13.4, 8.1 Hz, 1H), 1.97 – 1.87 (m, 3H), 1.82 (d, *J* = 4.3 Hz, 1H), 1.76 (s, 3H), 1.45 (s, 3H), 1.40 (s, 3H), 1.38 – 1.30 (m, 1H).

^{13}C{^{1}H} NMR (126 MHz, $CDCl_3$) δ 170.11, 156.17, 147.26, 140.15, 138.40, 135.72, 131.27, 129.09, 127.88, 79.58, 58.08, 50.73, 46.49, 39.49, 30.62, 26.77, 21.92, 21.05, 20.97.

HRMS (ESI): *m/z* for $C_{19}H_{21}Cl_2N_2O_2$ $[M+H]^+$ calcd.: 379.0975, found: 379.0982 (residual: 1.91 ppm).

4D. Curtius rearrangements

Adapted from a procedure reported by Dragoli.[6]

On the bench top, the carboxylic acid (1.00 equiv) was weighed into an oven-dried 1-dram screw-capped vial equipped with a magnetic stir bar and dissolved in anisole (0.125 M). To the resulting mixture was added Et_3N (1.60 equiv) and diphenylphosphoryl azide (1.50 equiv). The vial was capped with a Teflon-lined screw cap and the reaction was stirred for 16 h at 90 °C. The crude reaction mixture was diluted with ethyl acetate and sequentially washed with 5% w/v citric acid and sat. aq. $NaHCO_3$. The organic layer was dried over $MgSO_4$, filtered and concentrated by rotary evaporation. The crude product was purified by column chromatography on silica gel (ethyl acetate/hexanes) to give the desired product.

Compound 4ad:

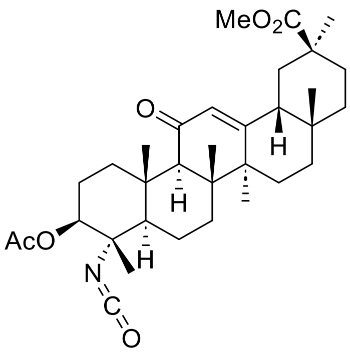

Following the procedure given above, acid **3a** (27.8 mg, 0.0500 mmol, 1.00 equiv) was converted to product **4ad** as a colorless solid (14.9 mg, 54%).

^{1}H NMR (700 MHz, $CDCl_3$) δ 5.68 (s, 1H), 4.77 (dd, *J* = 12.2, 4.7 Hz, 1H), 3.69 (s, 3H), 2.81 (dt, *J* = 13.9, 3.6 Hz, 1H), 2.39 (s, 1H), 2.11 (s, 3H), 2.08 – 1.97 (m, 2H), 1.92 (dt, *J* = 13.7, 3.6 Hz, 1H), 1.87 – 1.68 (m, 4H), 1.59 – 1.38 (m, 3H), 1.37 (s, 3H), 1.34 (s, 3H), 1.32 – 1.18 (m, 4H), 1.15 (s, 3H), 1.12 (two overlapping methyl singlets, 6H), 1.05 – 0.98 (m, 1H), 0.80 (s, 3H).

^{13}C{^{1}H} NMR δ 199.50, 177.03, 170.57, 169.95, 128.42, 125.45 (-N=**C**=O), 80.16, 65.18, 61.54, 55.12, 51.92, 48.56, 45.69, 44.17, 43.42, 41.21, 38.31, 37.84, 37.21, 32.21, 31.96, 31.24, 28.66, 28.44, 26.59, 26.47, 24.31, 23.49, 21.24, 19.11, 18.73, 18.16, 16.44.

HRMS (ESI): *m/z* for $C_{35}H_{50}N_2O_6Na$ $[M+ACN+Na]^+$ calcd.: 617.3561, found: 617.3534 (residual: 4.32 ppm).

Compound 4bc:

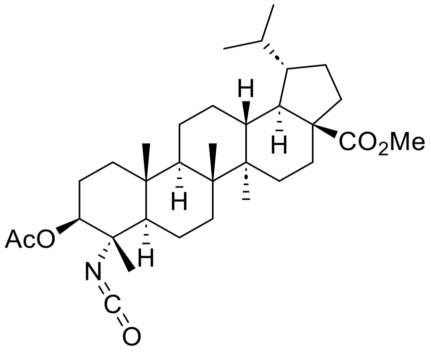

Following the procedure given above, acid **3a** (27.8 mg, 0.0500 mmol, 1.00 equiv) was converted to product **4ad** as a colorless solid (14.9 mg, 54%).

^{1}H NMR (500 MHz, $CDCl_3$) δ 4.73 (dd, *J* = 12.1, 4.6 Hz, 1H), 3.64 (s, 3H), 2.28 – 2.16 (m, 3H), 2.10 (s, 3H), 1.80 (ddt, *J* = 11.7, 7.0, 3.9 Hz, 3H), 1.68 (ddt, *J* = 16.6, 10.3, 3.2 Hz, 3H), 1.29 (s, 3H), 0.94 (s, 3H), 0.91 (s, 3H), 0.85 (d, *J* = 6.9 Hz, 3H), 0.83 (s, 3H), 0.75 (d, *J* = 6.8 Hz, 3H).

^{13}C{^{1}H} NMR (126 MHz, $CDCl_3$) δ 176.98, 170.61, 125.23 (-N=**C**=O), 80.36, 65.24, 57.12, 55.61, 51.33, 50.29, 49.03, 44.32, 42.77, 41.07, 38.18, 37.86, 37.64, 37.42, 33.88, 32.14, 29.90, 29.85, 29.79, 26.90, 24.32, 23.12, 22.91, 21.27, 21.20, 18.97, 18.91, 16.16, 16.06, 14.83, 14.71.

HRMS (ESI): *m/z* for $C_{35}H_{54}N_2O_5Na$ $[M+ACN+Na]^+$ calcd.: 605.3925, found: 605.3948 (residual: 3.82 ppm).

Compound 4ah:

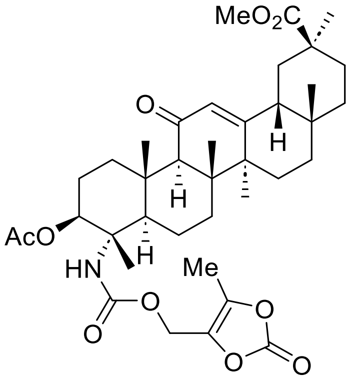

Adapted from a procedure reported by Dragoli.[6]

On the bench top, isocyanate **4ad** (14.9 mg, 0.0269 mmol, 1.00 equiv) and 4-(hydroxymethyl)-5-methyl-1,3-dioxol-2-one (4.2 mg, 0.0323 mmol, 1.20 equiv) were weighed into an oven-dried 1-dram screw-capped vial equipped with a magnetic stir bar and dissolved in dichloromethane (790 μL, 0.03 M). To the resulting mixture was added trimethylsilyl chloride (17.0 μL, 0.135 mmol, 5.00 equiv). The vial was capped with a Teflon-lined screw cap and the reaction was stirred for 16 h at room temperature. The crude reaction mixture was concentrated by rotary evaporation and

purified by column chromatography on silica gel (ethyl acetate/hexanes, 0% to 50%) to give **4ah** as a colorless solid (14.2 mg, 77%).

^{1}H NMR (500 MHz, $CDCl_3$) δ 5.67 (s, 1H), 5.44 (dd, *J* = 11.8, 5.2 Hz, 1H), 4.89 – 4.64 (m, 2H), 4.40 (s, 1H), 3.68 (s, 3H), 2.79 (dt, *J* = 13.7, 3.6 Hz, 1H), 2.46 (s, 1H), 2.18 (s, 3H), 2.11 – 2.05 (m, 1H), 2.02 (s, 3H), 1.14 (s, 3H), 1.14 (s, 3H), 1.11 (s, 6H), 1.05 – 0.97 (m, 1H), 0.79 (s, 3H).

^{13}C{^{1}H} NMR δ ^{13}C NMR (126 MHz, $CDCl_3$) δ 199.88, 177.08, 170.45, 169.93, 152.90, 152.51, 140.05, 134.25, 128.46, 74.34, 61.83, 60.32, 53.73, 51.92, 48.84, 48.56, 45.75, 44.19, 43.34, 41.19, 38.17, 37.86, 37.50, 32.16, 31.95, 31.25, 28.65, 28.45, 26.60, 26.50, 24.63, 23.64, 21.25, 18.66, 17.37, 17.29, 16.77, 9.53.

HRMS (ESI): *m/z* for $C_{40}H_{56}N_2O_{10}Na$ $[M+ACN+Na]^+$ calcd.: 747.3827, found: 747.3811 (residual: 2.16 ppm).

4E. Decarboxylative fluorination

Adapted from a procedure reported by Hu.[7]

On the bench top, to an oven-dried one-dram screw-capped vial equipped with stir bar was added the carboxylic acid (1.00 equiv). The vial was transferred to a N_2-filled glovebox and sequentially treated with $Fe(OAc)_2$ (10 mol%), 4,4' dimethoxy-2,2' bipyridyl (20 mol%), and 1-fluoro-4-methyl-1,4-diazabicyclo[2.2.2]octane-1,4-diium tetrafluoroborate (2.10 equiv). The vial was removed from the glovebox, and acetonitrile and deionized water (v/v = 1:1, 0.05 M) were added. The reaction mixture was treated with 2,6-lutidine (1.80 equiv), sealed with a Teflon-lined septum cap, and sparged with N_2 for 2 minutes. The reaction mixture was irradiated with blue LEDs at 30 °C for 16 h, with cooling from an overhead fan. The reaction mixture was washed with water and extracted with CH_2Cl_2. The combined organic layers were dried over $MgSO_4$, concentrated by rotary evaporation, and purified by column chromatography (ethyl acetate/hexanes) on silica gel to give the desired product.

Compound 4ae:

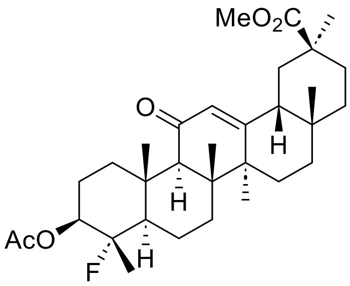

Following the procedure given above, acid **3a** (13.9 mg, 0.0250 mmol, 1.00 equiv) was converted to product **4ad** as a colorless solid (7.0 mg, 54%).

^{1}H NMR (500 MHz, $CDCl_3$) δ 5.69 (s, 1H), 4.90 (ddd, *J* = 15.4, 12.3, 5.2 Hz, 1H, C**H**–OAc), 3.69 (s, 2H), 2.77 (dt, *J* = 14.6, 2.9 Hz, 1H), 2.44 (s, 1H), 2.08 (s, 3H), 1.38 (s, 3H), 1.31 (d, *J* = 24.6 Hz, 3H, C(F)–**CH_3**), 1.15 (s, 2H), 1.13 (s, 3H), 1.11 (s, 3H), 1.03 (ddt, *J* = 13.7, 4.5, 2.2 Hz, 1H), 0.81 (s, 3H).

The peak at 4.90 ppm has $^3J_{H\text{-}F}$ = 15.4 Hz, which is inconsistent with coupling between an axial proton and an axial fluorine (typically >25 Hz). Therefore, the configuration of C-4, the carbon bearing the fluorine atom, is assigned as (S), with the fluorine atom being equatorial.

^{19}F NMR (470 MHz, $CDCl_3$) δ -133.08 (qt, *J* = 25.6, 13.3 Hz).

The fluorine atom couples to a methyl group with *J* = 25.6 Hz and to two axial protons with *J* = 13.3 Hz.

^{13}C{^{1}H} NMR (126 MHz, $CDCl_3$) δ 199.62, 177.07, 170.52, 170.03, 128.41, 97.50 (d, *J* = 171.6 Hz, **C**–F), 77.97 (d, *J* = 20.7 Hz), 61.23, 53.97 (d, *J* = 18.7 Hz, **C**H–OAc), 51.94, 48.62, 45.52, 44.20, 43.46, 41.25, 38.36, 37.88, 37.40 (d, *J* = 8.6 Hz), 31.98, 31.73, 28.69, 28.46, 26.62, 26.51, 25.25 (d, *J* = 8.6 Hz), 23.47, 21.40, 18.76, 16.61 (d, *J* = 3.0 Hz), 16.12 (d, *J* = 27.5 Hz, C(F)–**C**H_3), 15.63.

HRMS (ESI): *m/z* for $C_{34}H_{50}FNO_5Na$ [M+ACN+Na]$^+$ calcd.: 594.3566, found: 594.3593 (residual: 4.60 ppm).

Compound 4bd:

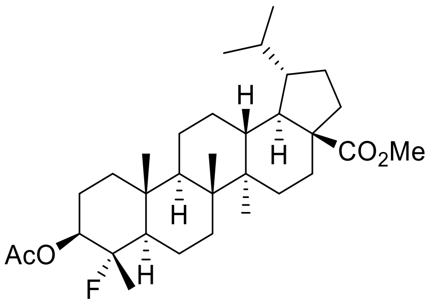

Following the procedure given above, acid **3b** (12.3 mg, 0.0226 mmol, 1.00 equiv) was converted to product **4bd** as a colorless solid (5.4 mg, 47%).

^{1}H NMR (500 MHz, $CDCl_3$) δ 4.87 (ddd, *J* = 15.3, 12.2, 5.2 Hz, 1H), 3.65 (s, 3H), 2.27 – 2.16 (m, 3H), 2.08 (s, 3H), 1.85 – 1.76 (m, 3H), 1.71 – 1.64 (m, 3H), 1.34 (d, *J* = 39.2 Hz, 3H), 1.25 (s, 3H), 0.95 (s, 3H), 0.92 (s, 3H), 0.85 (d, *J* = 6.9 Hz, 3H), 0.81 (s, 3H), 0.75 (d, *J* = 6.8 Hz, 3H).

^{19}F NMR (470 MHz, $CDCl_3$) δ -133.97 (qt, *J* = 25.4, 12.9 Hz).

^{13}C{^{1}H} NMR (126 MHz, $CDCl_3$) δ 177.01, 170.57, 97.69 (d, *J* = 171.5 Hz, **C**–F), 78.22 (d, *J* = 21.0 Hz, **C**H–OAc), 57.13, 54.29 (d, *J* = 18.4 Hz), 51.33, 49.97, 49.04, 44.33, 42.78, 40.88, 38.21, 37.96 (d, *J* = 8.1 Hz), 37.83, 37.43, 33.32, 32.16, 29.91, 29.78, 26.94, 25.20 (d, *J* = 8.5 Hz), 23.13, 22.92, 21.42, 21.35, 17.40, 16.07, 15.92 (d, *J* = 27.6 Hz, C(F)–**C**H_3), 15.44, 14.83, 14.68.

HRMS (ESI): *m/z* for $C_{32}H_{54}FNO_4Na$ [M+ACN+Na]$^+$ calcd.: 565.4086, found: 565.4109 (residual: 4.98 ppm).

Compound 4da:

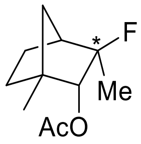

dr = 15:1

Following the procedure given above, acid **3d** (18.1 mg, 0.0800 mmol, 1.00 equiv) was converted to product **4da** as a colorless oil (7.5 mg, 47%).

^{1}H NMR (500 MHz, $CDCl_3$) δ 4.84 (dt, *J* = 30.8, 1.6 Hz, 1H), 2.31 – 2.20 (m, 1H), 2.11 (s, 3H), 1.79 (dq, *J* = 10.5, 1.9 Hz, 1H), 1.62 (dddd, *J* = 15.5, 11.2, 4.9, 2.0 Hz, 2H), 1.42 – 1.33 (m, 1H), 1.29 – 1.26 (m, 1H), 1.23 (d, *J* = 23.2 Hz, 3H), 1.15 – 1.11 (m, 1H), 1.10 (s, 3H).

In a norbornane derivative $^3J_{HF,cis}$ = 24.7 Hz (dihedral angle ~0°), $^3J_{HF,trans}$ = 12.2 Hz (dihedral angle ~120°).[8] Therefore, given the large coupling constant ($^3J_{HF}$ = 30.8 Hz) observed for the major diastereomer, we have assigned the configuration of the carbon bearing the fluorine atom as (R).

^{19}F NMR (471 MHz, $CDCl_3$) δ -125.92 (p, *J* = 25.9 Hz).

^{13}C{^{1}H} NMR (126 MHz, $CDCl_3$) δ 170.74, 102.80 (d, *J* = 178.4 Hz, **C**–F), 84.46 (d, *J* = 28.9 Hz, **C**–OAc), 47.57 (d, *J* = 23.6 Hz), 47.45, 40.32, 26.62, 24.99 (d, *J* = 8.9 Hz), 20.96, 18.68, 17.56 (d, *J* = 26.3 Hz).

HRMS (ESI): *m/z* for $C_{11}H_{17}FO_2$ $[M+ACN+Na]^+$ calcd.: 264.1370, found: 264.1381 (residual: 4.07 ppm).

4F. Decarboxylative deuteration

Adapted from a procedure reported by Zhu.[9]

On the bench top, to an oven-dried one-dram screw-capped vial equipped with stir bar was added the carboxylic acid (1.00 equiv) and $CsOH \cdot H_2O$ (0.90 equiv). The solids were dissolved in MeOH (0.25 M), and the reaction mixture was capped with a Teflon-lined septum cap and stirred for 1 h at room temperature. The crude cesium carboxylate was concentrated at 60 °C for 3 h (the stir bar was temporarily removed during this operation to prevent bumping).

The stir bar was replaced, and the colorless solid was treated with $Ir[dF(CF_3)ppy]_2(dtbbpy)PF_6$ (1.0 mol%). The reaction mixture was dissolved in CH_2Cl_2 and D_2O (4:1 v/v, 0.15 M). The vial was charged with 2,4,6-triisopropylbenzenethiol (10 mol%), capped with a Teflon-lined septum cap, and sparged with N_2 for 2 minutes. The reaction mixture was irradiated by blue LEDs for 16 h at 30 °C, with cooling from an overhead fan. The reaction mixture was diluted with CH_2Cl_2, dried over $MgSO_4$, concentrated by rotary evaporation, and purified by column chromatography (ethyl acetate/hexanes) on silica gel to give the desired product.

Compound 4af:

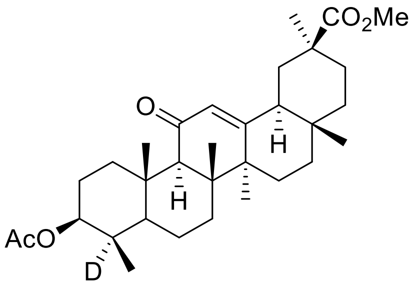

Following the procedure given above, acid **3a** (20.0 mg, 0.0360 mmol, 1.00 equiv) was converted to product **4af** as a colorless solid (14.9 mg, 81%).

^{1}H NMR (500 MHz, $CDCl_3$) δ 5.66 (s, 1H), 4.79 (dd, J = 12.3, 4.6 Hz, 1H), 3.68 (s, 3H), 2.79 (dt, J = 13.6, 3.6 Hz, 1H), 2.33 (s, 1H), 2.03 (s, 3H), 1.36 (s, 4H), 1.14 (s, 3H), 1.13 (s, 5H), 1.12 (s, 3H), 1.01 (tdd, J = 12.9, 6.6, 4.0 Hz, 2H), 0.87 (s, 3H), 0.80 (s, 3H).

The deuterium incorporation was measured by accurate integration of the ^{1}H NMR spectra of the deuterated and protonated compounds from 2.17 – 2.10 ppm (the alkene peak, 5.685 – 5.645 ppm was set to an integral of 1.00 for both spectra). The integration of this region was 0.20 for deuterated compound **4af**, while the integration of the same region was 1.19 for the protonated compound **4ag**. Based on these data, the deuterium incorporation was >95%.

^{2}H NMR (92 MHz, $CHCl_3$) δ 2.12 (s, 1H).

^{13}C NMR (126 MHz, $CDCl_3$) δ 200.19, 177.08, 170.85, 169.62, 128.57, 76.37, 61.36, 51.92, 48.57, 48.26, 45.95, 44.18, 43.39, 41.25, 39.38, 37.88, 37.31 (t, $^1J_{C\text{-}D}$ = 18.8 Hz), 36.49, 32.21, 31.95, 31.26, 28.66, 28.46, 26.61, 26.53, 23.51, 23.47, 22.45, 21.45, 18.76, 16.91, 9.59.

A small peak corresponding to *protio* compound was observed at 37.81 ppm.

HRMS (ESI): *m/z* for $C_{32}H_{48}DO_5$ $[M+H]^+$ calcd.: 514.3638, found: 514.3651 (residual: 2.57 ppm).

Compound 4be:

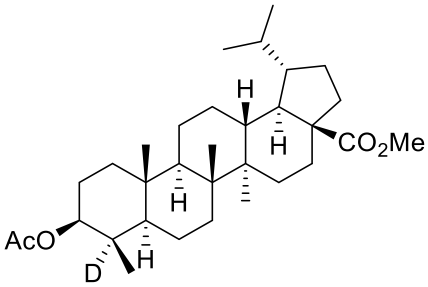

Following the procedure given above, acid **3b** (40.6 mg, 0.060 mmol, 1.00 equiv) was converted to product **4be** as a colorless solid (25.1 mg, 83%).

^{1}H NMR ^{1}H NMR (500 MHz, $CDCl_3$) δ 4.77 (dd, *J* = 12.0, 4.7 Hz, 1H), 3.64 (s, 3H), 2.34 – 2.12 (m, 3H), 2.02 (s, 3H), 1.83 – 1.73 (m, 2H), 1.73 – 1.61 (m, 4H), 1.57 – 1.41 (m, 5H), 1.36 – 1.27 (m, 6H), 1.26 – 1.04 (m, 6H), 0.93 (s, 3H), 0.90 (s, 3H), 0.84 (d, *J* = 6.9 Hz, 3H), 0.82 (m, 6H, two overlapping singlets), 0.74 (d, *J* = 6.7 Hz, 3H).

The deuterium incorporation was measured by accurate integration of the ^{1}H NMR spectra of the deuterated and protonated compounds from 2.09 – 1.99 ppm (the C**H**–OAc peak, 4.83 – 4.73 ppm was set to an integral of 1.00 for both spectra). The integration of this region was 3.21 for deuterated compound **4af**, while the integration of the same region was 4.16 for the protonated compound **4ag**. Based on this, the deuterium incorporation was 95%.

^{2}H NMR (92 MHz, $CHCl_3$) δ 2.03.

^{13}C NMR (126 MHz, $CDCl_3$) δ 177.00, 170.79, 76.64, 57.12, 51.28, 49.80, 49.07, 48.63, 44.33, 42.73, 41.39, 38.93, 38.28, 37.44, 37.20 (t, $^1J_{C\text{-}D}$ = 19.5 Hz), 36.63, 33.90, 32.20, 29.88, 29.79, 26.98, 24.39, 23.12, 22.91, 22.66, 21.46, 21.00, 16.58, 16.02, 14.81, 14.73, 9.47.

A small peak corresponding to *protio* compound was observed at 37.71 ppm.

HRMS (ESI): *m/z* for $C_{34}H_{54}DNO_4Na$ $[M+ACN+Na]^+$ calcd.: 565.4086, found: 565.4109 (residual: 4.13 ppm).

4G. Decarboxylative protonation

Adapted from a procedure reported by Zhu.[9]

On the bench top, to an oven-dried one-dram screw-capped vial equipped with stir bar was added the carboxylic acid (1.00 equiv) and $CsOH \cdot H_2O$ (0.90 equiv). The solids were dissolved in MeOH (0.25 M), and the reaction mixture was capped with a Teflon-lined septum cap and stirred for 1 h at room temperature. The crude cesium carboxylate was concentrated at 60 °C for 3 h (the stir bar was temporarily removed during this operation to prevent bumping).

The stir bar was replaced, and the colorless solid was treated with $Ir[dF(CF_3)ppy]_2(dtbbpy)PF_6$ (1.0 mol%). The reaction mixture was dissolved in CH_2Cl_2 and H_2O (4:1 v/v, 0.15 M). The vial was charged with 2,4,6-triisopropylbenzenethiol (10 mol%), capped with a Teflon-lined septum cap, and sparged with N_2 for 2 minutes. The reaction mixture was irradiated by blue LEDs for 16 h at 30 °C, with cooling from an overhead fan. The reaction mixture was diluted with CH_2Cl_2, dried over $MgSO_4$, concentrated by rotary evaporation, and purified by column chromatography (ethyl acetate/hexanes) on silica gel to give the desired product.

Compound 4ag:

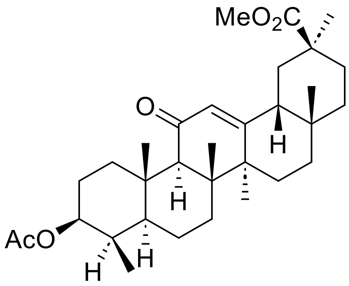

Following the procedure given above, acid **3a** (20.0 mg, 0.0360 mmol, 1.00 equiv) was converted to product **4ag** as a colorless solid (15.9 mg, 86%).

^{1}H NMR (500 MHz, $CDCl_3$) δ 5.66 (s, 1H), 4.80 (dt, *J* = 12.3, 5.0 Hz, 1H, C**H**–OAc), 3.68 (s, 3H), 2.79 (dt, *J* = 13.6, 3.6 Hz, 1H), 2.33 (s, 1H), 2.03 (s, 3H), 1.36 (s, 3H), 1.14 (s, 3H), 1.13 (s, 3H), 1.12 (s, 3H), 1.05 – 0.96 (m, 2H), 0.88 (d, *J* = 7.6 Hz, 3H), 0.80 (s, 3H).

The peak at 4.80 ppm (proton on C-3) has one coupling with $^3J_{HH}$ = 12.3 Hz and two couplings with $^3J_{H\text{-}H}$ ≈ 5.0 Hz. This proton is axial, so the larger coupling constant (12.3 Hz) must be from $^3J_{HH}$(axial-axial) with the axial proton on C-2. One of the smaller couplings (5.0 Hz) must be from $^3J_{HH}$(axial-equatorial) with the equatorial proton on C-2. This leaves a coupling constant of ~5.0 Hz for the coupling with the newly installed proton on C-4. The magnitude of this coupling constant is only consistent with an equatorial configuration of that proton. Therefore, we assign the configuration of C-4 as (R).

^{13}C NMR (126 MHz, $CDCl_3$) δ 200.19, 177.08, 170.84, 169.62, 128.57, 76.43, 61.37, 51.92, 48.58, 48.36, 45.95, 44.18, 43.39, 41.26, 39.39, 37.88, 37.81, 36.52, 32.21, 31.95, 31.27, 28.66, 28.46, 26.61, 26.53, 23.51, 22.46, 21.45, 18.76, 16.92, 9.71.

HRMS (ESI): *m/z* for $C_{64}H_{97}O_{10}$ $[2M+H]^+$ calcd.: 1025.7077, found: 1025.7084 (residual: 0.71 ppm).

Compound 4bf:

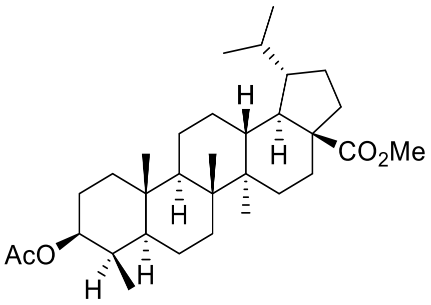

Following the procedure given above, acid **3b** (20.0 mg, 0.0367 mmol, 1.00 equiv) was converted to product **4bf** as a colorless solid (14.8 mg, 80%).

^{1}H NMR ^{1}H NMR (500 MHz, $CDCl_3$) δ 4.78 (dt, *J* = 12.2, 4.9 Hz, 1H), 3.64 (s, 3H), 2.27 – 2.14 (m, 3H), 2.07 – 2.03 (m, 1H), 2.03 (s, 3H), 1.84 – 1.74 (m, 2H), 1.73 – 1.61 (m, 4H), 1.57 – 1.40 (m, 5H), 1.35 – 1.28 (m, 6H), 1.26 – 1.05 (m, 6H), 0.94 (s, 3H), 0.90 (s, 3H), 0.85 (d, *J* = 6.9 Hz, 3H), 0.84 (d, *J* = 7.5 Hz, 3H), 0.83 (s, 3H), 0.74 (d, *J* = 6.8 Hz, 3H).

^{13}C NMR (126 MHz, $CDCl_3$) δ 177.02, 170.81, 76.71, 57.13, 51.30, 49.81, 49.08, 48.74, 44.34, 42.74, 41.41, 38.94, 38.29, 37.72, 37.45, 36.67, 33.92, 32.21, 29.89, 29.80, 26.99, 24.44, 23.13, 22.92, 22.68, 21.48, 21.01, 16.60, 16.03, 14.82, 14.74, 9.61.

HRMS (ESI): *m/z* for $C_{34}H_{55}NO_4Na$ $[M+ACN+Na]^+$ calcd.: 564.4023, found: 564.4001 (residual: 3.94 ppm).

4H. Retro-Claisen condensation sequence

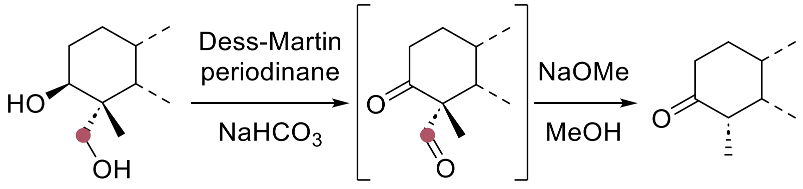

To a solution of the 1,3-diol (1.00 equiv) in dichloromethane (0.10 M) was added $NaHCO_3$ (10.0 equiv) and Dess-Martin periodinane (2.40 equiv). The resulting suspension was stirred at room temperature for 4 hours. After completion, a sat. aq. solution of $NaHCO_3$ and $Na_2S_2O_3$ (1:1 v/v) was added to quench the reaction, and the resulting solution was stirred for 15 min. The organic layer was separated and washed with brine, dried over $MgSO_4$ and concentrated under reduced pressure. The crude product was transferred into a glovebox, where NaOMe (1.50 equiv) and MeOH or THF (0.10 M) were added. The resulting solution was sealed and stirred at room temperature overnight. After completion, the reaction was quenched by adding water and the product was extracted with EtOAc (3 x 5 mL). The combined organic layers were washed with brine, dried over Na_2SO_4, concentrated under reduced pressure, and purified by column chromatography on silica to give the desired product.

Compound 6a:

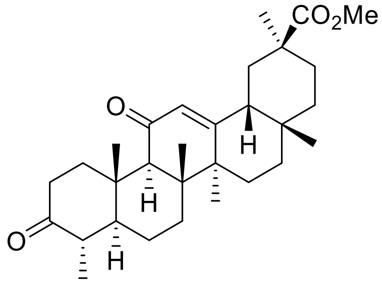

Compound **6a** was synthesized following the general procedure from **2d** (18.9 mg, 0.0403 mmol, 1.00 equiv). The crude product was purified by flash column chromatography (SiO_2, hexanes/EtOAc 85:15 to 4:1) to afford **6a** as a colorless solid (18.0 mg, 0.0384 mmol, 95% over 2 steps).

The ^1H NMR spectra of this compound were identical to those reported by Pinhey.[10]

^{1}H NMR (700 MHz, $CDCl_3$) δ 5.71 (s, 1H), 3.68 (s, 3H), 3.04 (ddd, *J* = 13.4, 6.8, 2.4 Hz, 1H), 2.55 (dddd, *J* = 14.9, 13.6, 6.8, 1.1 Hz, 1H), 2.43 (s, 1H), 2.33 (dtd, *J* = 13.1, 6.6, 1.0 Hz, 1H), 2.29 (dq, *J* = 15.1, 2.4 Hz, 1H), 2.10 (ddd, *J* = 13.5, 4.5, 1.8 Hz, 1H), 2.06 – 1.97 (m, 2H), 1.91 (ddd, *J* = 13.6, 4.4, 2.7 Hz, 1H), 1.83 (td, *J* = 13.8, 4.8 Hz, 1H), 1.69 – 1.56 (m, 4H), 1.44 – 1.38 (m, 3H), 1.35 (d, *J* = 1.0 Hz, 3H), 1.33 (s, 3H), 1.32 – 1.27 (m, 3H), 1.20 (dq, *J* = 13.9, 2.4 Hz, 1H), 1.17 (s, 3H), 1.14 (s, 3H), 1.10 (td, *J* = 12.2, 2.6 Hz, 1H), 1.00 (d, *J* = 6.6 Hz, 3H), 0.81 (s, 3H) ppm.

$^{13}C\{^1H\}$ NMR (176 MHz, $CDCl_3$) δ 213.3, 199.8, 177.0, 169.9, 128.5, 59.5, 54.0, 51.9, 48.6, 45.2, 44.6, 44.2, 43.5, 41.3, 40.5, 37.9, 37.6, 36.4, 32.0, 31.7, 31.2, 28.7, 28.4, 26.6, 26.5, 23.4, 21.2, 18.8, 13.6, 11.8 ppm.

Compound 6b:

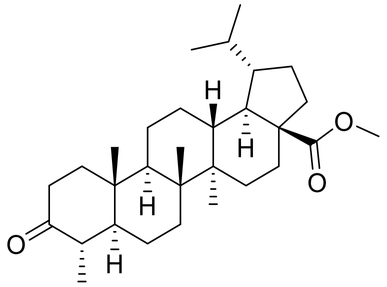

Compound **6b** was synthesized following the general procedure from **2i** (40.0 mg, 0.082 mmol). The crude product was purified by flash column chromatography (SiO_2, hexanes/EtOAc 95:5 to 3:2) to afford **6b** as a colorless solid (15.0 mg, 0.033 mmol, 40% over 2 steps).

This compound has been reported by Valterová and coworkers.[11] The authors did not report ^1H or ^{13}C NMR spectra for the compound.

^1H NMR (500 MHz, $CDCl_3$) δ 3.65 (s, 3H), 2.43 (dddd, J = 14.5, 13.2, 7.0, 1.0 Hz, 1H), 2.34 – 2.19 (m, 5H), 2.07 – 1.96 (m, 1H), 1.83 – 1.77 (m, 2H), 1.72 – 1.65 (m, 1H), 1.02 (s, 3H), 0.97 (d, J = 6.6 Hz, 3H), 0.96 (s, 3H), 0.93 (s, 3H), 0.85 (d, J = 6.9 Hz, 3H), 0.74 (d, J = 6.7 Hz, 3H).

$^{13}C\{^1H\}$ NMR (126 MHz, $CDCl_3$) δ 214.01, 176.99, 57.11, 53.45, 51.33, 49.03, 48.10, 44.72, 44.33, 42.78, 40.50, 40.49, 38.22, 37.60, 37.43, 36.91, 33.28, 32.19, 29.89, 29.73, 27.00, 23.12, 22.91, 22.33, 21.72, 16.08, 14.82, 14.58, 13.65, 11.74.

HRMS (ESI) *m/z* for $C_{32}H_{51}NO_3Na$ $[M+ACN+Na]^+$ calcd.: 520.3761, found: 520.3774 (residual: 2.57 ppm).

Compound 6c:

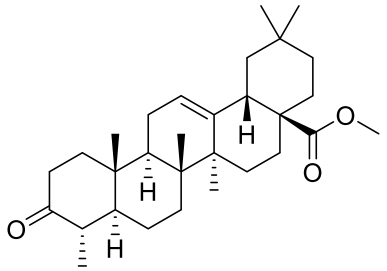

Compound **6c** was synthesized following the above procedure from **2c** (48.7 mg, 0.100 mmol, 1.00 equiv). The crude product was purified by flash column chromatography (SiO_2, hexanes/EtOAc 95:5 to 3:2) to afford **6c** as a colorless solid (34.9 mg, 0.0768 mmol, 77% over 2 steps).

The spectra of compound **6c** match those reported by Willing,[12] thereby confirming the assigned configuration at C-4. The configuration at C-4 for the other triterpenoid-derived products **6a** and **6b** were assigned by analogy to **6c**.

^{1}H NMR (500 MHz, $CDCl_3$) δ 5.36 – 5.25 (m, 1H), 3.63 (s, 3H), 2.88 (dd, *J* = 13.9, 4.6 Hz, 1H), 2.45 (td, *J* = 14.3, 6.8 Hz, 1H), 2.34 – 2.25 (m, 2H), 2.07 – 1.90 (m, 4H), 1.74 – 1.56 (m, 5H), 1.52 (dt, *J* = 13.8, 3.5 Hz, 1H), 1.47 – 1.39 (m, 1H), 1.39 – 1.24 (m, 5H), 1.22 – 1.15 (m, 2H), 1.15 – 1.04 (m, 8H), 0.99 (d, *J* = 6.5 Hz, 3H), 0.93 (s, 3H), 0.89 (s, 3H), 0.80 (s, 3H).

^{13}C{^{1}H} NMR (126 MHz, $CDCl_3$) δ 213.8, 178.4, 144.2, 122.3, 53.8, 51.7, 46.9, 45.9, 45.4, 44.9, 41.9, 41.5, 40.3, 39.2, 37.6, 36.8, 33.9, 33.2, 32.5, 31.7, 30.8, 27.8, 25.9, 24.1, 23.8, 23.2, 22.2, 17.1, 13.3, 11.8.

HRMS (ESI) *m/z* for $C_{30}H_{47}O_3$ $[M+H]^+$ calcd.: 455.3530, found: 455.3525.

Compound 6d:

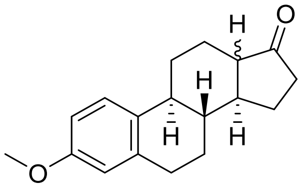

Compound **6d** was synthesized following the general procedure using **2o** (15.5 mg, 0.0500 mmol, 1.00 equiv). The crude product was purified by flash column chromatography (SiO_2, hexanes/EtOAc 1:0 to 3:2) to afford **6d** as a colorless solid (6.5 mg, 0.0240 mmol, 48% over 2 steps, 2:1 dr).

Major:

^{1}H NMR (500 MHz, $CDCl_3$) δ 7.18 (d, *J* = 8.6 Hz, 1H), 6.77 – 6.66 (m, 1H, overlapping), 6.60 (d, *J* = 2.8 Hz, 1H), 3.77 (s, 3H, overlapping), 2.85 – 2.78 (m, 2H), 2.38 (dt, *J* = 13.8, 3.9 Hz, 2H, overlapping), 2.33 – 2.21 (m, 2H, overlapping), 2.21 – 2.05 (m, 2H, overlapping), 1.98 – 1.87 (m, 1H), 1.78 – 1.63 (m, 1H, overlapping), 1.53 – 1.31 (m, 2H, overlapping), 1.30 – 1.19 (m, 2H, overlapping), 1.07 – 0.82 (m, 2H, overlapping) ppm.

^{13}C NMR (126 MHz, $CDCl_3$) δ 219.6, 157.6, 138.2, 132.0, 127.1, 113.7, 111.9, 55.3, 50.3, 42.6, 41.5, 40.4, 34.6, 30.3, 28.2, 27.9, 23.2, 23.0 ppm.

Minor:

^{1}H NMR (500 MHz, $CDCl_3$) δ 7.22 (d, *J* = 8.6 Hz, 1H), 6.77 – 6.66 (m, 1H, overlapping), 6.64 (d, *J* = 2.8 Hz, 1H), 3.78 (s, 3H, overlapping), 2.91 – 2.85 (m, 2H), 2.64 – 2.51 (m, 1H), 2.38 (dt, *J* = 13.8, 3.9 Hz, 2H, overlapping), 2.33 – 2.21 (m, 2H, overlapping), 2.21 – 2.05 (m, 2H, overlapping), 1.78 – 1.63 (m, 1H, overlapping), 1.53 – 1.31 (m, 2H, overlapping), 1.30 – 1.19 (m, 2H, overlapping), 1.07 – 0.82 (m, 2H, overlapping) ppm.

^{13}C NMR (126 MHz, $CDCl_3$) δ 218.2, 157.7, 138.1, 131.9, 126.9, 114.0, 111.8, 55.6, 48.0, 46.6, 43.1, 37.7, 30.6, 30.0, 29.8, 26.7, 25.5, 24.7 ppm.

HRMS (ESI) *m/z* for $C_{18}H_{23}O_2$ $[M+H]^+$ calcd.: 271.1698, found: 271.1700.

4I. Ring-opening of bicyclic terpenoids

The retro-Claisen condensation of fenchol-derived diol **2a** and camphor-derived diol **2b** led to ring-opening products rather than formal demethylation.

To a solution of the 1,3-diol (1.00 equiv) in dichloromethane (0.10 M) was added $NaHCO_3$ (10.0 equiv) and Dess-Martin periodinane (2.40 equiv). The resulting suspension was stirred at room temperature for 4 hours. After completion, a sat. aq. solution of $NaHCO_3$ and $Na_2S_2O_3$ (1:1 v/v) was added to quench the reaction, and the resulting solution was stirred for 15 min. The organic layer was separated and washed with brine, dried over $MgSO_4$ and concentrated under reduced pressure. The crude product was transferred into a glovebox, where NaOMe (1.50 equiv) and MeOH or THF (0.10 M) were added. The resulting solution was sealed and stirred at room temperature overnight. After completion, the reaction was quenched by adding water and the product was extracted with EtOAc (3 x 5 mL). The combined organic layers were washed with brine, dried over Na_2SO_4, concentrated under reduced pressure, and purified by column chromatography on silica to give the desired product.

Compound 6e:

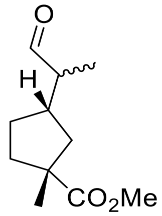

dr = 1:1

Compound **6e** was synthesized following the general procedure from **2a** (102 mg, 0.600 mmol, 1.00 equiv). The crude product was purified by flash column chromatography (SiO_2, hexanes/EtOAc 9:1 to 85:15) to afford **6e** as a colorless oil (48 mg, 0.24 mmol, 40% over 2 steps, 1:1 dr).

^{1}H NMR (500 MHz, $CDCl_3$) δ 9.64 (m, 1H, overlapping), 3.67 (m, 3H, overlapping), 2.29 (pdd, J = 6.7, 3.3, 2.2 Hz, 1H, overlapping), 2.24 – 2.15 (m, 2H, overlapping), 2.00 – 1.69 (m, 3H, overlapping), 1.55 – 1.36 (m, 2H, overlapping), 1.26 (s, 3H, overlapping), 1.08 (m, 3H, overlapping).

^{13}C{^{1}H} NMR (126 MHz, $CDCl_3$) δ 204.9, 204.9, 178.9 (overlapping), 52.1, 52.0, 51.5, 51.4, 49.2, 48.5, 42.0, 41.5, 39.9, 39.8, 37.7, 37.3, 30.3, 29.8, 25.6, 25.4, 12.4, 12.2.

HRMS (ESI) *m/z* for $C_{11}H_{22}NO_3$ $[M+NH_4]^+$ calcd.: 216.1600, found: 216.1597.

Compound 6f

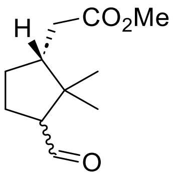

dr = 3:1

Compound **6f** was synthesized following the general procedure from **2b** (49.8 mg, 0.300 mmol, 1.00 equiv). The crude product was purified by flash column chromatography (SiO_2, hexanes/EtOAc 9:1 to 85:15) to afford **6f** as a colorless oil (48.1 mg, 0.243 mmol, 81% over 2 steps, 3:1 dr).

Major:

^{1}H NMR (500 MHz, $CDCl_3$) δ 9.74 (d, *J* = 2.4 Hz, 1H), 3.67 (s, 3H, overlapping), 2.47 – 2.34 (m, 2H, overlapping), 2.13 – 1.93 (m, 4H, overlapping), 1.88 – 1.69 (m, 1H, overlapping), 1.49 – 1.35 (m, 1H, overlapping), 1.19 (s, 3H), 0.74 (s, 3H).

^{13}C{^{1}H} NMR (126 MHz, $CDCl_3$) δ 204.7, 173.8 (overlapping), 61.9, 51.8 (overlapping), 47.7, 44.8, 34.1, 28.7, 26.6, 21.3, 16.6.

Minor:

^{1}H NMR (500 MHz, $CDCl_3$) δ 9.71 (d, *J* = 3.5 Hz, 1H), 3.67 (s, 3H, overlapping), 2.47 – 2.34 (m, 2H, overlapping), 2.13 – 1.93 (m, 4H, overlapping), 1.88 – 1.69 (m, 1H, overlapping), 1.49 – 1.35 (m, 1H, overlapping), 1.06 (s, 3H), 0.97 (s, 3H).

^{13}C{^{1}H} NMR (126 MHz, $CDCl_3$) δ 204.8, 173.8 (overlapping), 61.4, 51.8 (overlapping), 46.2, 44.9, 35.1, 29.9, 24.6, 24.1, 22.4.

HRMS (ESI) *m/z* for $C_{11}H_{22}NO_3$ $[M+NH_4]^+$ calcd.: 216.1600, found: 216.1596.

5. Elimination of Methyl Groups

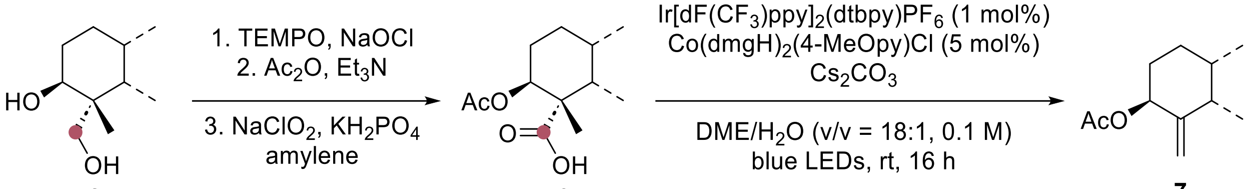

Synthesis of β-acetoxyacid 3

See Section 4a. **GP-6**, **GP-7**, and **GP-8** were followed to synthesize β-acetoxyacid **3**.

General procedure for the Decarboxyolefination of β-acetoxyacid 3

Adapted from a procedure reported by Ritter.[13]

On the benchtop, to an oven-dried one-dram screw-capped vial equipped with a magnetic stir bar was added the carboxylic acid (1.00 equiv), Ir(dF(CF$_3$)ppy]$_2$(dtbpy)PF$_6$ (1.0 mol%), Co(dmgH)$_2$(4-OMe-py)Cl (5.0 mol%), Cs_2CO_3 (1.00 equiv). The solids were dissolved in anhydrous DME (0.10 M) and H_2O (30.0 equiv). The reaction mixture was capped with a Teflon-lined septum cap and sparged with N_2 for 2 minutes. The resulting sealed reaction mixture was irradiated by blue LEDs for 16 h at 30 °C, with cooling from an overhead fan. The reaction mixture was diluted in CH_2Cl_2, dried over $MgSO_4$, passed through a Celite plug, and concentrated by rotary evaporation. The crude mixture was purified by column chromatography (ethyl acetate/hexanes) on silica gel to give the desired product.

Compound 7a:

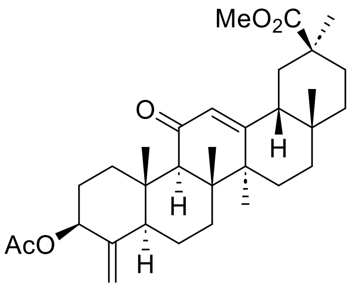

Compound **7a** was synthesized following the general procedure from **3a** (13.0 mg, 0.0234 mmol, 1.00 equiv). The crude product was purified by flash column chromatography on silica gel (hexanes/EtOAc, 0% to 30%) to afford **7a** as a colorless solid (8.3 mg, 70%).

^{1}H NMR (400 MHz, $CDCl_3$) δ 5.70 (s, 1H), 5.12 (dd, *J* = 11.8, 5.7 Hz, 1H), 4.87 (dt, *J* = 2.1, 1.1 Hz, 1H), 4.66 – 4.52 (m, 1H), 3.69 (s, 3H), 2.76 (ddd, *J* = 13.6, 4.4, 2.9 Hz, 1H), 2.52 (s, 1H), 2.12 (s, 4H), 1.39 (s, 3H), 1.15 (s, 5H), 1.14 (s, 4H), 0.98 (s, 3H), 0.81 (s, 3H).

$^{13}C\{^1H\}$ NMR (126 MHz, $CDCl_3$) δ 199.96, 177.07, 170.39, 169.77, 147.35 (R_2**C**$=CH_2$), 128.55, 103.82 ($R_2C=$**C**H_2), 74.63, 59.48, 51.94, 50.68, 48.66, 45.52, 44.19, 43.56, 41.28, 38.90, 38.15, 37.89, 32.00, 31.28, 31.23, 29.09, 28.70, 28.47, 26.62, 26.56, 23.39, 21.31, 20.37, 18.68, 13.74.

HRMS (ESI) *m/z* for $C_{30}H_{45}O_4$ $[M+H]^+$ calcd.: 511.3418, found: 511.3410 (residual: 1.52 ppm).

Compound 7b:

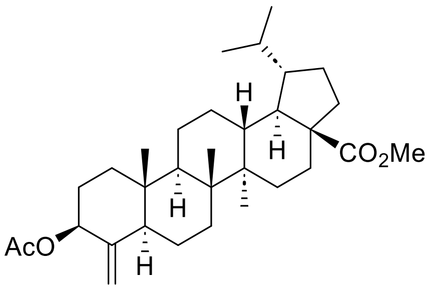

Compound **7b** was synthesized following the general procedure from **3b** (114.5 mg, 0.230 mmol, 1.00 equiv). The crude product was purified by flash column chromatography on silica gel (hexanes/EtOAc, 0% to 30%) to afford **7b** as a colorless solid (95.2 mg, 83%).

^1H NMR (500 MHz, $CDCl_3$) δ 5.11 (dd, *J* = 11.6, 5.8 Hz, 1H), 4.81 (dt, *J* = 2.2, 1.2 Hz, 1H), 4.59 (d, *J* = 1.9 Hz, 1H), 3.65 (s, 2H), 2.24 (ttd, *J* = 12.4, 6.2, 3.4 Hz, 3H), 2.11 (s, 3H), 1.90 (dddd, *J* = 12.7, 6.6, 4.3, 2.6 Hz, 1H), 1.86 – 1.75 (m, 3H), 1.73 – 1.60 (m, 2H), 1.57 – 1.07 (m, 17H), 0.96 (s, 3H), 0.93 (s, 3H), 0.85 (d, *J* = 6.9 Hz, 3H), 0.75 (d, *J* = 6.8 Hz, 3H), 0.66 (s, 3H).

$^{13}C\{^1H\}$ NMR (126 MHz, $CDCl_3$) δ 199.96, 177.07, 170.39, 169.77, 147.35 (R_2**C**$=CH_2$), 128.55, 103.82 ($R_2C=$**C**H_2), 74.63, 59.48, 51.94, 50.68, 48.66, 45.52, 44.19, 43.56, 41.28, 38.90, 38.15, 37.89, 32.00, 31.28, 31.23, 29.09, 28.70, 28.47, 26.62, 26.56, 23.39, 21.31, 20.37, 18.68, 13.74.

HRMS (ESI) *m/z* for $C_{32}H_{54}NO_4$ $[M+NH_4]^+$ calcd.: 516.4047, found: 516.4062 (residual: 2.86 ppm).

Compound 8b:

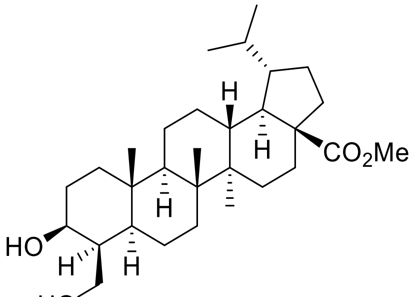

Adapted from a procedure reported by Li.[14]

On the benchtop, to a solution of the olefin **7b** (24.9 mg, 0.0500 mmol, 1.00 equiv) in anhydrous THF (0.500 mL) was added BH_3·THF (1.0 M in THF, 12.5 μL, 0.125 mmol, 2.50 equiv) at 0 °C. The reaction mixture was stirred at room temperature for 6 h until the alkene was completely

consumed (by TLC analysis). The reaction mixture was then cooled to 0 °C. NaOH (3.0 M, 50 µL, 3.00 equiv) and H_2O_2 (30% aq. solution, 29 µL, 5.00 equiv) were added sequentially. The reaction was allowed to warm to room temperature and stirred for 16 h. After completion, the reaction was quenched with a solution of saturated aqueous $Na_2S_2O_3$ (5 mL), and the product was extracted with EtOAc (3 x 5 mL). The combined organic layers were washed with brine, dried over $MgSO_4$, filtered, and concentrated under reduced pressure. The crude mixture was purified by column chromatography on silica (hexanes/EtOAc, 0% to 70%) to afford diol **8b** as a colorless solid (15.7 mg, 66%).

^{1}H NMR ^{1}H NMR (500 MHz, $CDCl_3$) δ 3.96 (t, *J* = 10.5 Hz, 1H), 3.89 (dt, *J* = 10.8, 5.1 Hz, 1H), 3.64 (s, 3H), 3.56 (d, *J* = 11.0 Hz, 1H), 2.95 (s, 1H), 2.61 (s, 1H), 2.31 – 2.15 (m, 3H), 2.11 (dt, *J* = 9.9, 5.0 Hz, 1H), 1.86 – 1.75 (m, 2H), 1.73 – 1.55 (m, 5H), 1.51 – 1.39 (m, 4H), 1.36 – 1.09 (m, 11H), 0.93 (s, 3H), 0.88 (s, 3H), 0.84 (d, *J* = 6.8 Hz, 3H), 0.73 (d, *J* = 6.8 Hz, 3H), 0.66 (s, 3H).

^{13}C{^{1}H} NMR (126 MHz, $CDCl_3$) δ 177.00, 75.01, 60.65, 57.11, 51.33, 49.41, 49.05, 48.94, 48.21, 44.31, 42.74, 41.35, 38.76, 38.24, 37.43, 36.14, 33.93, 32.18, 29.88, 29.77, 27.23, 26.98, 24.32, 23.11, 22.90, 21.38, 15.97, 15.69, 14.81, 14.74.

HRMS (ESI) *m/z* for $C_{32}H_{53}NO_4Na$ $[M+ACN+Na]^+$ calcd.: 538.3867, found: 538.3876 (residual: 1.74 ppm).

Compound 7c:

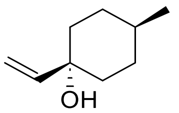

Compound **7c** was synthesized following the general procedure from **3e** (16.5 mg, 0.0886 mmol, 1.00 equiv). The crude product was purified by flash column chromatography on silica gel (pentane/Et_2O, 0% to 40%) to afford **7c** as a colorless solid (10.6 mg, 85%).

Due to the high volatility of **7c**, the yield of this compound was first calculated without complete removal of pentane and the mass of pentane was subtracted (amount of pentane determined by ^{1}H NMR spectroscopy). The compound was then carefully dried *in vacuo* and ^{1}H and ^{13}C NMR spectra were recorded.

The ^{1}H and ^{13}C NMR spectra of this compound were identical to those reported by Fernández.[15]

^{1}H NMR (500 MHz, $CDCl_3$) δ 6.08 (dd, *J* = 17.5, 10.8 Hz, 1H), 5.31 (dd, *J* = 17.5, 1.3 Hz, 1H), 5.14 (dd, *J* = 10.9, 1.3 Hz, 1H), 1.84 – 1.77 (m, 2H), 1.72 – 1.64 (m, 2H), 1.57 (d, *J* = 3.5 Hz, 2H), 1.55 (s, 1H), 1.09 (dddd, *J* = 19.4, 12.3, 9.7, 5.4 Hz, 3H), 0.91 (d, *J* = 6.6 Hz, 3H).

^{13}C{^{1}H} NMR (126 MHz, $CDCl_3$) δ 143.57, 113.53, 72.23, 37.98, 31.68, 31.48, 29.86, 21.40, 7.12.

Compound 7d:

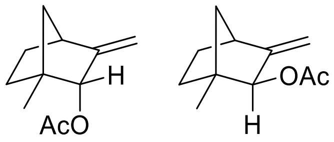

10 : 1

Compound **7d** was synthesized following the general procedure from **3d** (34.0 mg, 0.150 mmol, 1.00 equiv). The crude product was purified by flash column chromatography on silica gel (pentane/Et_2O, 0% to 40%) to afford **7d** as a colorless oil (17.6 mg, 65%) as a 10:1 ratio of diastereomers.

Major diastereomer:

^{1}H NMR (400 MHz, $CDCl_3$) δ 5.29 (d, *J* = 2.3 Hz, 1H), 4.90 (d, *J* = 2.5 Hz, 1H), 4.77 (d, *J* = 2.1 Hz, 1H), 2.68 (d, *J* = 4.1 Hz, 1H), 2.12 (s, 3H), 1.89 – 1.75 (m, 2H), 1.51 – 1.32 (m, 4H), 1.29 – 1.15 (m, 1H), 1.11 (s, 3H).

^{13}C{^{1}H} NMR (126 MHz, $CDCl_3$) δ 171.73, 154.61, 105.66, 80.05, 47.78, 45.54, 42.96, 32.11, 27.74, 21.35, 18.71.

HRMS (ESI) *m/z* for $C_{13}H_{19}NO_2Na$ $[M+ACN+Na]^+$ calcd.: 244.1308, found: 244.1313 (residual: 2.19 ppm).

Minor diastereomer (only distinct peaks included):

^{1}H NMR (400 MHz, $CDCl_3$) δ 5.03 (m, 1H), 5.01 (m, 1H), 4.96 (m, 1H), 2.07 (s, 3H), 1.09 (s, 3H).

In the minor diastereomer, the proton adjacent to –OAc has a lower chemical shift (δ 5.01 vs δ 5.29) than that of the major diastereomer. This difference is consistent with this proton being pseudoaxial rather than pseudoequatorial. The epimerization to form the minor diasteromer likely occurred by ionization or some type of degenerate substitution of the allylic OAc group.

6. Integration of Methyl Groups

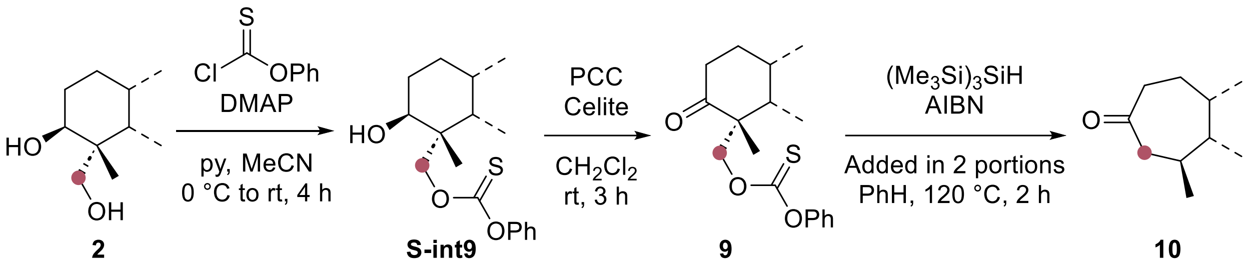

General procedure for selective formation of a thionocarbonate from 1,3-diols (GP-9)

On the benchtop, diol **2** (1.00 equiv) and DMAP (10 mol%) were weighed into a screw-capped one-dram vial. A magnetic stir bar was added, and dry MeCN (0.10 M) was added, followed by pyridine (2.00 equiv). The vial was cooled to 0 °C and O-phenyl chlorothionoformate (1.20 equiv) was added. The resulting solution was allowed to warm to room temperature and stirred for 3 h or until complete consumption of the starting material, as determined by TLC analysis. More O-phenyl chlorothionoformate was added in some cases to achieve full conversion. The reaction was quenched with 2 M HCl, and the resulting mixture was extracted three times with CH_2Cl_2. The combined organic layers were dried over $MgSO_4$ and concentrated by rotary evaporation. The crude mixture was purified by column chromatography on silica gel to give **S-int9**.

General procedure for oxidation of the secondary alcohol (GP-10)

On the bench top, alcohol **S-int9** (1.00 equiv), pyridinium chlorochromate (1.50 equiv) and Celite (twice the mass of PCC used) were weighed into a 20 mL screw-capped vial equipped with a magnetic stir bar. CH_2Cl_2 (0.20 M) was added, the vial was sealed with a Teflon-lined screw cap, and the reaction was stirred for 4 h at room temperature or until complete consumption of starting material, as determined by TLC analysis. The crude reaction mixture was diluted with CH_2Cl_2, passed through a Celite plug, and concentrated by rotary evaporation. The crude product was purified by column chromatography on silica gel to give ketone **9**.

General procedure for Dowd-Beckwith ring expansion of the thionocarbonate (GP-11)

In a nitrogen-filled glovebox, ketone **9** (1.00 equiv) and AIBN (0.150 equiv) were added to a 20 mL screw-capped vial equipped with a magnetic stir bar. Benzene (0.03 M) and tris(trimethylsilyl)silane (0.750 equiv) were then added, and the vial was sealed with a Teflon-lined screw cap. The reaction mixture was heated at 120 °C for 1 h and then transferred back into the glovebox. A second portion of AIBN (0.150 equiv) and tris(trimethylsilyl)silane (0.750 equiv) was added. The vial was sealed and heated at 120 °C for 1 h. The reaction was diluted with Et_2O and washed twice with sat. aq. Na_2CO_3 (to remove the phenol byproduct), dried over $MgSO_4$, filtered, and concentrated by rotary evaporation. The crude mixture was purified by column chromatography on silica gel to give product **10**. Note: The retention factors of the products are often similar to those of the starting materials. Careful TLC with a lower-polarity solvent mixture is often necessary to differentiate the two. In addition, the products are usually UV-inactive, while the starting materials are UV-active.

Investigation of conditions for oxidation of the secondary alcohol

Several conditions were investigated for the oxidation of alcohol **S-int9** to ketone **9**. The results of the investigation are summarized in the table below.

Conditions	Yield of 9	Remarks
Dess-Martin periodinane, CH_2Cl_2, rt, 16 h	0%	Decomp.
Dess-Martin periodinane, $NaHCO_3$, CH_2Cl_2, 0 °C to rt, 1.5 h	11%	
$(COCl)_2$, DMSO, CH_2Cl_2 -78 °C, then Et_3N, 0 °C<br>[Swern oxidation]	47%<br><5%	With fenchol derivative<br>With methyl glycyrrhetinate derivative
Py·SO3, DMSO, Et_3N, rt, 2 h<br>[Parikh-Doering oxidation]	0%	No conversion
N-methylmorpholine oxide, TPAP (10 mol%), 3A mol. sieves, CH_2Cl_2<br>[Ley-Griffith oxidation]	22%	
N-methylmorpholine oxide, $Cu(MeCN)_4PF_6$ (10 mol%), 4,4'-dimethoxybipyridine (10 mol%), ABNO (5 mol%), N-methylimidazole (20 mol%), MeCN/THF, O_2 (1 atm), 65 °C, 16 h<br>[Stahl oxidation]	0%	No conversion
Pyridinium chlorochromate, Celite, CH_2Cl_2, rt, 4 h	81%	

Dowd-Beckwith ring expansion of fenchol-derived thionocarbonate in C_6D_6

In a nitrogen-filled glovebox, fenchol-derived ketone **9e** (4.8 mg, 0.0159 mmol, 1.00 equiv) and AIBN (0.5 mg, 0.0032 mmol, 0.20 equiv) were added to a one-dram screw-capped vial equipped with a magnetic stir bar. Benzene-d_6 (0.03 M, 500 μL) and tris(trimethylsilyl)silane (7.36 μL, 0.0239 mmol, 1.50 equiv) were then added, and the vial was sealed with a Teflon-lined screw cap. The reaction mixture was heated at 120 °C for 4 h. The reaction mixture was allowed to cool to room temperature, transferred to an NMR tube, and analyzed by ^1H NMR spectroscopy.

As the thionocarbonate moiety had been fully converted into phenol, the aromatic protons of phenol were used as an internal standard. (−)-Fenchone was formed in 77% yield. No other ketone products were observed in more than trace yield based on NMR spectroscopy. The presence of (−)-fenchone was also confirmed by GC-MS analysis against an authentic standard.

Compound 9a:

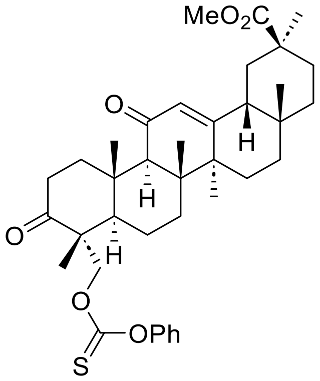

Compound **9a** was synthesized following the general procedure from **S-int9a** (101.9 mg, 0.160 mmol, 1.00 equiv). The crude product was purified by flash column chromatography (SiO_2, hexanes/EtOAc 0% to 30%) to afford **9a** as a colorless solid (80.3 mg, 79%).

9a could not be isolated as a completely pure material, even after column chromatography. Regardless, the crude **9a** was carried forward to the Dowd-Beckwith ring expansion, whereupon the desired product was isolated in pure form.

^{1}H NMR (500 MHz, $CDCl_3$) δ 7.39 (t, *J* = 7.9 Hz, 2H), 7.30 – 7.27 (m, 1H), 7.16 – 6.90 (m, 2H), 5.72 (s, 1H), 4.61 (d, *J* = 10.6 Hz, 1H), 4.39 (d, *J* = 10.6 Hz, 1H), 3.70 (s, 3H), 2.94 (ddd, *J* = 13.6, 7.2, 2.7 Hz, 1H), 2.63 (ddd, *J* = 17.5, 12.6, 7.1 Hz, 1H), 2.39 (s, 1H), 2.31 (ddd, *J* = 17.6, 6.0, 2.7 Hz, 1H), 2.12 (dd, *J* = 13.0, 4.4 Hz, 1H), 2.09 – 1.99 (m, 2H), 1.97 – 1.90 (m, 1H), 1.85 (dt, *J* = 14.4, 7.2 Hz, 1H), 1.38 (s, 3H), 1.30 (s, 2H), 1.18 (s, 3H), 1.17 (s, 3H), 1.07 (s, 3H), 0.91 – 0.84 (m, 1H), 0.83 (s, 3H).

Compound 10a:

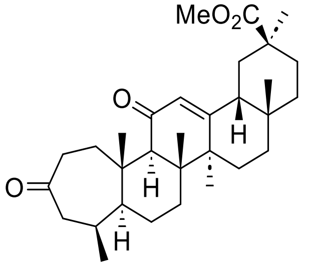

Compound **10a** was synthesized following the general procedure from **9a** (37.0 mg, 0.0583 mmol, 1.00 equiv). The crude product was purified by flash column chromatography (SiO_2, hexanes/EtOAc 0% to 30%) to afford **10a** as a colorless solid (13.5 mg, 48%). Overall yield: 24% over 3 steps from **2d**.

^{1}H NMR (500 MHz, $CDCl_3$) δ 5.67 (s, 1H), 3.68 (s, 3H), 2.89 (dd, *J* = 11.6, 4.5 Hz, 1H), 2.55 (s, 3H), 2.43 (dd, *J* = 11.6, 6.9 Hz, 1H), 2.37 – 2.23 (m, 1H), 2.09 (dd, *J* = 13.7, 4.2 Hz, 1H), 2.05 – 1.97 (m, 3H), 1.92 (ddd, *J* = 13.5, 4.4, 2.6 Hz, 1H), 1.86 – 1.73 (m, 5H), 1.70 – 1.56 (m, 2H), 1.39 (s, 5H), 1.33 – 1.27 (m, 3H), 1.25 – 1.18 (m, 1H), 1.16 (s, 3H), 1.14 (s, 3H), 1.13 (s, 4H), 1.04 – 0.99 (m, 1H), 0.97 (d, *J* = 7.5 Hz, 3H, $R_2CH\mathbf{C}H_3$), 0.80 (s, 3H).

$^{13}C\{^1H\}$ NMR (151 MHz, $CDCl_3$) δ 214.53, 199.96, 177.04, 169.04, 128.94, 58.11, 53.50, 51.93, 49.60, 48.54, 45.78, 44.19, 43.75, 41.20, 40.84, 40.08, 37.91, 36.82, 36.76, 32.46, 32.05, 31.30, 28.68, 28.47, 26.68, 26.57, 24.81, 23.28, 19.10, 16.18, 15.81.

HRMS (ESI) *m/z* for $C_{33}H_{49}NO_4Na$ $[M+ACN+Na]^+$ calcd.: 546.3554, found: 546.3548 (residual: 1.03 ppm).

Compound S-int9b:

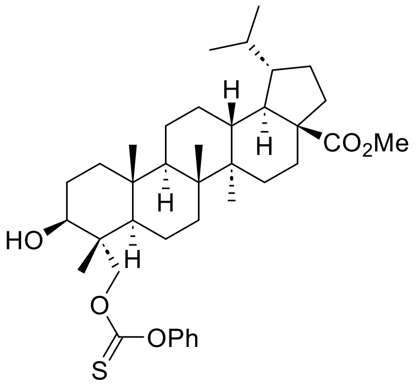

Compound **S-int9b** was synthesized following the general procedure from diol **2i** (97.8 mg, 0.200 mmol, 1.00 equiv). The crude product was purified by flash column chromatography (SiO_2, hexanes/EtOAc 0% to 30%) to afford **S-int9b** as a colorless solid (74.7 mg, 60%).

^1H NMR (600 MHz, $CDCl_3$) δ 7.43 (dd, *J* = 8.5, 7.3 Hz, 2H), 7.30 (t, *J* = 7.4 Hz, 1H), 7.17 – 7.08 (m, 2H), 4.56 (d, *J* = 11.2 Hz, 1H), 4.31 (d, *J* = 11.2 Hz, 1H), 3.65 (s, 3H), 3.53 (dd, *J* = 10.7, 5.7 Hz, 1H), 2.30 – 2.17 (m, 3H), 1.80 (dd, *J* = 11.8, 7.1 Hz, 2H), 1.74 – 1.61 (m, 3H), 1.50 – 1.44 (m, 4H), 1.42 – 1.12 (m, 12H), 1.01 (dd, *J* = 10.9, 2.9 Hz, 1H), 0.96 (s, 3H), 0.92 (s, 3H), 0.91 – 0.87 (m, 2H), 0.87 (s, 3H), 0.86 (d, *J* = 6.9 Hz, 3H), 0.81 (s, 3H), 0.76 (d, *J* = 6.7 Hz, 3H).

$^{13}C\{^1H\}$ NMR (126 MHz, $CDCl_3$) δ 194.89, 176.99, 153.30, 129.71, 126.75, 121.95, 76.75, 72.24, 57.14, 51.33, 50.41, 49.07, 48.24, 44.34, 42.73, 42.70, 40.79, 38.43, 38.22, 37.44, 37.26, 34.09, 32.20, 29.90, 29.82, 27.01, 26.86, 23.13, 22.91, 21.10, 18.47, 16.65, 16.07, 14.92, 14.85, 11.82.

Compound 9b:

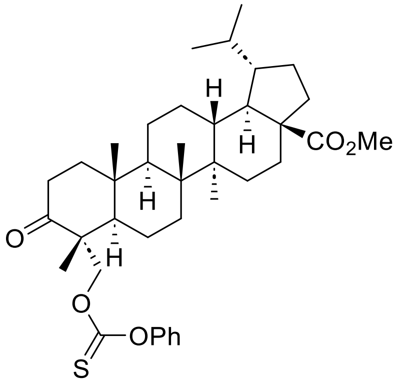

Compound **9b** was synthesized following the general procedure from **S-int9b** (72.9 mg, 0.117 mmol, 1.00 equiv). The crude product was purified by flash column chromatography (SiO_2, hexanes/EtOAc 0% to 20%) to afford **9b** as a colorless solid (61.5 mg, 84%).

^{1}H NMR (700 MHz, $CDCl_3$) δ 7.40 (t, *J* = 7.7 Hz, 2H), 7.28 (dd, *J* = 15.1, 7.6 Hz, 1H), 7.08 (d, *J* = 8.0 Hz, 2H), 4.53 (d, *J* = 10.5 Hz, 1H), 4.38 (d, *J* = 10.6 Hz, 1H), 3.65 (s, 3H), 2.50 (ddd, *J* = 18.6, 11.6, 7.6 Hz, 1H), 2.34 (ddd, *J* = 17.4, 6.8, 2.7 Hz, 1H), 2.25 (m, 3H), 1.91 (ddd, *J* = 13.5, 7.7, 2.9 Hz, 1H), 1.80 (dd, *J* = 11.9, 7.1 Hz, 2H), 1.73 – 1.68 (m, 1H), 1.63 – 1.43 (m, 4H), 1.42 – 1.31 (m, 8H), 1.29 – 1.12 (m, 4H), 1.03 (s, 3H), 0.96 (m, 9H, three overlapping methyl peaks), 0.86 (m, 4H, methyl peak overlapping with another peak), 0.76 (d, *J* = 6.7 Hz, 3H).

^{13}C{^{1}H} NMR (176 MHz, $CDCl_3$) δ 213.95, 194.07, 176.94, 153.25, 129.66, 126.67, 121.92, 76.63, 57.09, 51.35, 50.62, 49.42, 48.99, 47.94, 44.27, 42.76, 40.72, 38.23, 37.82, 37.40, 36.66, 35.16, 33.58, 32.11, 29.89, 29.75, 27.00, 23.11, 22.89, 21.43, 19.58, 17.32, 16.04, 15.67, 14.84, 14.72.

HRMS (ESI) *m/z* for $C_{38}H_{54}O_5SNa$ $[M+Na]^+$calcd.: 645.3584, found: 645.3597 (residual: 1.99 ppm).

Compound 10b:

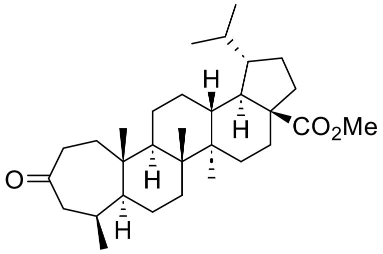

Compound **10b** was synthesized following the general procedure from **9b** (60.7 mg, 0.0974 mmol, 1.00 equiv). The crude product was purified by flash column chromatography (SiO_2, hexanes/EtOAc 0% to 20%) to afford **10b** as a colorless solid (33.3 mg, 73%). Overall yield: 36% over 3 steps from **2d**.

^{1}H NMR (600 MHz, $CDCl_3$) δ 3.64 (s, 3H), 2.86 (dd, *J* = 11.6, 4.7 Hz, 1H), 2.42 (dd, *J* = 11.6, 6.8 Hz, 1H), 2.39 – 2.31 (m, 2H), 2.29 – 2.19 (m, 3H), 1.99 (tt, *J* = 7.2, 4.5 Hz, 1H), 1.92 (dt, *J* = 15.2, 5.1 Hz, 1H), 1.82 – 1.76 (m, 2H), 1.71 (tt, *J* = 12.7, 3.4 Hz, 2H), 1.58 – 1.42 (m, 5H), 1.38 – 1.13 (m, 11H), 0.97 – 0.91 (m, 9H), 0.85 (d, *J* = 6.9 Hz, 3H), 0.79 (s, 3H), 0.75 (d, *J* = 6.8 Hz, 3H).

^{13}C{^{1}H} NMR (126 MHz, $CDCl_3$) δ 214.68, 177.00, 57.15, 53.85, 51.34, 49.88, 48.98, 46.66, 44.24, 43.02, 41.45, 41.35, 40.18, 38.47, 37.44, 37.24, 35.74, 34.18, 32.20, 29.82, 29.73, 27.43, 25.97, 23.11, 22.90, 22.09, 16.78, 16.23, 15.59, 14.83, 14.60.

HRMS (ESI) *m/z* for $C_{33}H_{53}NO_3Na$ $[M+ACN+Na]^+$ calcd.: 534.3918, found: 534.3940 (residual: 4.18 ppm).

Compound 9c:

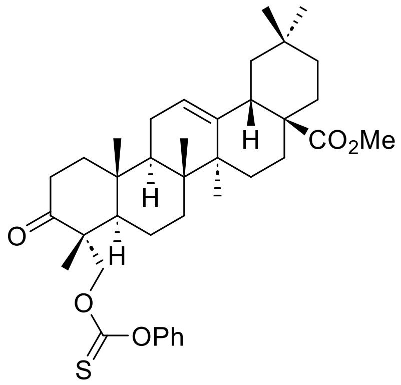

Compound **9c** was synthesized following the general procedure from **S-int9c** (73.0 mg, 0.117 mmol, 1.00 equiv). The crude product was purified by flash column chromatography (SiO_2, hexanes/EtOAc 0% to 20%) to afford **9c** as a colorless solid (54.8 mg, 75%).

^{1}H NMR (500 MHz, $CDCl_3$) δ 5.67 (s, 1H), 3.68 (s, 3H), 2.89 (dd, *J* = 11.6, 4.5 Hz, 1H), 2.55 (s, 3H), 2.43 (dd, *J* = 11.6, 6.9 Hz, 1H), 2.37 – 2.23 (m, 1H), 2.09 (dd, *J* = 13.7, 4.2 Hz, 1H), 2.05 – 1.97 (m, 3H), 1.92 (ddd, *J* = 13.5, 4.4, 2.6 Hz, 1H), 1.86 – 1.73 (m, 5H), 1.70 – 1.56 (m, 2H), 1.39 (s, 5H), 1.33 – 1.27 (m, 3H), 1.25 – 1.18 (m, 1H), 1.16 (s, 3H), 1.14 (s, 3H), 1.13 (s, 4H), 1.04 – 0.99 (m, 1H), 0.97 (d, *J* = 7.5 Hz, 3H, $R_2CH\mathbf{C}H_3$), 0.80 (s, 3H).

^{13}C{^{1}H} NMR (126 MHz, $CDCl_3$) δ 213.72, 194.02, 178.38, 153.26, 144.12, 129.69, 126.70, 122.17, 121.90, 76.77 (shoulder on the peak of $CDCl_3$), 51.72, 50.66, 48.08, 46.87, 46.67, 45.97, 41.96, 41.52, 39.37, 37.38, 36.52, 35.03, 34.00, 33.27, 32.48, 32.08, 30.87, 27.82, 26.06, 23.78, 23.57, 23.17, 19.58, 17.64, 17.02, 15.00.

HRMS (ESI) *m/z* for $C_{38}H_{54}O_5SNa$ $[M+Na]^+$ calcd.: 643.3427, found: 643.3449 (residual: 3.39 ppm).

Compound 10c:

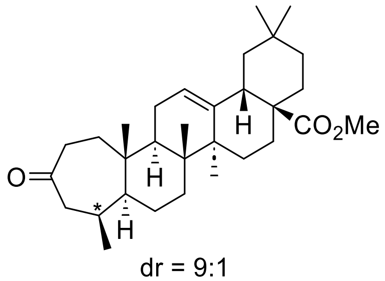

dr = 9:1

Compound **10c** was synthesized following the general procedure from **9c** (53.5 mg, 0.0862 mmol, 1.00 equiv). The crude product was purified by flash column chromatography (SiO_2, hexanes/EtOAc 0% to 20%) to afford **10c** as a colorless solid (28.6 mg, 71%). Overall yield: 32% over 3 steps from **2c**.

^{1}H NMR (600 MHz, $CDCl_3$) δ 5.30 (t, *J* = 3.7 Hz, 1H), 3.61 (s, 3H), 2.85 (td, *J* = 11.5, 4.6 Hz, 2H), 2.43 (dd, *J* = 11.7, 7.0 Hz, 1H), 2.34 (dd, *J* = 8.6, 5.1 Hz, 2H), 2.06 – 1.91 (m, 3H), 1.90 – 1.78

(m, 2H), 1.14 (s, 3H), 0.94 (d, *J* = 7.5 Hz, 3H), 0.92 (s, 3H), 0.89 (s, 3H), 0.87 (s, 3H), 0.77 (s, 3H).

Almost all of the peaks of the minor epimer overlapped with those of the major epimer but a few distinct peaks allowed an estimation of the dr to be 9:1.

$^{13}C\{^{1}H\}$ NMR (151 MHz, $CDCl_3$) δ 214.30, 178.21, 143.74, 122.59, 53.44, 51.52, 49.65, 46.81, 45.68, 43.82, 42.22, 41.47, 41.21, 39.80, 39.78, 36.88, 35.49, 33.88, 33.11, 32.48, 32.33, 30.68, 27.72, 25.66, 25.53, 23.93, 23.61, 23.07, 17.43, 15.81, 15.56.

Peaks of the minor epimer can be seen in the ^{13}C NMR spectrum.

HRMS (ESI) *m/z* for $C_{33}H_{51}NO_3Na$ $[M+ACN+Na]^+$ calcd.: 532.3761, found: 532.3749 (residual: 2.19 ppm).

Compound 9d:

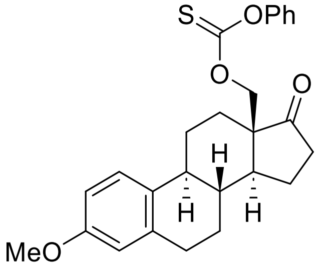

Compound **9d** was synthesized following the general procedure from **S-int9d** (25.5 mg, 0.0581 mmol, 1.00 equiv). The crude product was purified by flash column chromatography (SiO_2, hexanes/EtOAc 0% to 30%) to afford **9d** as a colorless solid (7.1 mg, 28%).

9d could not be isolated as a completely pure material, even after column chromatography. Regardless, the crude **9d** was carried forward to the Dowd-Beckwith ring expansion, whereupon the desired product was isolated in pure form.

Discussion: The reaction of estrone-derived diol **2o** to form **S-int9d** occurred with little to no selectivity for the primary alcohol over the secondary alcohol. We assume that this lack of selectivity is because the hydroxyl group in **2o** is on a very sterically hindered axial methyl group (see Scheme 2 and the discussion in the manuscript), while the secondary alcohol is relatively unhindered. Therefore, the rates of reaction of the two alcohols with *O*-phenyl chlorothionoformate are likely to be similar, and both primary and secondary alcohols are functionalized. Separation of the desired product from the isomer with the thionocarbonyl group on the secondary alcohol and the difunctionalized compound was challenging. This challenge led to a poor yield for the first step of the sequence (~28%). Some impurities remained after purification and likely had a detrimental effect on the yield of the second step (oxidation with PCC to form **9d**). Therefore, the yield of the overall 3-step sequence was significantly lower than for compounds **10a**-**10c**.

^{1}H NMR (400 MHz, C_6D_6) δ 7.03 – 6.94 (m, 6H), 6.86 (ddd, *J* = 8.5, 5.6, 2.4 Hz, 1H), 6.76 (dd, *J* = 8.6, 2.8 Hz, 1H), 6.70 (d, *J* = 2.7 Hz, 1H), 4.82 (d, *J* = 11.6 Hz, 1H), 4.57 (d, *J* = 11.6 Hz, 1H), 3.43 (s, 3H), 2.64 (dd, *J* = 9.6, 4.5 Hz, 2H), 2.50 – 2.39 (m, 1H), 2.39 – 2.28 (m, 1H), 2.06 (dd, *J*

= 11.4, 4.9 Hz, 1H), 1.88 (ddt, *J* = 28.1, 18.6, 9.7 Hz, 2H), 1.59 – 1.50 (m, 1H), 1.46 – 1.13 (m, 5H), 1.01 (ddt, *J* = 15.3, 10.9, 4.7 Hz, 2H).

Compound 10d:

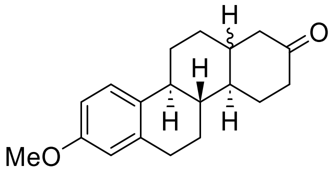

Compound **10d** was synthesized following the general procedure from **9d** (7.1 mg, 0.0163 mmol, 1.00 equiv). The crude product was purified by flash column chromatography (SiO_2, hexanes/EtOAc 0% to 20%) to afford **10d** as a colorless solid (3.7 mg, 80%).

The C-13β diastereomer of this compound had been reported by Johns.[16] The authors did not report ^{1}H NMR spectra, ^{13}C NMR spectra, IR spectra, or HRMS data for the compound.

Major diastereomer, ^{1}H NMR (600 MHz, $CDCl_3$) δ 7.22 (dd, *J* = 11.6, 8.6 Hz, 1H, overlapping), 6.73 (td, *J* = 8.3, 2.8 Hz, 1H, overlapping), 6.64 (dd, *J* = 12.1, 2.8 Hz, 1H), 3.78 (s, 3H), 2.84 (dd, *J* = 7.9, 3.7 Hz, 2H), 2.51 – 2.27 (m, 6H, overlapping), 2.15 (ddt, *J* = 15.8, 10.6, 6.4 Hz, 2H, overlapping), 1.96 – 1.84 (m, 1H, overlapping), 1.80 – 1.66 (m, 2H, overlapping), 1.51 – 1.21 (m, 5H, overlapping).

Minor diastereomer, ^{1}H NMR (600 MHz, $CDCl_3$) δ 7.22 (dd, *J* = 11.6, 8.6 Hz, 1H, overlapping), 6.73 (td, *J* = 8.3, 2.8 Hz, 1H, overlapping), 6.64 (dd, *J* = 12.1, 2.8 Hz, 1H), 3.79 (s, 3H), 2.89 (dd, *J* = 8.9, 4.4 Hz, 2H), 2.60 (t, *J* = 14.2 Hz, 1H), 2.51 – 2.27 (m, 5H, overlapping), 2.15 (ddt, *J* = 15.8, 10.6, 6.4 Hz, 2H, overlapping), 1.96 – 1.84 (m, 1H, overlapping), 1.80 – 1.66 (m, 2H, overlapping), 1.51 – 1.21 (m, 5H, overlapping).

It was not possible to clearly differentiate all the peaks belonging to the major diastereomer from those belonging to the minor diastereomer in the ^{13}C NMR spectrum. Therefore, all observed peaks are reported below, and peaks that clearly belong to the major or minor diastereomer are listed separately.

^{13}C{^{1}H} NMR (151 MHz, $CDCl_3$) δ 212.68, 211.70, 157.75, 157.67, 138.11, 138.00, 132.49, 132.45, 126.72, 126.62, 113.70, 113.60, 111.92, 111.87, 55.38, 55.36, 48.63, 45.97, 44.98, 43.36, 43.02, 42.94, 42.81, 41.37, 40.07, 37.98, 36.75, 35.67, 34.25, 31.00, 30.88, 30.34, 30.32, 30.14, 27.57, 26.57, 26.40, 25.64.

Major diastereomer, distinct peaks, ^{13}C{^{1}H} NMR (151 MHz, $CDCl_3$) δ 211.70, 157.67, 138.11, 132.45, 126.72, 113.60, 111.87, 55.36.

Minor diastereomer, distinct peaks, ^{13}C{^{1}H} NMR (151 MHz, $CDCl_3$) δ 212.68, 157.75, 138.00, 132.49, 126.62, 113.70, 111.92, 55.38.

The ^{1}H NMR spectrum of estrone contains a peak at 0.8-0.9 ppm, and the ^{13}C NMR spectrum contains a peak at 15 ppm corresponding to the C-18 methyl group. Neither are present in the spectra of **10d**, conclusively excluding the possibility that the Barton-McCombie deoxygenation occurred without rearrangement.

HRMS (ESI) *m/z* for $C_{38}H_{49}O_4$ $[2M+H]^+$ calcd.: 591.3444, found: 591.3469 (residual: 4.20 ppm).

7. References

(1) Simmons, E. M.; Hartwig, J. F. Catalytic Functionalization of Unactivated Primary C–H Bonds Directed by an Alcohol. *Nature* **2012**, *483*, 70–73. https://doi.org/10.1038/nature10785.

(2) Dauzonne, D.; Goasdoue, N.; Platzer, N. Carbon-13 NMR Studies of Monohydroxylated and Monochlorinated Derivatives of Z- and E-p-Menthanes. *Org. Magn. Reson.* **1981**, *17*, 18–25. https://doi.org/10.1002/mrc.1270170106.

(3) Na, C. G.; Kang, S. H.; Sarpong, R. Development of a C–C Bond Cleavage/Vinylation/Mizoroki–Heck Cascade Reaction: Application to the Total Synthesis of 14- and 15-Hydroxypatchoulol. *J. Am. Chem. Soc.* **2022**, *144*, 19253–19257. https://doi.org/10.1021/jacs.2c09201.

(4) Chu, L.; Ohta, C.; Zuo, Z.; MacMillan, D. W. C. Carboxylic Acids as A Traceless Activation Group for Conjugate Additions: A Three-Step Synthesis of (±)-Pregabalin. *J. Am. Chem. Soc.* **2014**, *136*, 10886–10889. https://doi.org/10.1021/ja505964r.

(5) Sherwood, T. C.; Li, N.; Yazdani, A. N.; Dhar, T. G. M. Organocatalyzed, Visible-Light Photoredox-Mediated, One-Pot Minisci Reaction Using Carboxylic Acids via N-(Acyloxy)Phthalimides. *J. Org. Chem.* **2018**, *83*, 3000–3012. https://doi.org/10.1021/acs.joc.8b00205.

(6) Dragoli, D. Glycyrrhetinic Acid Derivatives for Use in Treating Hyperkalemia. WO2020163642A1, August 13, 2020.

(7) Zhang, Y.; Qian, J.; Wang, M.; Huang, Y.; Hu, P. Visible-Light-Induced Decarboxylative Fluorination of Aliphatic Carboxylic Acids Catalyzed by Iron. *Org. Lett.* **2022**, *24*, 5972–5976. https://doi.org/10.1021/acs.orglett.2c02242.

(8) Williamson, K. L.; Hsu, Y.-F. L.; Hall, F. H.; Swager, S.; Coulter, M. S. Dihedral Angle and Bond Angle Dependence of Vicinal Proton-Fluorine Spin-Spin Coupling. *J. Am. Chem. Soc.* **1968**, *90*, 6717–6722. https://doi.org/10.1021/ja01026a028.

(9) Li, N.; Ning, Y.; Wu, X.; Xie, J.; Li, W.; Zhu, C. A Highly Selective Decarboxylative Deuteration of Carboxylic Acids. *Chem. Sci.* **2021**, *12*, 5505–5510. https://doi.org/10.1039/D1SC00528F.

(10) Cohen, K. F.; Kazlauskas, R.; Pinhey, J. T. A General Method for Removal of a 4-Methyl Group from Triterpenoids. Synthesis of 4β-Demethylglycyrrhetinic Acid. *J. Chem. Soc., Perkin Trans. 1* **1973**, 2076–2082. https://doi.org/10.1039/P19730002076.

(11) Valterová, I.; Klinot, J.; Vystrčil, A. 3,4-Seco-3,28-Lupanedioic Acids Substituted in the Side Chain at $C_{(5)}$. *Collect. Czech. Chem. Commun.* **1980**, *45*, 1964–1973. https://doi.org/10.1135/cccc19801964.

(12) Johns, S. R.; Lamberton, J. A.; Morton, T. C.; Suares, H.; Willing, R. I. Triterpenes of Lantana Tiliaefolia. 24-Hydroxy-3-Oxours-12-En-28-Oic Acid, a New Triterpene. *Aust. J. Chem.* **1983**, *36*, 2537–2547. https://doi.org/10.1071/ch9832537.

(13) Sun, X.; Chen, J.; Ritter, T. Catalytic Dehydrogenative Decarboxyolefination of Carboxylic Acids. *Nature Chem* **2018**, *10*, 1229–1233. https://doi.org/10.1038/s41557-018-0142-4.

(14) Xu, H.; Tang, H.; Feng, H.; Li, Y. Metal-Mediate Reactions Based Formal Synthesis of Triptonide and Triptolide. *Tetrahedron Lett.* **2014**, *55*, 7118–7120. https://doi.org/10.1016/j.tetlet.2014.11.010.

(15) Miralles, N.; Alam, R.; Szabó, K. J.; Fernández, E. Transition-Metal-Free Borylation of Allylic and Propargylic Alcohols. *Angew. Chem. Int. Ed.* **2016**, *55*, 4303–4307. https://doi.org/10.1002/anie.201511255.

(16) Johns, W. F. Synthesis of 3-Methoxy-17-Acetyl-18-Norestra-1,3,5(10),16-Tetraene. *J. Org. Chem.* **1963**, *28*, 1856–1861. https://doi.org/10.1021/jo01042a029.

8. NMR Spectra

Compound S1:

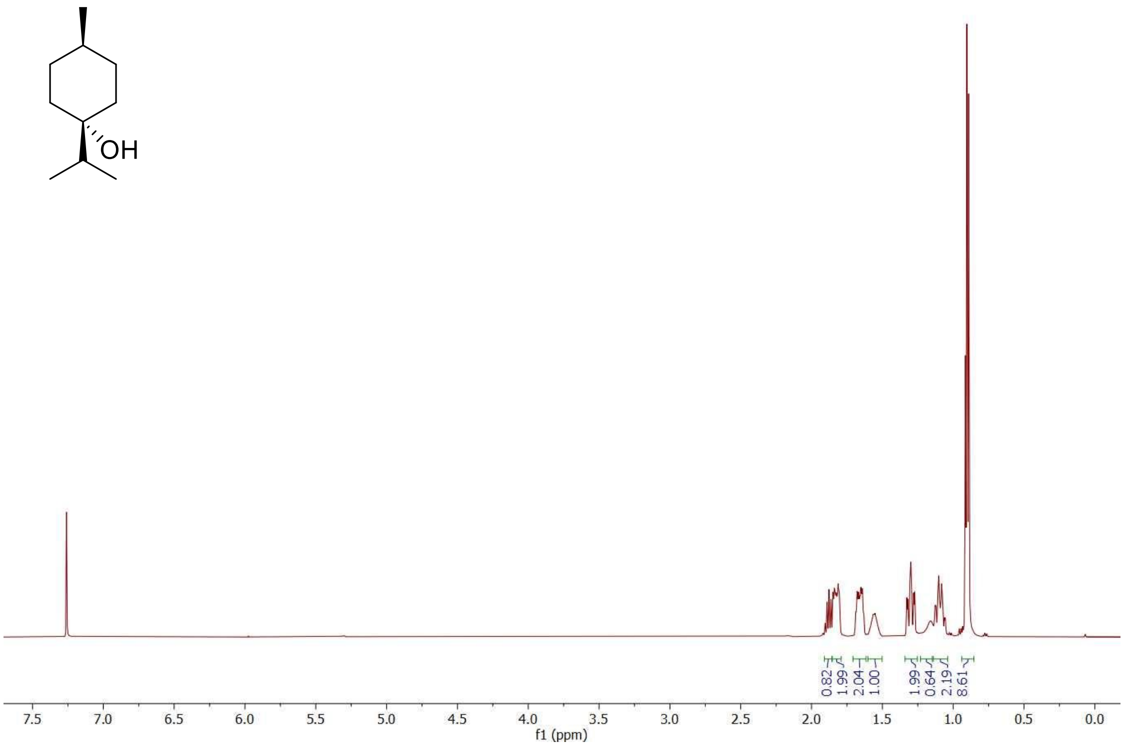

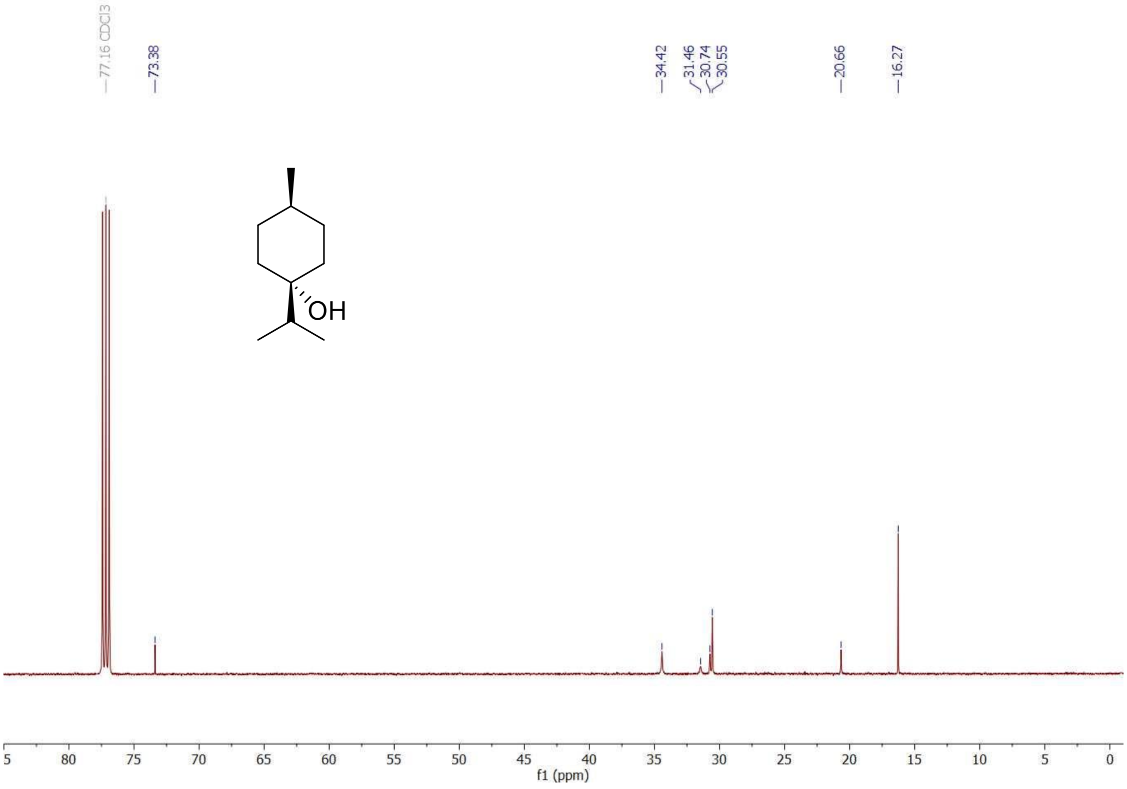

Compound S2:

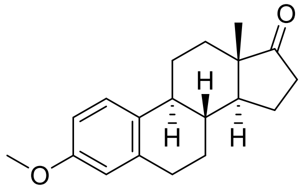
O
H
H
H
O

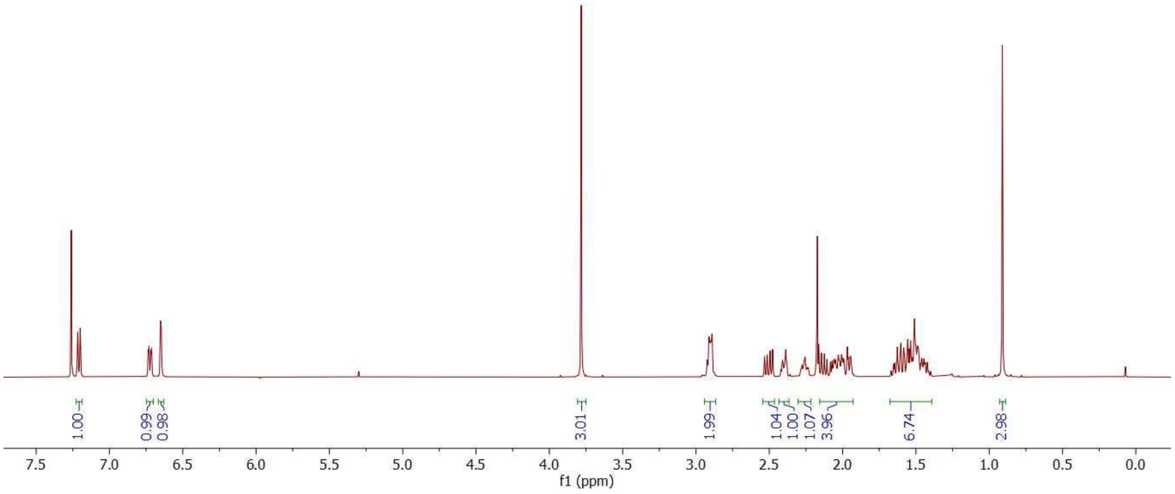
1.00
0.99
0.98
3.01
1.99
1.04
1.00
1.07
3.96
6.74
2.98
7.5 7.0 6.5 6.0 5.5 5.0 4.5 4.0 3.5 3.0 2.5 2.0 1.5 1.0 0.5 0.0
f1 (ppm)

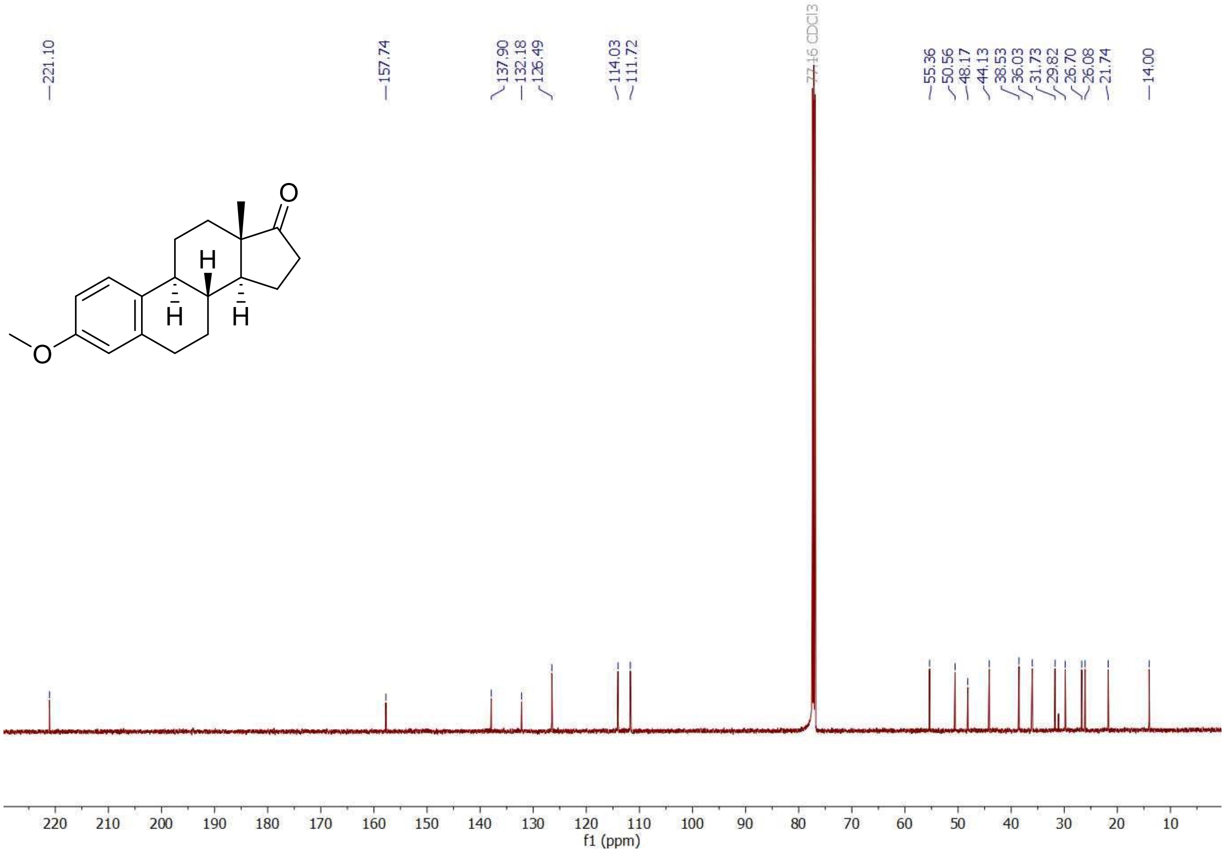
221.10
157.74
137.90
132.18
126.49
114.03
111.72
77.16 CDCl3
55.36
50.56
48.17
44.13
38.53
36.03
31.73
29.82
26.70
26.08
21.74
14.00
220 210 200 190 180 170 160 150 140 130 120 110 100 90 80 70 60 50 40 30 20 10
f1 (ppm)

Compound S3:

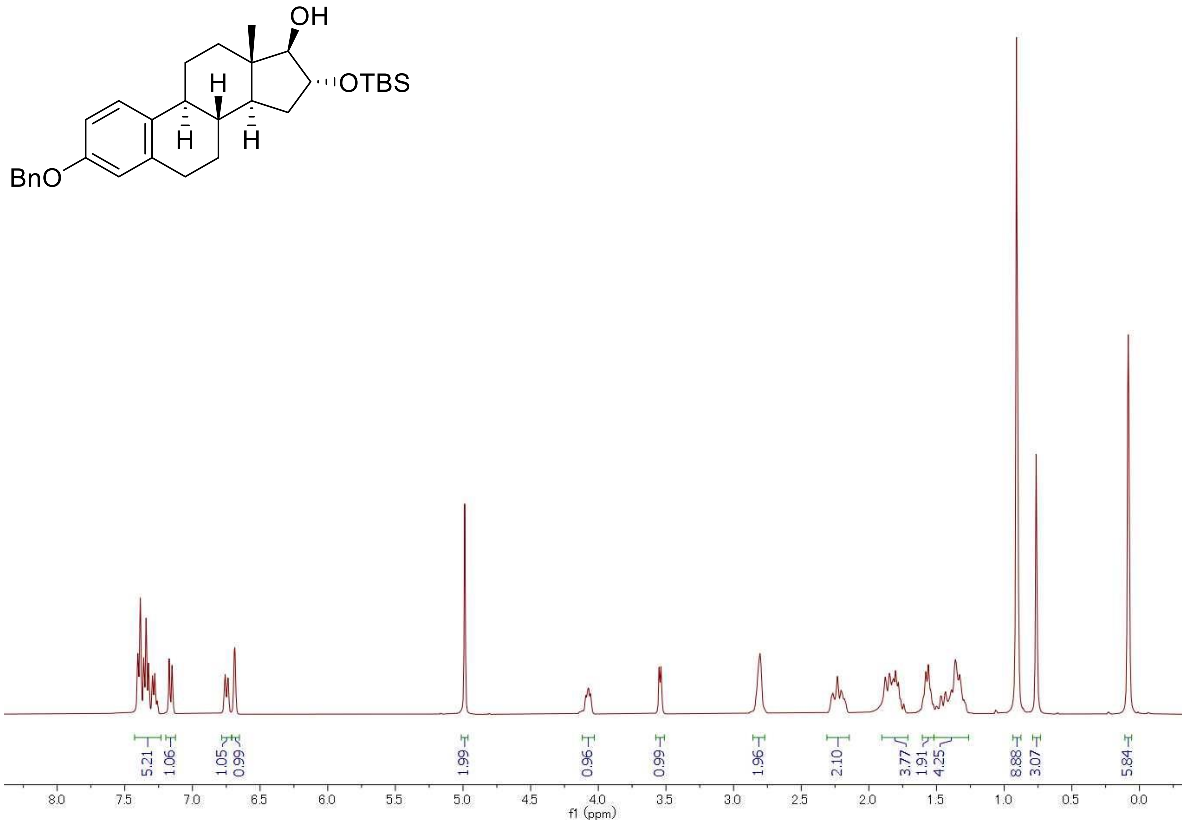

OH
OTBS
H
H
H
BnO
5.21
1.06
1.05
0.99
1.99
0.96
0.99
1.96
2.10
3.77
1.91
4.25
8.88
3.07
5.84
8.0
7.5
7.0
6.5
6.0
5.5
5.0
4.5
4.0
3.5
3.0
2.5
2.0
1.5
1.0
0.5
0.0
f1 (ppm)

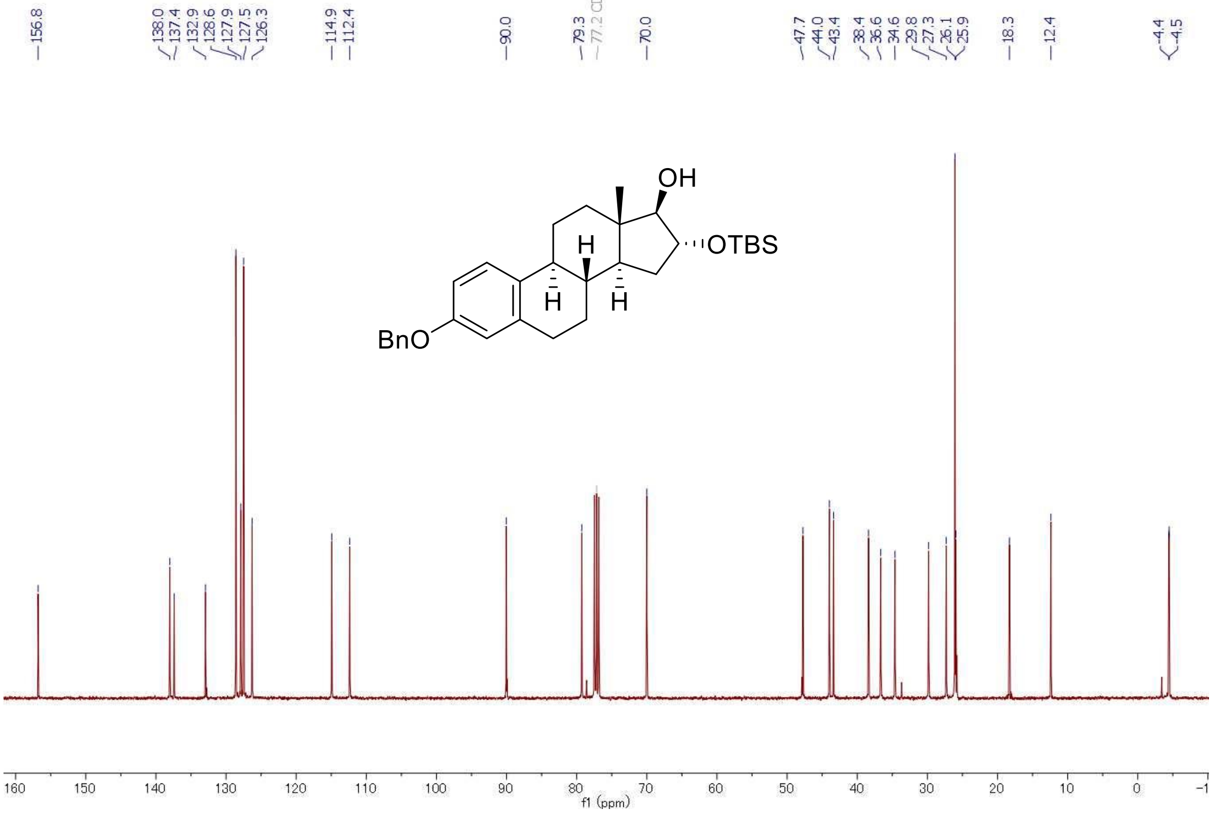

156.8
138.0
137.4
132.9
128.6
127.9
127.5
126.3
114.9
112.4
90.0
79.3
77.2 CDCl3
70.0
47.7
44.0
43.4
38.4
36.6
34.6
29.8
27.3
26.1
25.9
18.3
12.4
-4.4
-4.5
OH
OTBS
H
H
H
BnO
160
150
140
130
120
110
100
90
80
70
60
50
40
30
20
10
0
-1
f1 (ppm)

Compound S4:

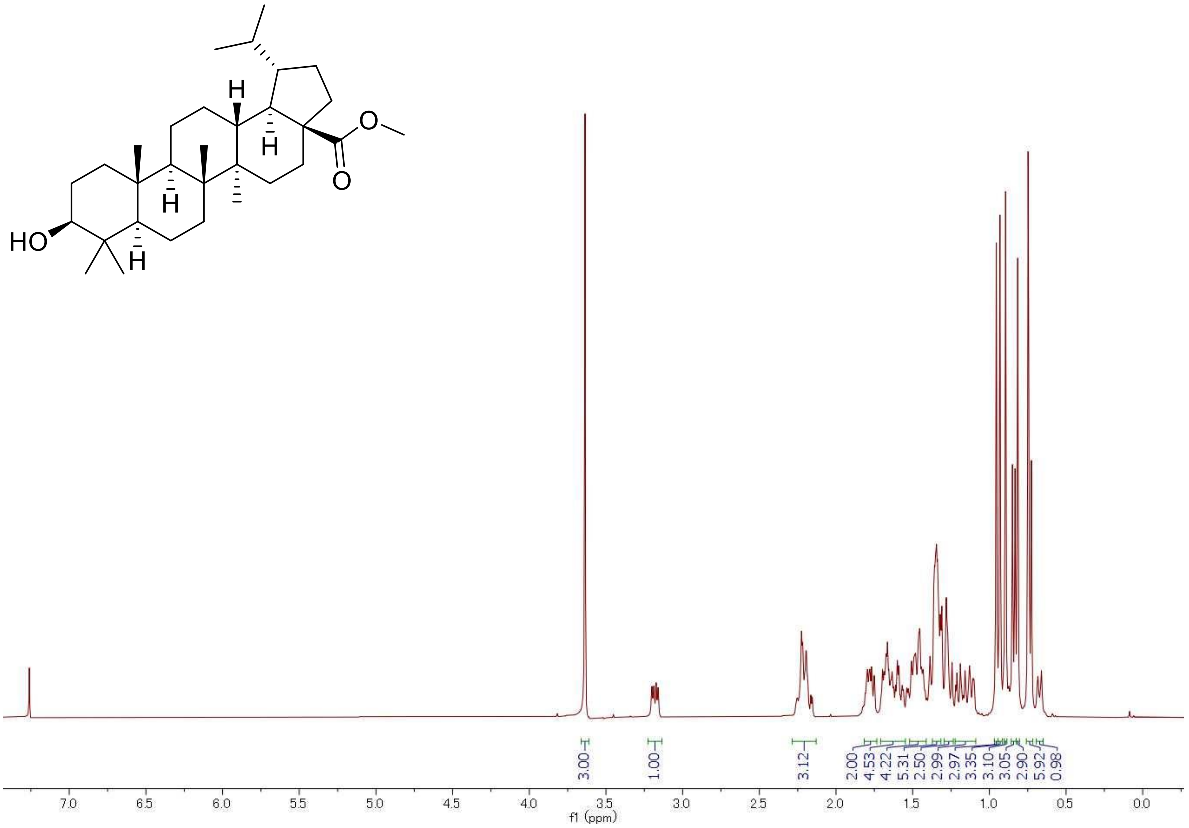

H
H
H
H
HO
O
O
3.00
1.00
3.12
2.00
4.53
4.22
5.31
2.50
2.99
2.97
3.35
3.10
3.05
2.90
5.92
0.98
7.0 6.5 6.0 5.5 5.0 4.5 4.0 3.5 3.0 2.5 2.0 1.5 1.0 0.5 0.0
f1 (ppm)

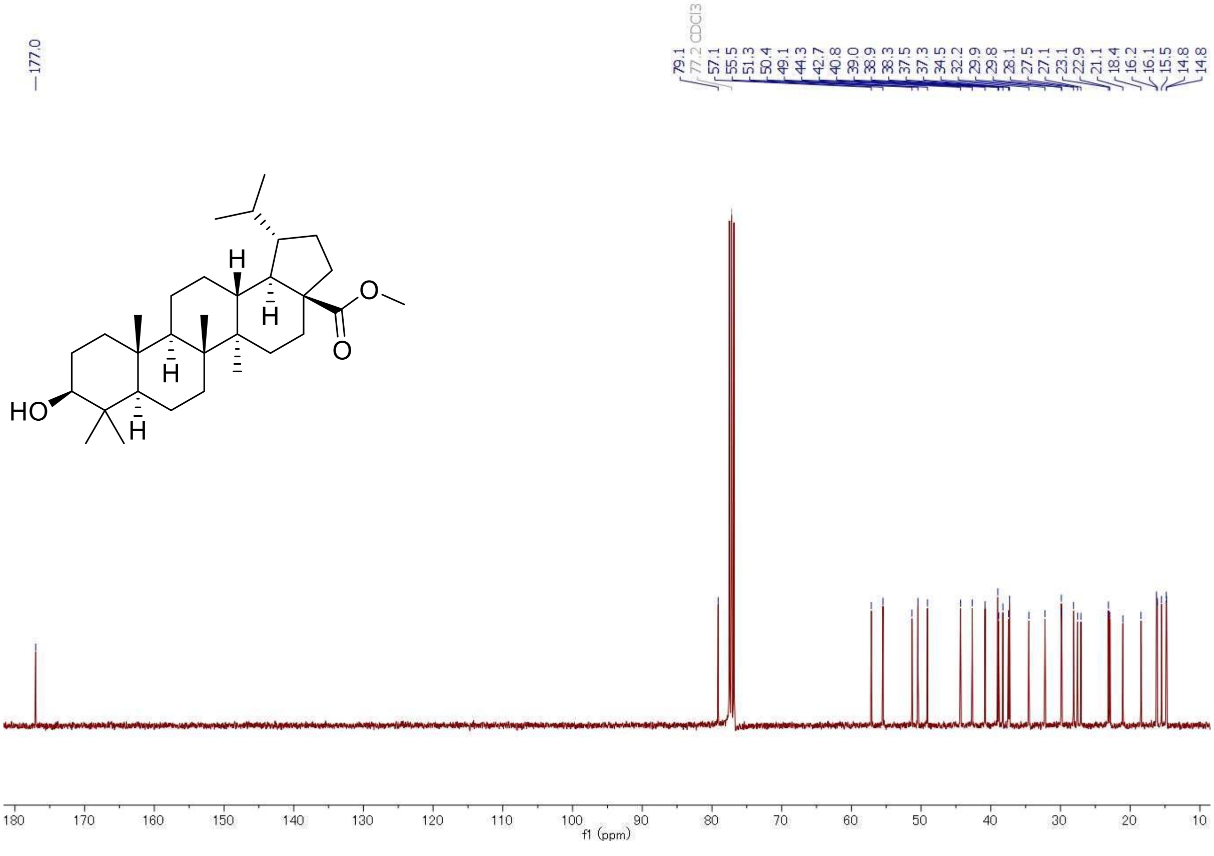

177.0
79.1
77.2 CDCl3
57.1
55.5
51.3
50.4
49.1
44.3
42.7
40.8
39.0
38.9
38.3
37.5
37.3
34.5
32.2
29.9
29.8
28.1
27.5
27.1
23.1
22.9
21.1
18.4
16.2
16.1
15.5
14.8
14.8
H
H
H
H
HO
O
O
180 170 160 150 140 130 120 110 100 90 80 70 60 50 40 30 20 10
f1 (ppm)

Compound S5:

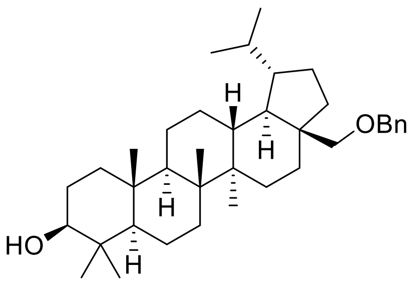

H
H
OBn
H
HO
H

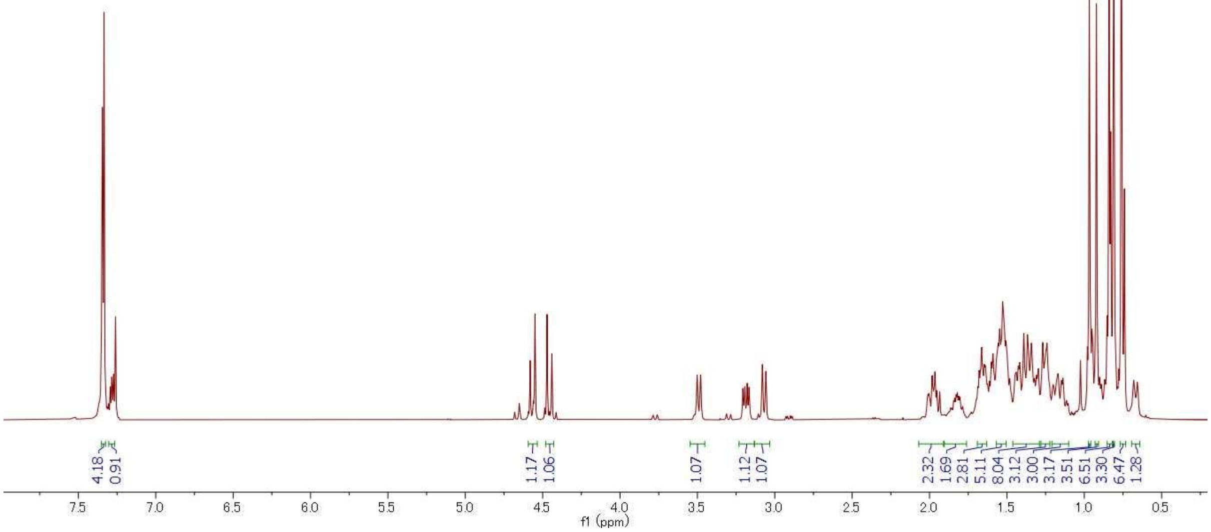

4.18
0.91
1.17
1.06
1.07
1.12
1.07
2.32
1.69
2.81
5.11
8.04
3.12
3.00
3.17
3.51
6.51
3.30
6.47
1.28
7.5 7.0 6.5 6.0 5.5 5.0 4.5 4.0 3.5 3.0 2.5 2.0 1.5 1.0 0.5
f1 (ppm)

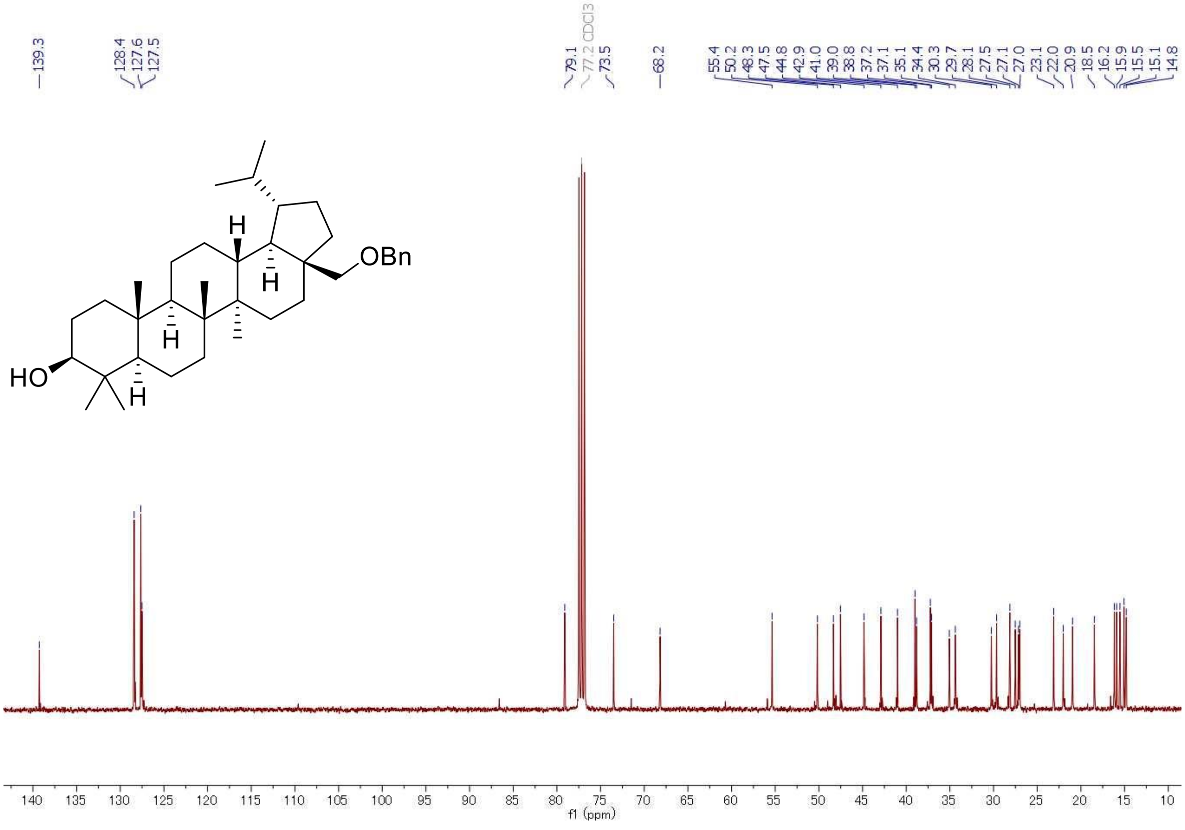

139.3
128.4
127.6
127.5
79.1
77.2 CDCl3
73.5
68.2
55.4
50.2
48.3
47.5
44.8
42.9
41.0
39.0
38.8
37.2
37.1
35.1
34.4
30.3
29.7
28.1
27.5
27.1
27.0
23.1
22.0
20.9
18.5
16.2
15.9
15.5
15.1
14.8
140 135 130 125 120 115 110 105 100 95 90 85 80 75 70 65 60 55 50 45 40 35 30 25 20 15 10
f1 (ppm)

Compound S6:

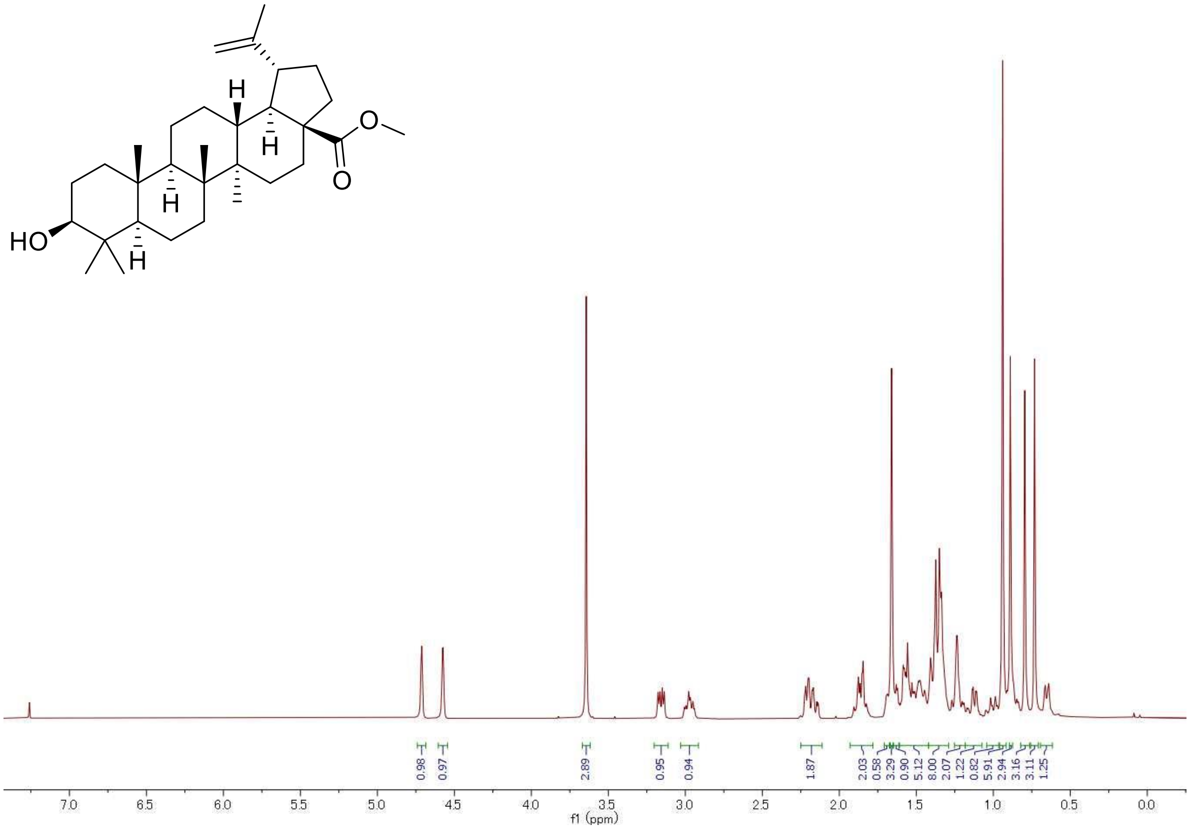
HO
H
H
H
H
O
O
0.98
0.97
2.89
0.95
0.94
1.87
2.03
0.58
3.29
0.90
5.12
8.00
2.07
1.22
0.82
5.91
2.94
3.16
3.11
1.25
7.0
6.5
6.0
5.5
5.0
4.5
4.0
3.5
3.0
2.5
2.0
1.5
1.0
0.5
0.0
f1 (ppm)

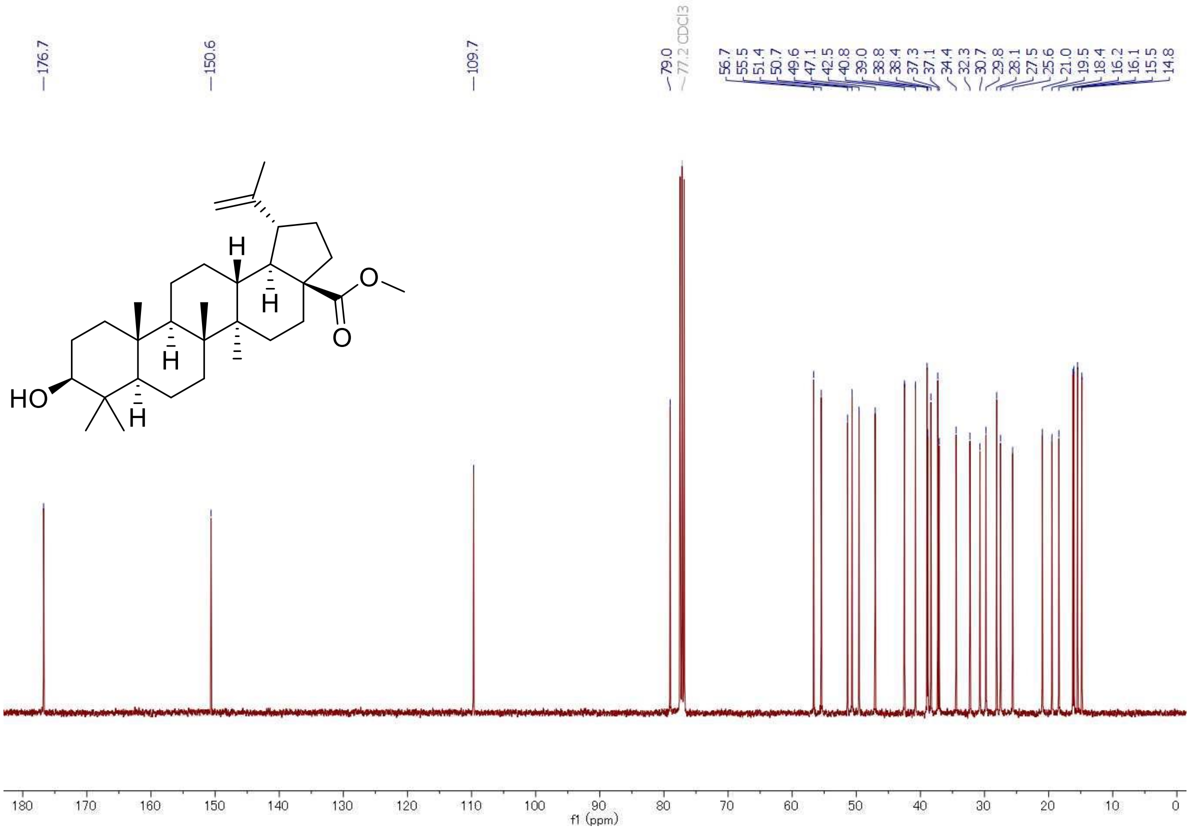
176.7
150.6
109.7
79.0
77.2 CDCl3
56.7
55.5
51.4
50.7
49.6
47.1
42.5
40.8
39.0
38.8
38.4
37.3
37.1
34.4
32.3
30.7
29.8
28.1
27.5
25.6
21.0
19.5
18.4
16.2
16.1
15.5
14.8
HO
H
H
H
O
O
180
170
160
150
140
130
120
110
100
90
80
70
60
50
40
30
20
10
0
f1 (ppm)

Compound S7:

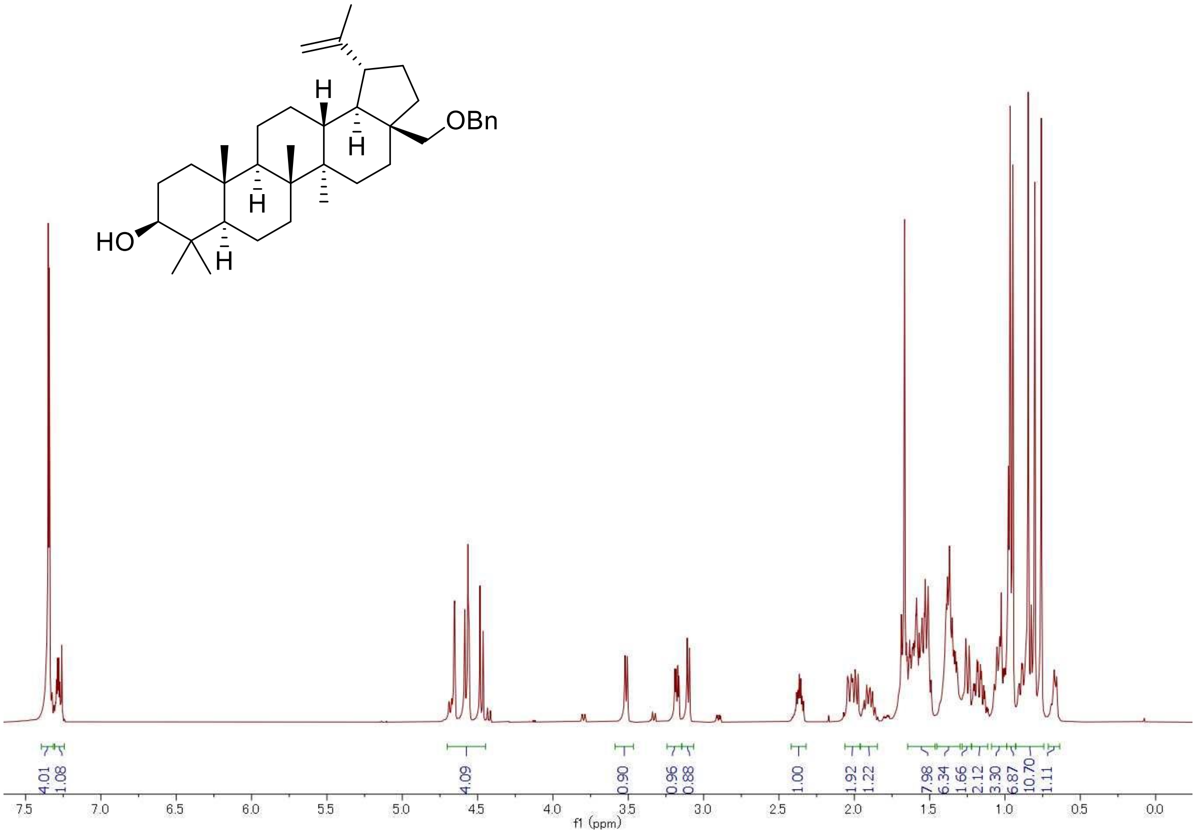
OBn
HO
H
4.01
1.08
4.09
0.90
0.96
0.88
1.00
1.92
1.22
7.98
6.34
1.66
2.12
3.30
6.87
10.70
1.11
7.5 7.0 6.5 6.0 5.5 5.0 4.5 4.0 3.5 3.0 2.5 2.0 1.5 1.0 0.5 0.0
f1 (ppm)

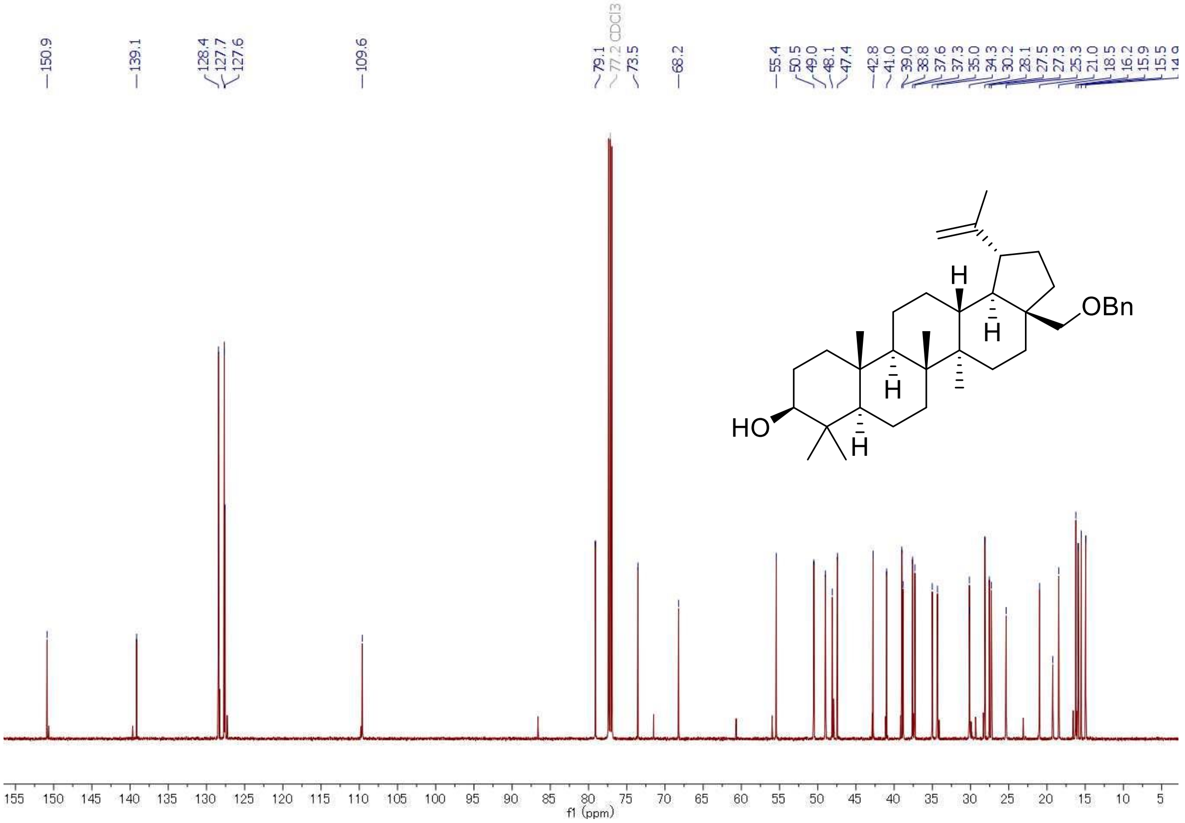
150.9
139.1
128.4
127.7
127.6
109.6
79.1
77.2 CDCl3
73.5
68.2
55.4
50.5
49.0
48.1
47.4
42.8
41.0
39.0
38.8
37.6
37.3
35.0
34.3
30.2
28.1
27.5
27.3
25.3
21.0
18.5
16.2
15.9
15.5
OBn
HO
H
155 150 145 140 135 130 125 120 115 110 105 100 95 90 85 80 75 70 65 60 55 50 45 40 35 30 25 20 15 10 5
f1 (ppm)

Compound S8:

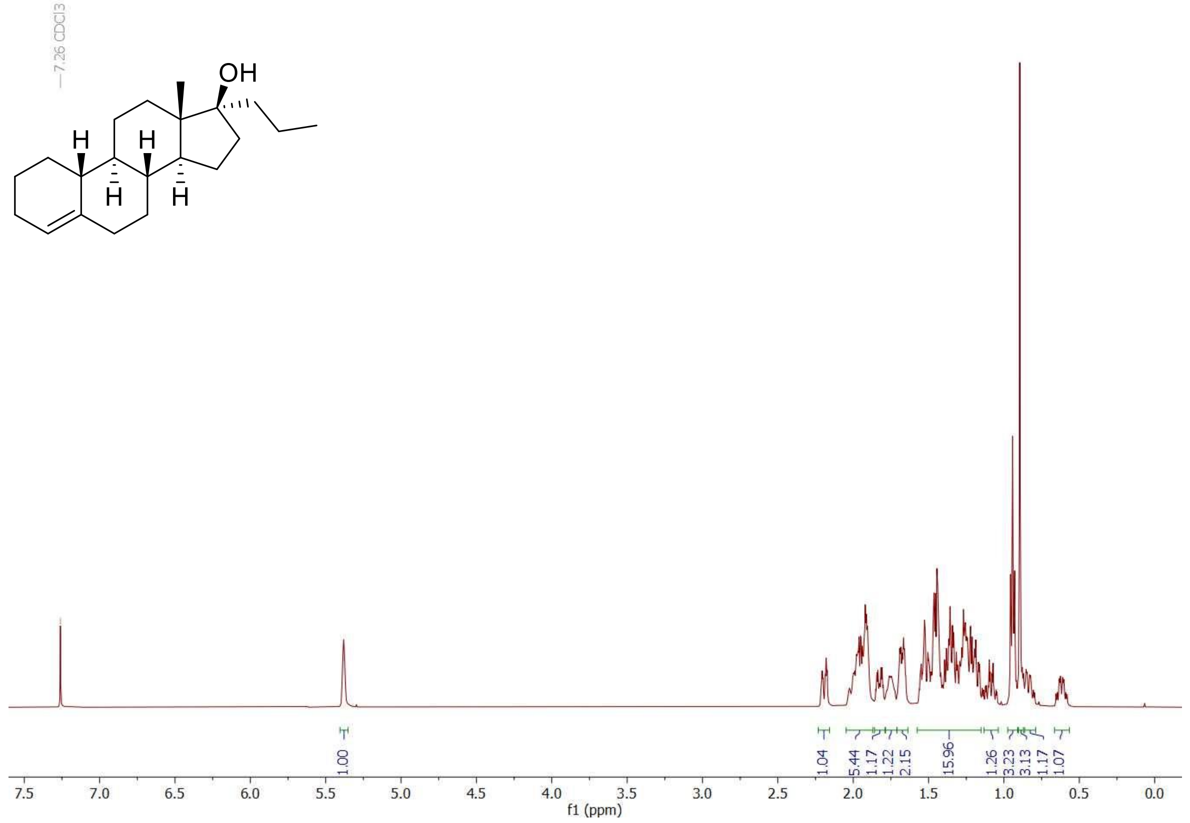

OH
H
H
H
H
7.26 CDCl3
1.00
1.04
5.44
1.17
1.22
2.15
15.96
1.26
3.23
3.13
1.17
1.07
f1 (ppm)

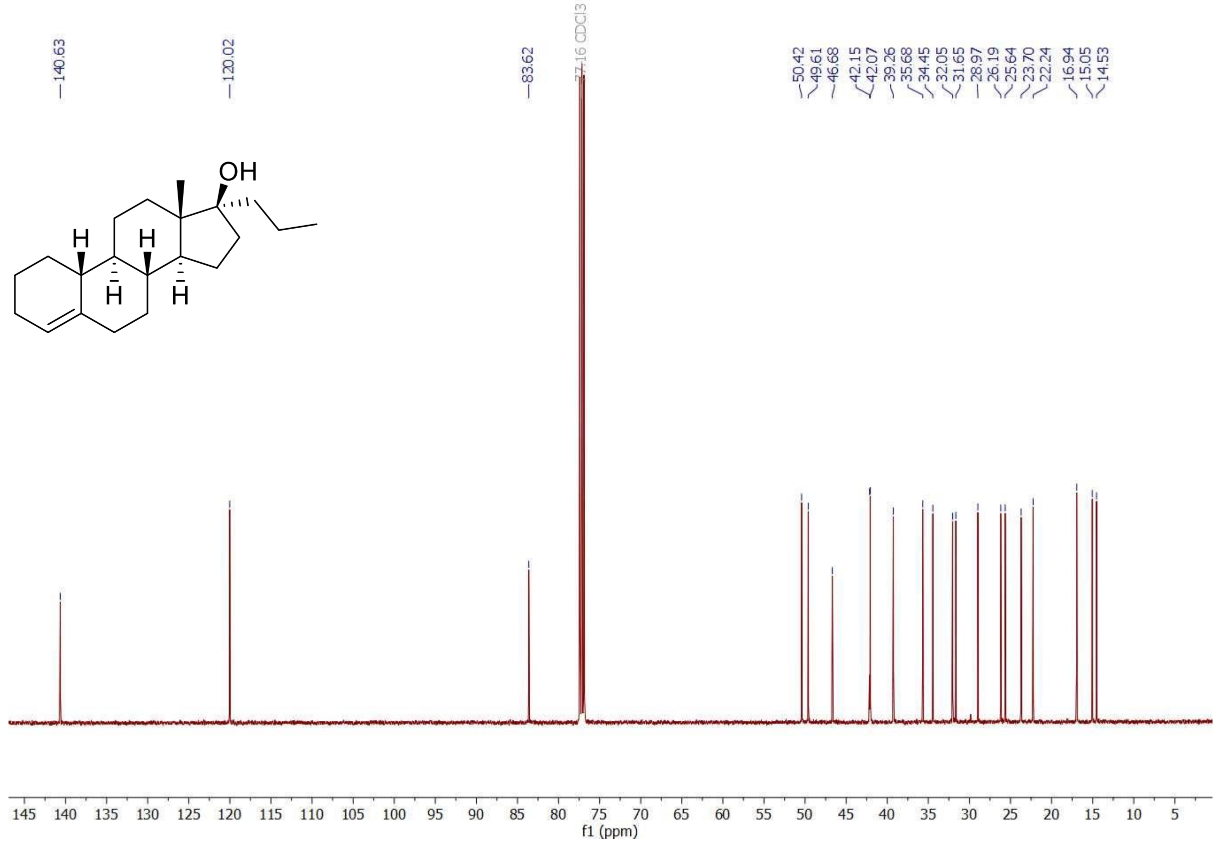

OH
H
H
H
H
140.63
120.02
83.62
77.16 CDCl3
50.42
49.61
46.68
42.15
42.07
39.26
35.68
34.45
32.05
31.65
28.97
26.19
25.64
23.70
22.24
16.94
15.05
14.53
f1 (ppm)

Compound 2a:

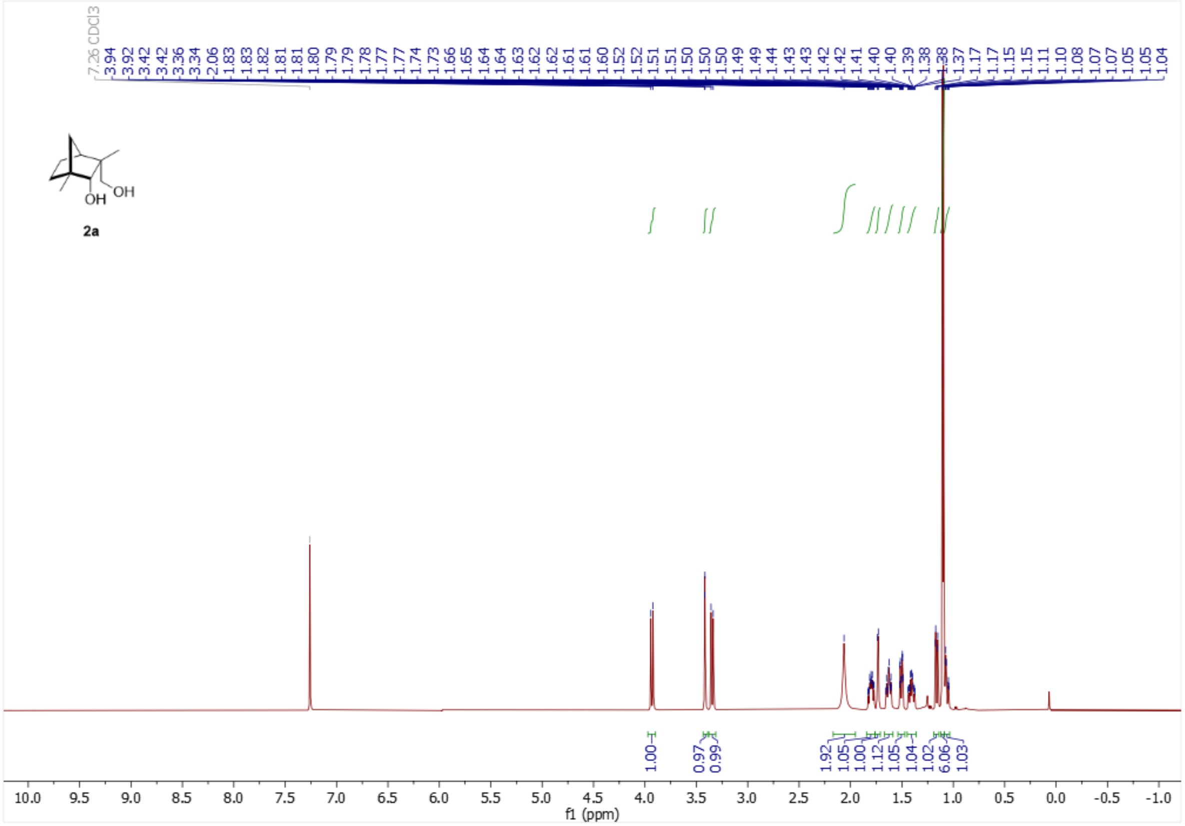
7.26 CDCl3
3.94
3.92
3.42
3.36
3.34
2.06
1.83
1.83
1.82
1.81
1.81
1.80
1.79
1.79
1.78
1.77
1.77
1.74
1.73
1.66
1.65
1.64
1.64
1.63
1.62
1.62
1.61
1.61
1.60
1.52
1.52
1.51
1.51
1.50
1.50
1.50
1.49
1.49
1.44
1.43
1.43
1.42
1.42
1.41
1.40
1.40
1.39
1.38
1.38
1.37
1.17
1.17
1.15
1.15
1.11
1.10
1.08
1.07
1.07
1.05
1.04
OH
OH
2a
1.00
0.97
0.99
1.92
1.05
1.00
1.12
1.05
1.04
1.02
6.06
1.03
10.0 9.5 9.0 8.5 8.0 7.5 7.0 6.5 6.0 5.5 5.0 4.5 4.0 3.5 3.0 2.5 2.0 1.5 1.0 0.5 0.0 -0.5 -1.0
f1 (ppm)

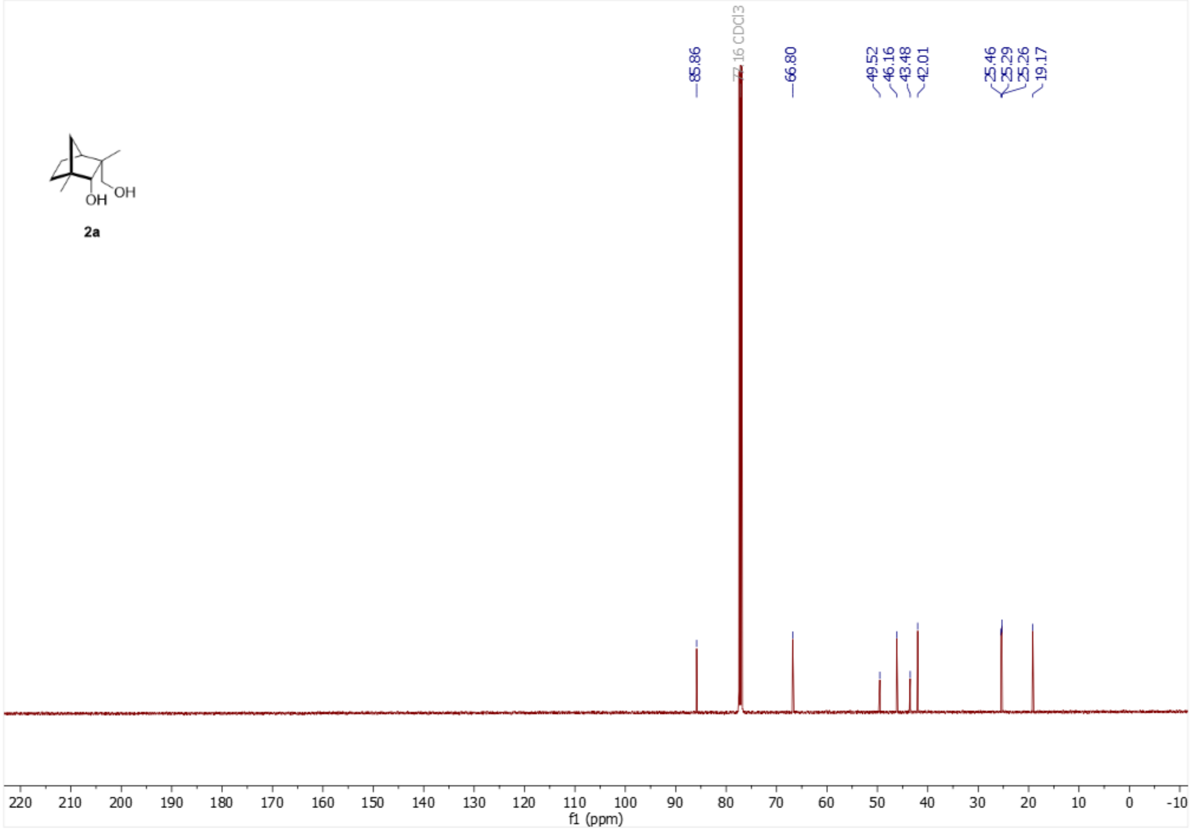
85.86
77.16 CDCl3
66.80
49.52
46.16
43.48
42.01
25.46
25.29
25.26
19.17
OH
OH
2a
220 210 200 190 180 170 160 150 140 130 120 110 100 90 80 70 60 50 40 30 20 10 0 -10
f1 (ppm)

Compound 2b:

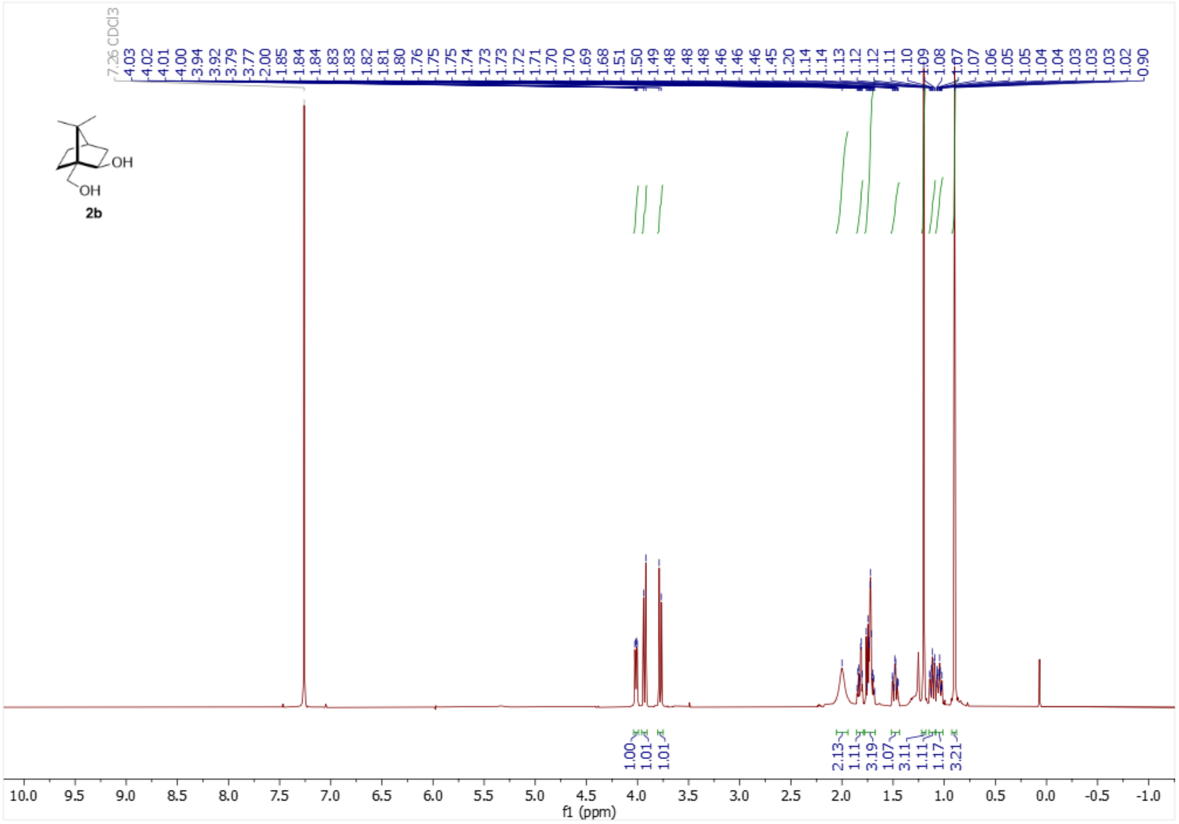
7.26 CDCl3
4.03
4.02
4.01
4.00
3.94
3.92
3.79
3.77
2.00
1.85
1.84
1.84
1.83
1.83
1.82
1.81
1.80
1.76
1.75
1.75
1.74
1.73
1.73
1.72
1.71
1.70
1.70
1.69
1.68
1.51
1.50
1.49
1.48
1.48
1.46
1.46
1.46
1.45
1.20
1.14
1.14
1.13
1.12
1.12
1.11
1.10
1.09
1.08
1.07
1.07
1.06
1.05
1.05
1.04
1.04
1.03
1.03
1.02
0.90
OH
OH
2b
1.00
1.01
1.01
2.13
1.11
3.19
1.07
3.11
1.11
1.17
3.21
f1 (ppm)

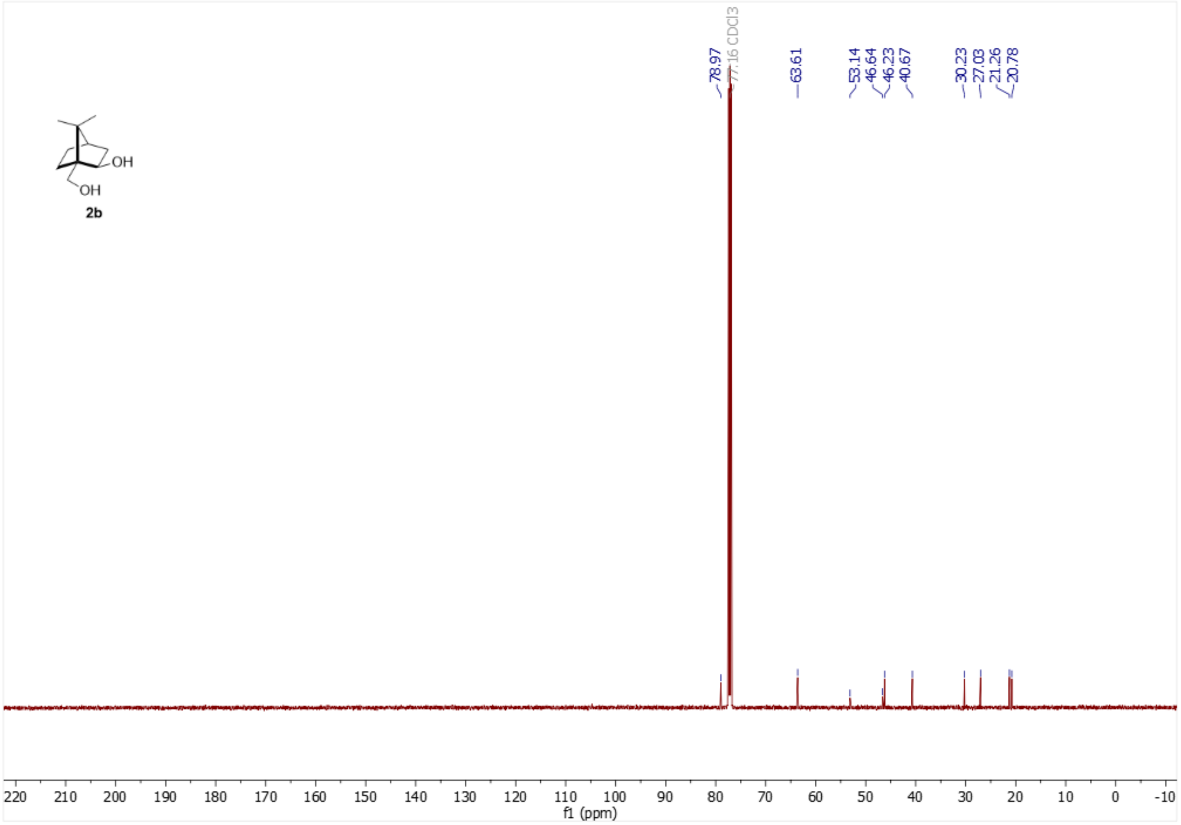
78.97
77.16 CDCl3
63.61
53.14
46.64
46.23
40.67
30.23
27.03
21.26
20.78
OH
OH
2b
f1 (ppm)

Compound 2e:

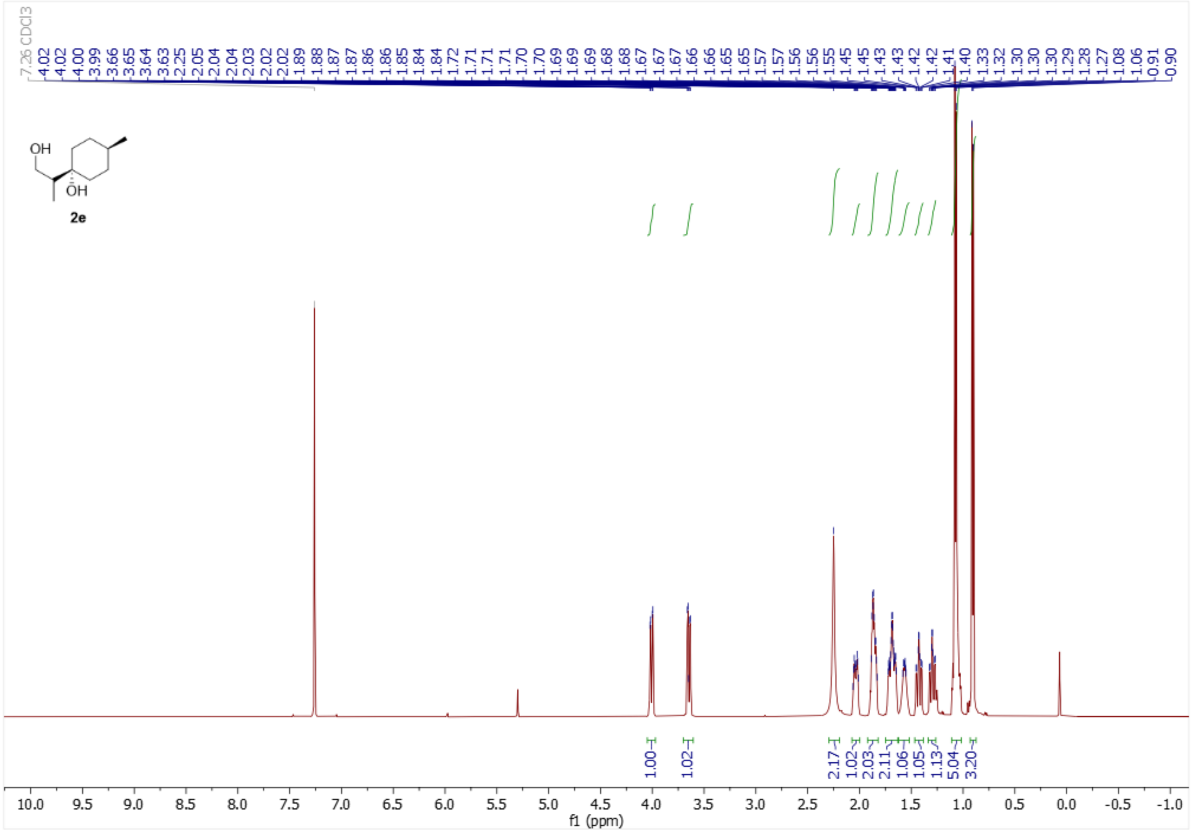

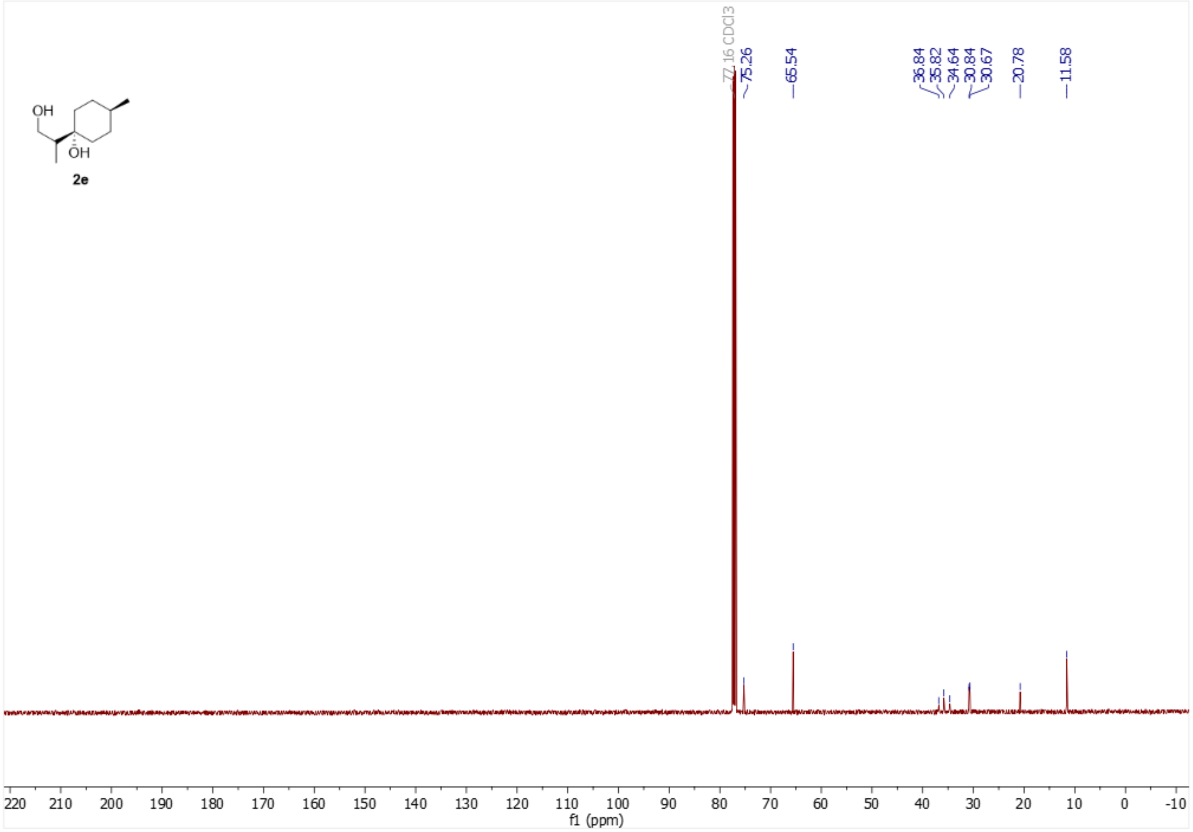

Compound 2f:

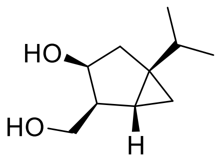

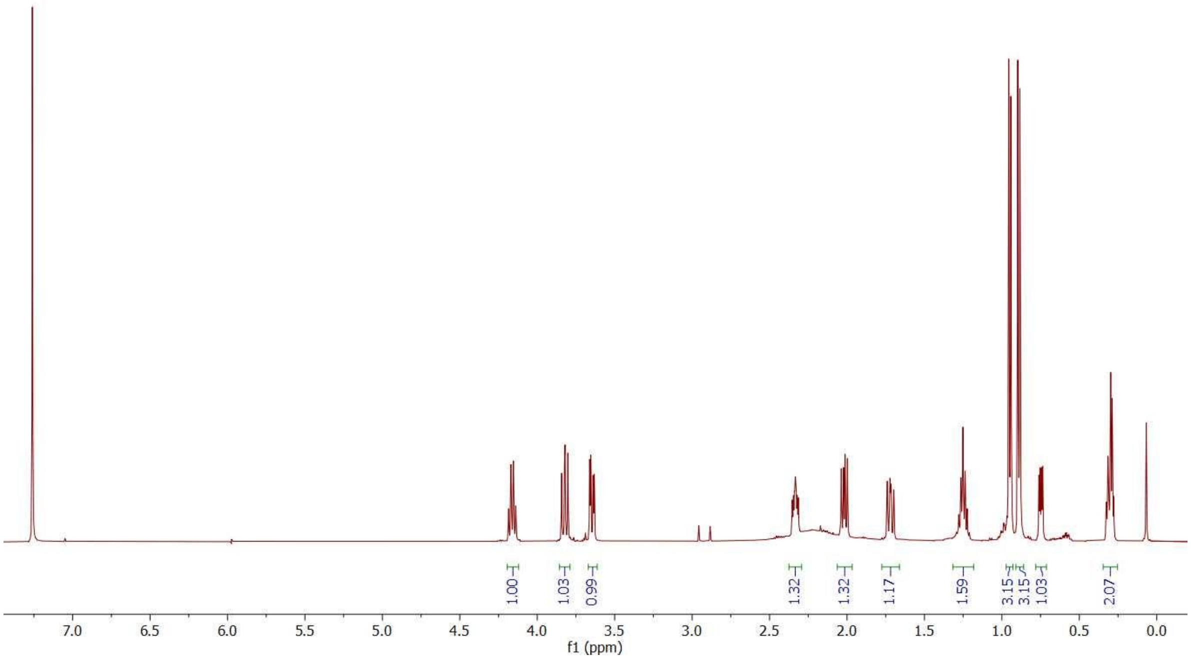
1.00
1.03
0.99
1.32
1.32
1.17
1.59
3.15
3.15
1.03
2.07
7.0
6.5
6.0
5.5
5.0
4.5
4.0
3.5
3.0
2.5
2.0
1.5
1.0
0.5
0.0
f1 (ppm)

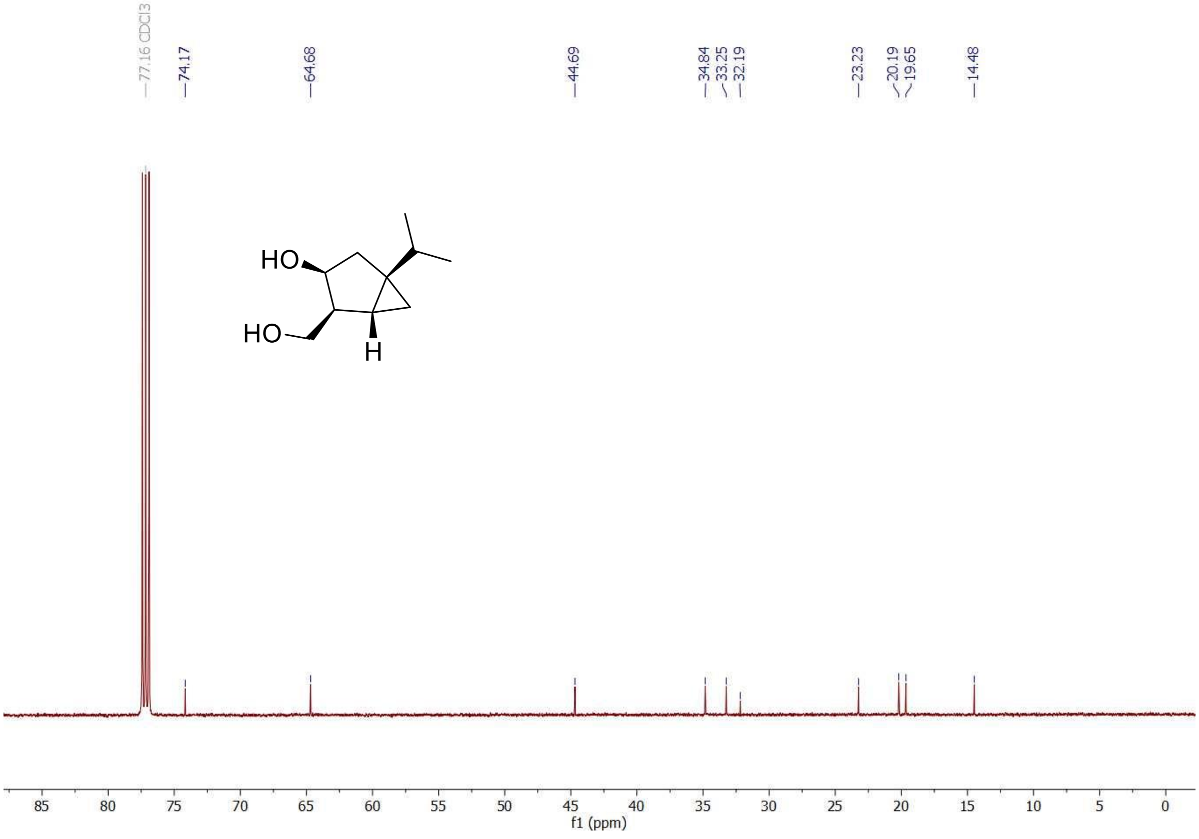
77.16 CDCl3
74.17
64.68
44.69
34.84
33.25
32.19
23.23
20.19
19.65
14.48
85
80
75
70
65
60
55
50
45
40
35
30
25
20
15
10
5
0
f1 (ppm)

Compound 2g:

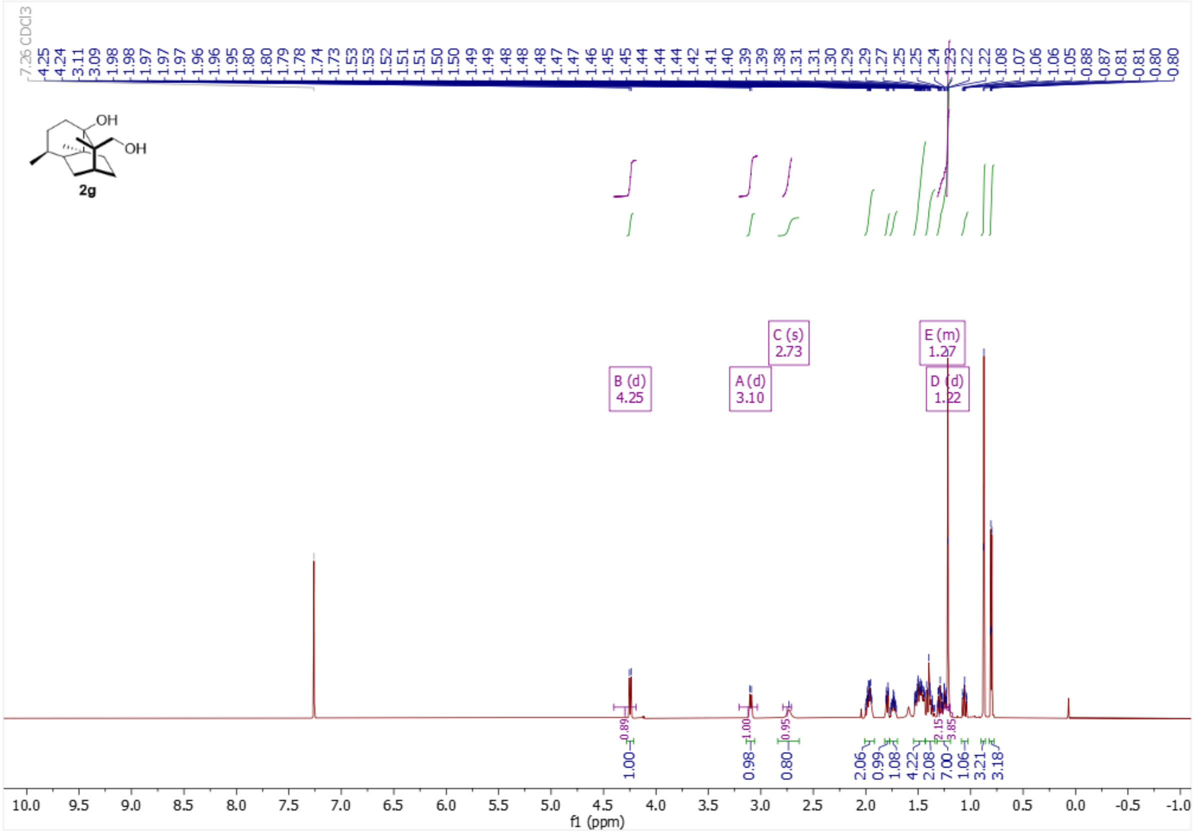

2g
B (d) 4.25
A (d) 3.10
C (s) 2.73
E (m) 1.27
D (d) 1.22
f1 (ppm)

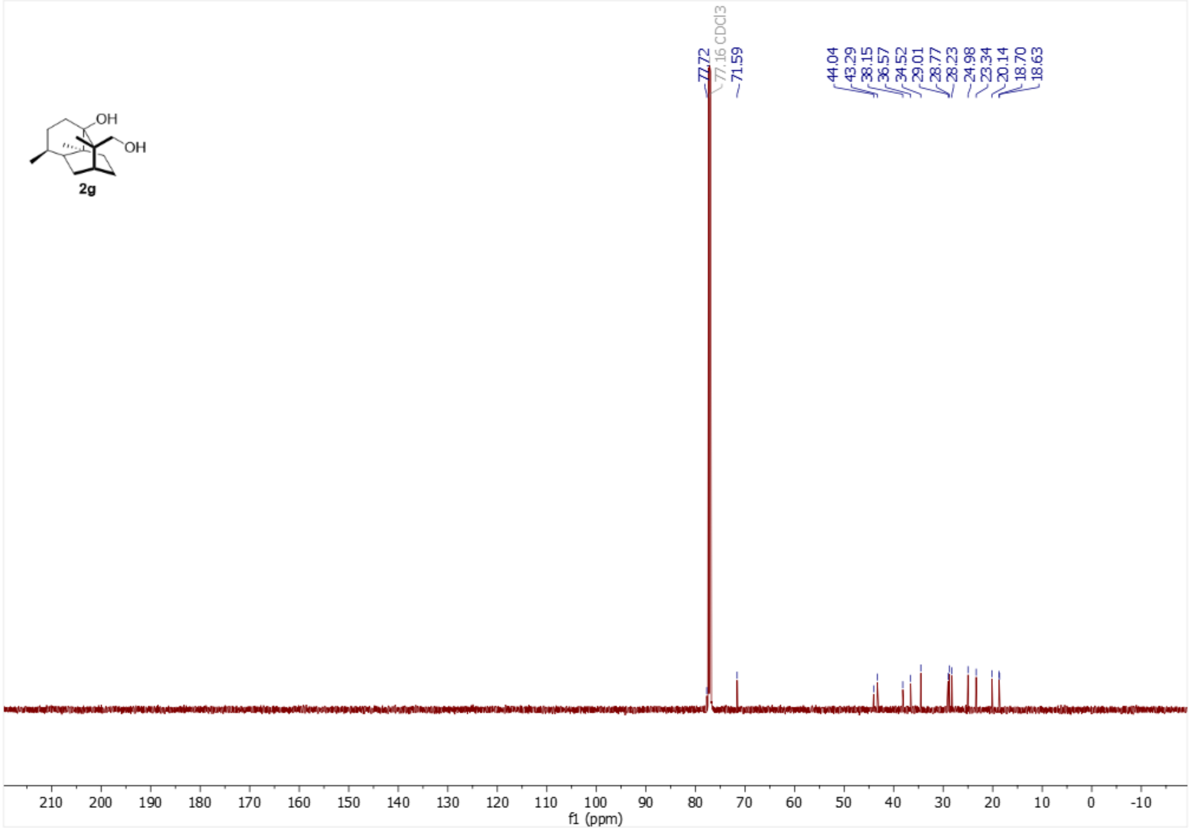

2g
77.72
77.16 CDCl3
71.59
44.04
43.29
38.15
36.57
34.52
29.01
28.77
28.23
24.98
23.34
20.14
18.70
18.63
f1 (ppm)

Compound 2h:

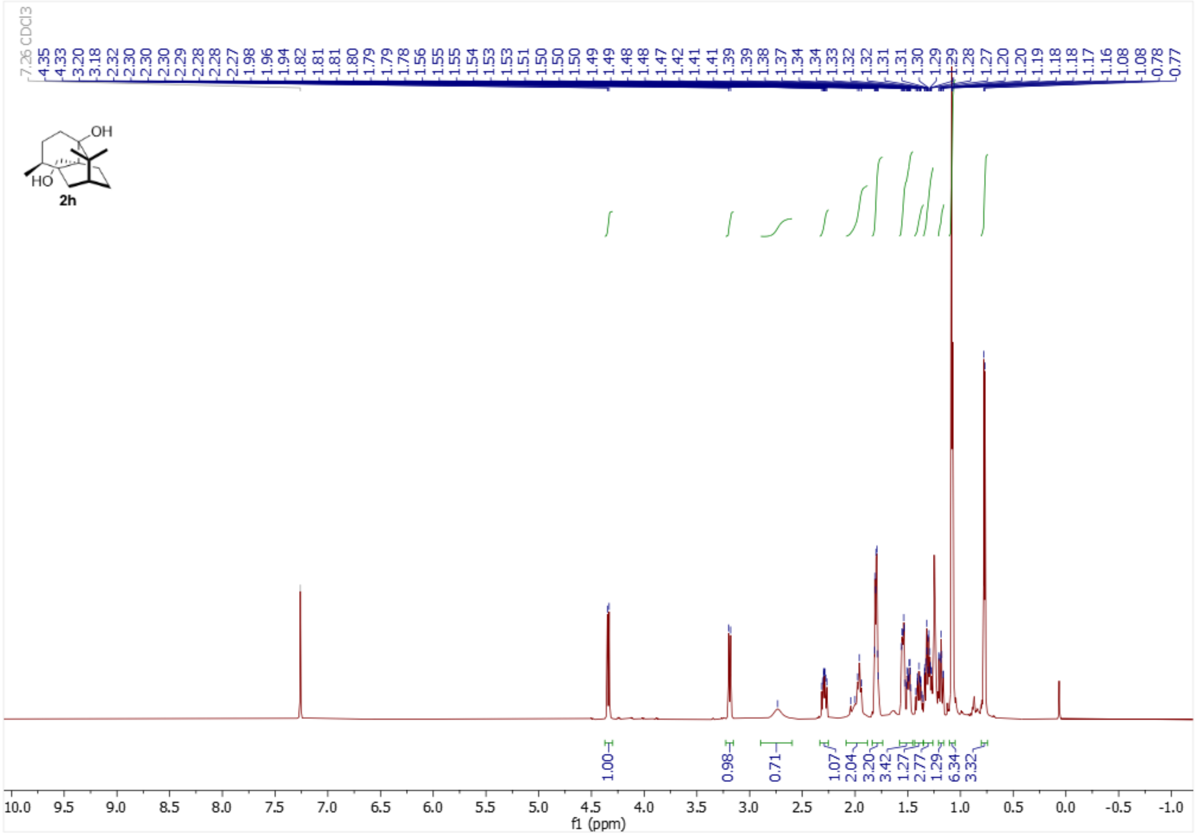
7.26 CDCl3
4.35
4.33
3.20
3.18
2.32
2.30
2.30
2.30
2.29
2.28
2.28
2.27
1.98
1.96
1.94
1.82
1.81
1.81
1.80
1.79
1.79
1.78
1.56
1.55
1.55
1.54
1.53
1.53
1.51
1.50
1.50
1.50
1.49
1.49
1.48
1.48
1.47
1.42
1.41
1.41
1.39
1.39
1.39
1.38
1.37
1.34
1.34
1.33
1.32
1.32
1.32
1.31
1.31
1.30
1.29
1.29
1.28
1.27
1.20
1.20
1.19
1.18
1.18
1.17
1.16
1.08
0.78
0.77
OH
HO
2h
1.00
0.98
0.71
1.07
2.04
3.20
3.42
1.27
2.77
1.29
6.34
3.32
10.0
9.5
9.0
8.5
8.0
7.5
7.0
6.5
6.0
5.5
5.0
4.5
4.0
3.5
3.0
2.5
2.0
1.5
1.0
0.5
0.0
-0.5
-1.0
f1 (ppm)

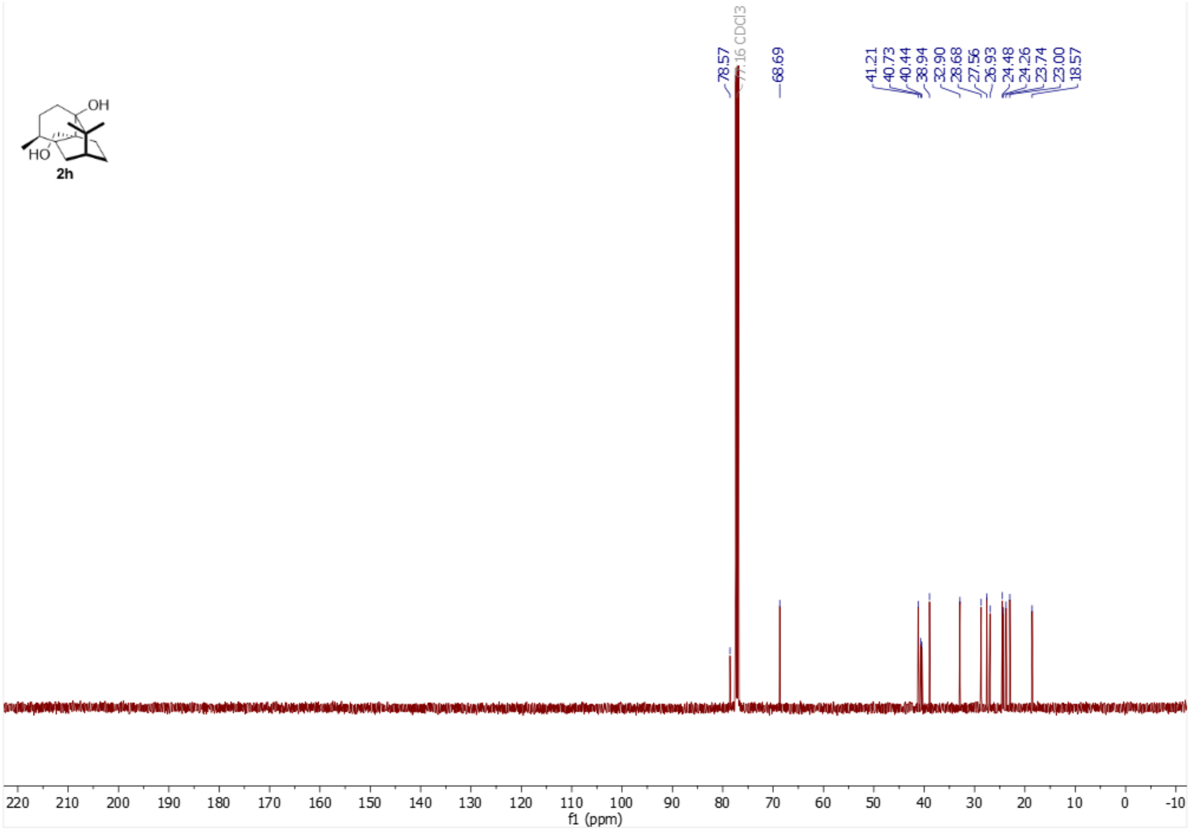
78.57
77.16 CDCl3
68.69
41.21
40.73
40.44
38.94
32.90
28.68
27.56
26.93
24.48
24.26
23.74
23.00
18.57
OH
HO
2h
220
210
200
190
180
170
160
150
140
130
120
110
100
90
80
70
60
50
40
30
20
10
0
-10
f1 (ppm)

Compound 2i:

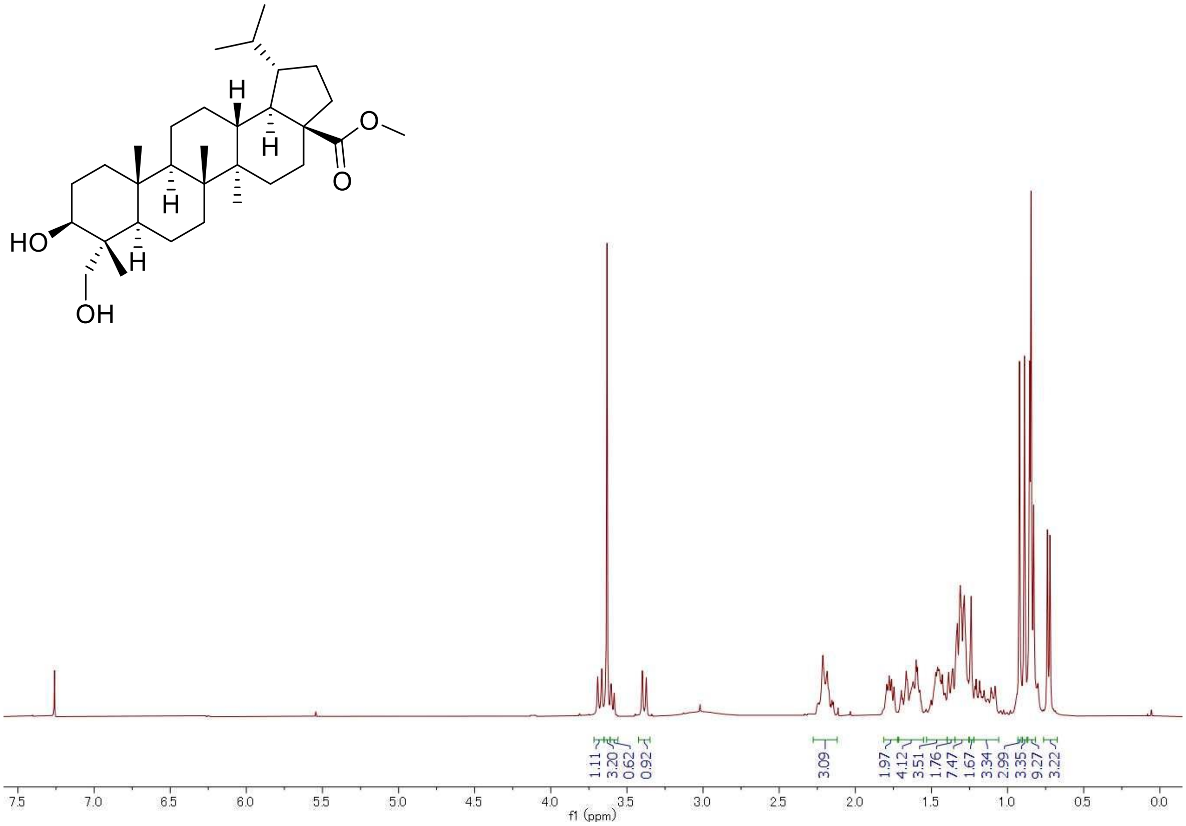

HO
OH
H
O
1.11
3.20
0.62
0.92
3.09
1.97
4.12
3.51
1.76
7.47
1.67
3.34
2.99
3.35
9.27
3.22
f1 (ppm)

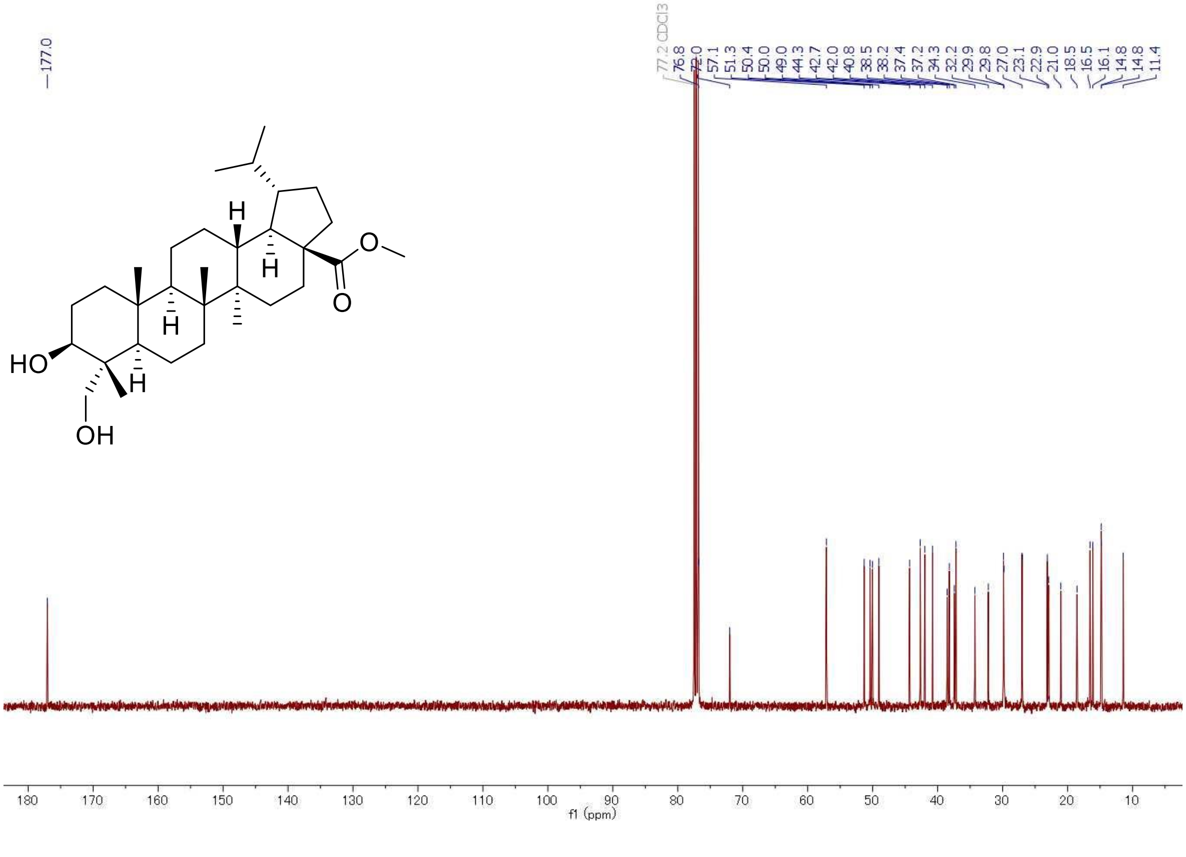

177.0
77.2 CDCl3
76.8
72.0
57.1
51.3
50.4
50.0
49.0
44.3
42.7
42.0
40.8
38.5
38.2
37.4
37.2
34.3
32.2
29.9
29.8
27.0
23.1
22.9
21.0
18.5
16.5
16.1
14.8
14.8
11.4
HO
OH
H
O
f1 (ppm)

Compound 2j:

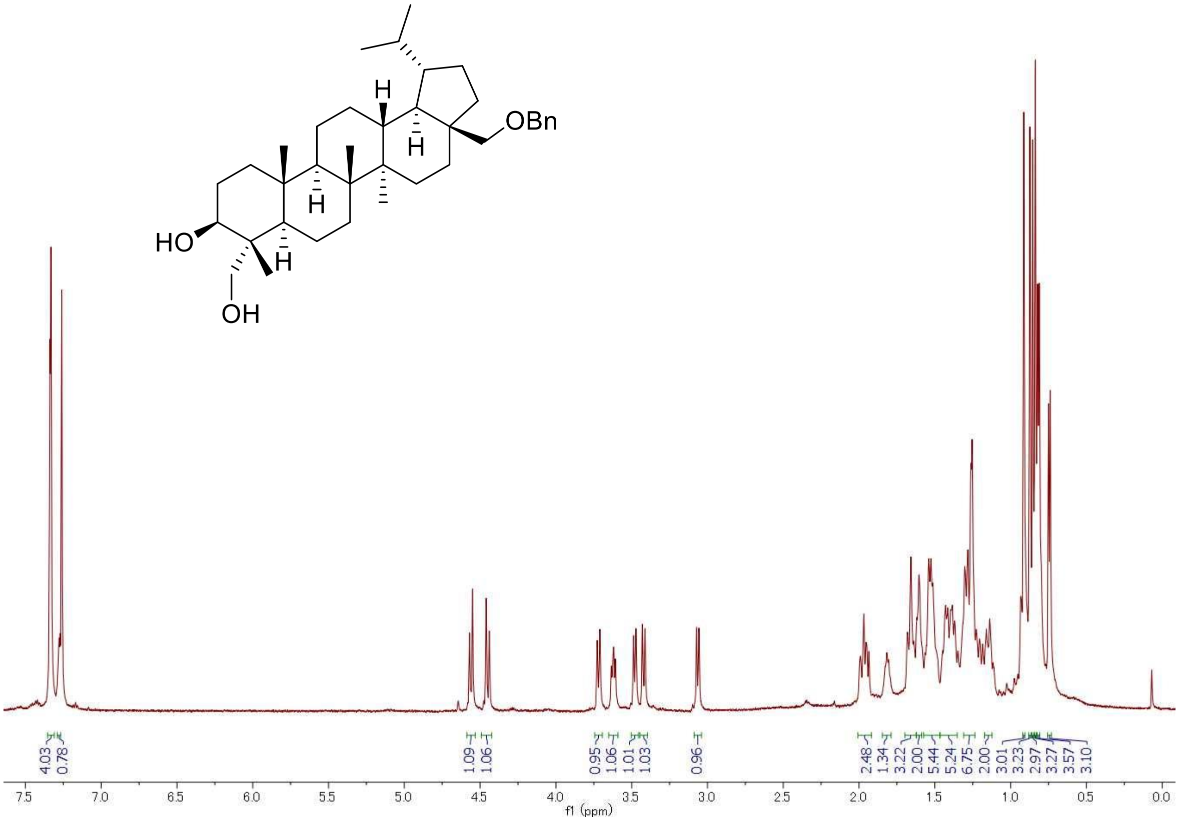

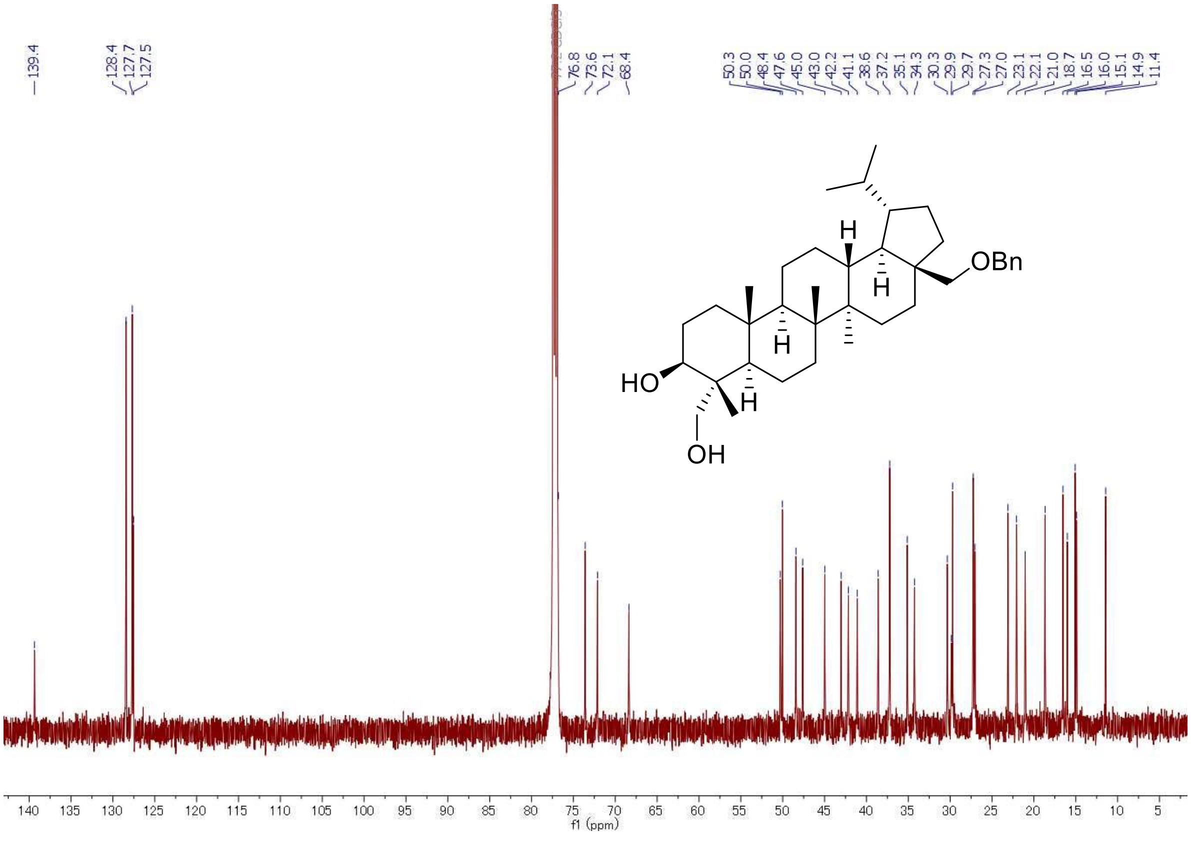

Compound 2k:

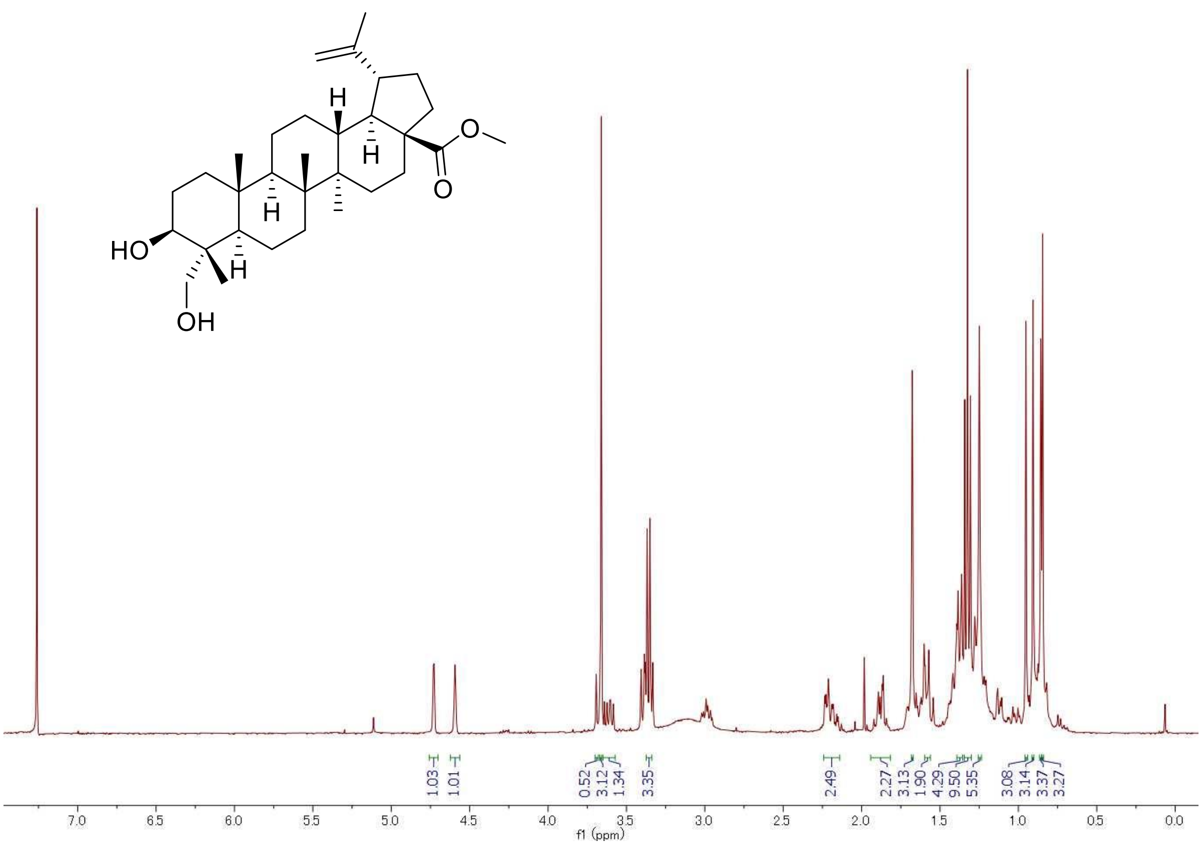

HO
OH
H
H
H
H
O
O
1.03
1.01
0.52
3.12
1.34
3.35
2.49
2.27
3.13
1.90
4.29
9.50
5.35
3.08
3.14
3.37
3.27
7.0 6.5 6.0 5.5 5.0 4.5 4.0 3.5 3.0 2.5 2.0 1.5 1.0 0.5 0.0
f1 (ppm)

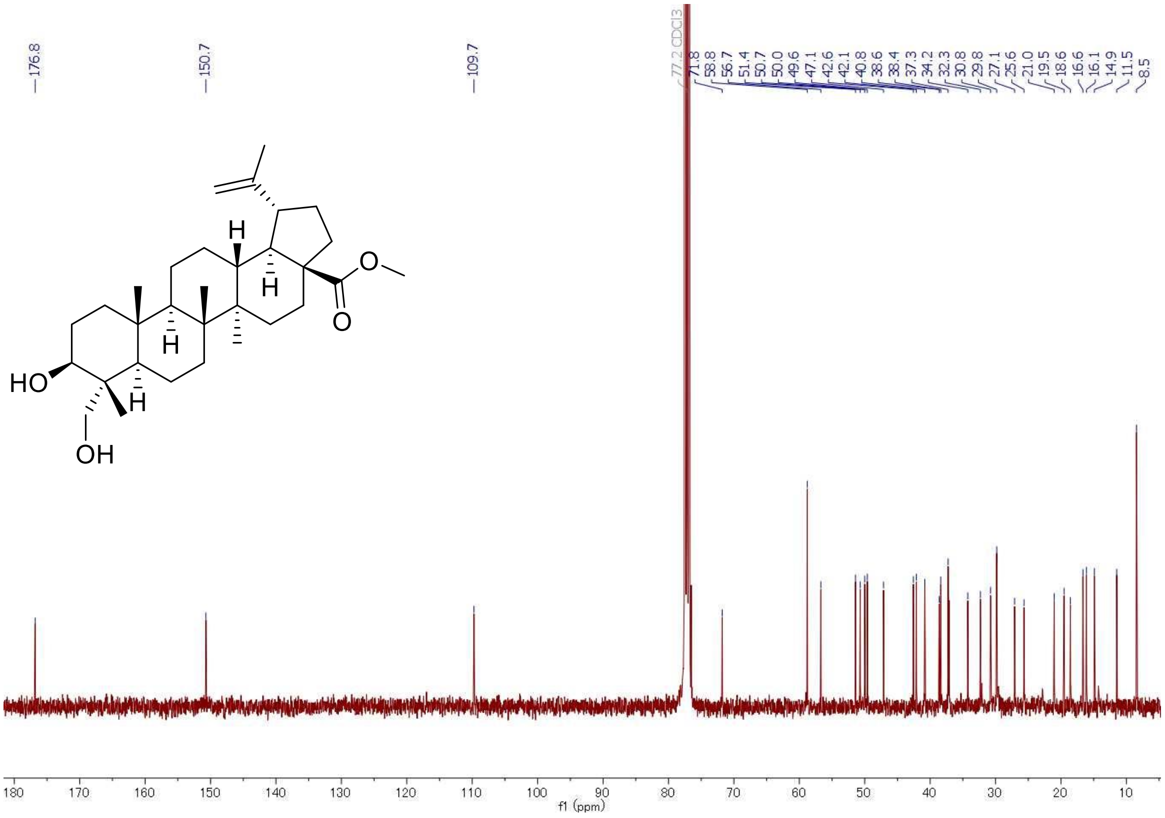

176.8
150.7
109.7
77.2 CDCl3
71.8
58.8
56.7
51.4
50.7
50.0
49.6
47.1
42.6
42.1
40.8
38.6
38.4
37.3
34.2
32.3
30.8
29.8
27.1
25.6
21.0
19.5
18.6
16.6
16.1
14.9
11.5
8.5
HO
OH
H
H
H
H
O
O
180 170 160 150 140 130 120 110 100 90 80 70 60 50 40 30 20 10
f1 (ppm)

Compound 2I:

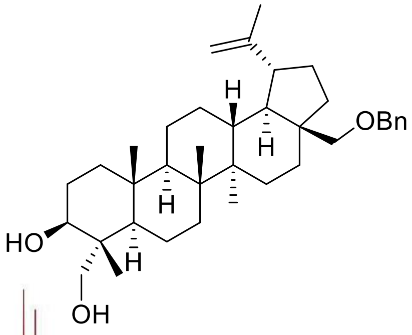

OBn
HO
OH
H

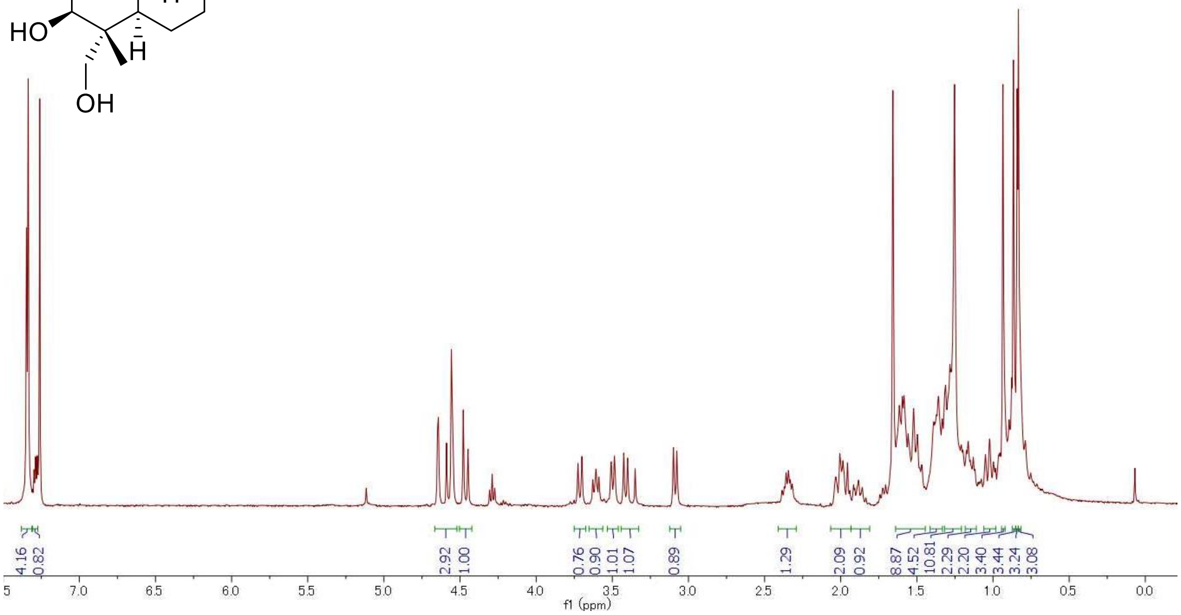

4.16
0.82
2.92
1.00
0.76
0.90
1.01
1.07
0.89
1.29
2.09
0.92
8.87
4.52
10.81
2.29
2.20
3.40
3.44
3.24
3.08
f1 (ppm)

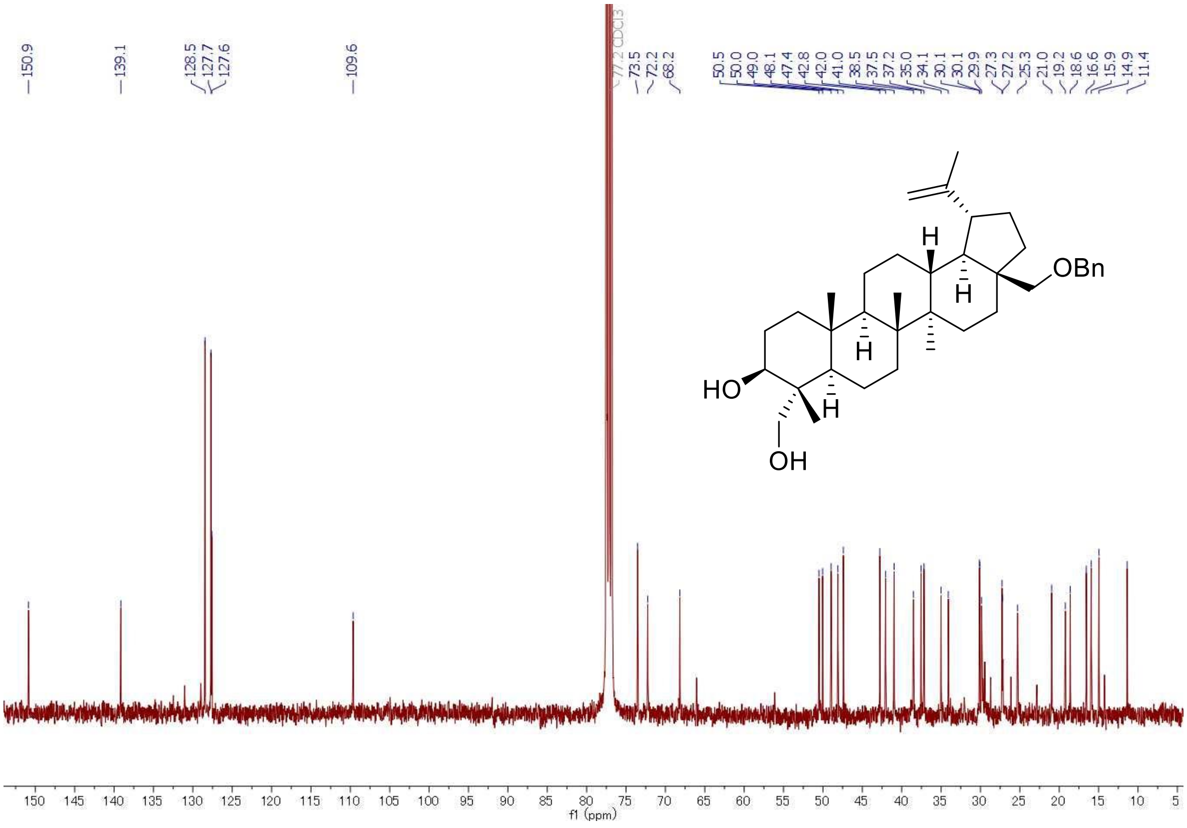

150.9
139.1
128.5
127.7
127.6
109.6
77.2 CDCl3
73.5
72.2
68.2
50.5
50.0
49.0
48.1
47.4
42.8
42.0
41.0
38.5
37.5
37.2
35.0
34.1
30.1
30.1
29.9
27.3
27.2
25.3
21.0
19.2
18.6
16.6
15.9
14.9
11.4
OBn
HO
OH
H
f1 (ppm)

Compound 2m and 2n:

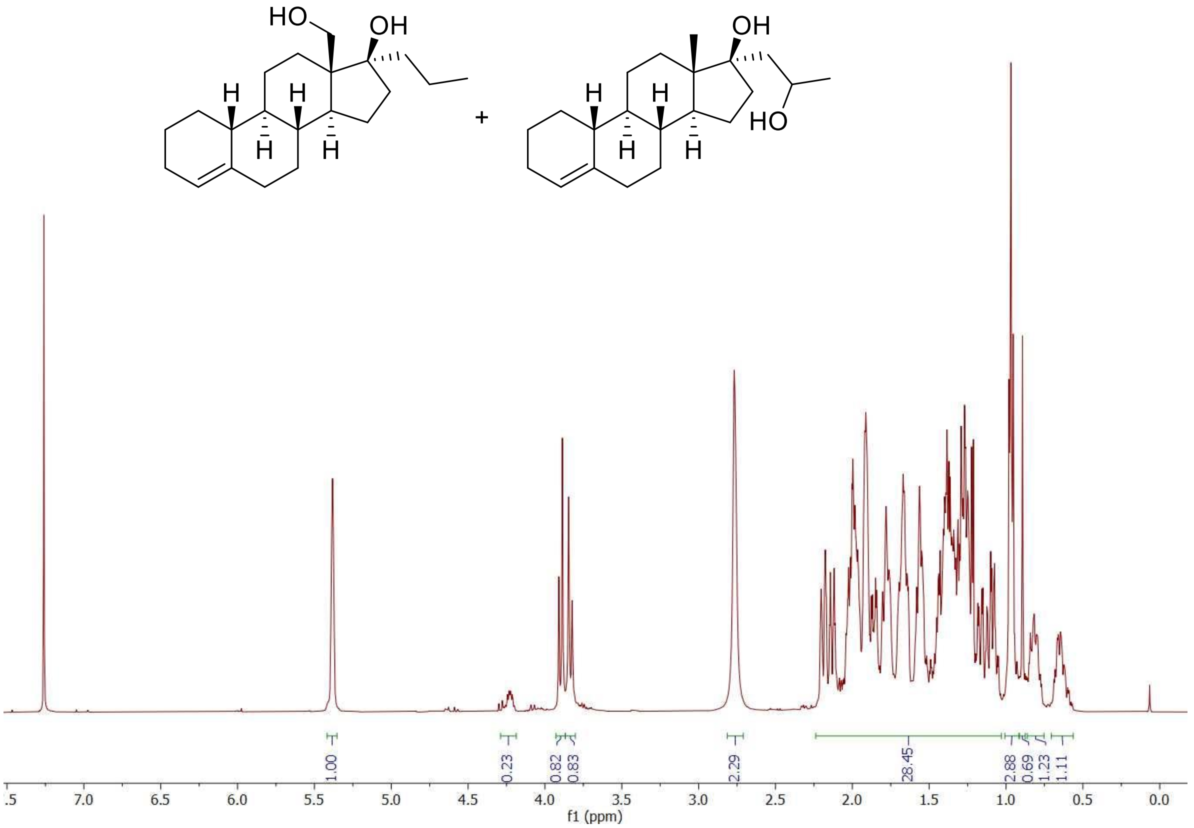

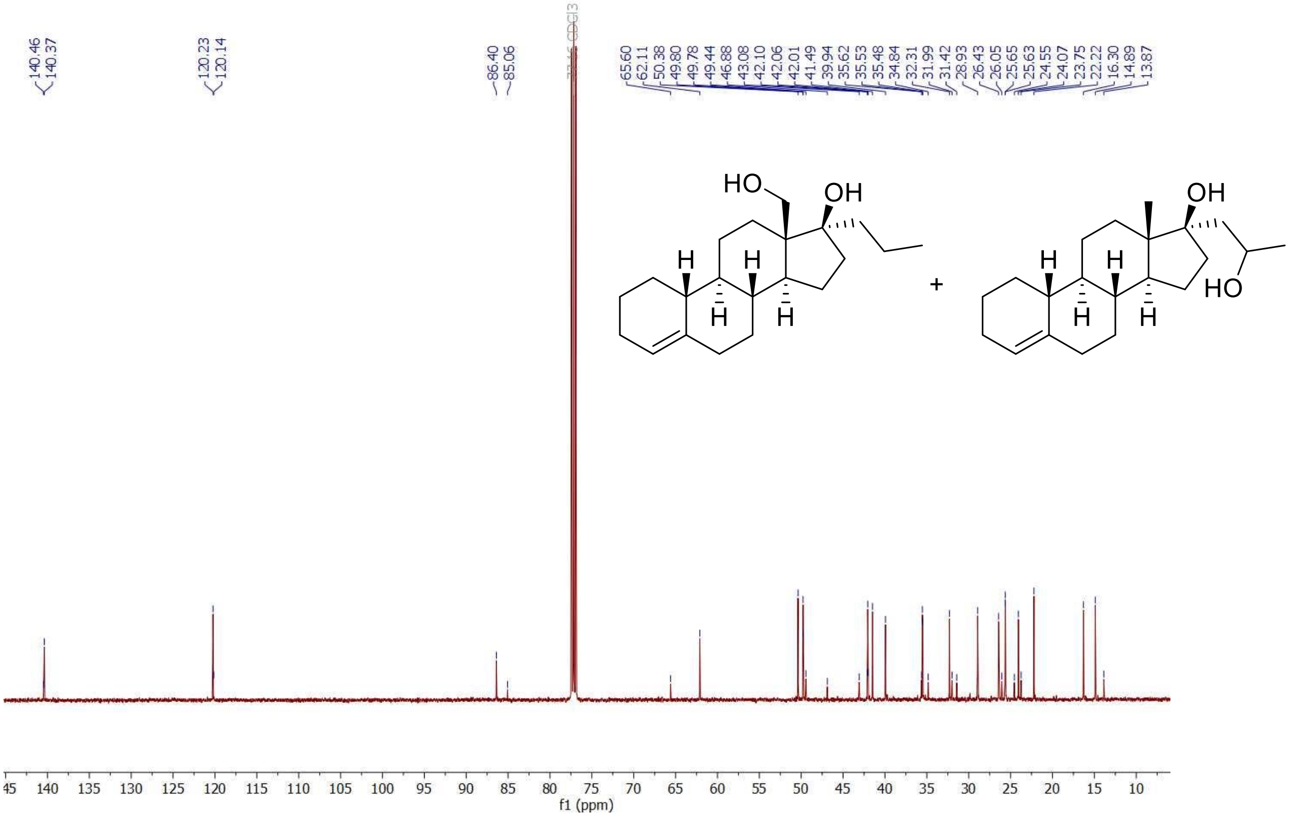

Compound 2o:

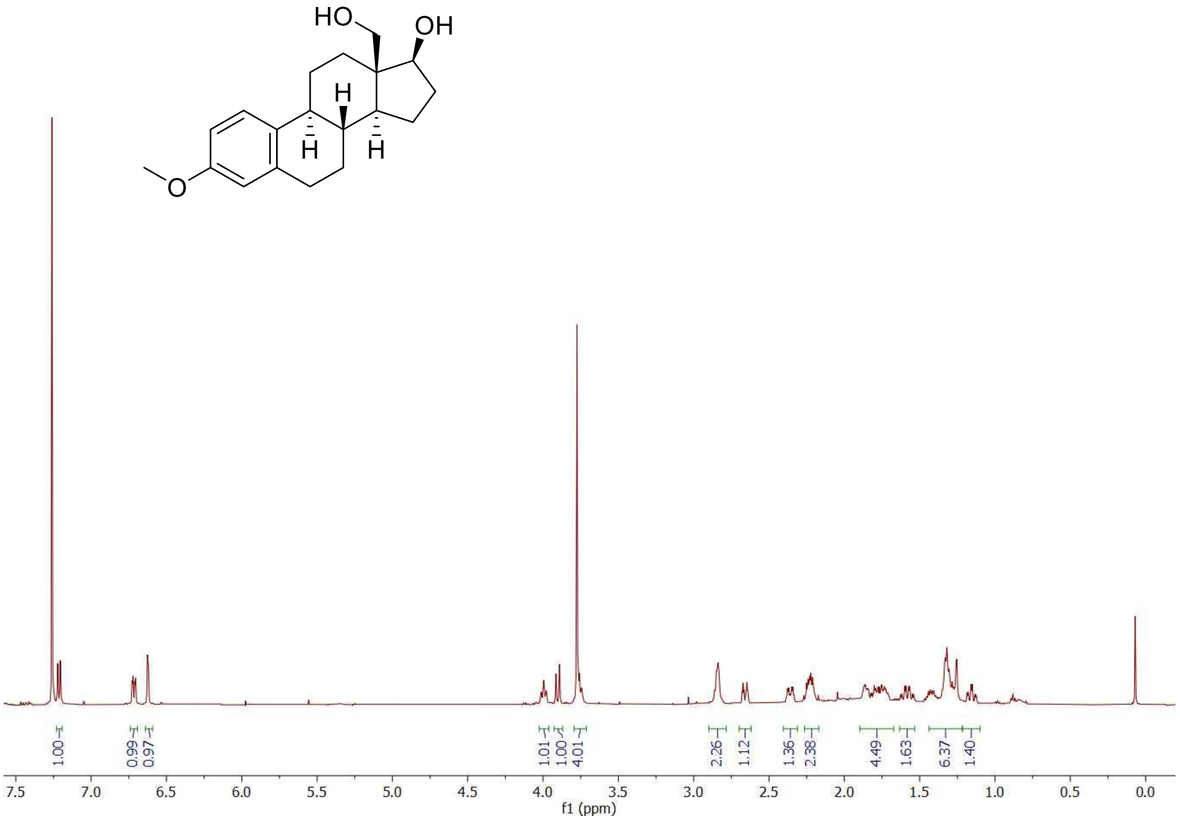
HO
OH
H
H
H
O
1.00
0.99
0.97
1.01
1.00
4.01
2.26
1.12
1.36
2.38
4.49
1.63
6.37
1.40
7.5 7.0 6.5 6.0 5.5 5.0 4.5 4.0 3.5 3.0 2.5 2.0 1.5 1.0 0.5 0.0
f1 (ppm)

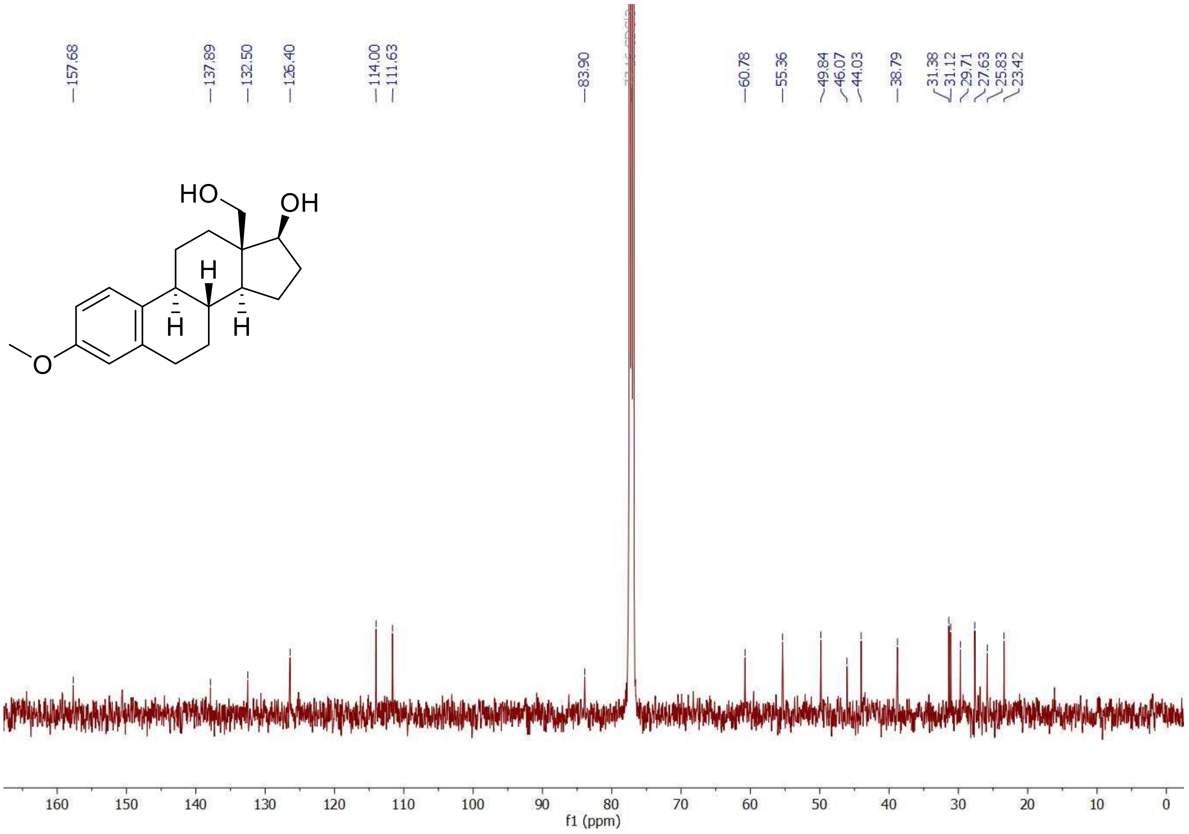
157.68
137.89
132.50
126.40
114.00
111.63
83.90
60.78
55.36
49.84
46.07
44.03
38.79
31.38
31.12
29.71
27.63
25.83
23.42
HO
OH
H
H
H
O
160 150 140 130 120 110 100 90 80 70 60 50 40 30 20 10 0
f1 (ppm)

Compound 2p:

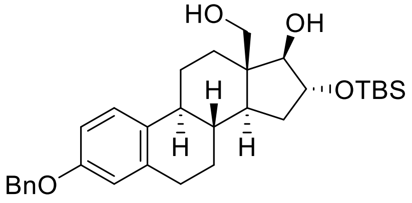
HO
OH
OTBS
H
H
H
BnO

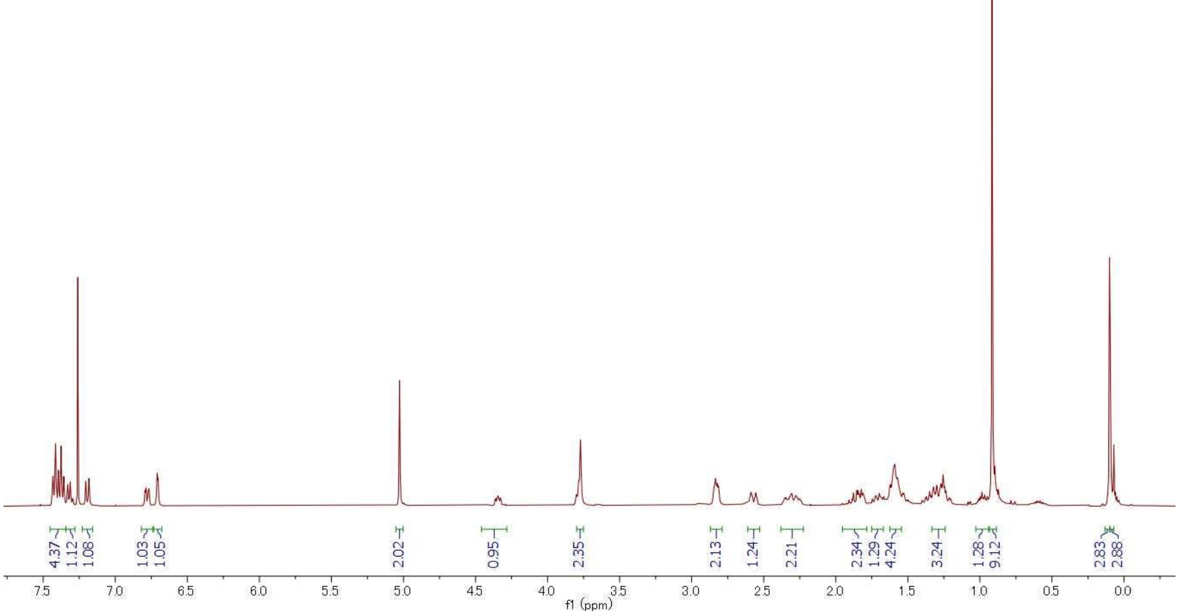
4.37
1.12
1.08
1.03
1.05
2.02
0.95
2.35
2.13
1.24
2.21
2.34
1.29
4.24
3.24
1.28
9.12
2.83
2.88
7.5 7.0 6.5 6.0 5.5 5.0 4.5 4.0 3.5 3.0 2.5 2.0 1.5 1.0 0.5 0.0
f1 (ppm)

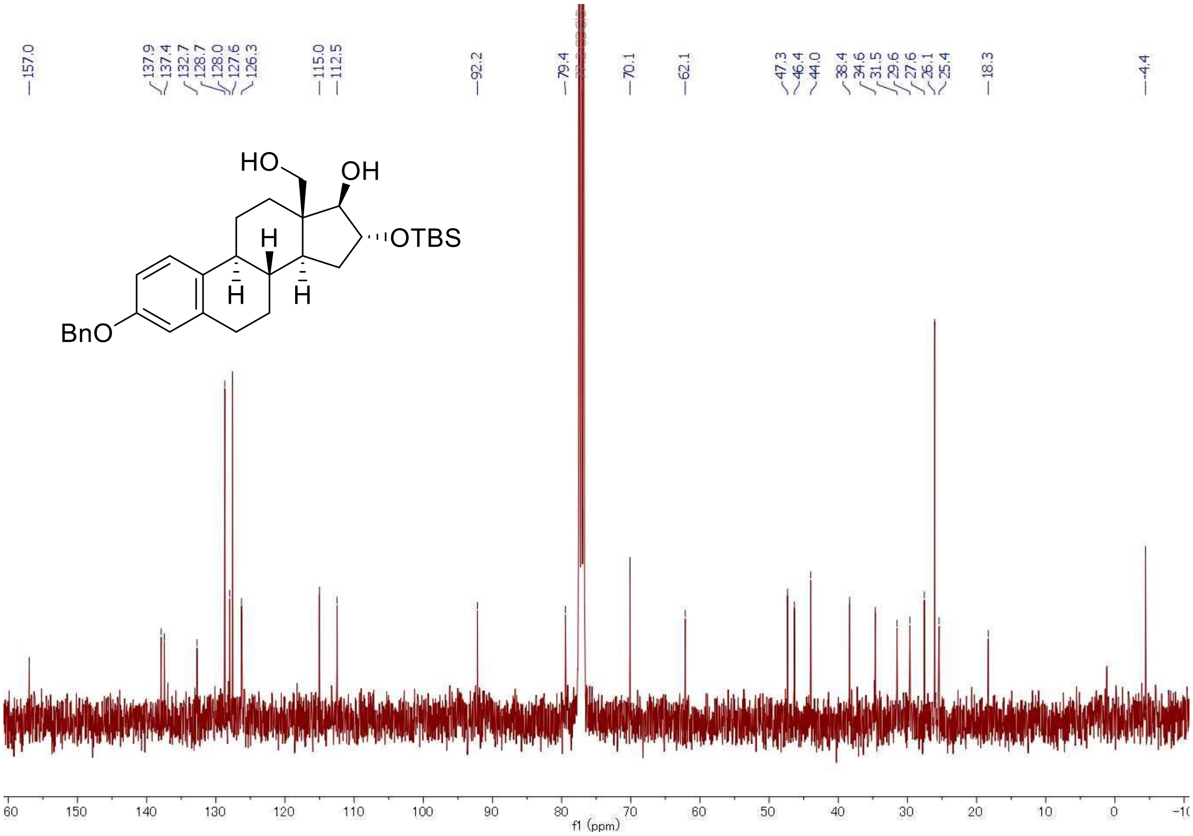
157.0
137.9
137.4
132.7
128.7
128.0
127.6
126.3
115.0
112.5
92.2
79.4
70.1
62.1
47.3
46.4
44.0
38.4
34.6
31.5
29.6
27.6
26.1
25.4
18.3
-4.4
HO
OH
OTBS
H
H
H
BnO
150 140 130 120 110 100 90 80 70 60 50 40 30 20 10 0
f1 (ppm)

Compound 2q:

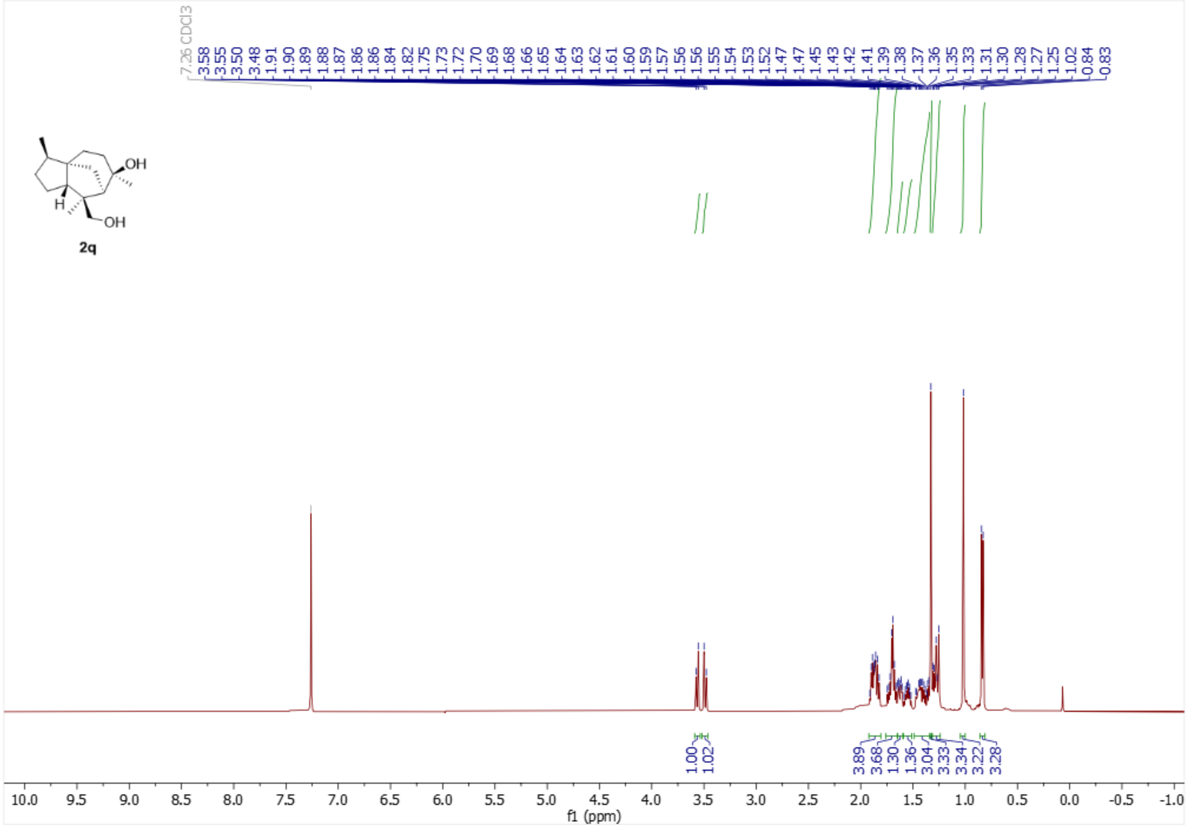

7.26 CDCl3
3.58
3.55
3.50
3.48
1.91
1.90
1.89
1.88
1.87
1.86
1.86
1.84
1.82
1.75
1.73
1.72
1.70
1.69
1.68
1.66
1.65
1.64
1.63
1.62
1.61
1.60
1.59
1.57
1.56
1.56
1.55
1.54
1.53
1.52
1.47
1.47
1.45
1.43
1.42
1.41
1.39
1.38
1.37
1.36
1.35
1.33
1.31
1.30
1.28
1.27
1.25
1.02
0.84
0.83
OH
H
OH
2q
1.00
1.02
3.89
3.68
1.30
1.36
3.04
3.33
3.34
3.22
3.28
f1 (ppm)

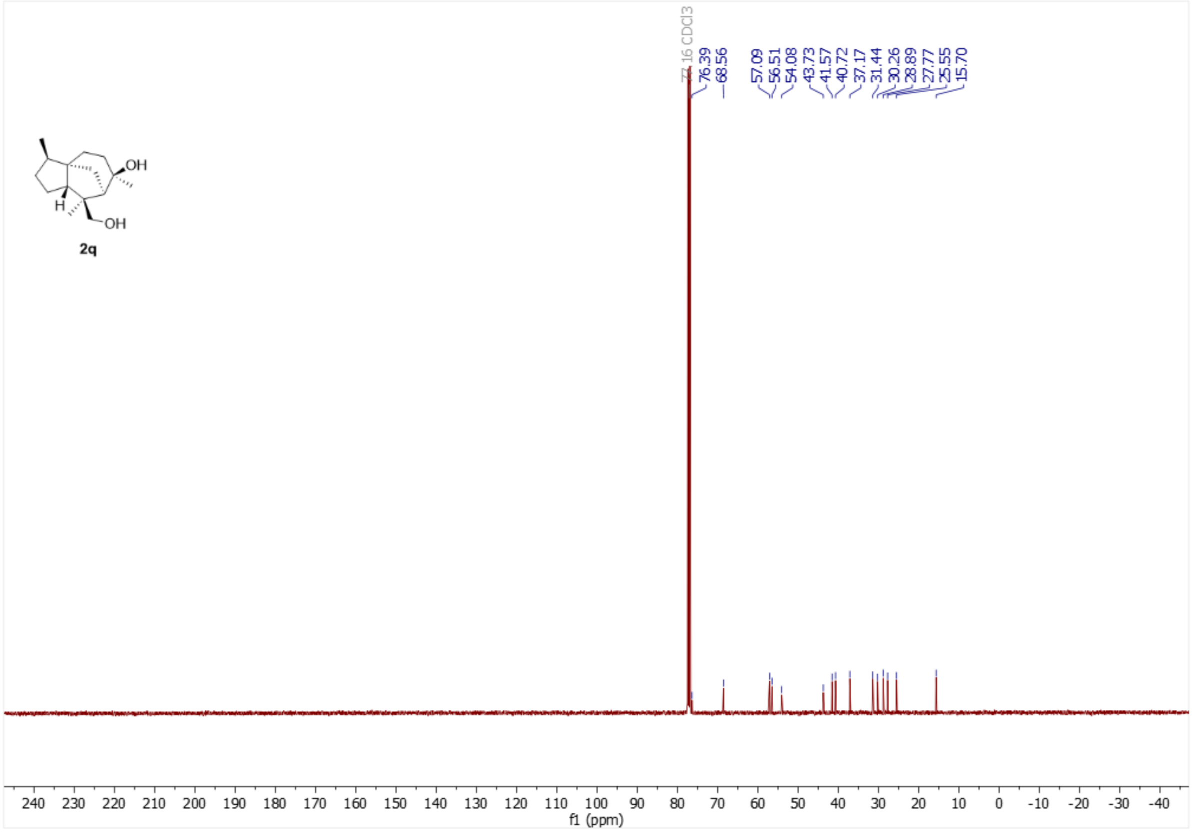

77.16 CDCl3
76.39
68.56
57.09
56.51
54.08
43.73
41.57
40.72
37.17
31.44
30.26
28.89
27.77
25.55
15.70
OH
H
OH
2q
f1 (ppm)

Compound 3a:

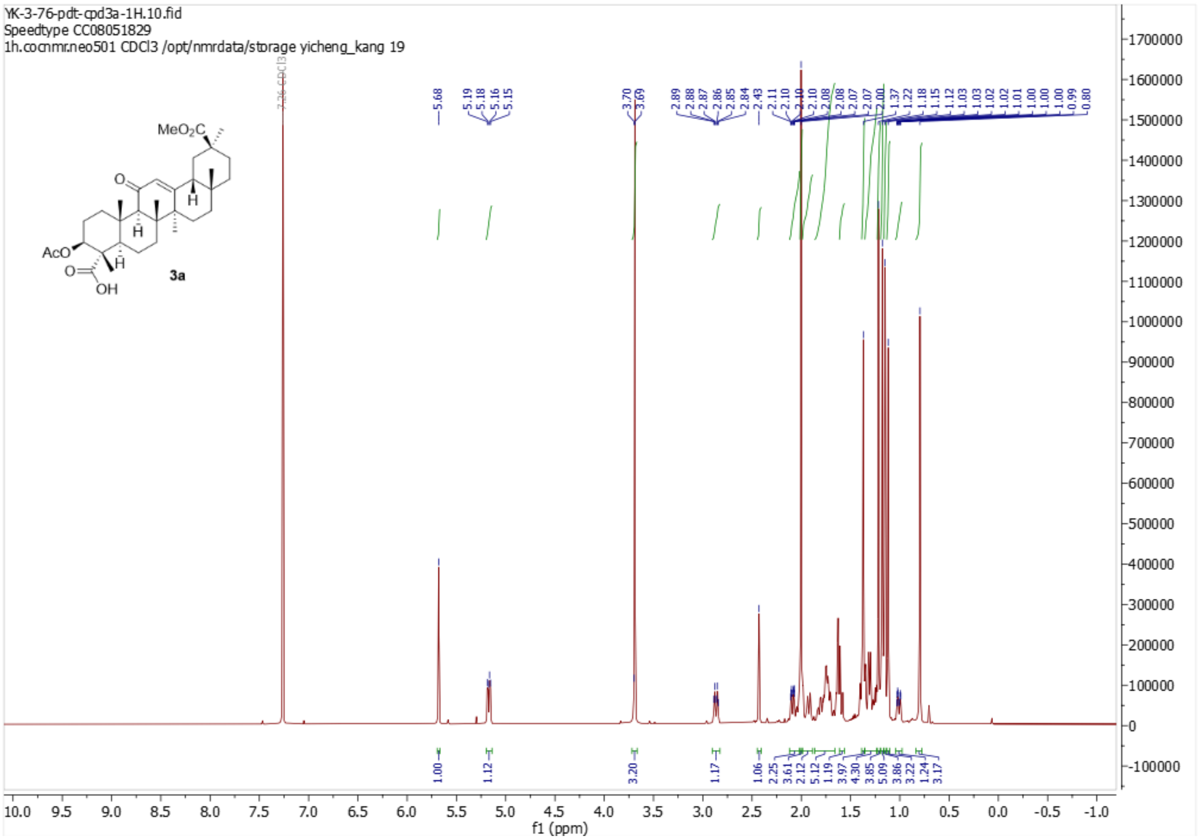

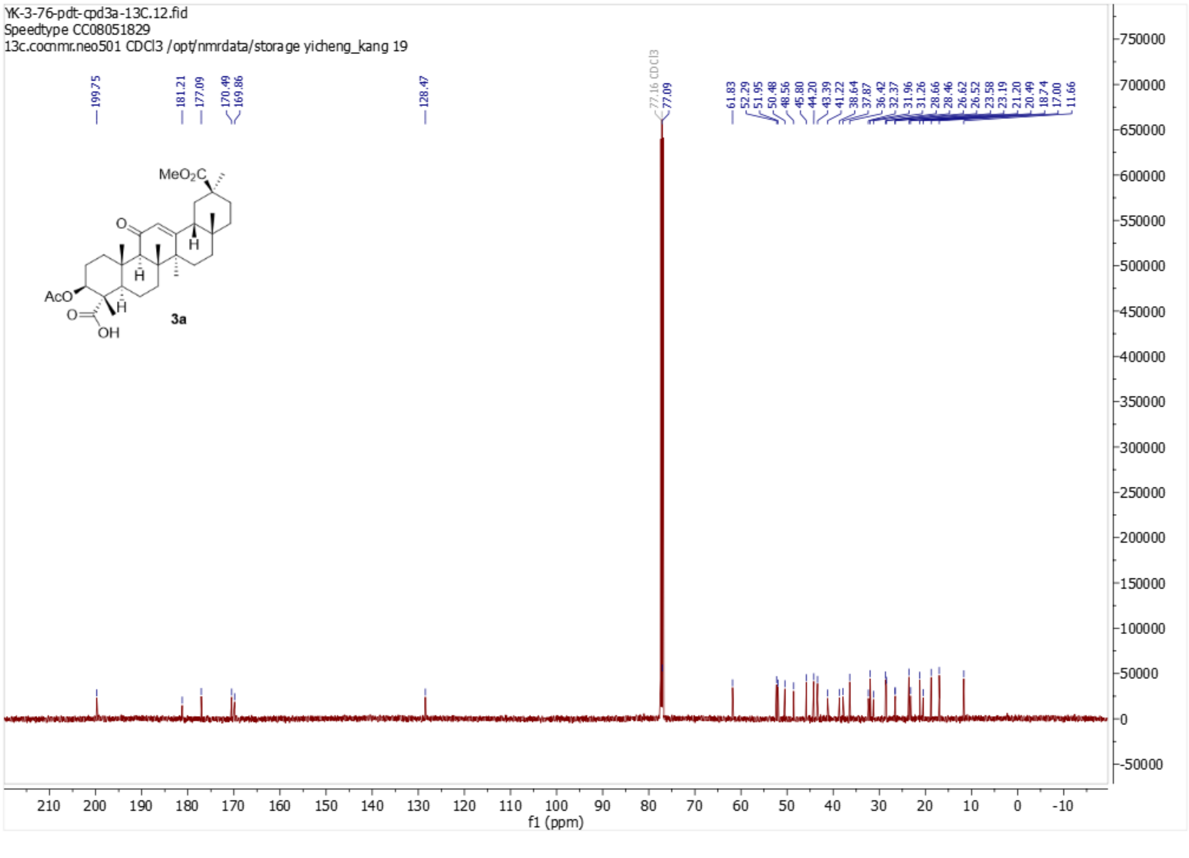

Compound 3b:

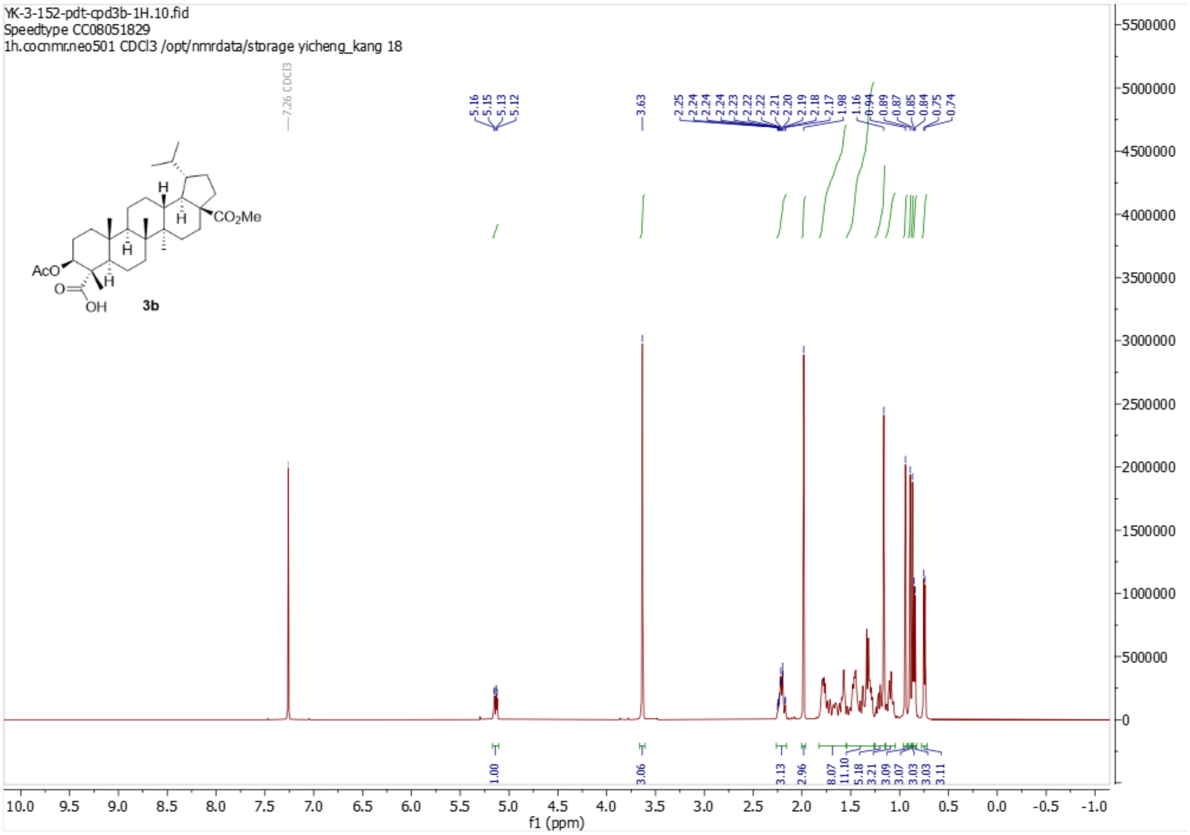
YK-3-152-pdt-cpd3b-1H.10.fid
Speedtype CC08051829
1h.cocnmr.neo501 CDCl3 /opt/nmrdata/storage yicheng_kang 18
7.26 CDCl3
5.16
5.15
5.13
5.12
3.63
2.25
2.24
2.24
2.24
2.23
2.22
2.22
2.21
2.20
2.19
2.18
2.17
1.98
1.16
0.94
0.89
0.87
0.85
0.84
0.75
0.74
1.00
3.06
3.13
2.96
8.07
11.10
5.18
3.21
3.09
3.07
3.03
3.03
3.11
AcO
O
OH
H
CO2Me
3b
f1 (ppm)

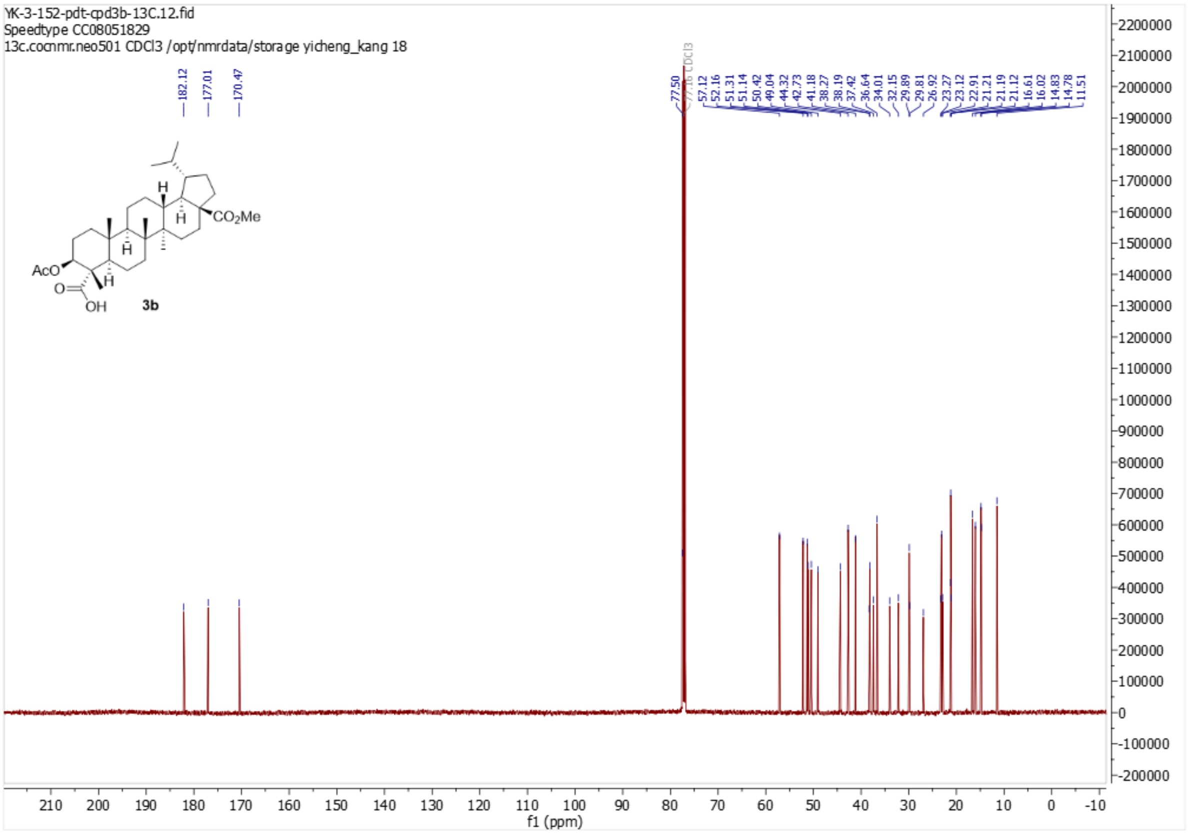
YK-3-152-pdt-cpd3b-13C.12.fid
Speedtype CC08051829
13c.cocnmr.neo501 CDCl3 /opt/nmrdata/storage yicheng_kang 18
182.12
177.01
170.47
77.50
CDCl3
57.12
52.16
51.31
51.14
50.42
49.04
44.32
42.73
41.18
38.27
38.19
37.42
36.64
34.01
32.15
29.89
29.81
26.92
23.27
23.12
22.91
21.21
21.19
21.12
16.61
16.02
14.83
14.78
11.51
AcO
O
OH
H
CO2Me
3b
f1 (ppm)

Compound 3c:

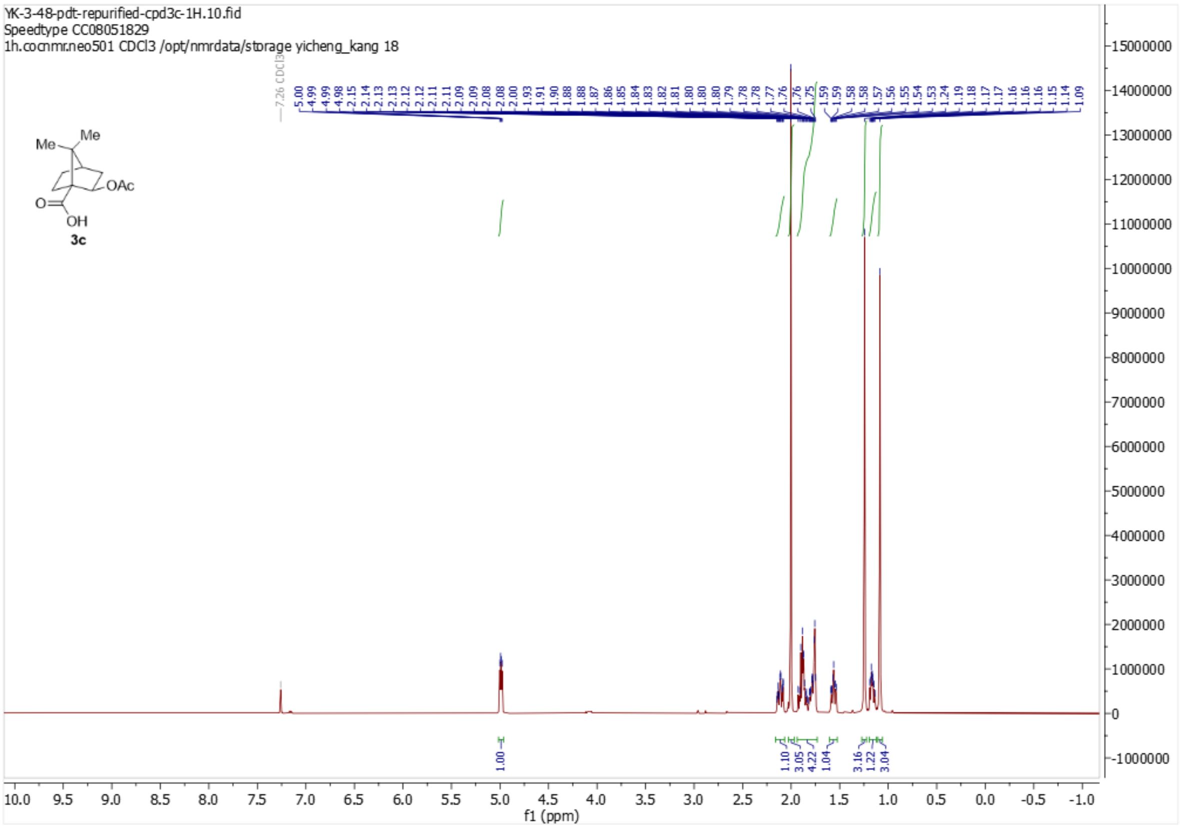

YK-3-48-pdt-repurified-cpd3c-1H.10.fid
Speedtype CC08051829
1h.cocnmr.neo501 CDCl3 /opt/nmrdata/storage yicheng_kang 18
3c
f1 (ppm)

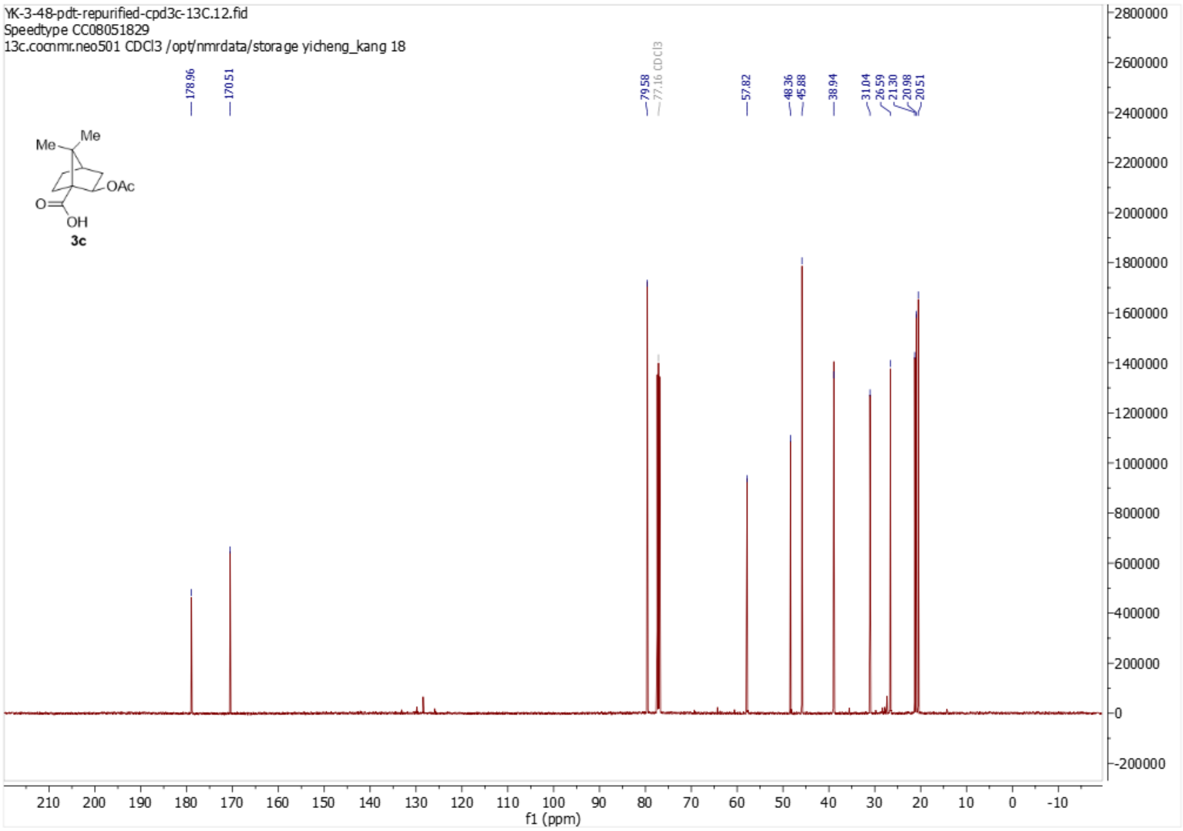

YK-3-48-pdt-repurified-cpd3c-13C.12.fid
Speedtype CC08051829
13c.cocnmr.neo501 CDCl3 /opt/nmrdata/storage yicheng_kang 18
178.96
170.51
79.58
77.16 CDCl3
57.82
48.36
45.88
38.94
31.04
26.59
21.30
20.98
20.51
3c
f1 (ppm)

Compound 3d:

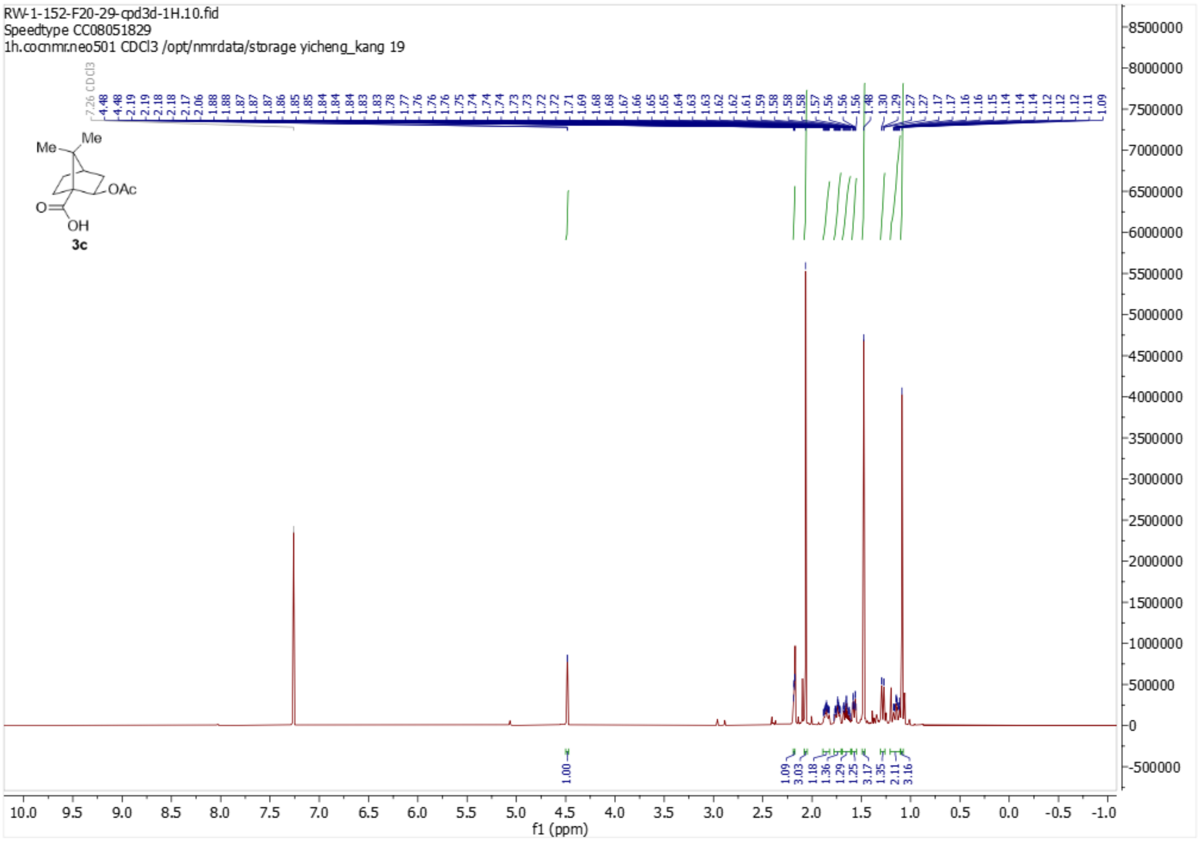
RW-1-152-F20-29-cpd3d-1H.10.fid
Speedtype CC08051829
1h.cocnmr.neo501 CDCl3 /opt/nmrdata/storage yicheng_kang 19
7.26 CDCl3
Me
Me
OAc
O
OH
3c
1.00
1.09
3.03
1.18
1.36
1.29
1.25
3.17
1.35
2.11
3.16
f1 (ppm)

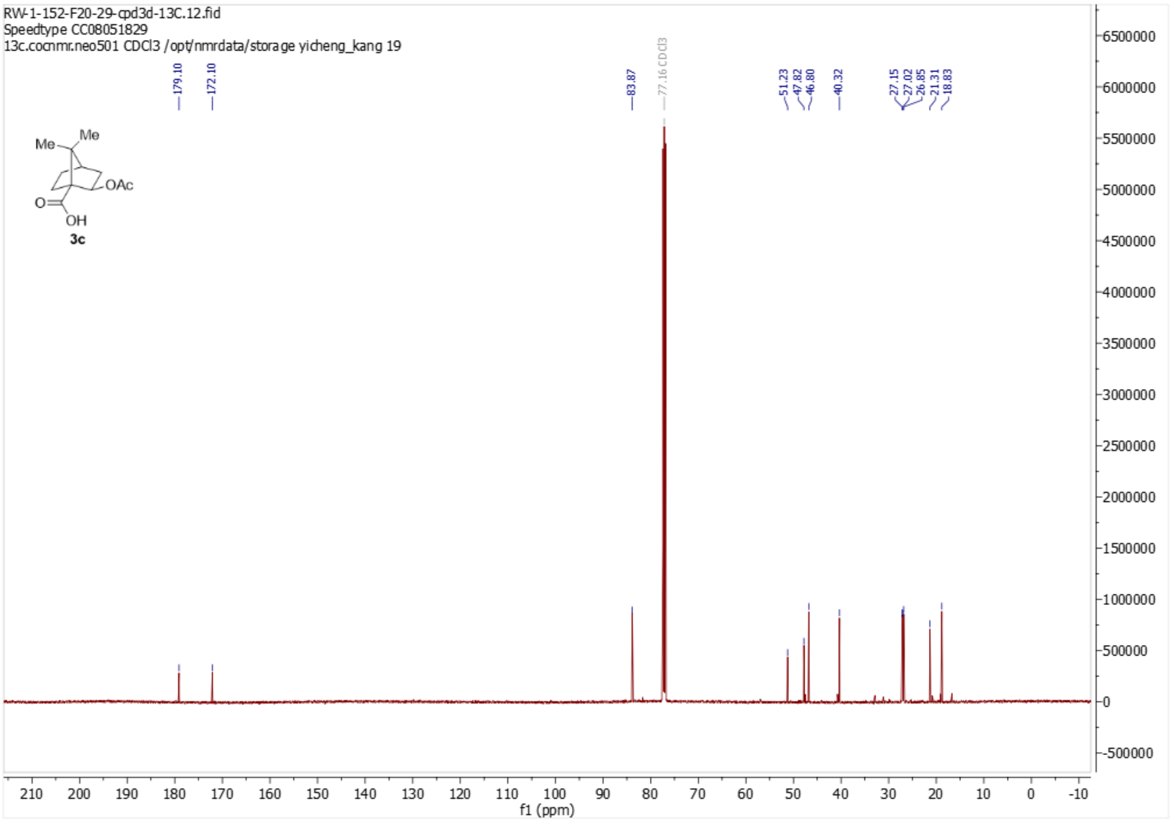
RW-1-152-F20-29-cpd3d-13C.12.fid
Speedtype CC08051829
13c.cocnmr.neo501 CDCl3 /opt/nmrdata/storage yicheng_kang 19
179.10
172.10
83.87
77.16 CDCl3
51.23
47.82
46.80
40.32
27.15
27.02
26.85
21.31
18.83
Me
Me
OAc
O
OH
3c
f1 (ppm)

Compound 3e:

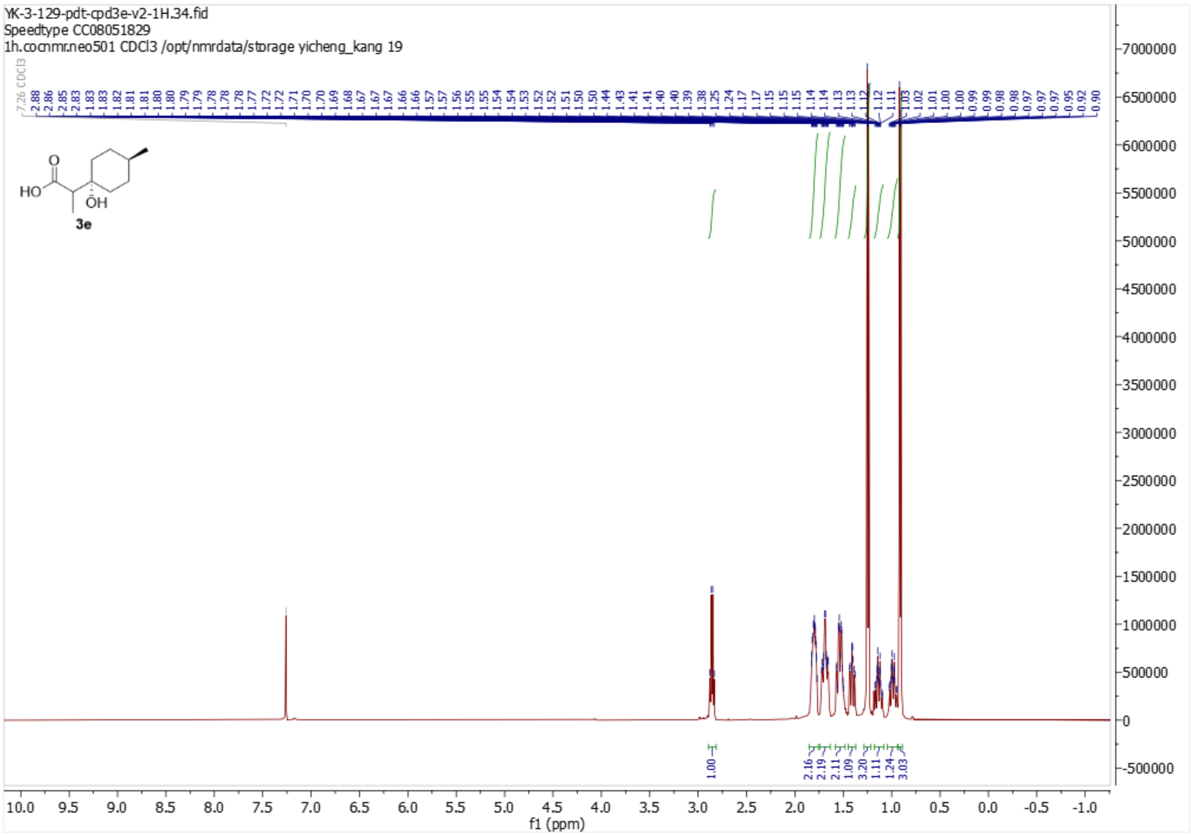
YK-3-129-pdt-cpd3e-v2-1H.34.fid
Speedtype CC08051829
1h.cocnmr.neo501 CDCl3 /opt/nmrdata/storage yicheng_kang 19
3e
f1 (ppm)

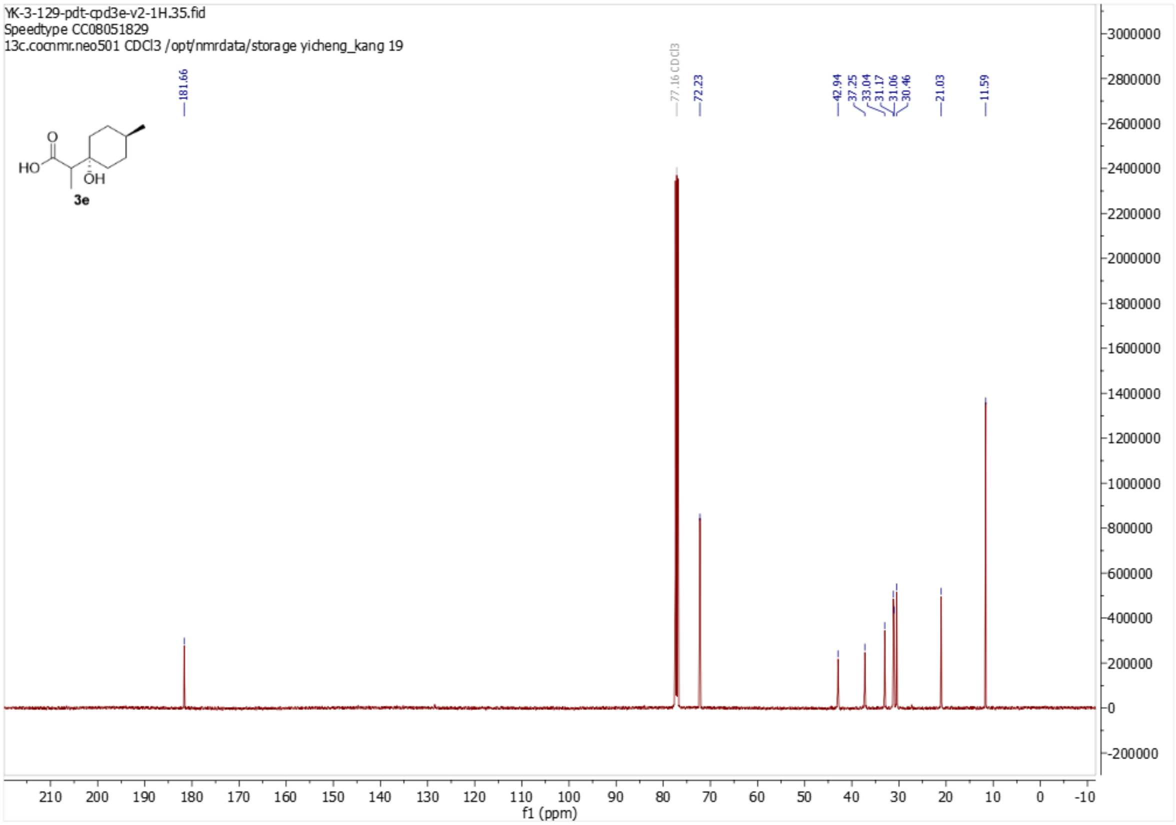
YK-3-129-pdt-cpd3e-v2-1H.35.fid
Speedtype CC08051829
13c.cocnmr.neo501 CDCl3 /opt/nmrdata/storage yicheng_kang 19
181.66
77.16 CDCl3
72.23
42.94
37.25
33.04
31.17
31.06
30.46
21.03
11.59
3e
f1 (ppm)

Compound 4aa:

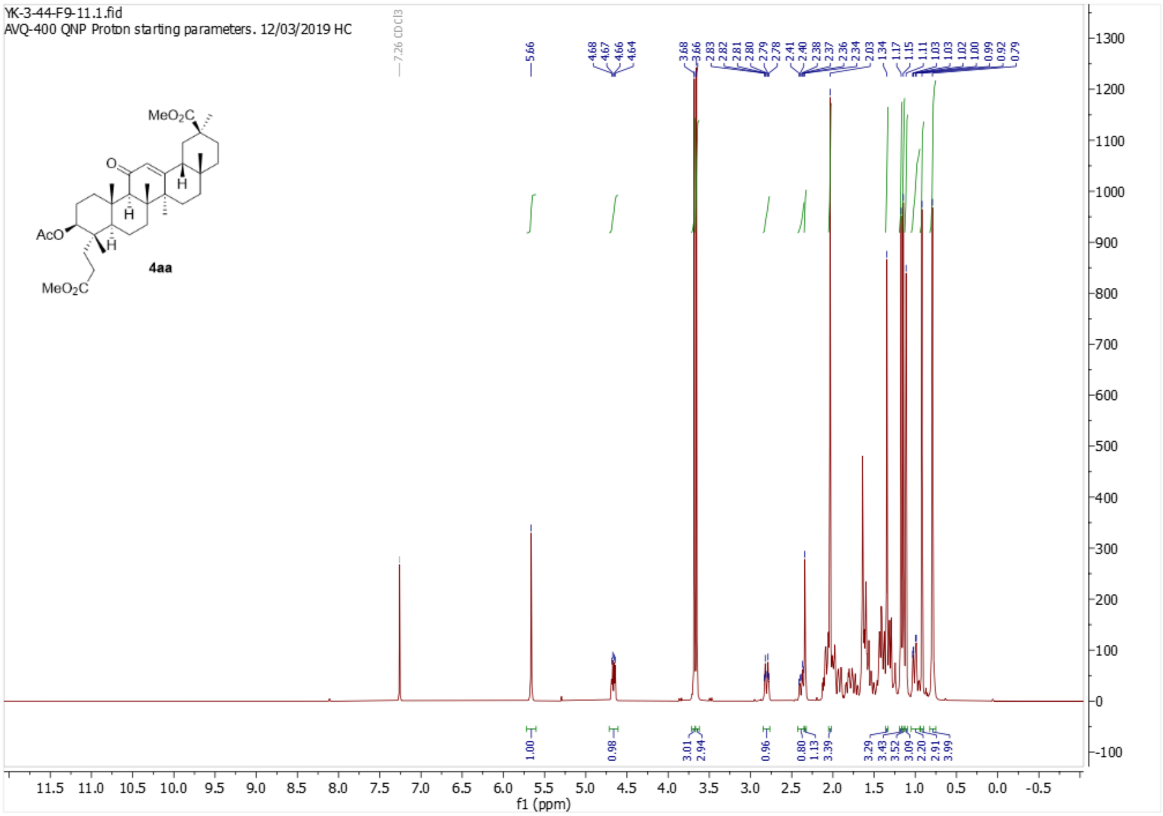

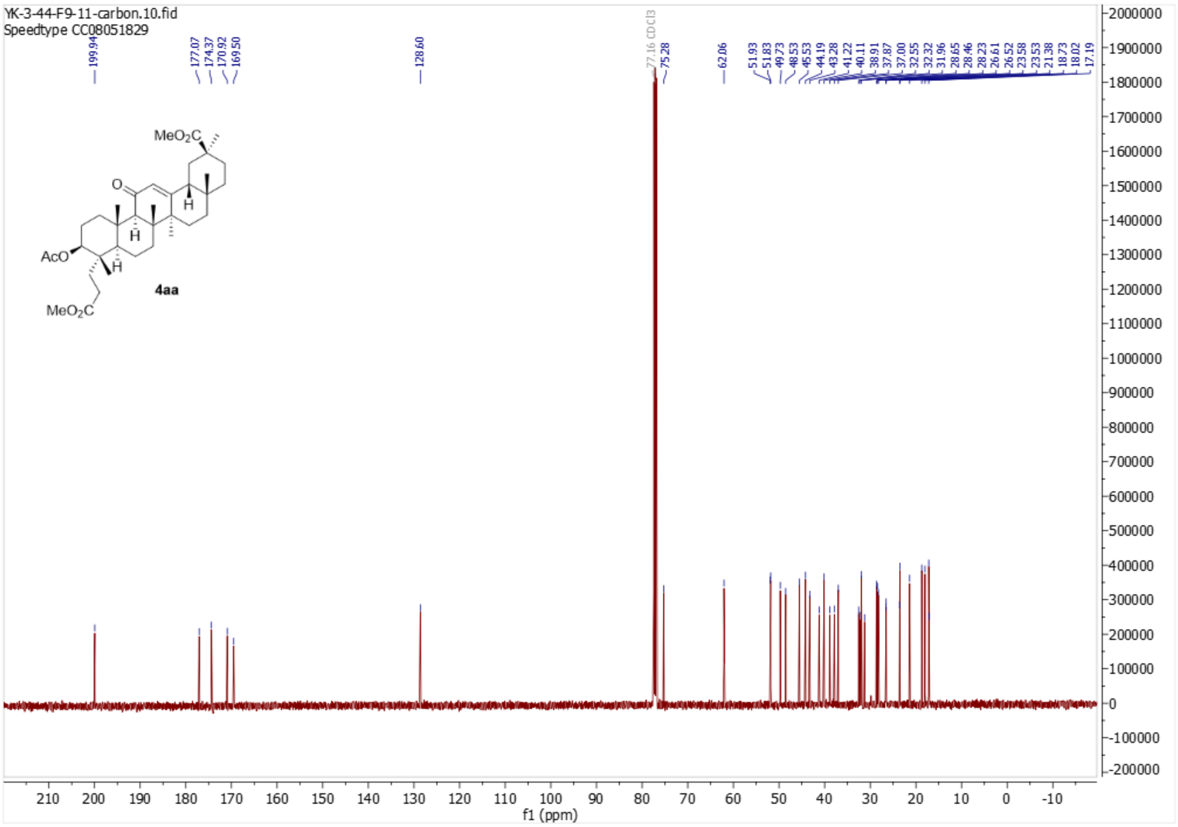

Compound 4ab:

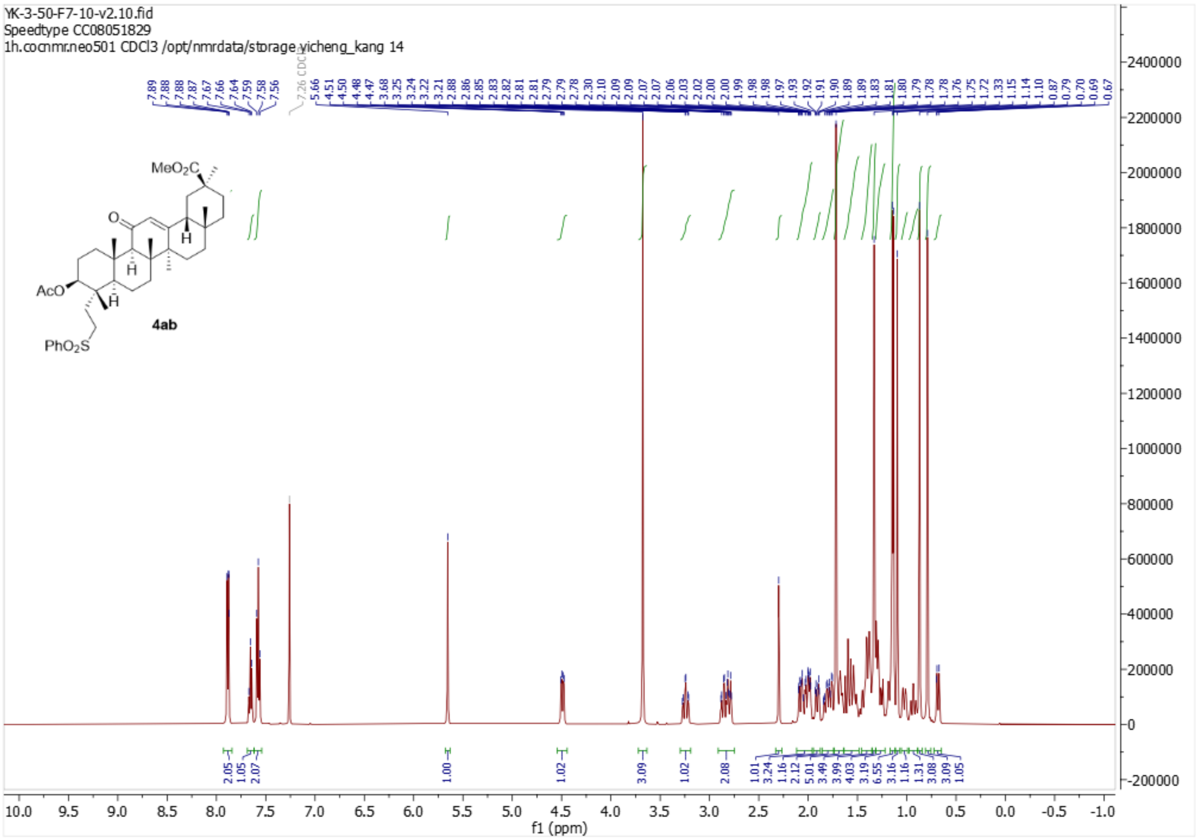

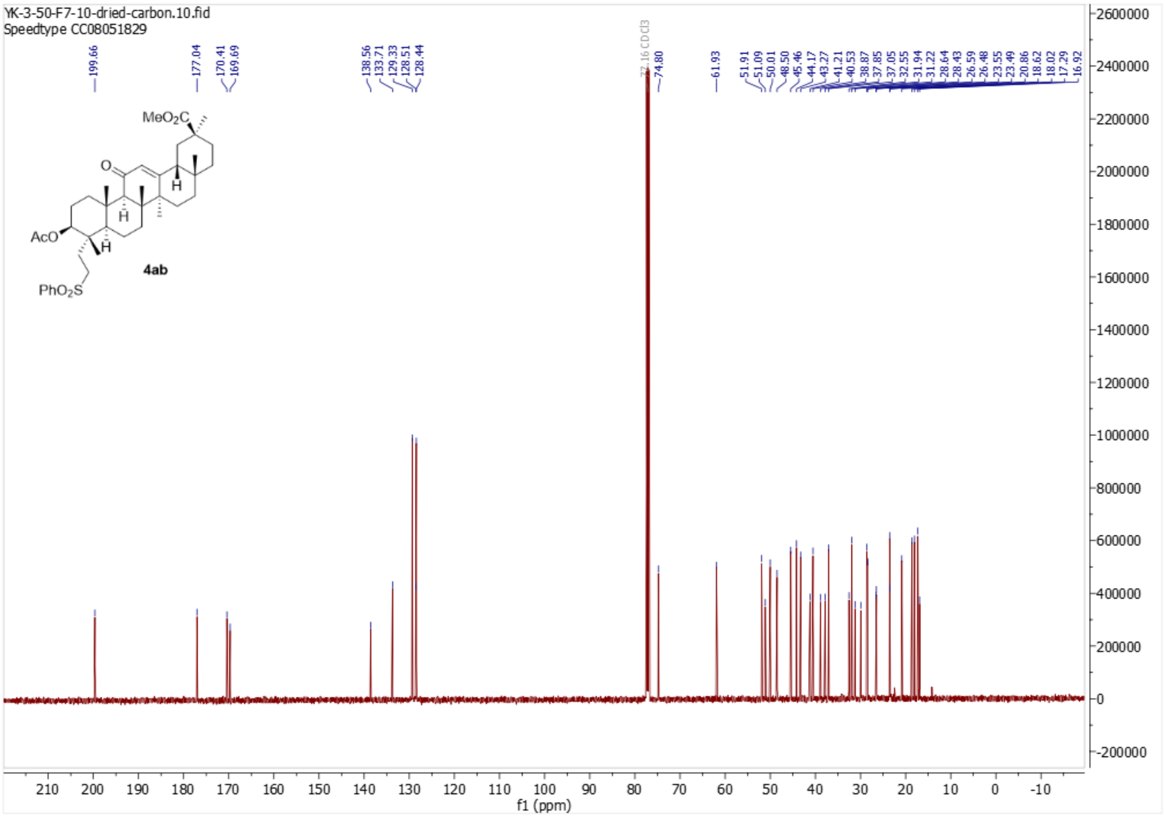

Compound 4ba:

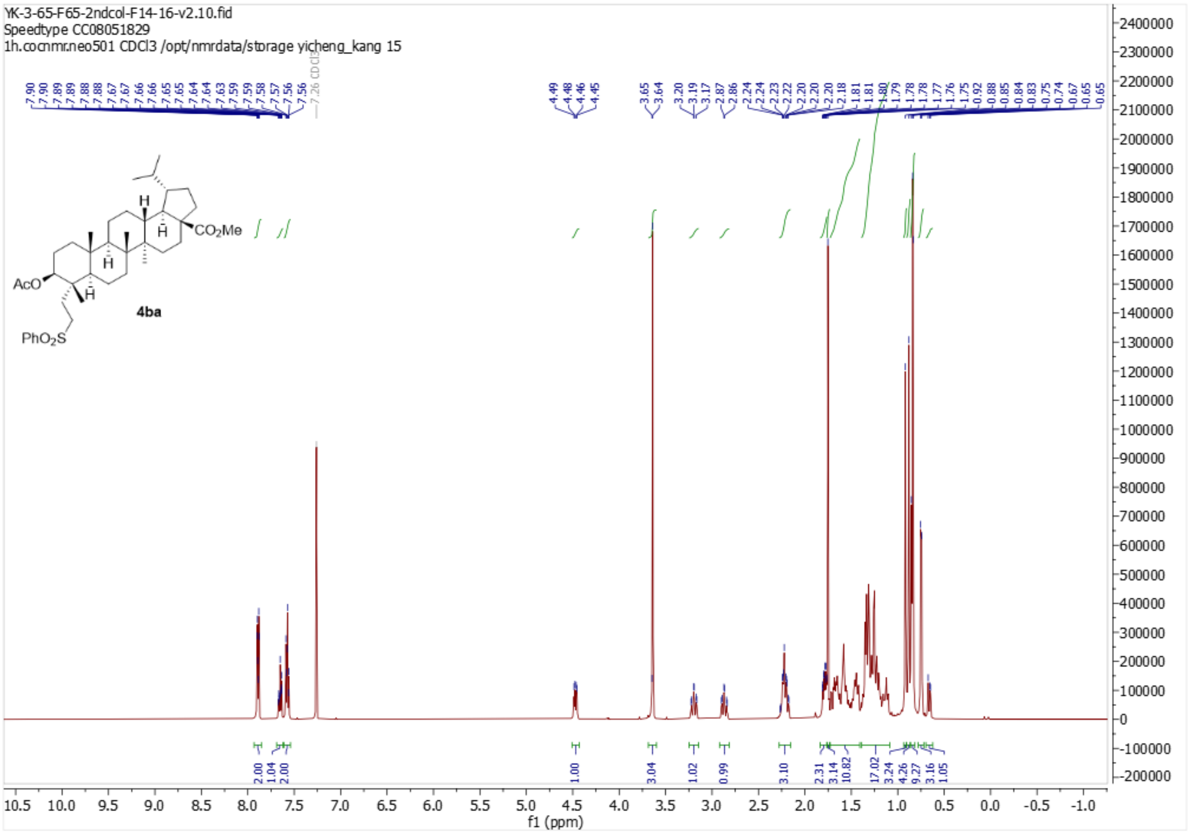

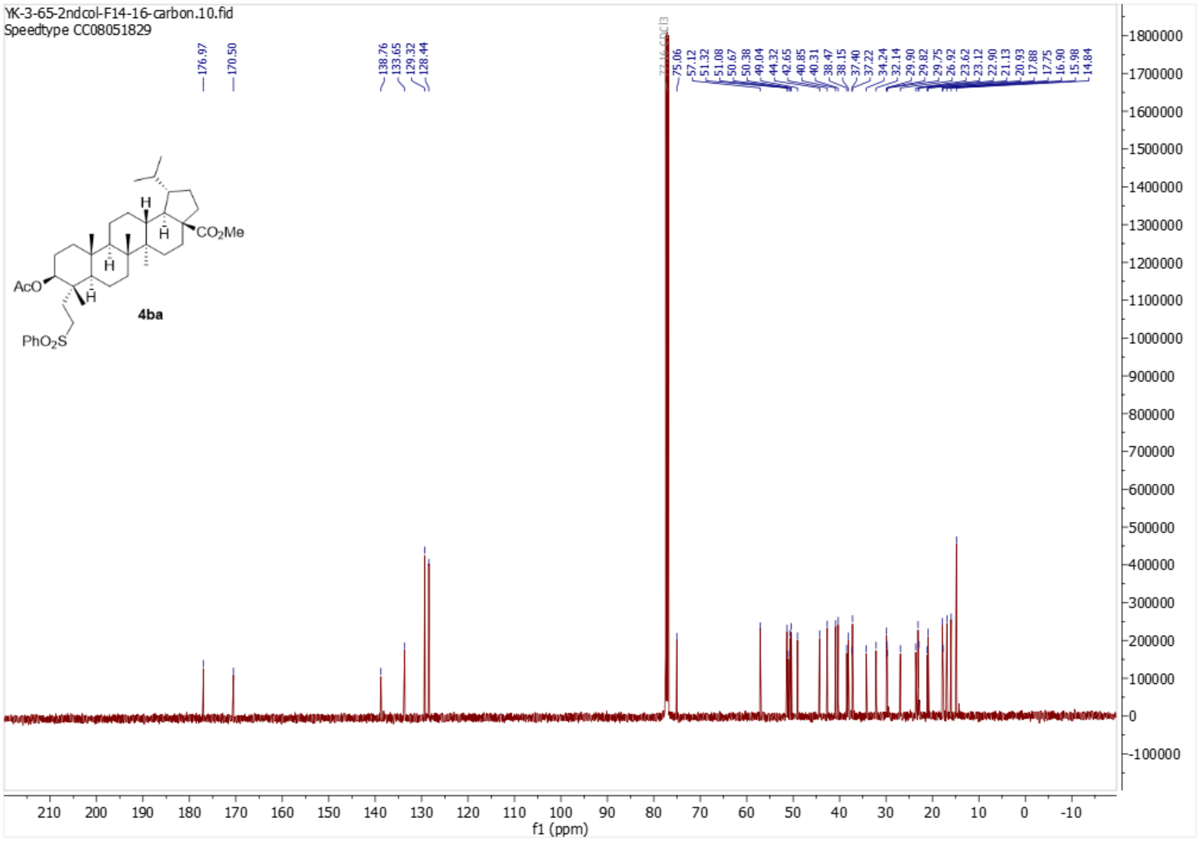

Compound 4ac:

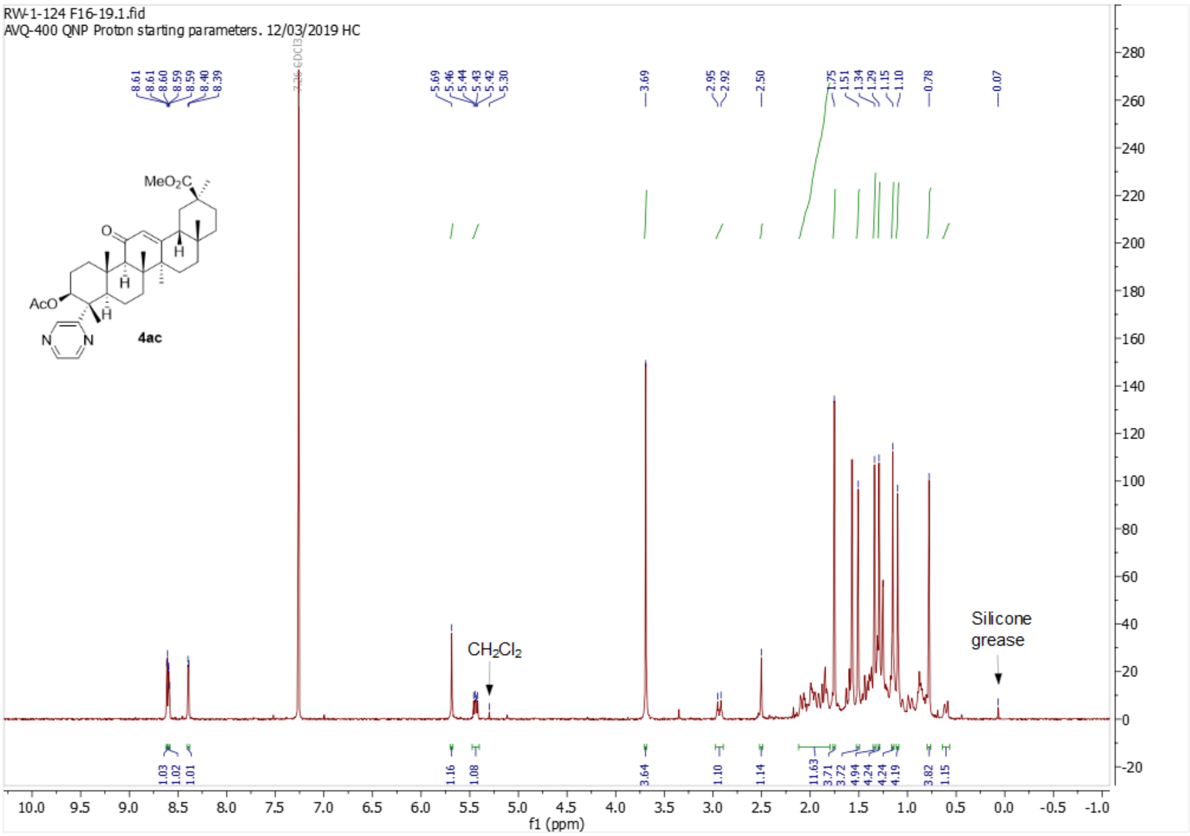

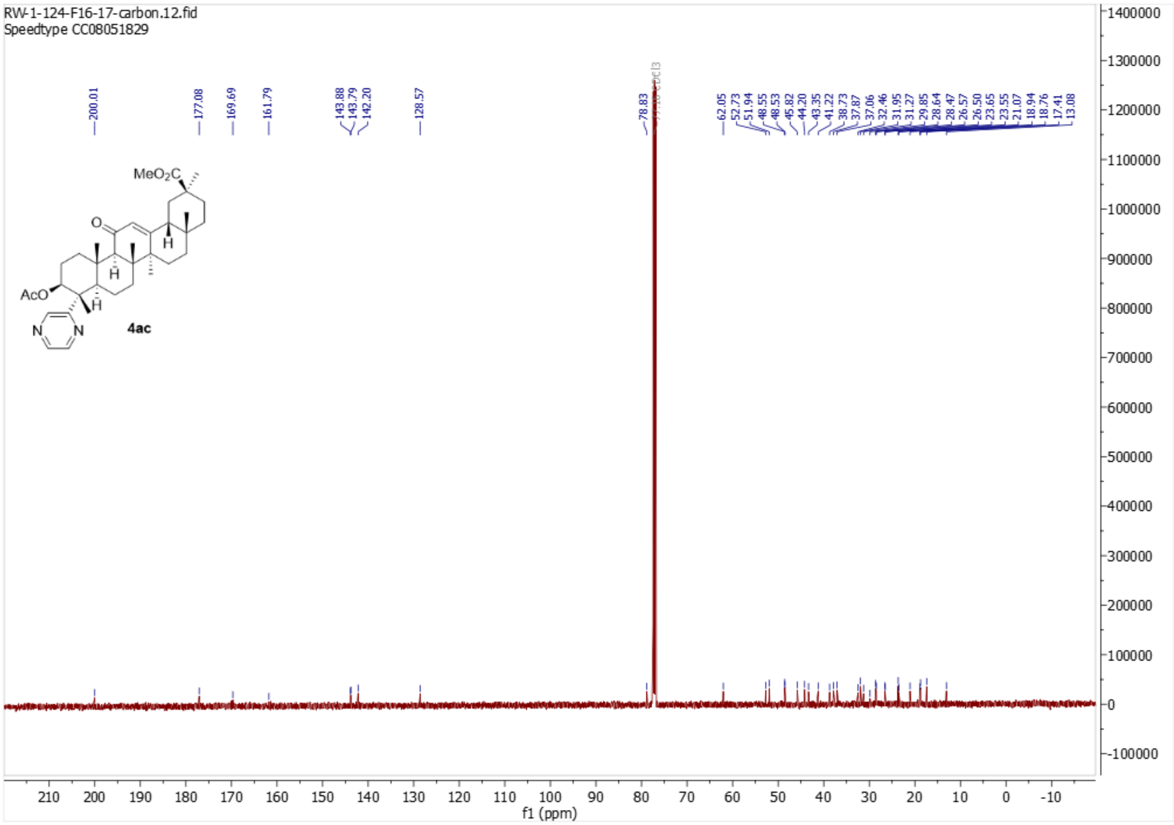

Compound 4bb:

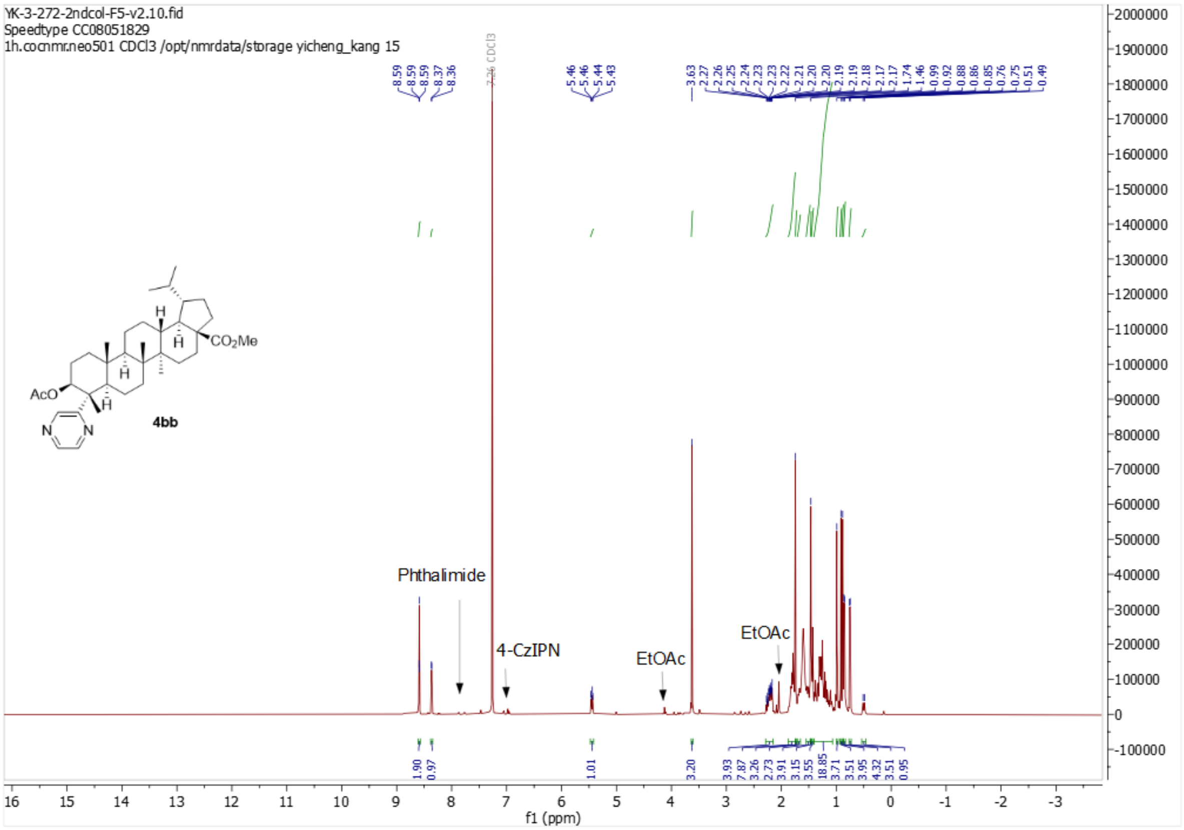

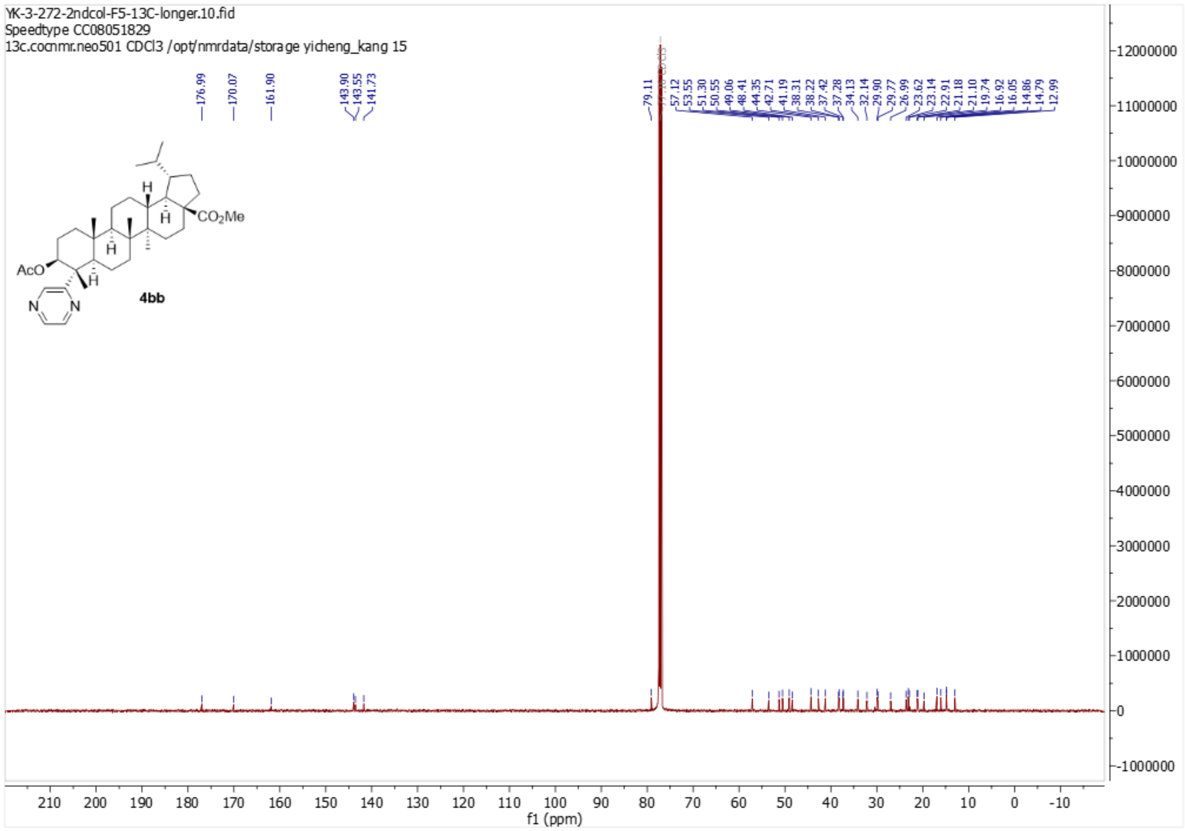

Compound 4ca:

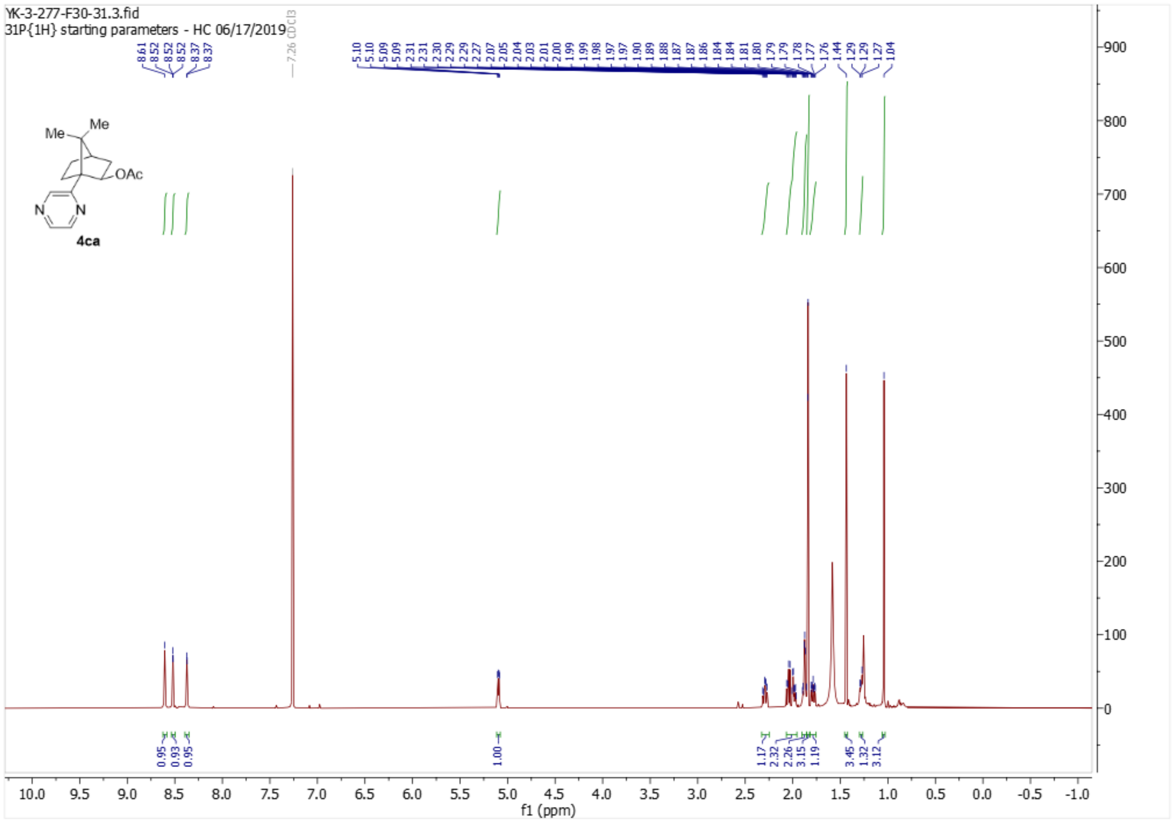

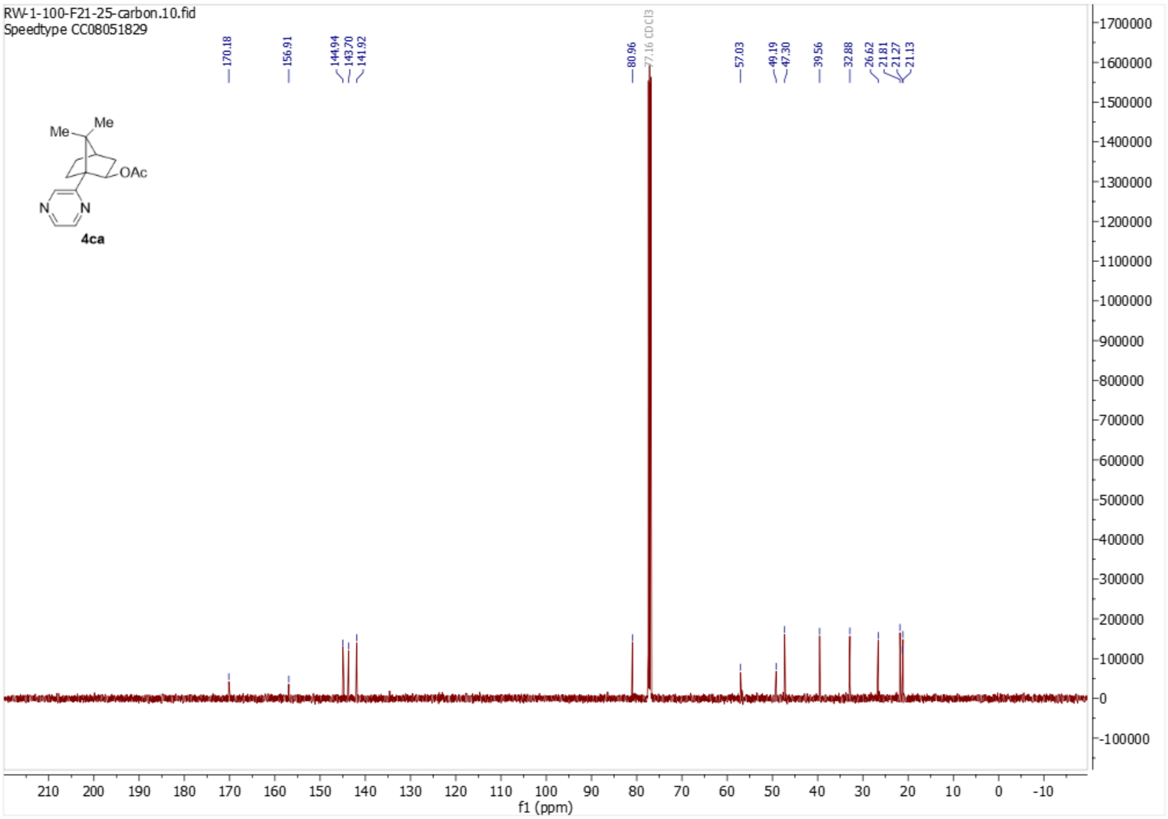

Compound 4cb:

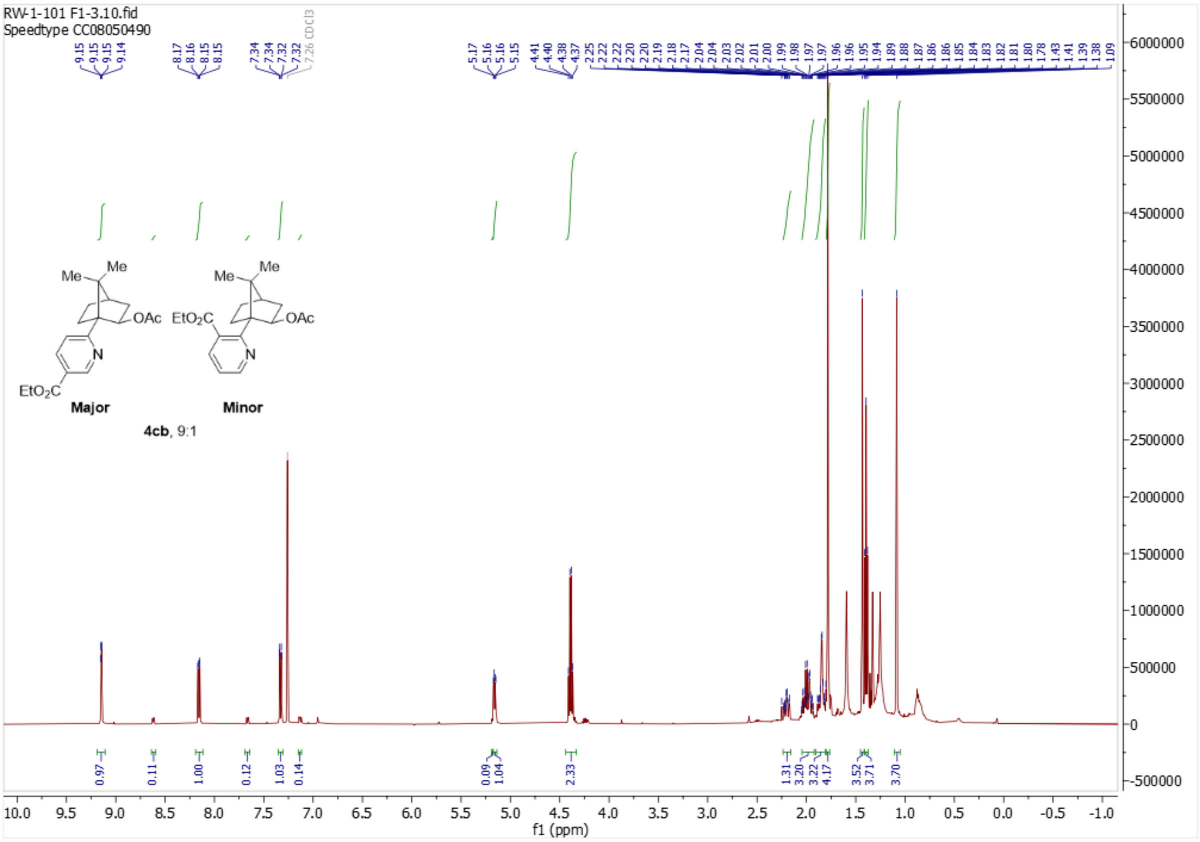

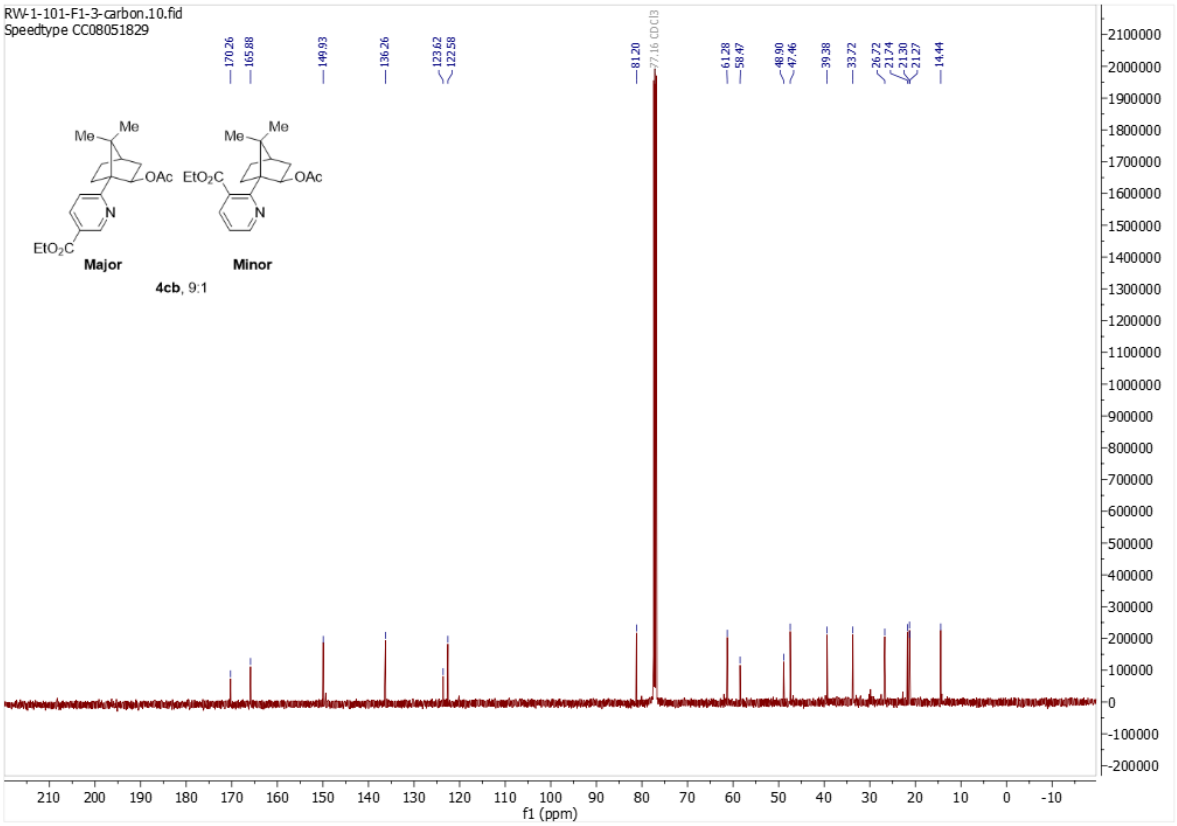

Compound 4cc:

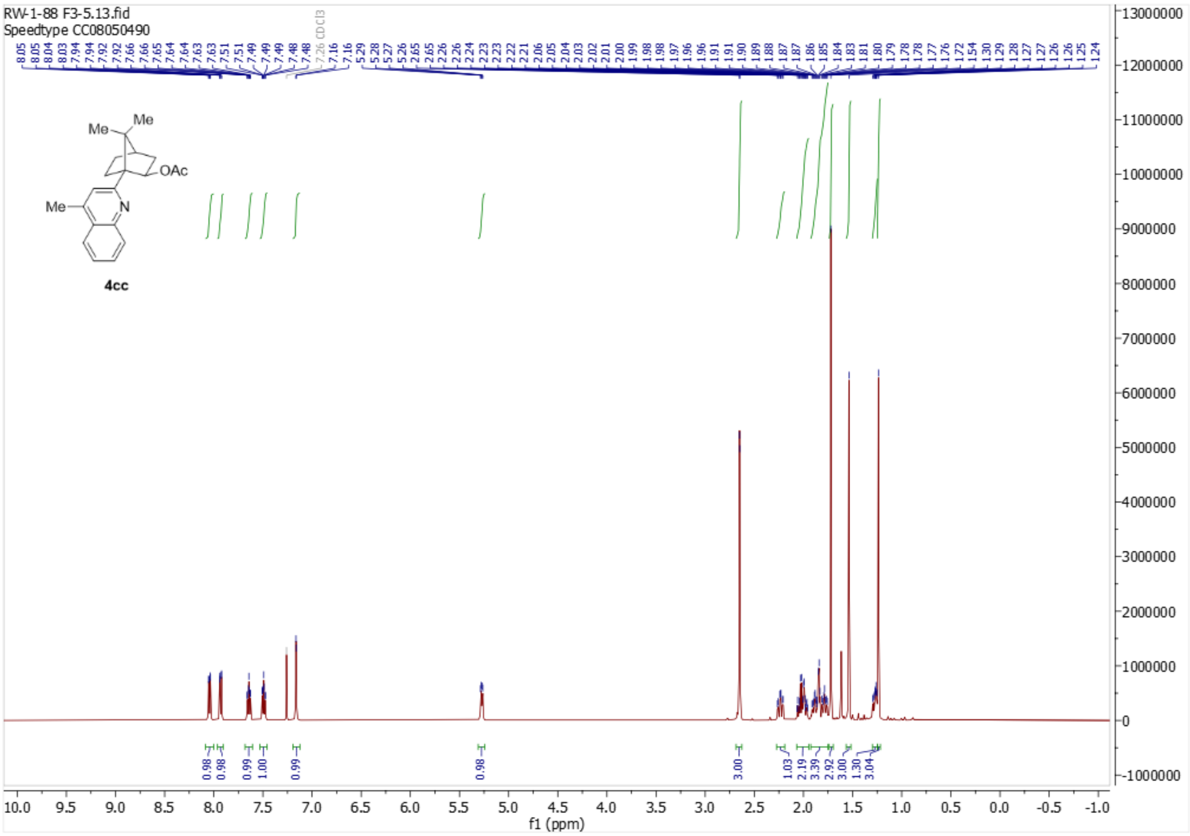

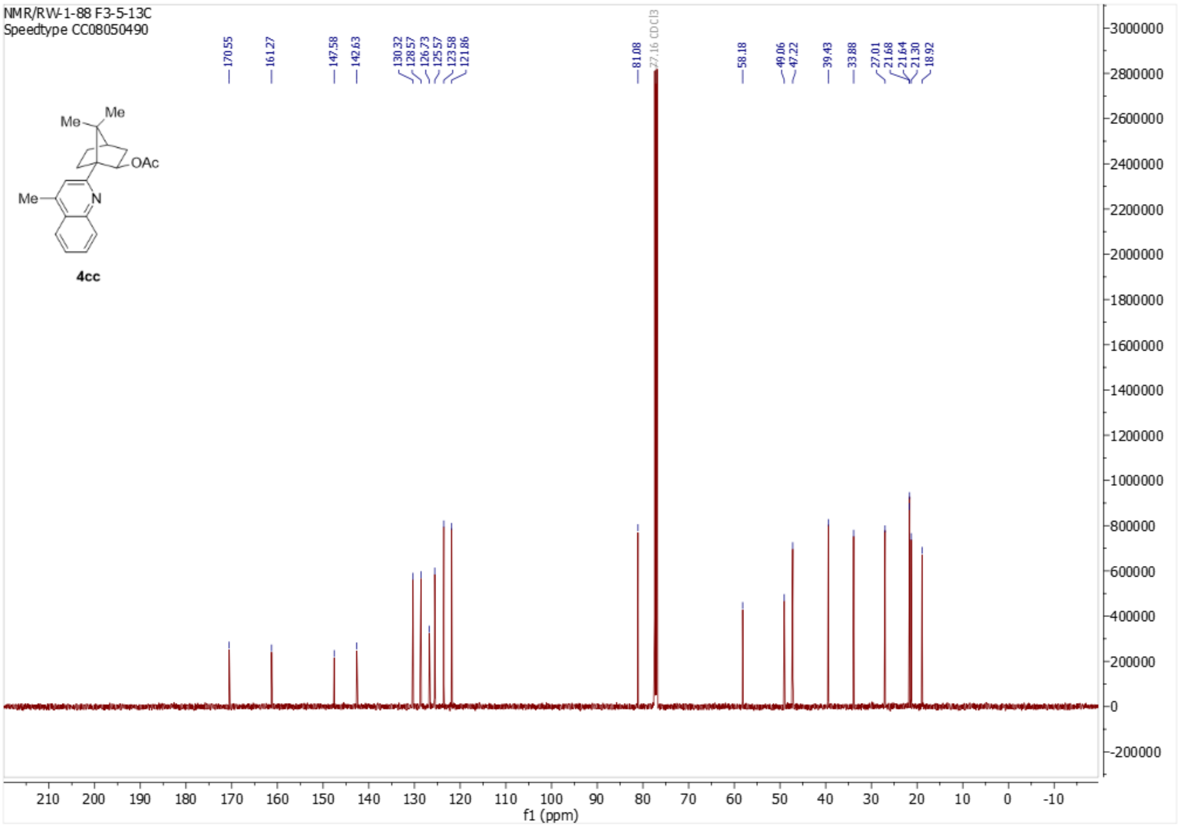

Compound 4cd:

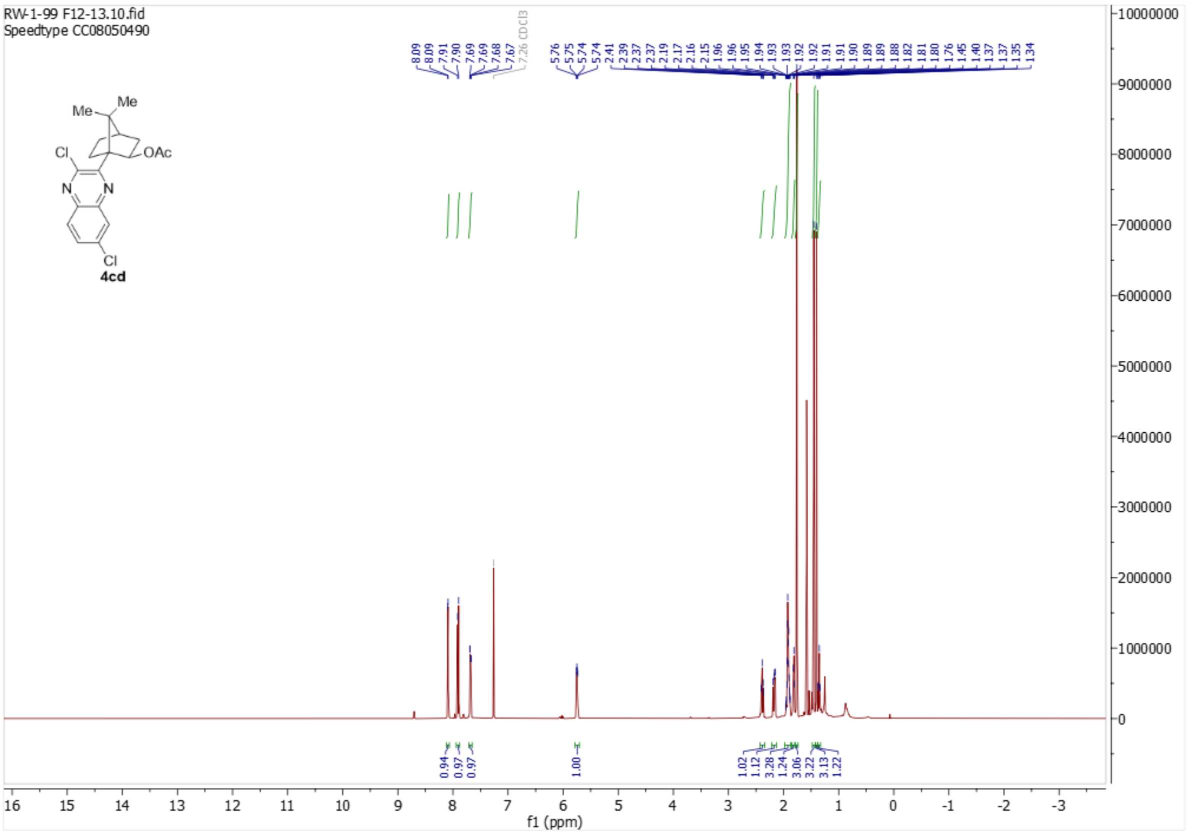

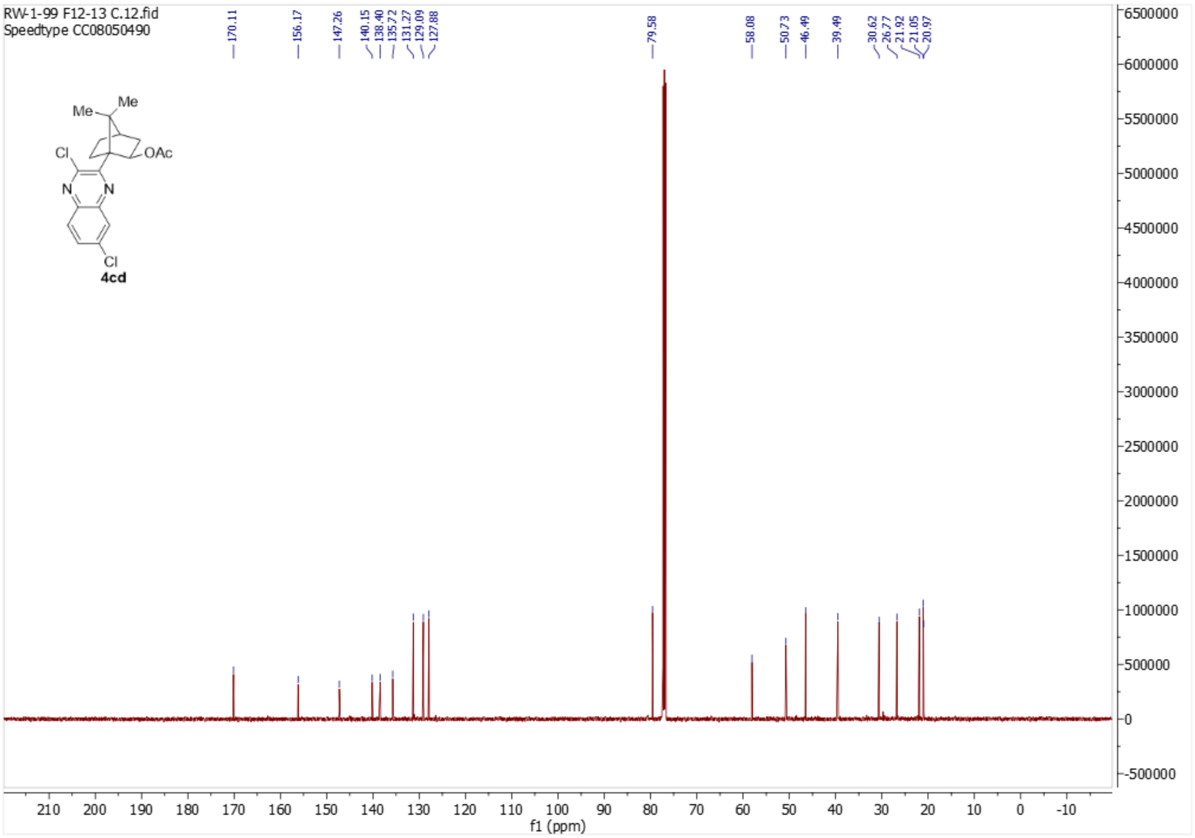

Compound 4ad:

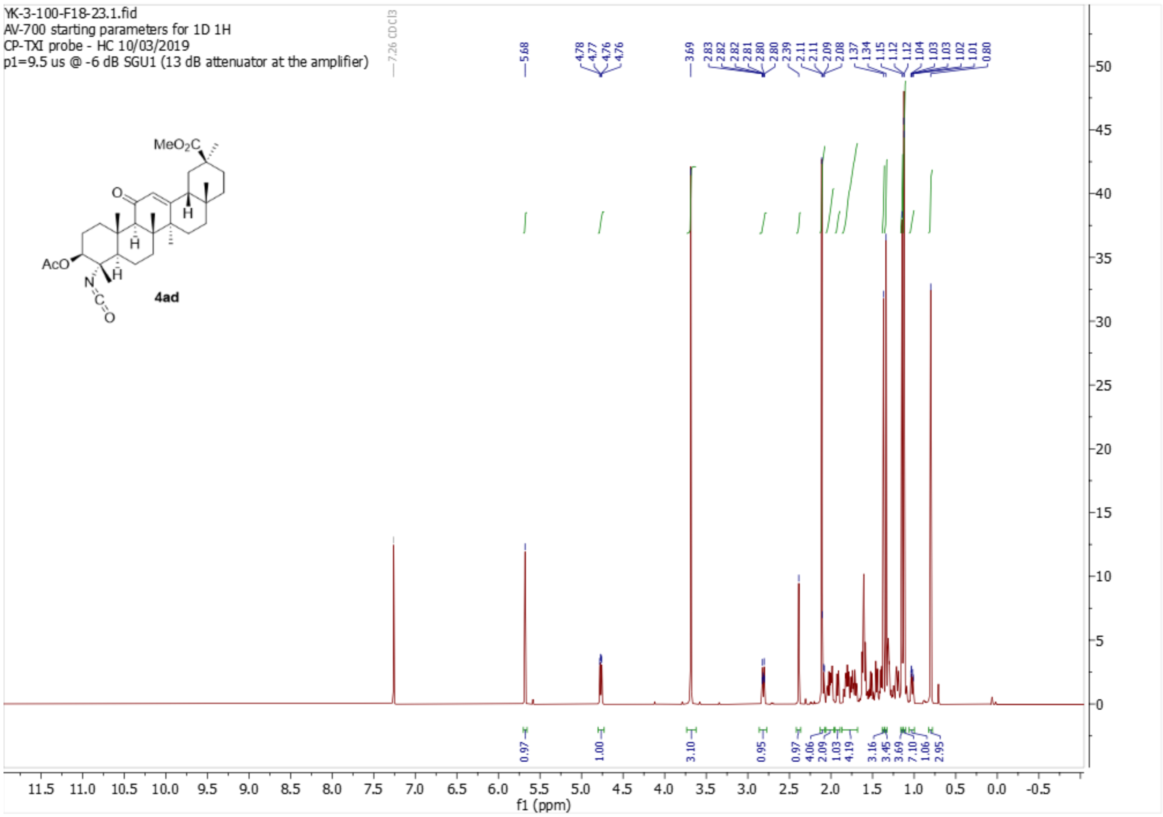

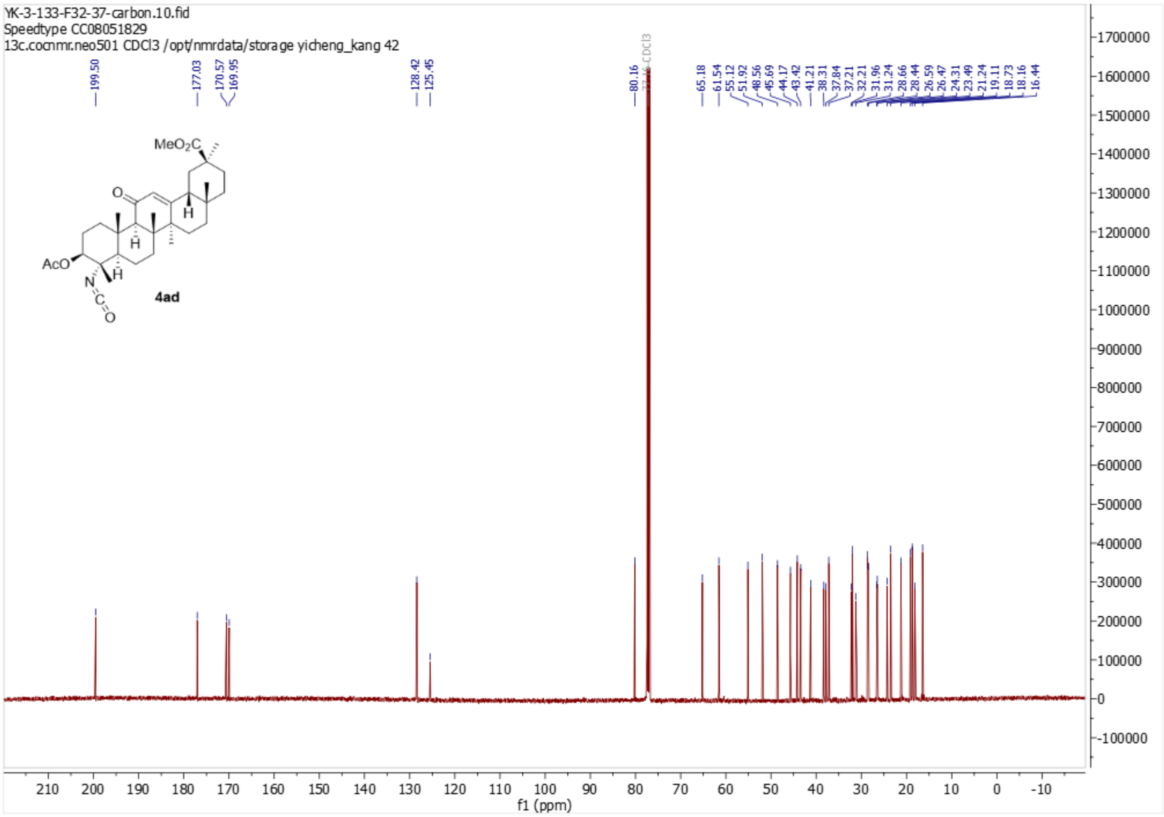

Compound 4bc:

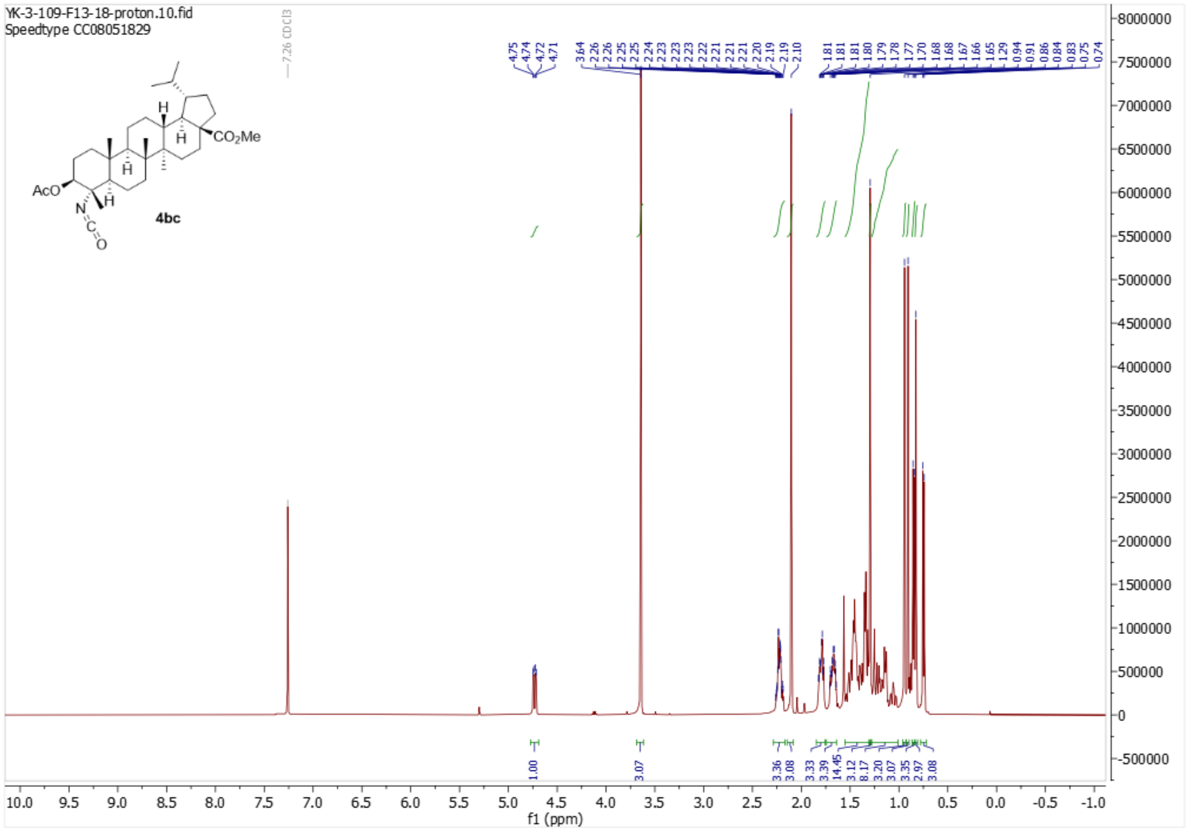

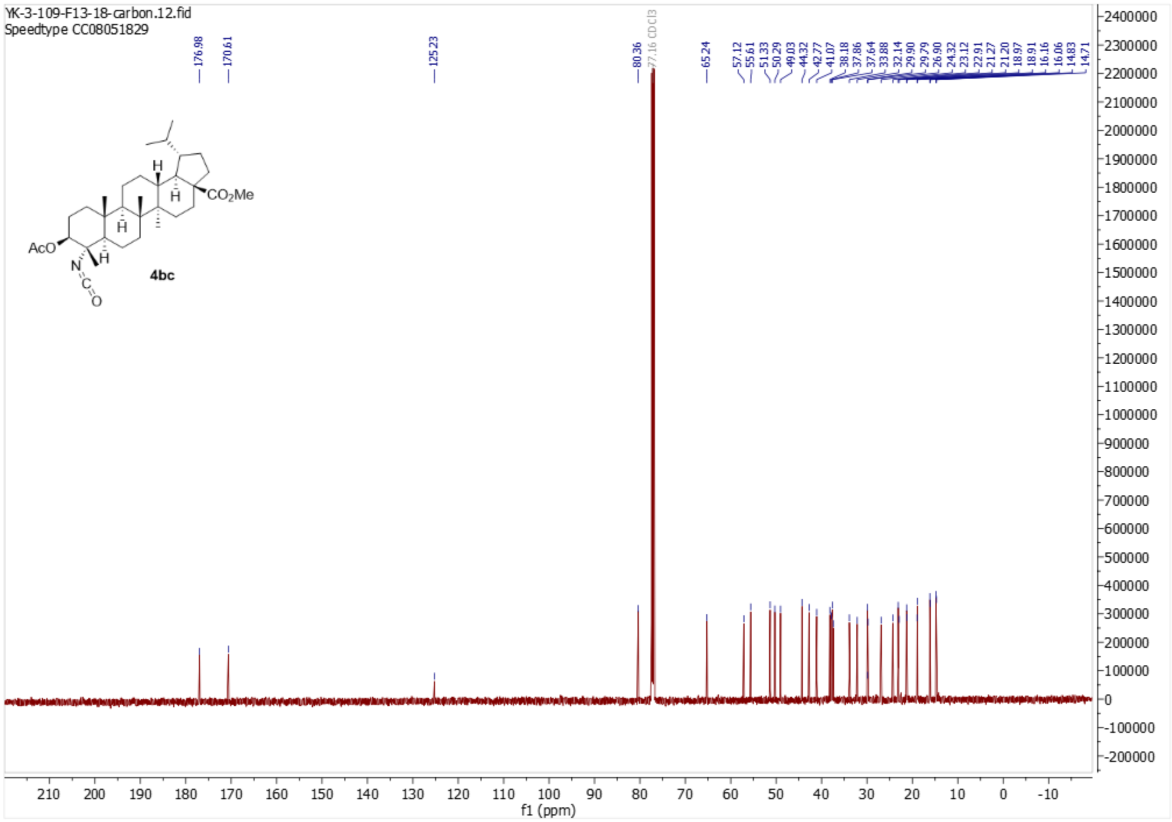

Compound 4ah:

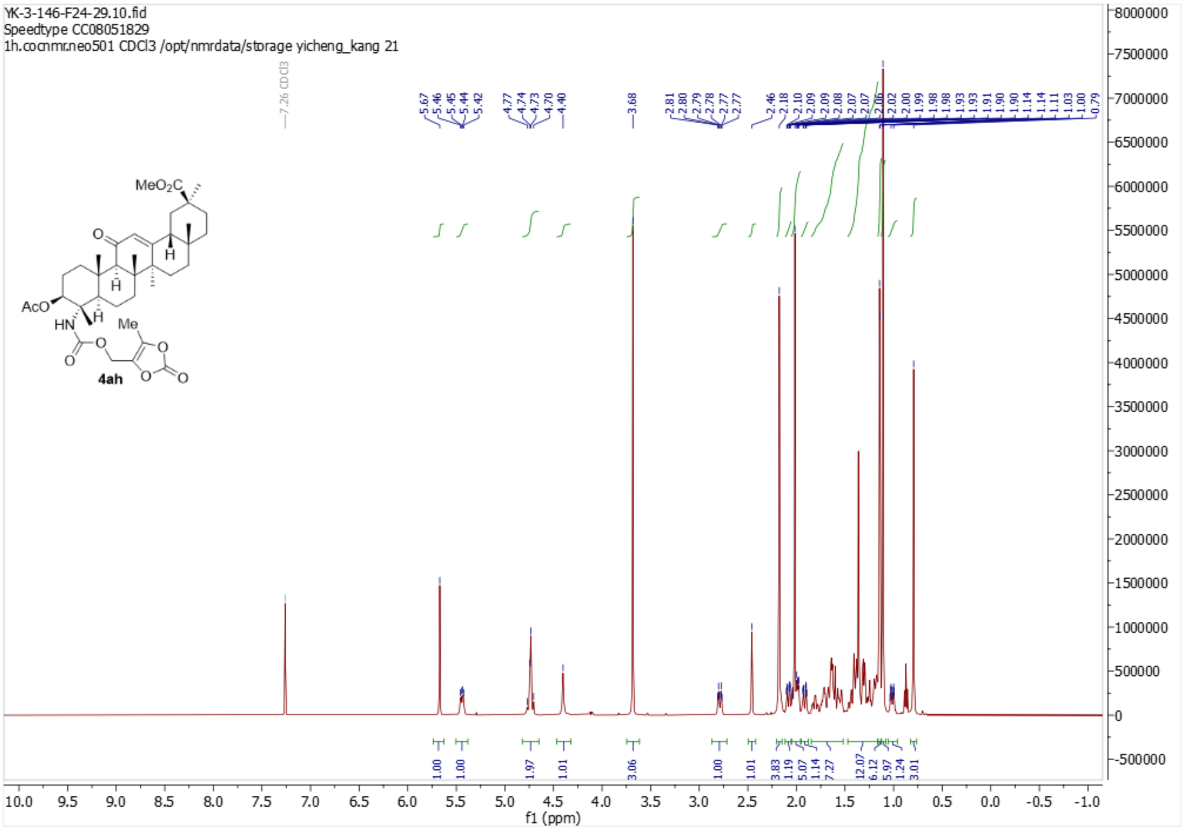

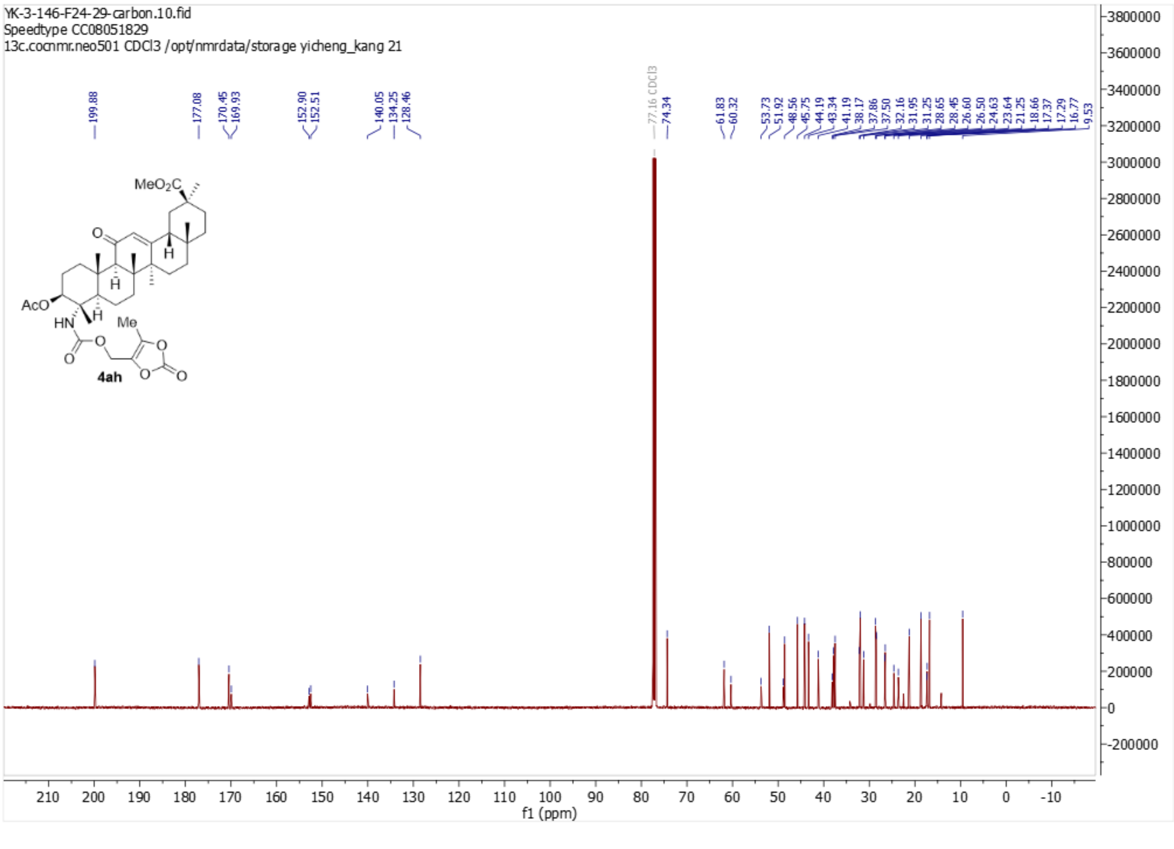

Compound 4ae:

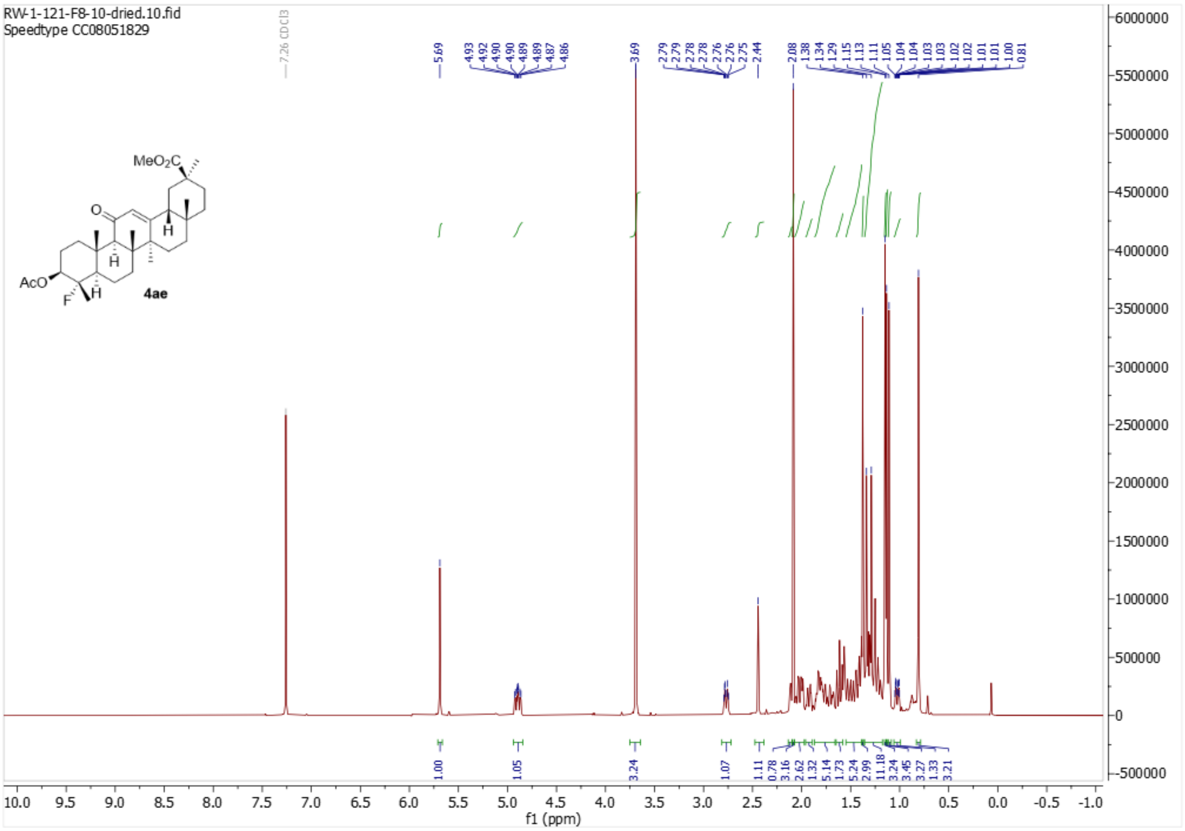

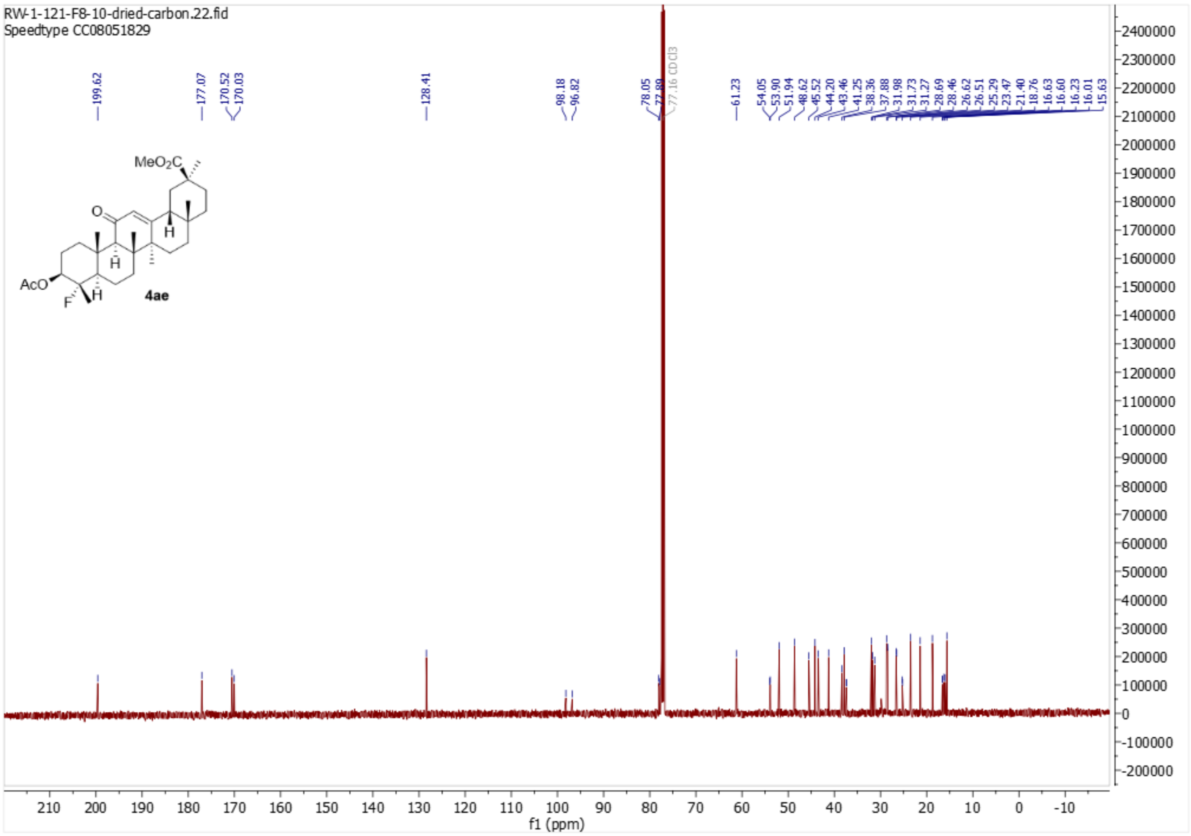

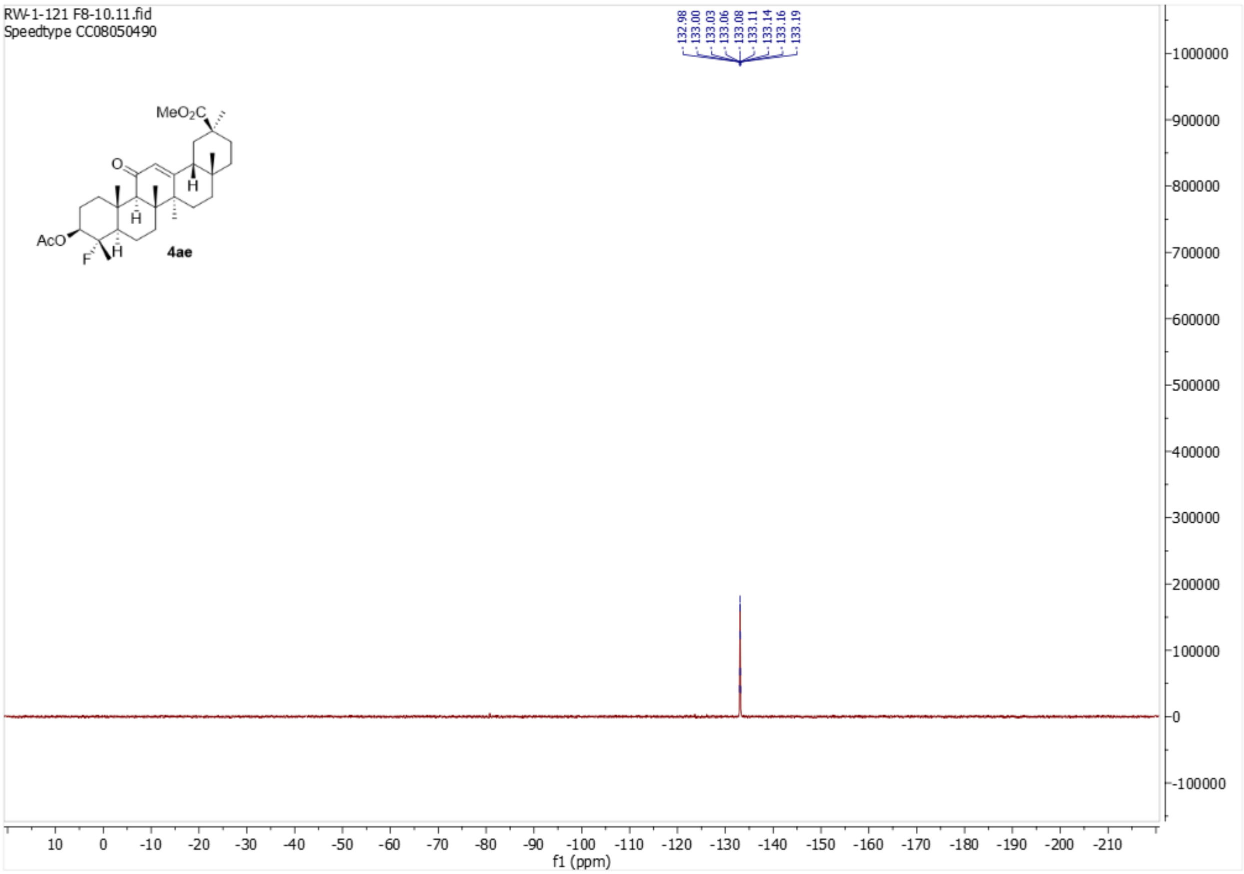
RW-1-121 F8-10.11.fid
Speedtype CC08050490
132.98
133.00
133.03
133.06
133.08
133.11
133.14
133.16
133.19
MeO2C
O
H
H
AcO
F
H
4ae
1000000
900000
800000
700000
600000
500000
400000
300000
200000
100000
0
-100000
10 0 -10 -20 -30 -40 -50 -60 -70 -80 -90 -100 -110 -120 -130 -140 -150 -160 -170 -180 -190 -200 -210
f1 (ppm)

Compound 4bd:

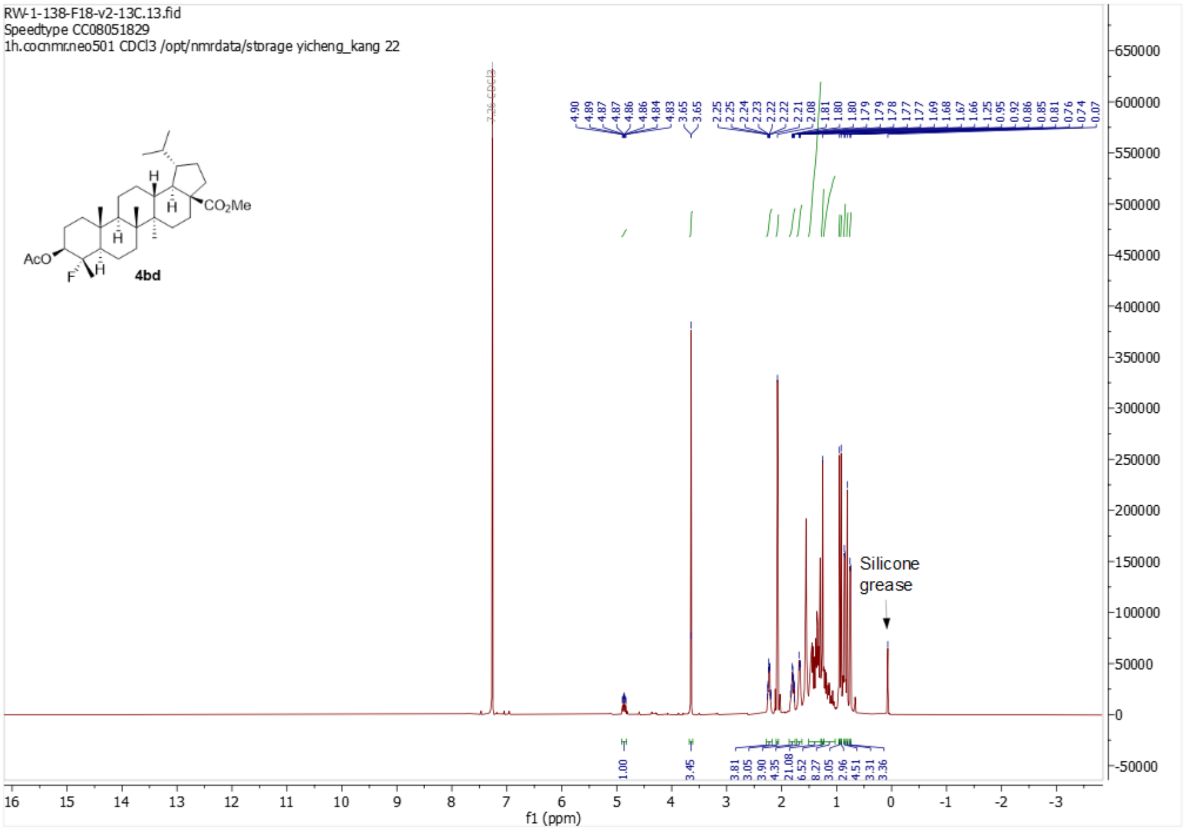

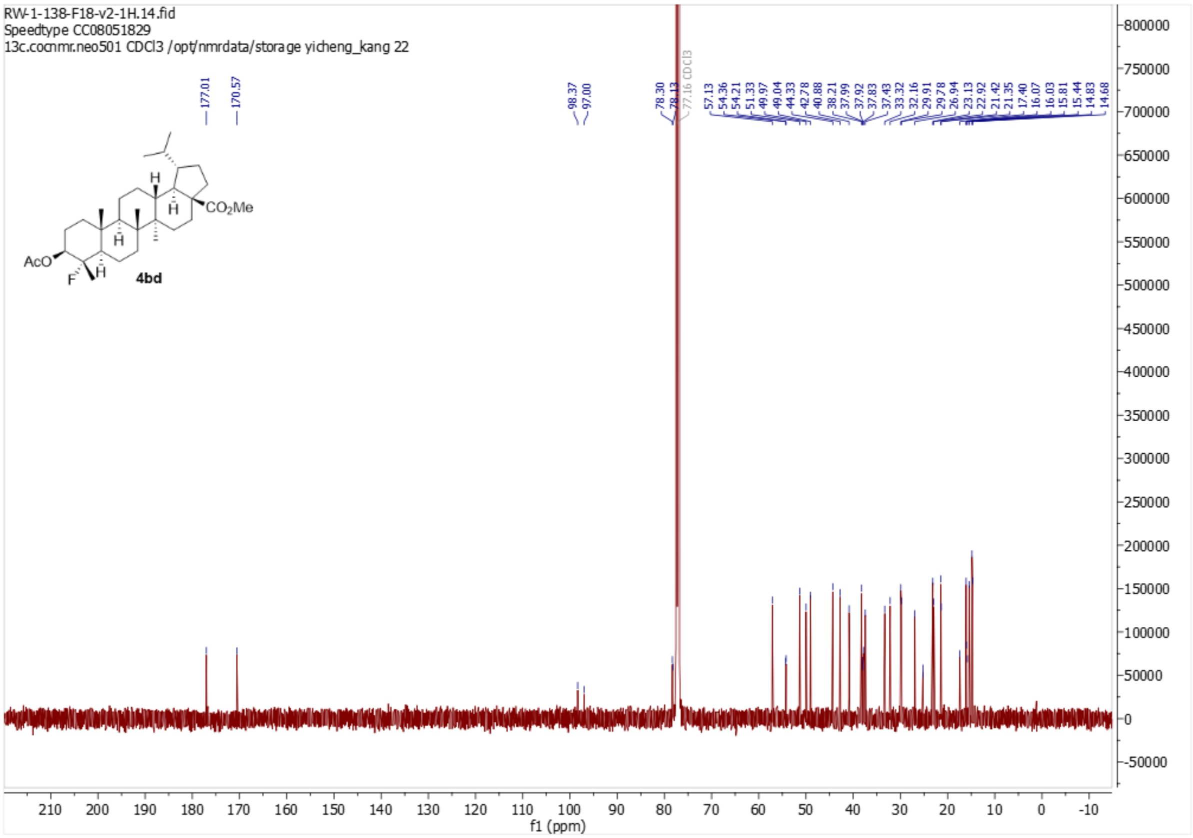

Compound 4da:

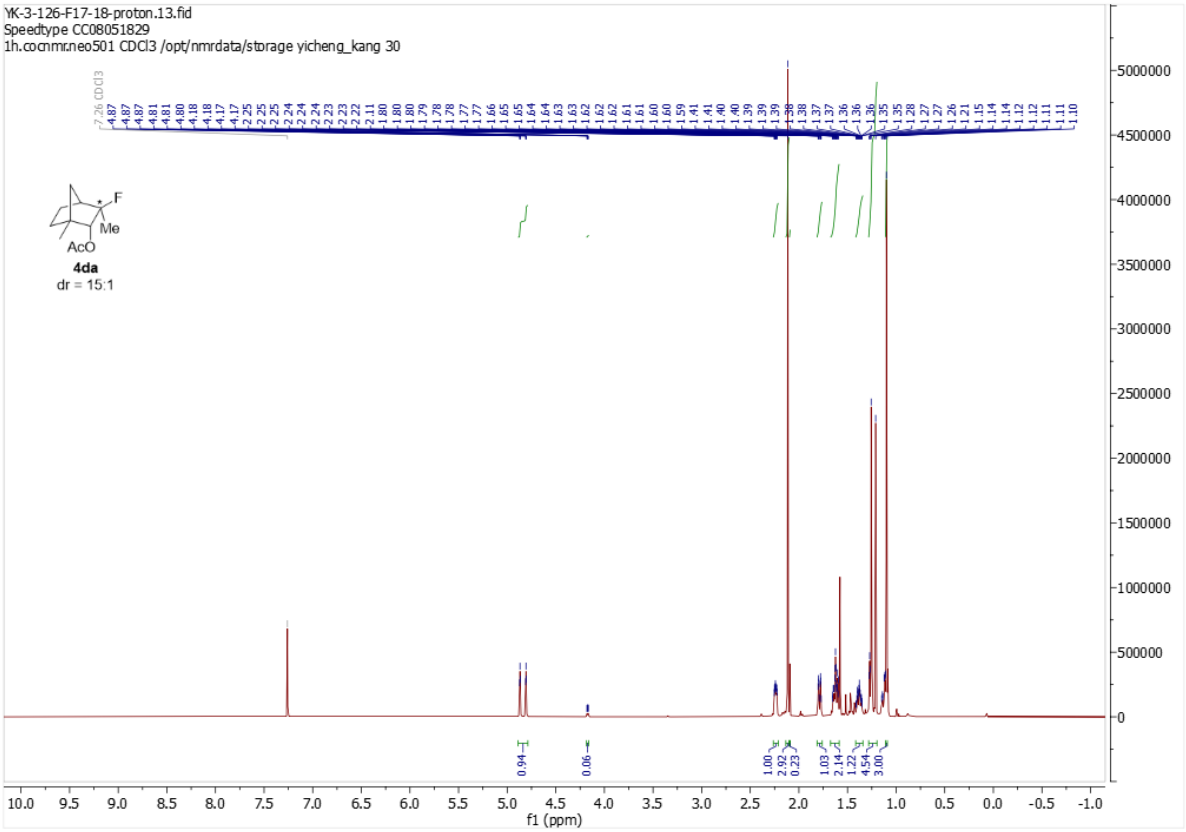

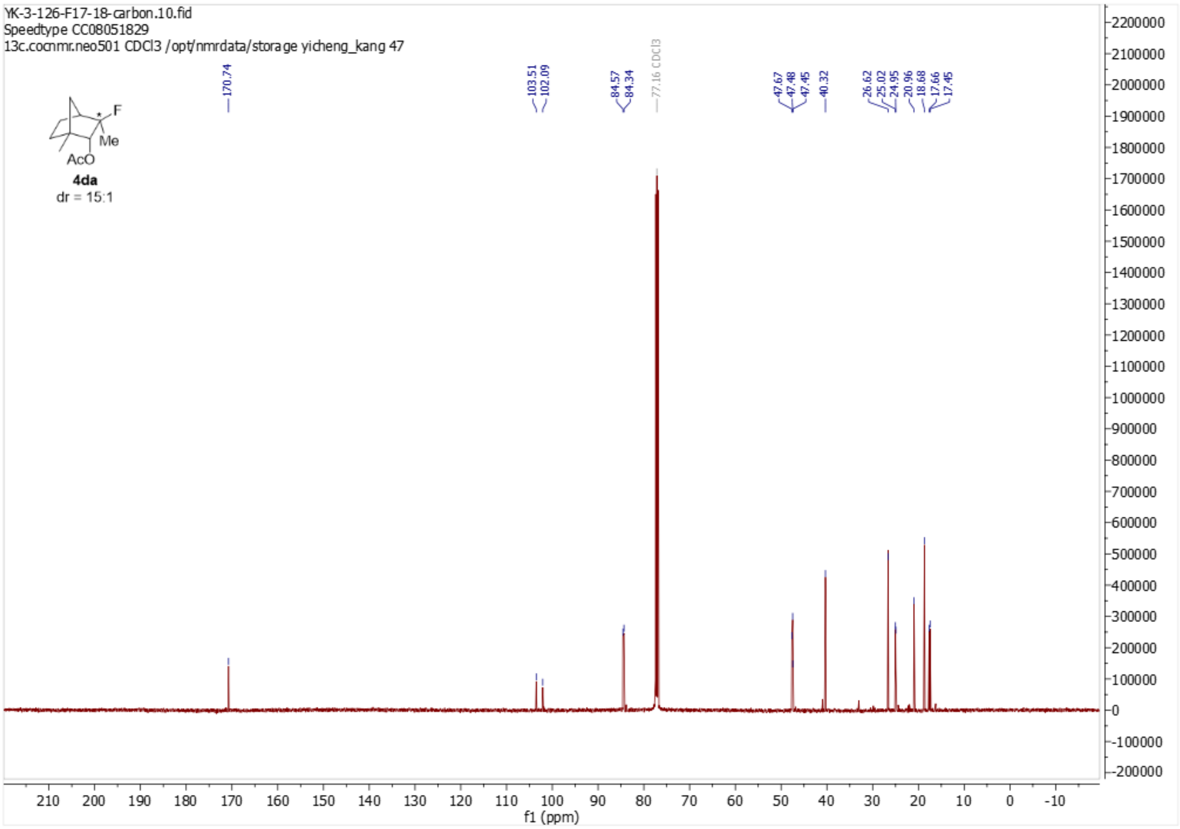

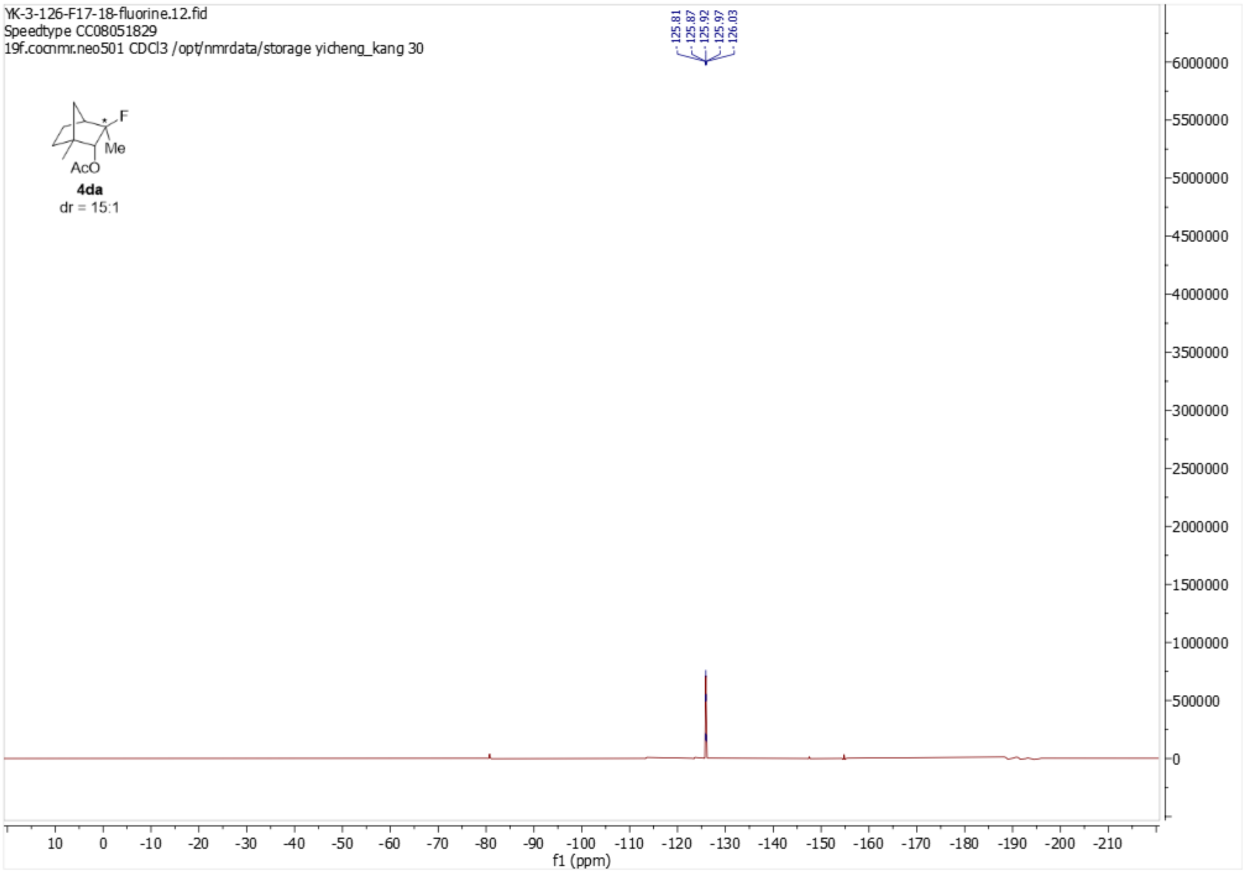
YK-3-126-F17-18-fluorine.12.fid
Speedtype CC08051829
19f.cocnmr.neo501 CDCl3 /opt/nmrdata/storage yicheng_kang 30
125.81
125.87
125.92
125.97
126.03
F
Me
AcO
4da
dr = 15:1
f1 (ppm)

Compound 4af:

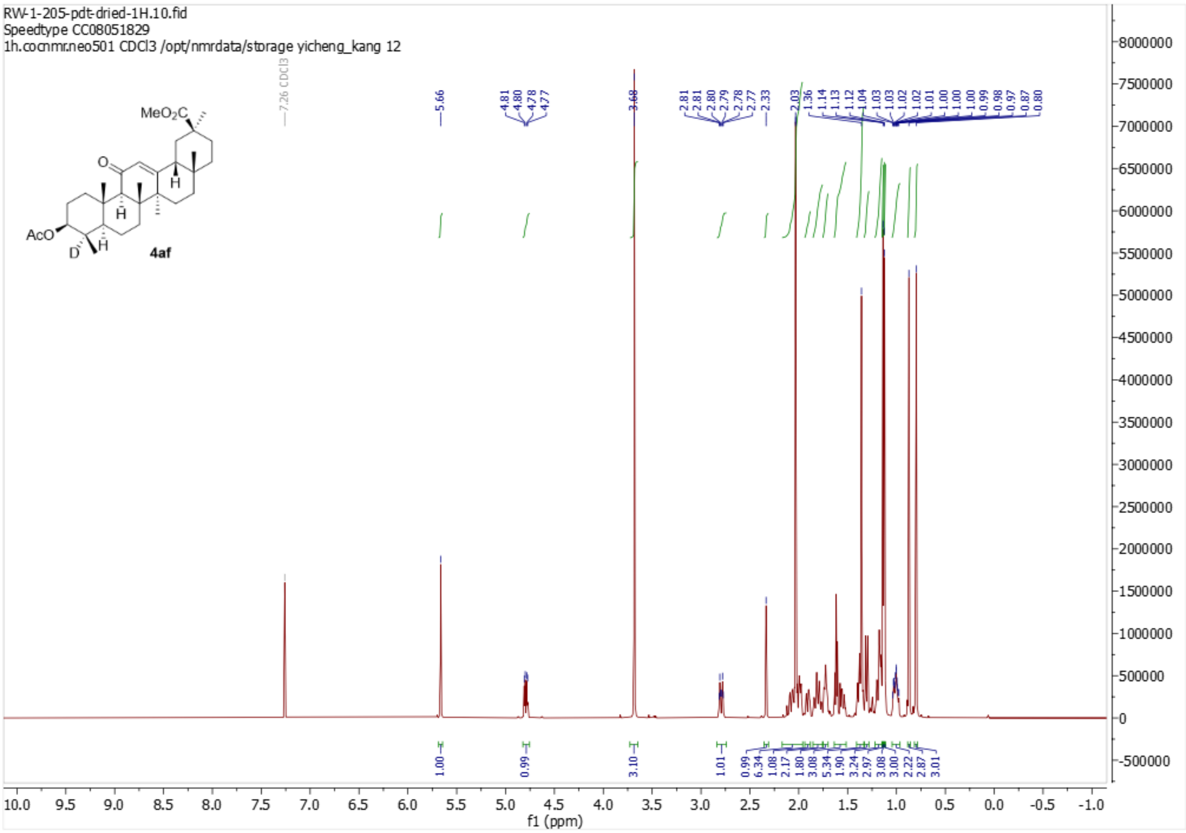

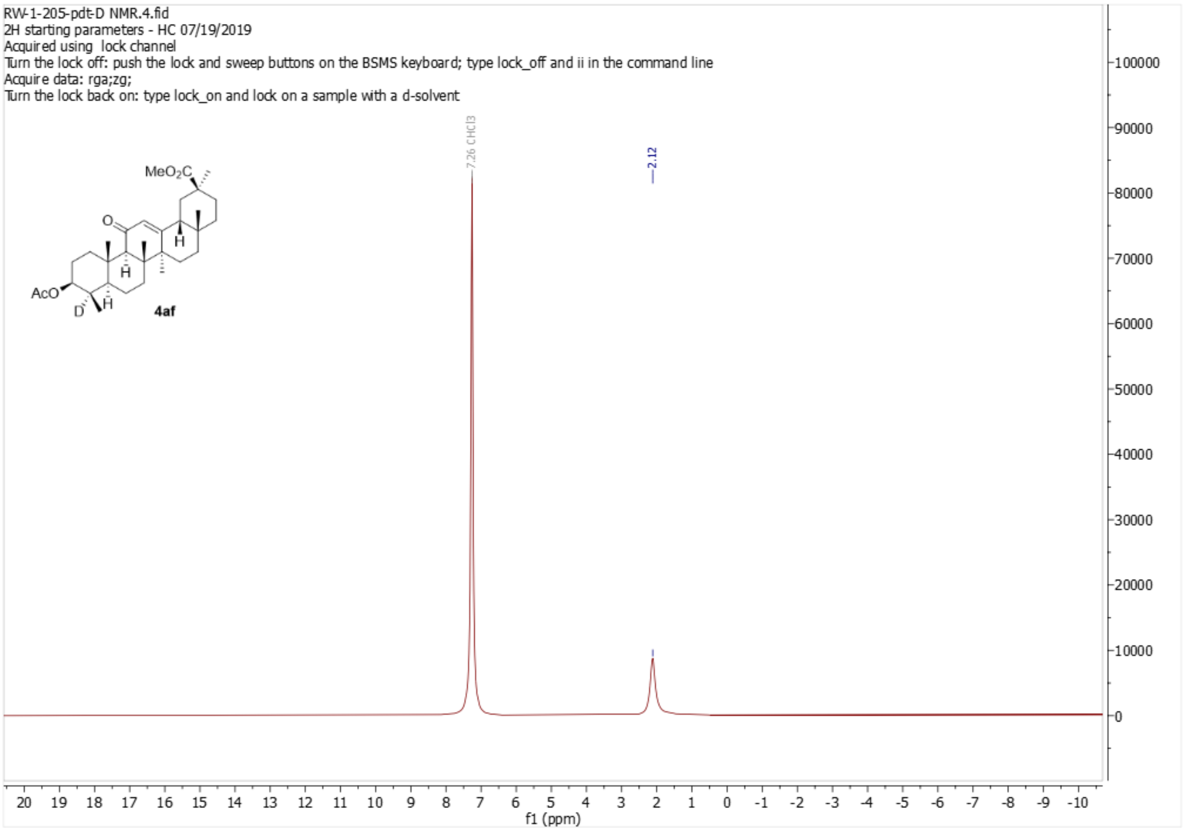

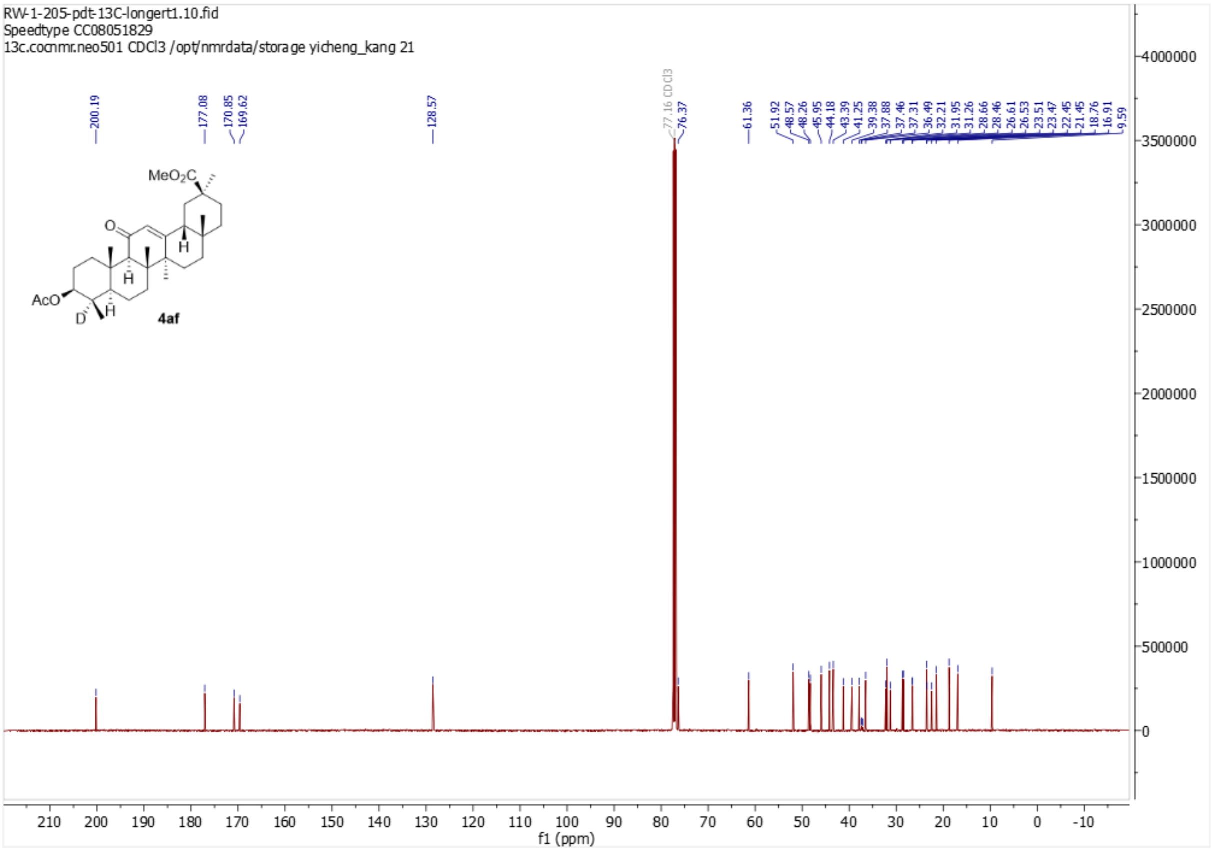
RW-1-205-pdt-13C-longert1.10.fid
Speedtype CC08051829
13c.cocnmr.neo501 CDCl3 /opt/nmrdata/storage yicheng_kang 21
200.19
177.08
170.85
169.62
128.57
77.16 CDCl3
76.37
61.36
51.92
48.57
48.26
45.95
44.18
43.39
41.25
39.38
37.88
37.46
37.31
36.49
32.21
31.95
31.26
28.66
28.46
26.61
26.53
23.51
23.47
22.45
21.45
18.76
16.91
9.59
MeO2C
O
H
H
AcO
D
4af
f1 (ppm)

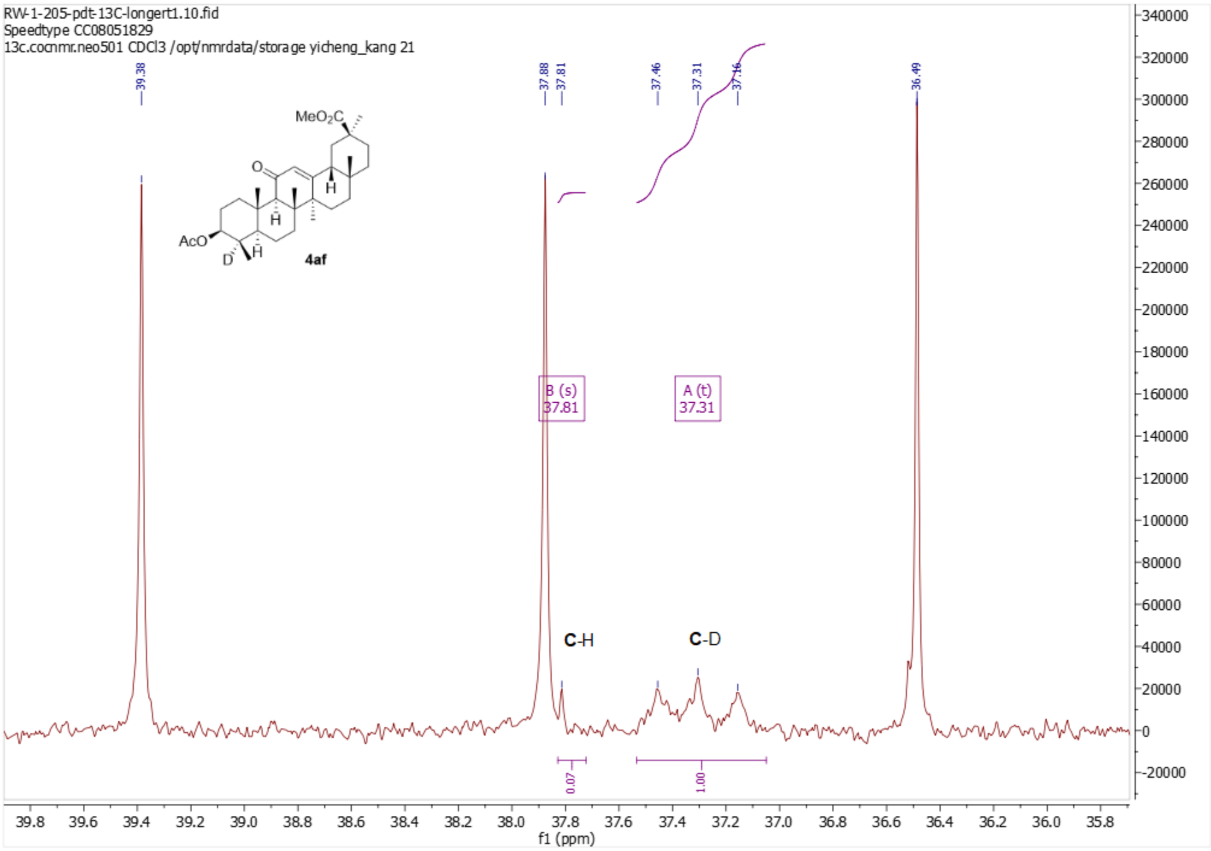
RW-1-205-pdt-13C-longert1.10.fid
Speedtype CC08051829
13c.cocnmr.neo501 CDCl3 /opt/nmrdata/storage yicheng_kang 21
39.38
37.88
37.81
37.46
37.31
37.16
36.49
MeO2C
O
H
H
AcO
D
4af
B (s)
37.81
A (t)
37.31
C-H
C-D
0.07
1.00
f1 (ppm)

Compound 4be:

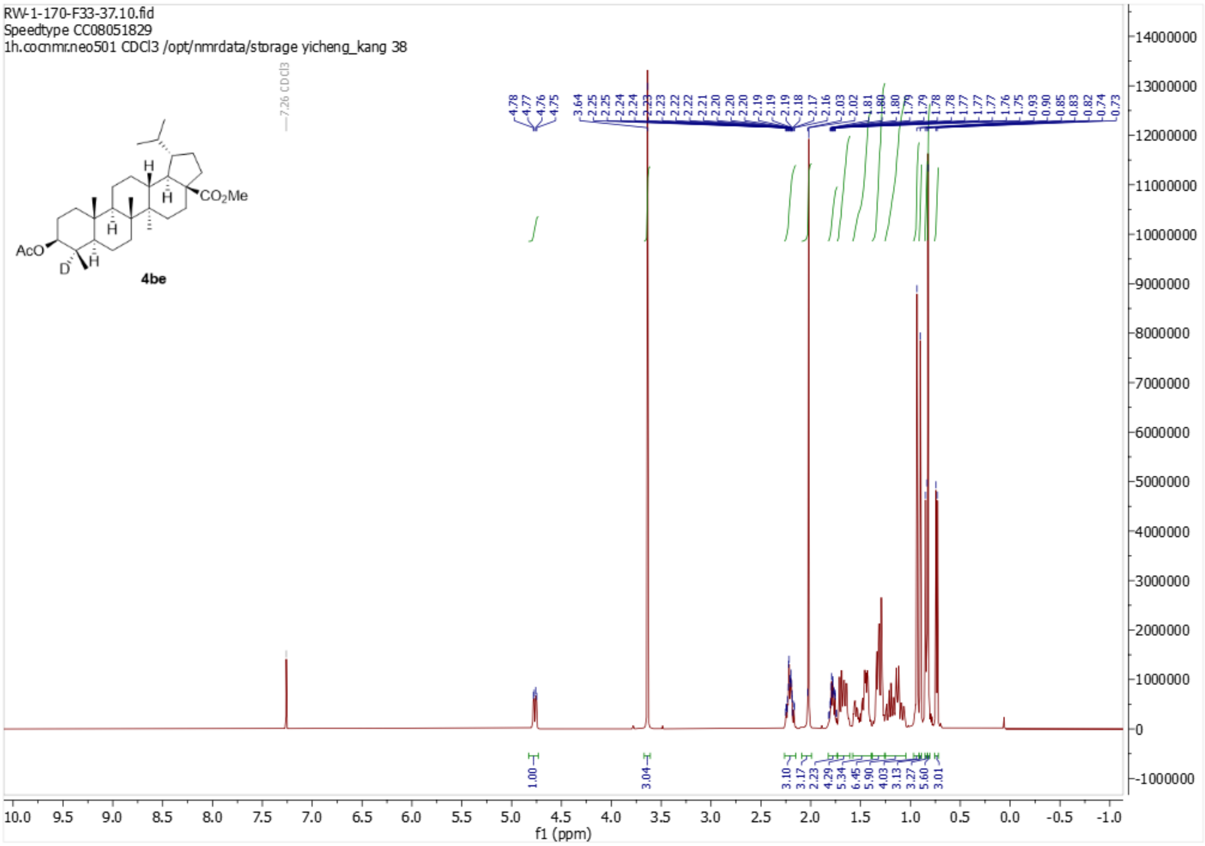

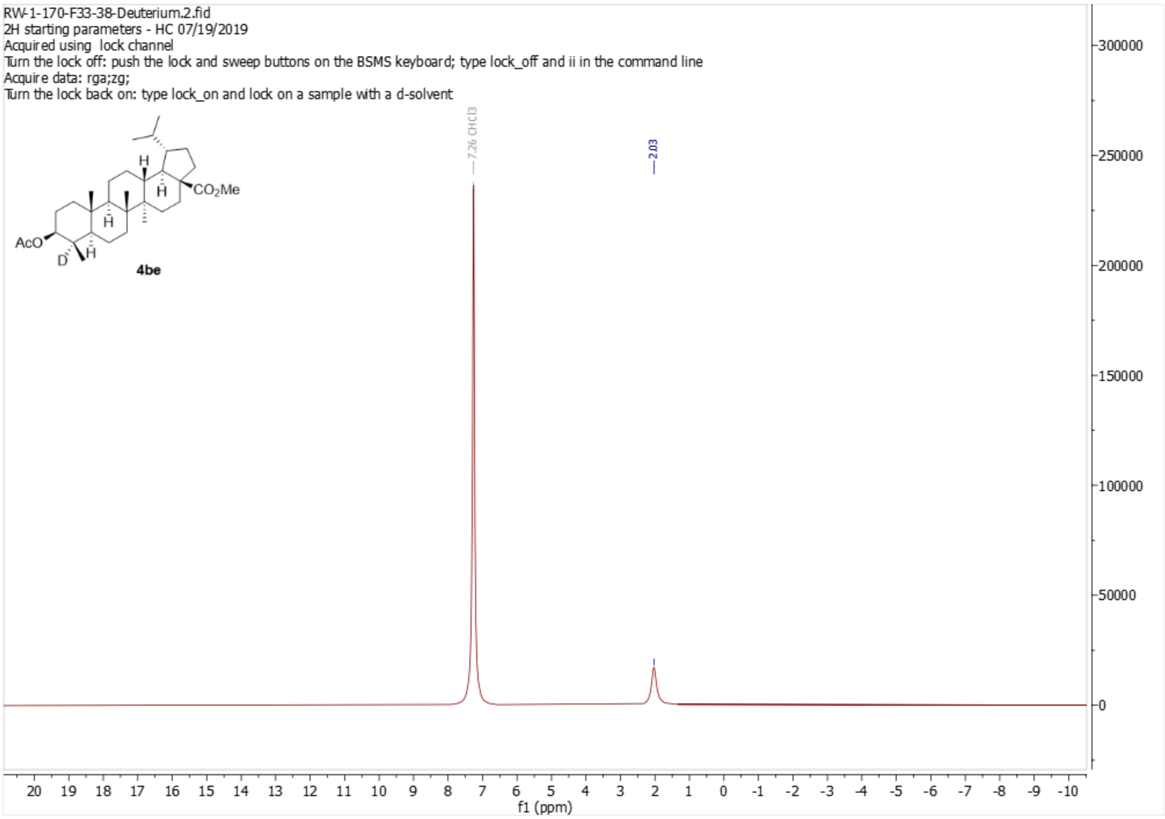

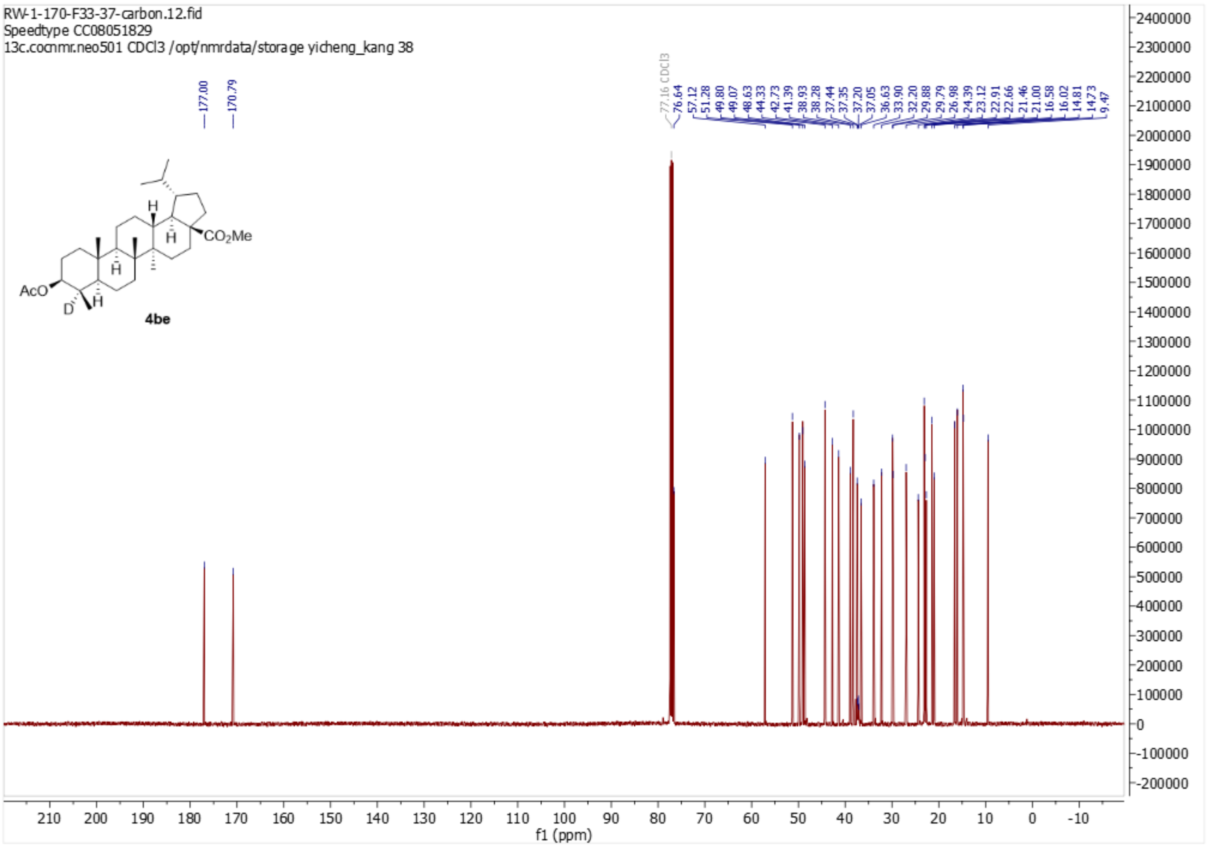
RW-1-170-F33-37-carbon.12.fid
Speedtype CC08051829
13c.cocnmr.neo501 CDCl3 /opt/nmrdata/storage yicheng_kang 38
177.00
170.79
77.16 CDCl3
76.64
57.12
51.28
49.80
49.07
48.63
44.33
42.73
41.39
38.93
38.28
37.44
37.35
37.20
37.05
36.63
33.90
32.20
29.88
29.79
26.98
24.39
23.12
22.91
22.66
21.46
21.00
16.58
16.02
14.81
14.73
9.47
H
H
CO2Me
H
AcO
D
H
4be
f1 (ppm)

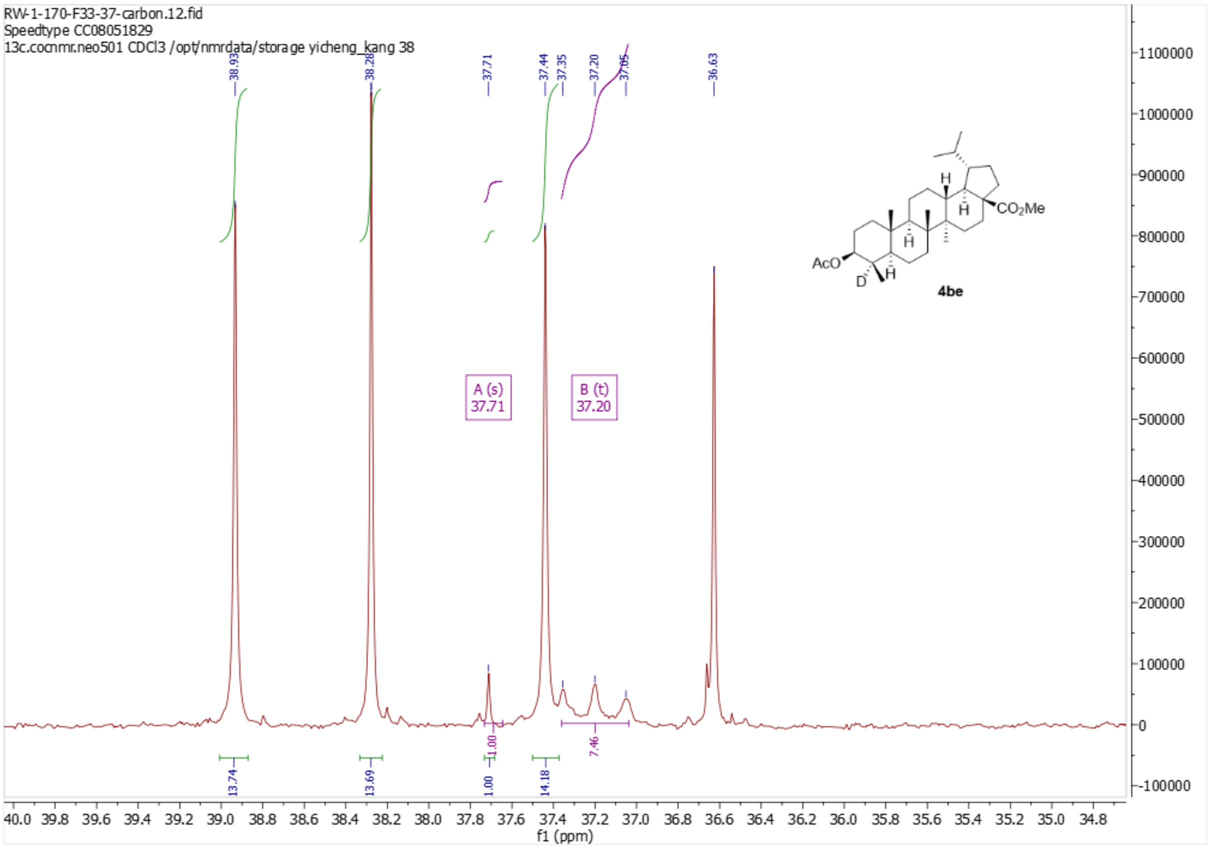
RW-1-170-F33-37-carbon.12.fid
Speedtype CC08051829
13c.cocnmr.neo501 CDCl3 /opt/nmrdata/storage yicheng_kang 38
38.93
38.28
37.71
37.44
37.35
37.20
37.05
36.63
A (s)
37.71
B (t)
37.20
13.74
13.69
1.00
1.00
14.18
7.46
H
H
CO2Me
H
AcO
D
H
4be
f1 (ppm)

Compound 4ag:

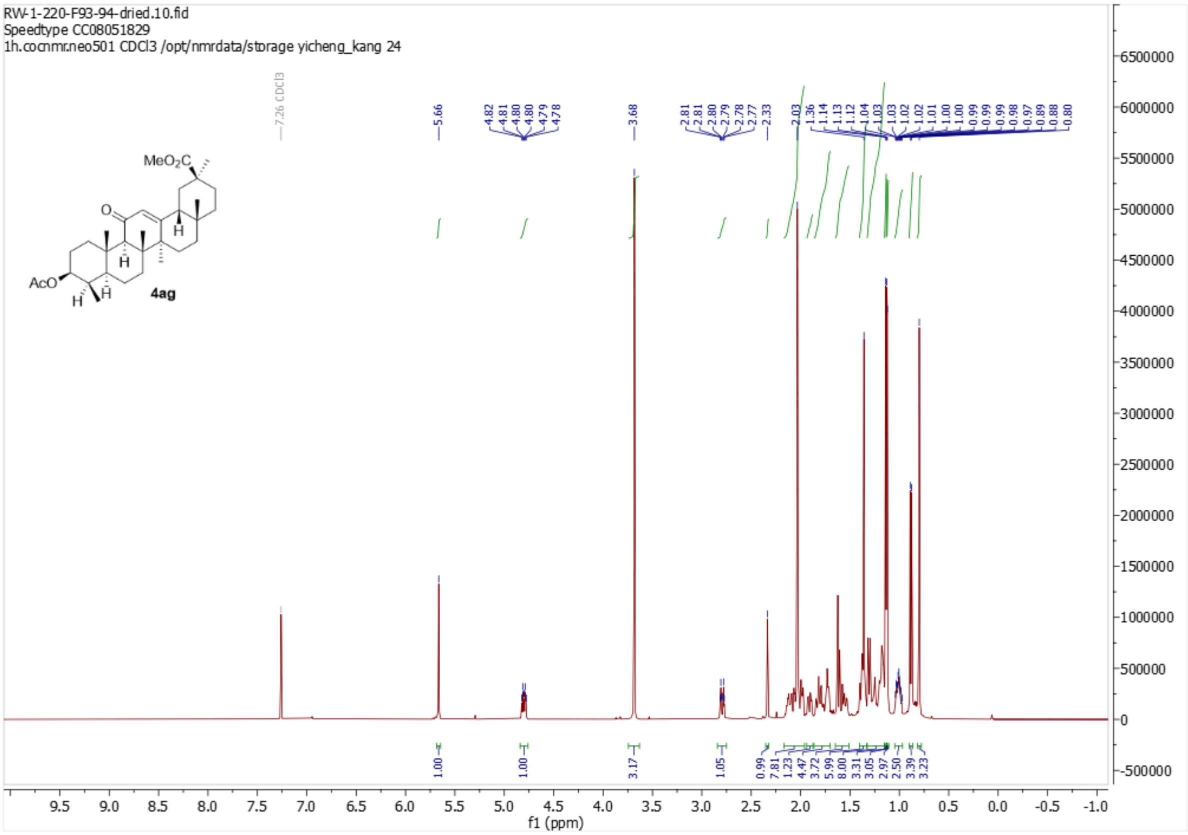

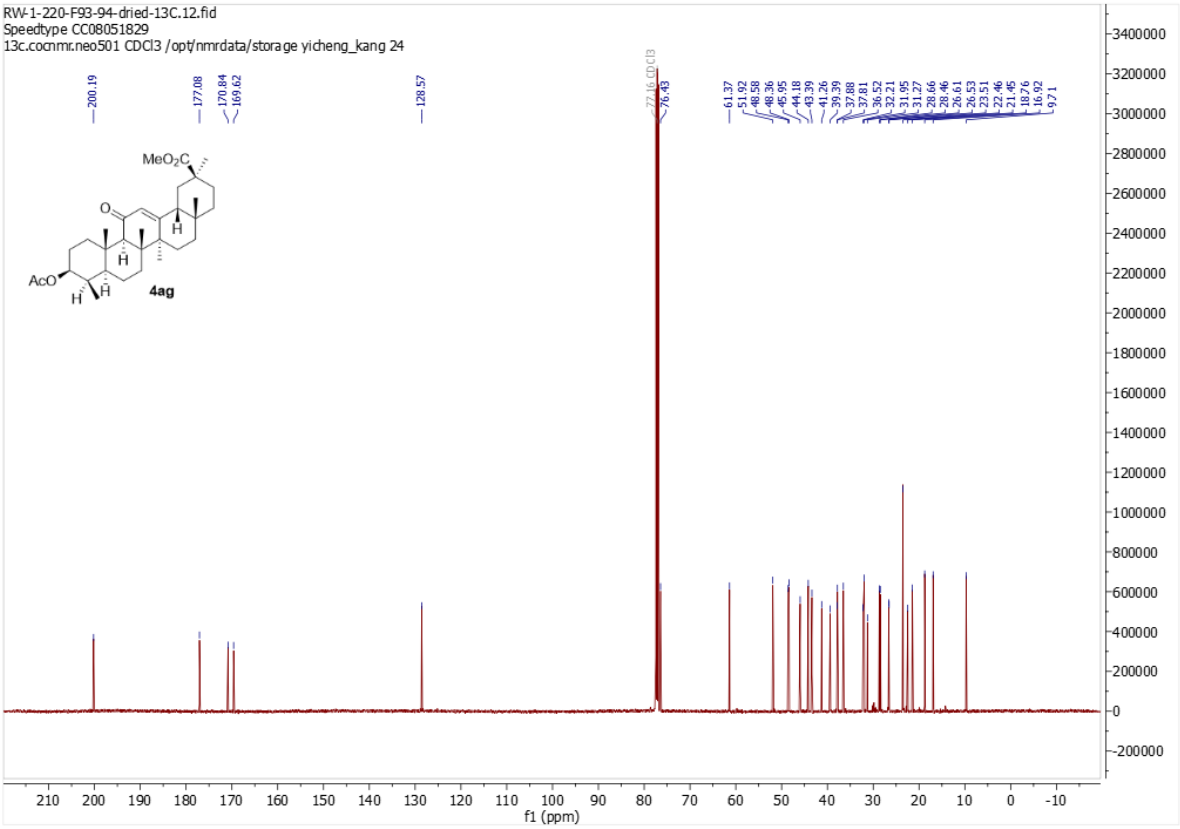

Compound 4bf:

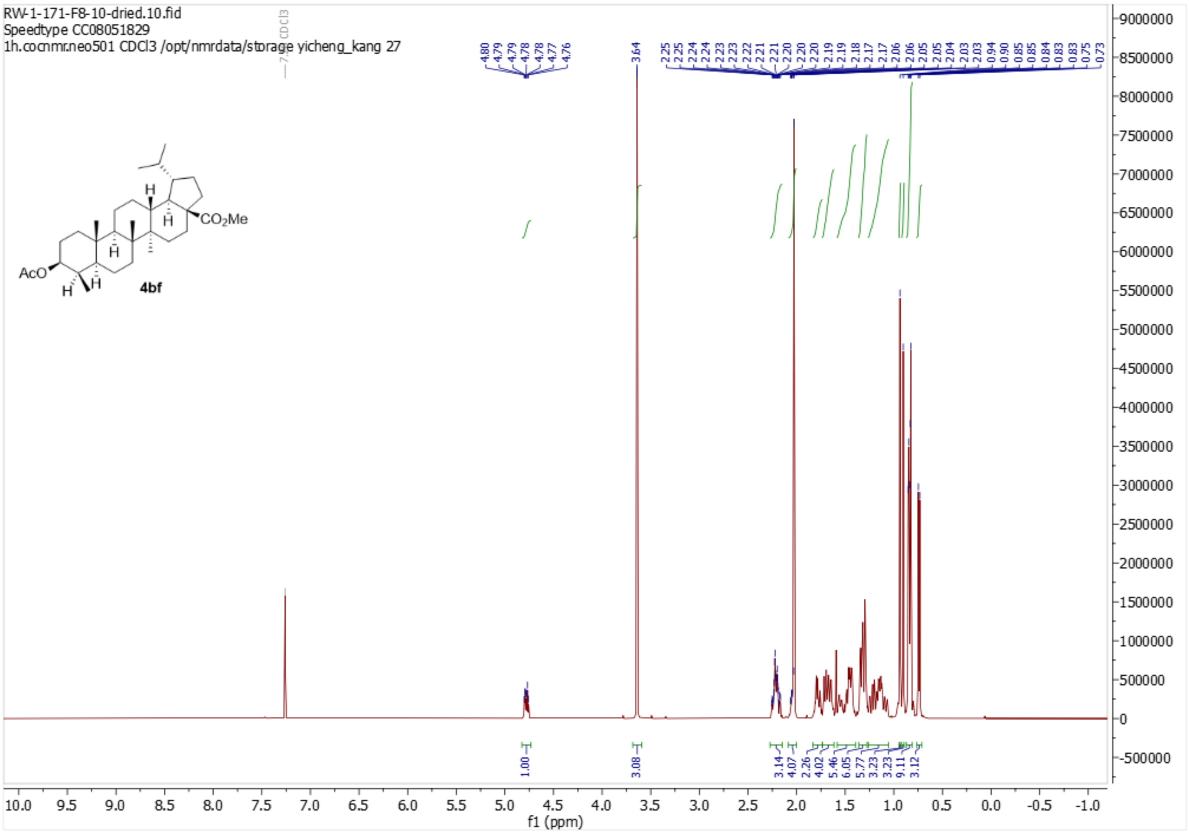

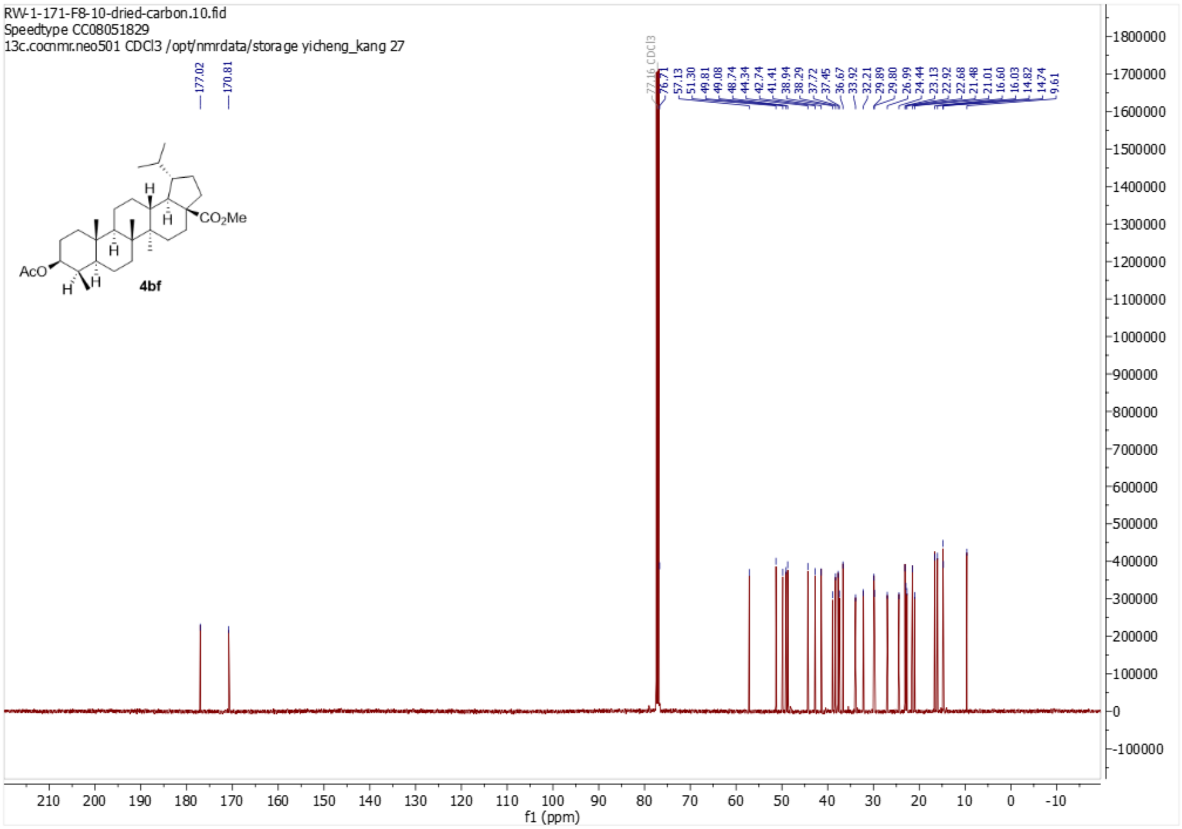

Compound S11:

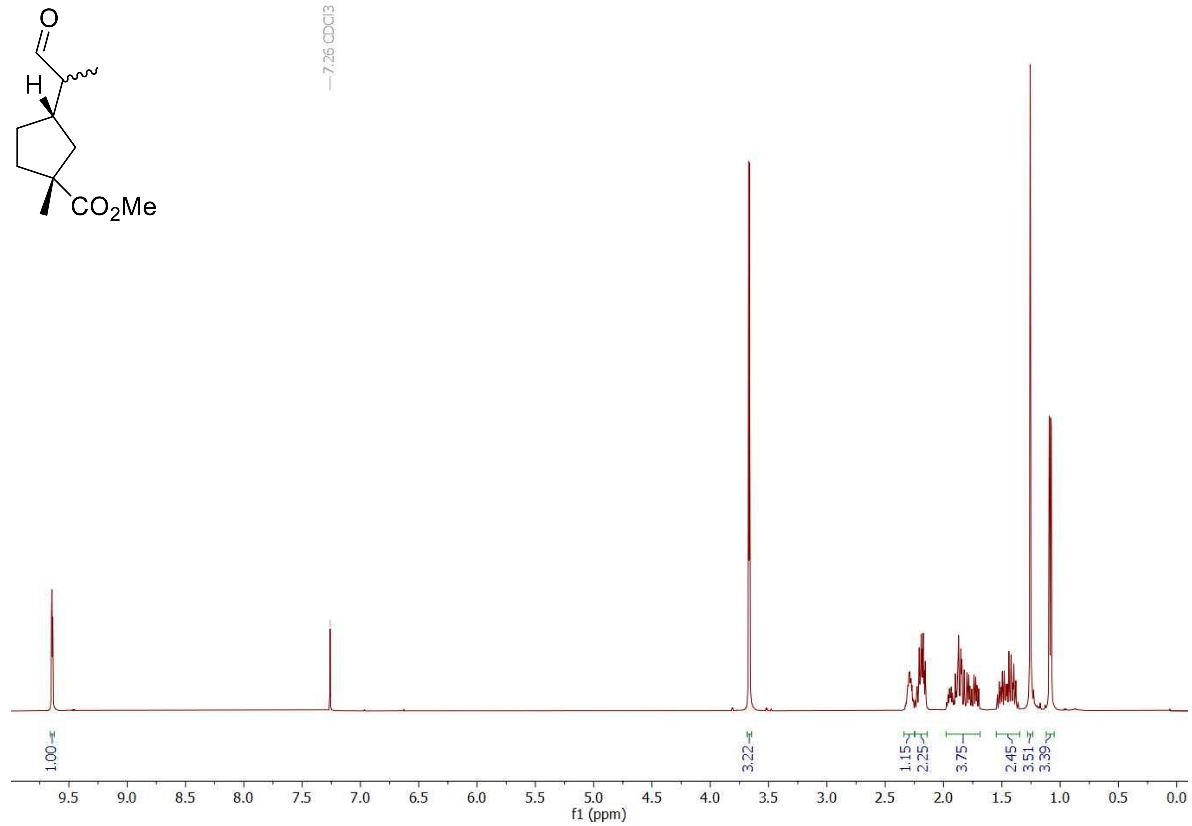

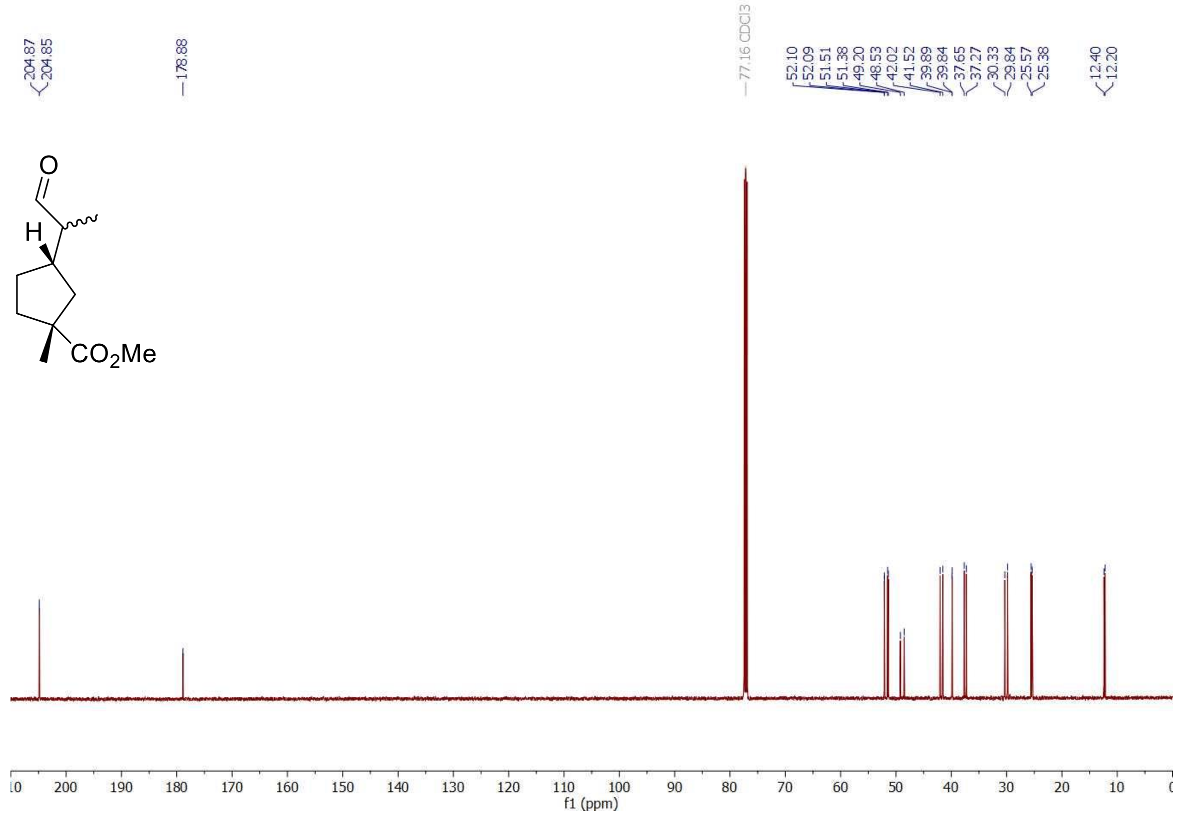

Compound S12:

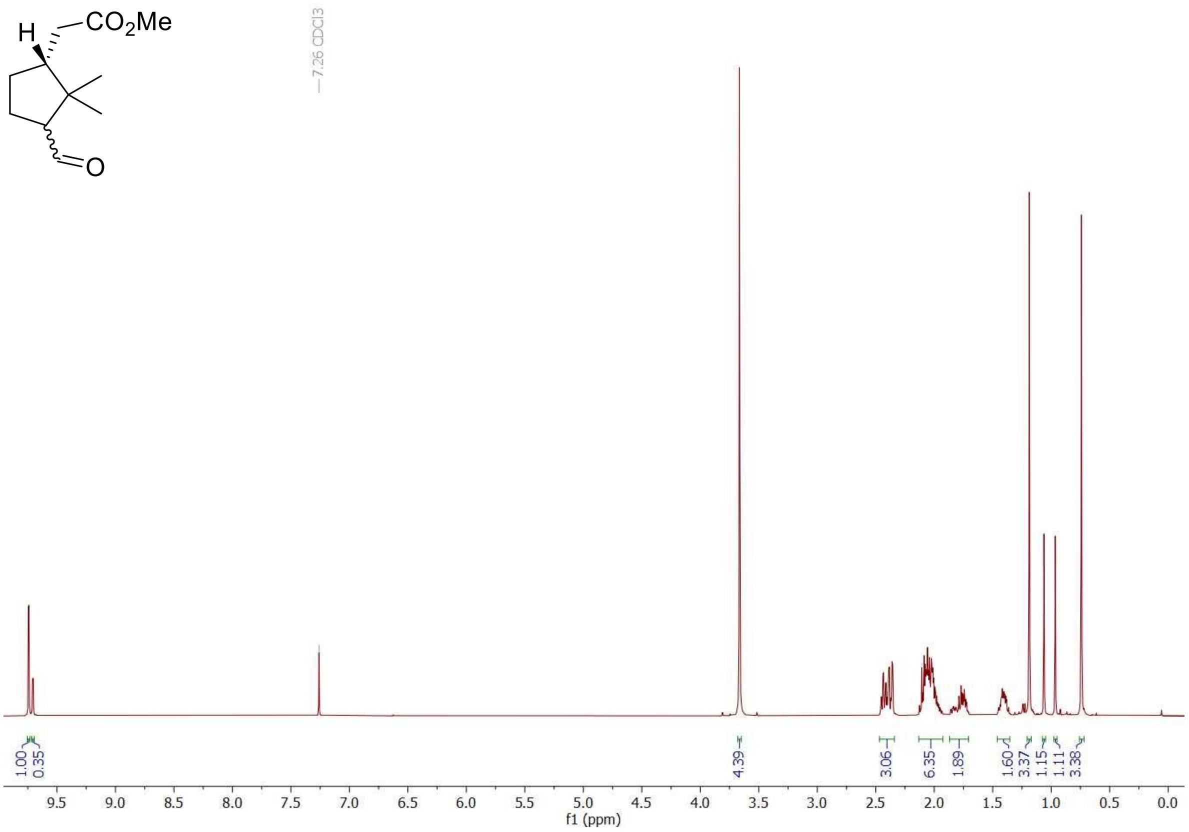

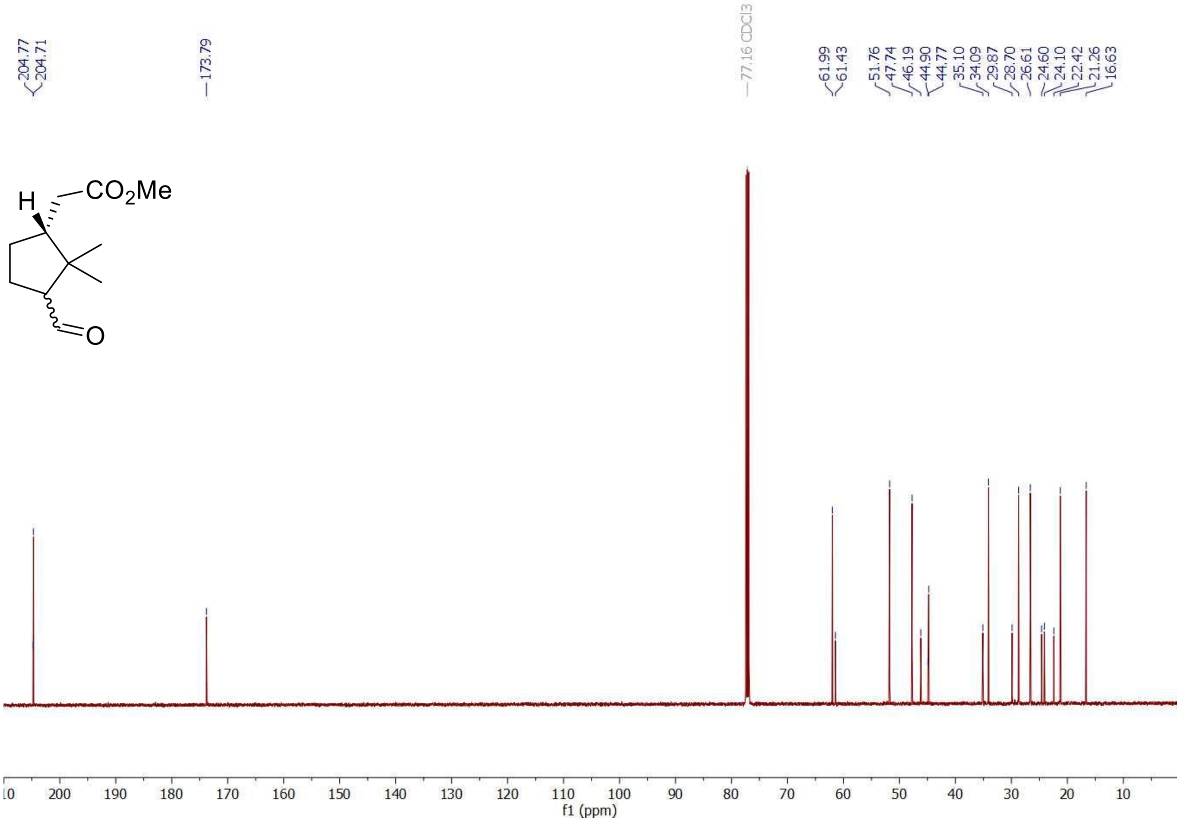

Compound 6a:

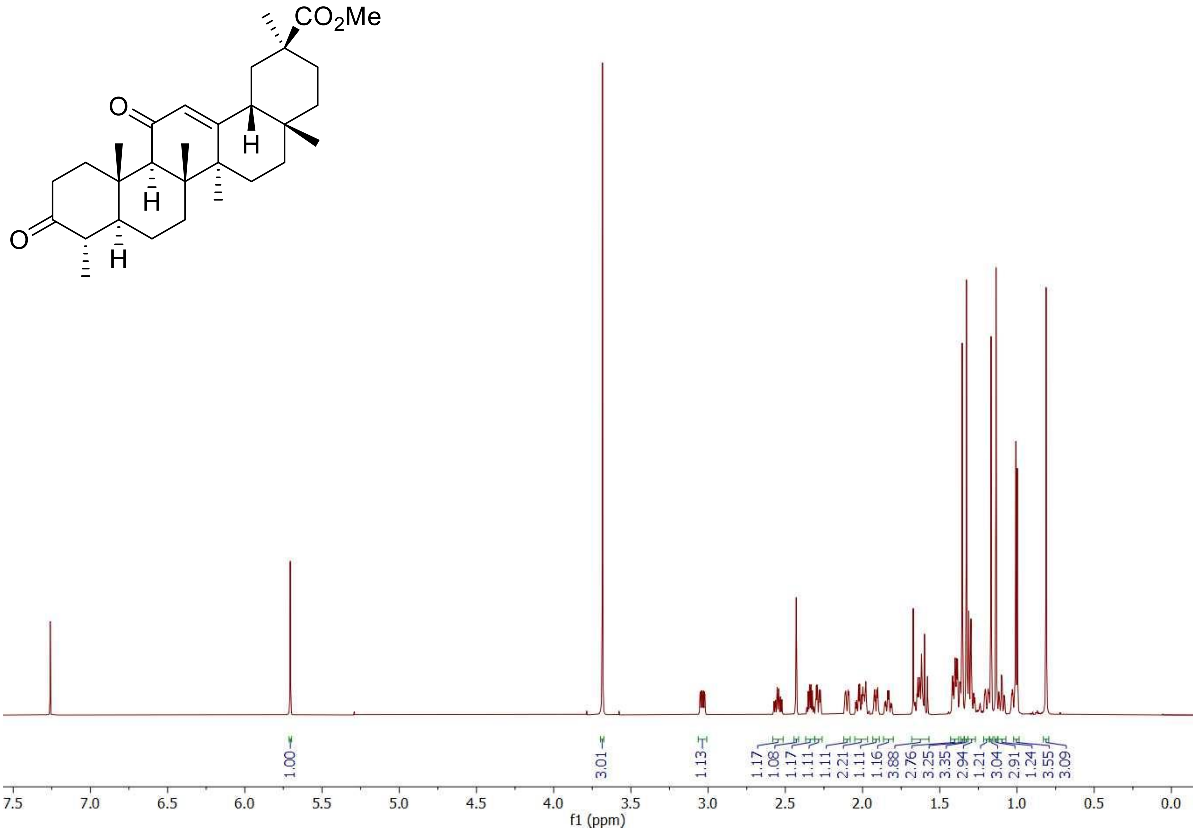

CO2Me
O
H
H
O
H
1.00
3.01
1.13
1.17
1.08
1.17
1.11
1.11
2.21
1.11
1.16
3.88
2.76
3.25
3.35
2.94
1.21
3.04
2.91
1.24
3.55
3.09
7.5
7.0
6.5
6.0
5.5
5.0
4.5
4.0
3.5
3.0
2.5
2.0
1.5
1.0
0.5
0.0
f1 (ppm)

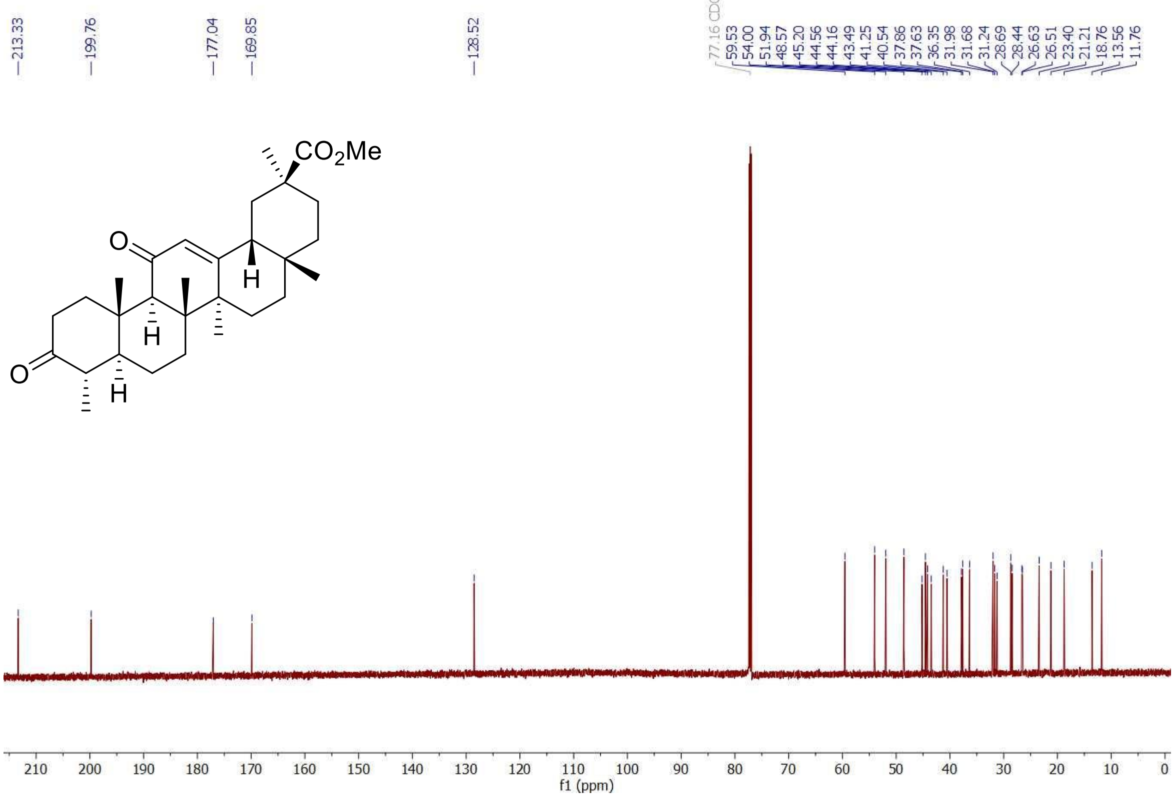

213.33
199.76
177.04
169.85
128.52
77.16 CDCl3
59.53
54.00
51.94
48.57
45.20
44.56
44.16
43.49
41.25
40.54
37.86
37.63
36.35
31.98
31.68
31.24
28.69
28.44
26.63
26.51
23.40
21.21
18.76
13.56
11.76
CO2Me
O
H
H
O
H
210
200
190
180
170
160
150
140
130
120
110
100
90
80
70
60
50
40
30
20
10
0
f1 (ppm)

Compound 6b:

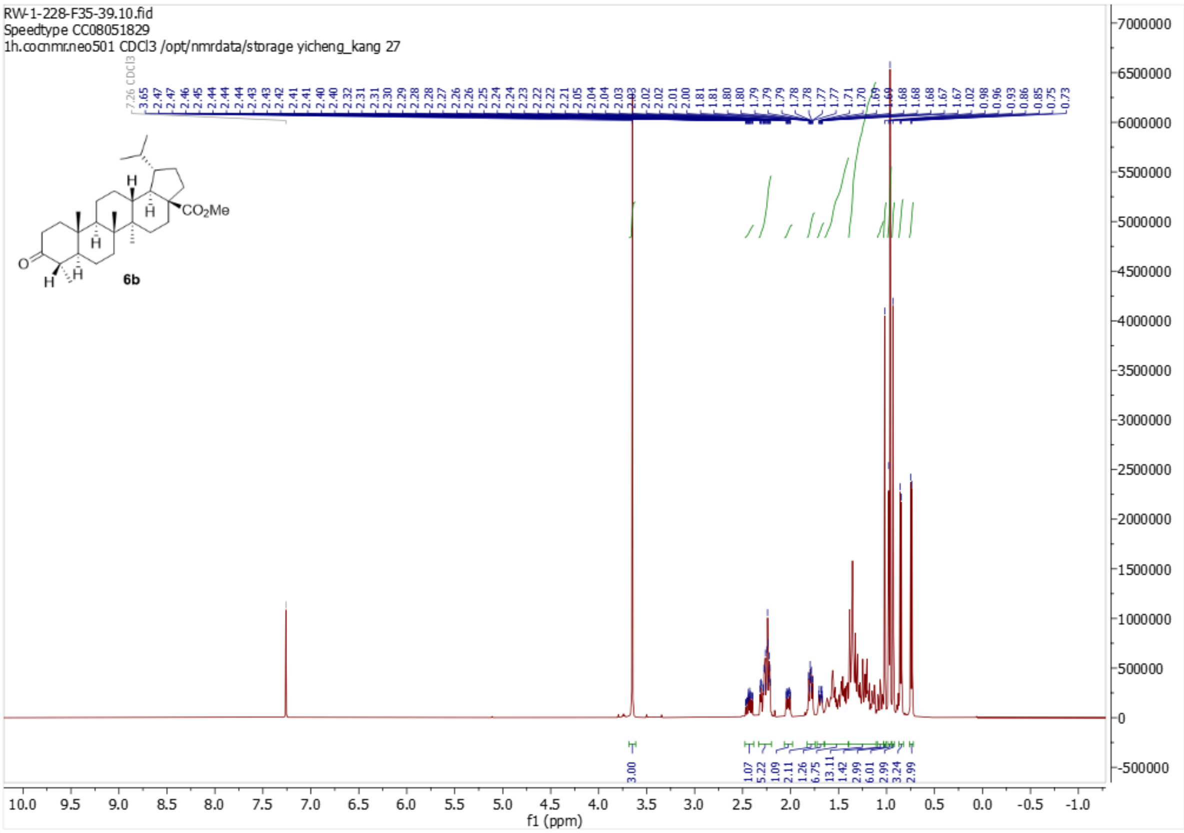
RW-1-228-F35-39.10.fid
Speedtype CC08051829
1h.cocnmr.neo501 CDCl3 /opt/nmrdata/storage yicheng_kang 27
6b
CO2Me
f1 (ppm)

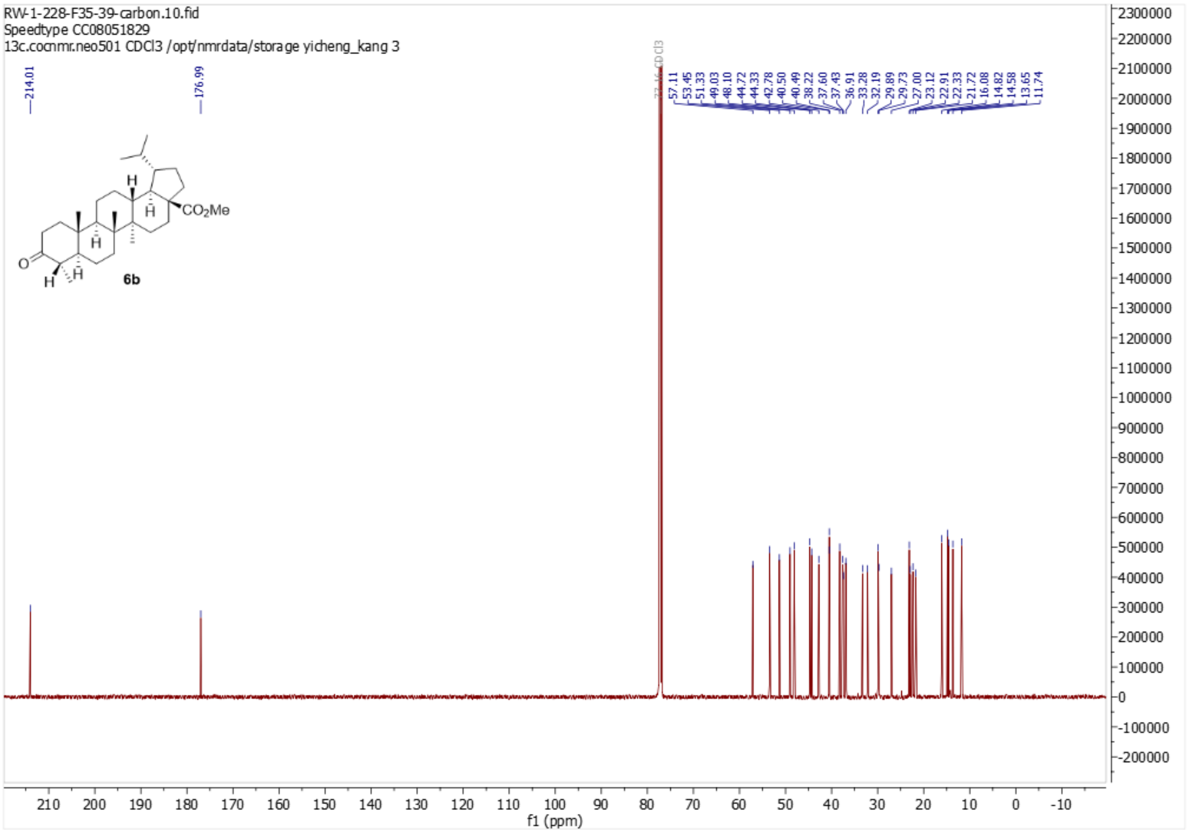
RW-1-228-F35-39-carbon.10.fid
Speedtype CC08051829
13c.cocnmr.neo501 CDCl3 /opt/nmrdata/storage yicheng_kang 3
214.01
176.99
57.11
53.45
51.33
49.03
48.10
44.72
44.33
42.78
40.50
40.49
38.22
37.60
37.43
36.91
33.28
32.19
29.89
29.73
27.00
23.12
22.91
22.33
21.72
16.08
14.82
14.58
13.65
11.74
6b
CO2Me
f1 (ppm)

Compound 6c:

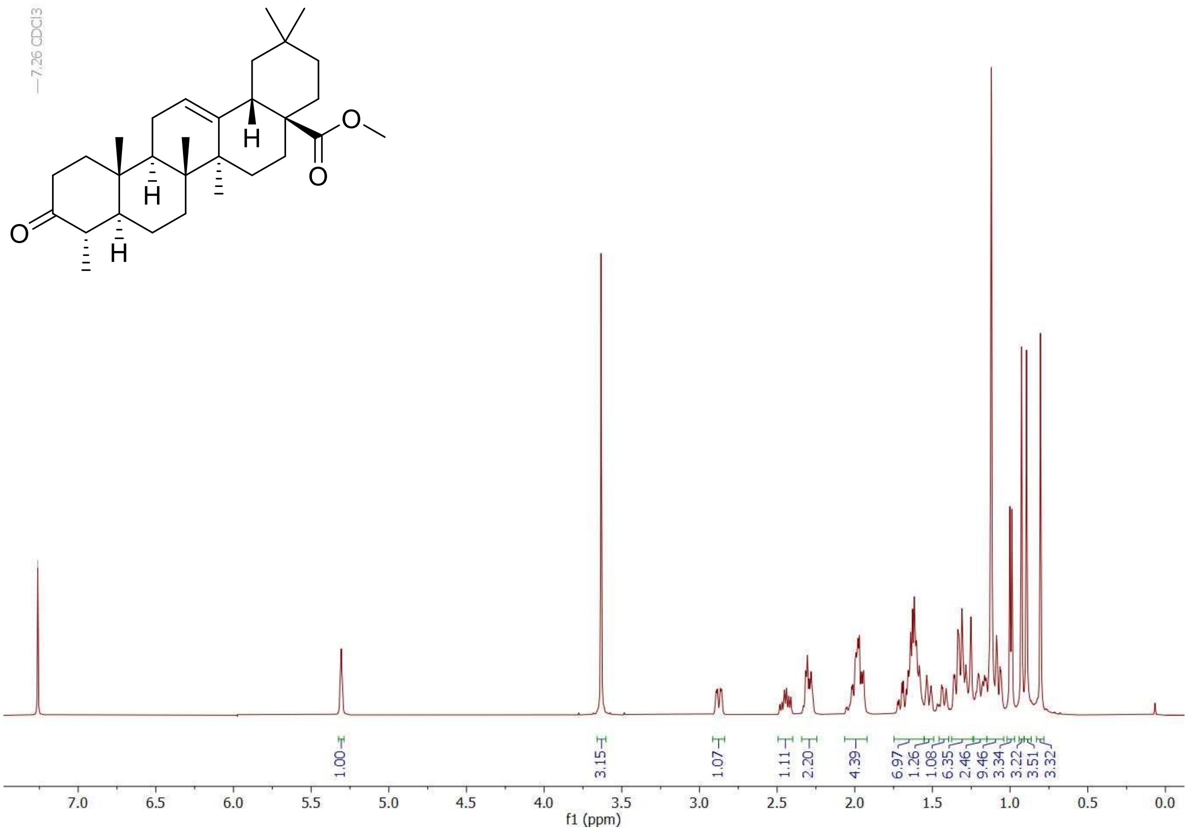

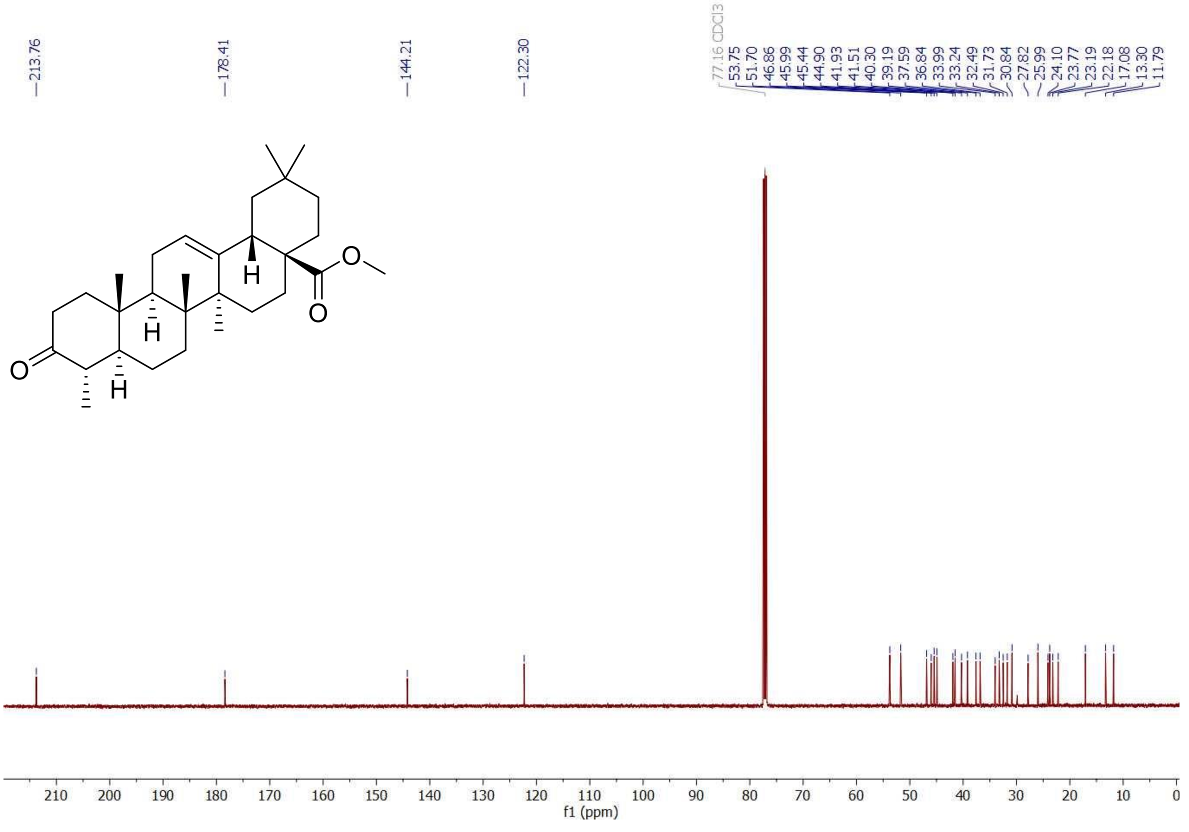

Compound 6d:

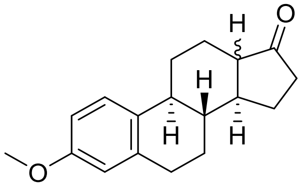

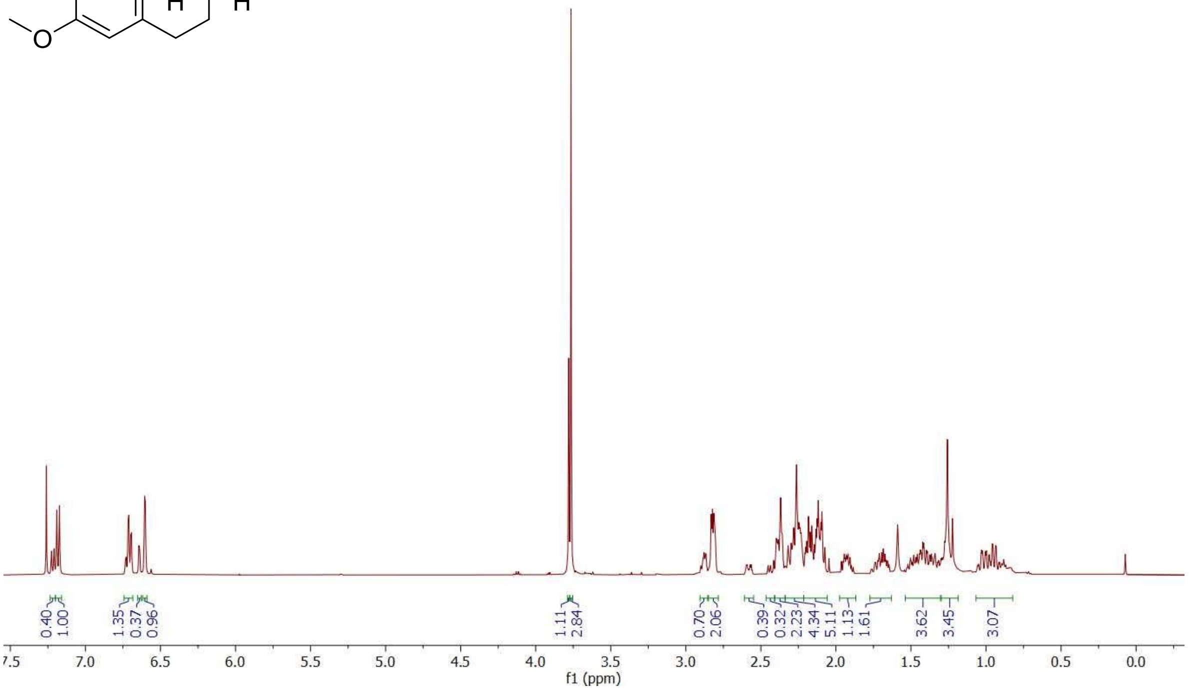

0.40
1.00
1.35
0.37
0.96
1.11
2.84
0.70
2.06
0.39
0.32
2.23
4.34
5.11
1.13
1.61
3.62
3.45
3.07
7.5 7.0 6.5 6.0 5.5 5.0 4.5 4.0 3.5 3.0 2.5 2.0 1.5 1.0 0.5 0.0
f1 (ppm)

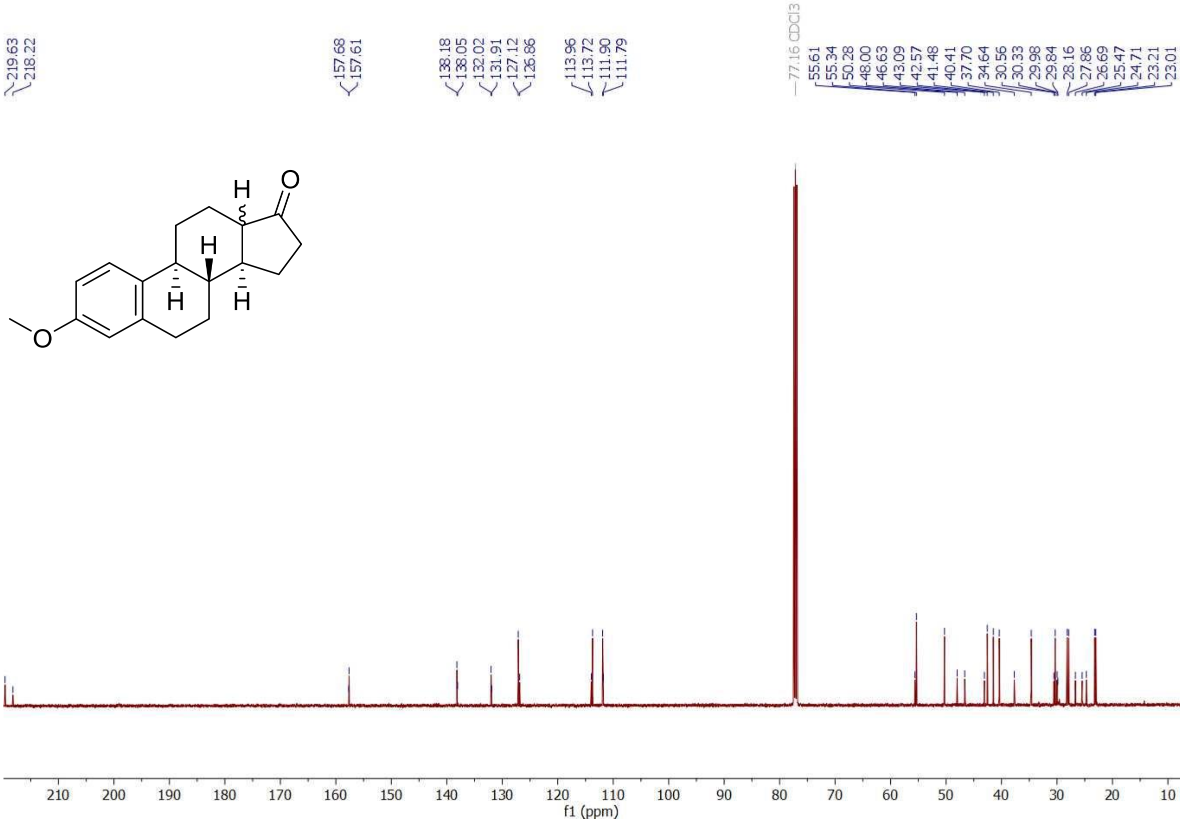

219.63
218.22
157.68
157.61
138.18
138.05
132.02
131.91
127.12
126.86
113.96
113.72
111.90
111.79
77.16 CDCl3
55.61
55.34
50.28
48.00
46.63
43.09
42.57
41.48
40.41
37.70
34.64
30.56
30.33
29.98
29.84
28.16
27.86
26.69
25.47
24.71
23.21
23.01
H
O
H
H
H
O
210 200 190 180 170 160 150 140 130 120 110 100 90 80 70 60 50 40 30 20 10
f1 (ppm)

Compound 7a:

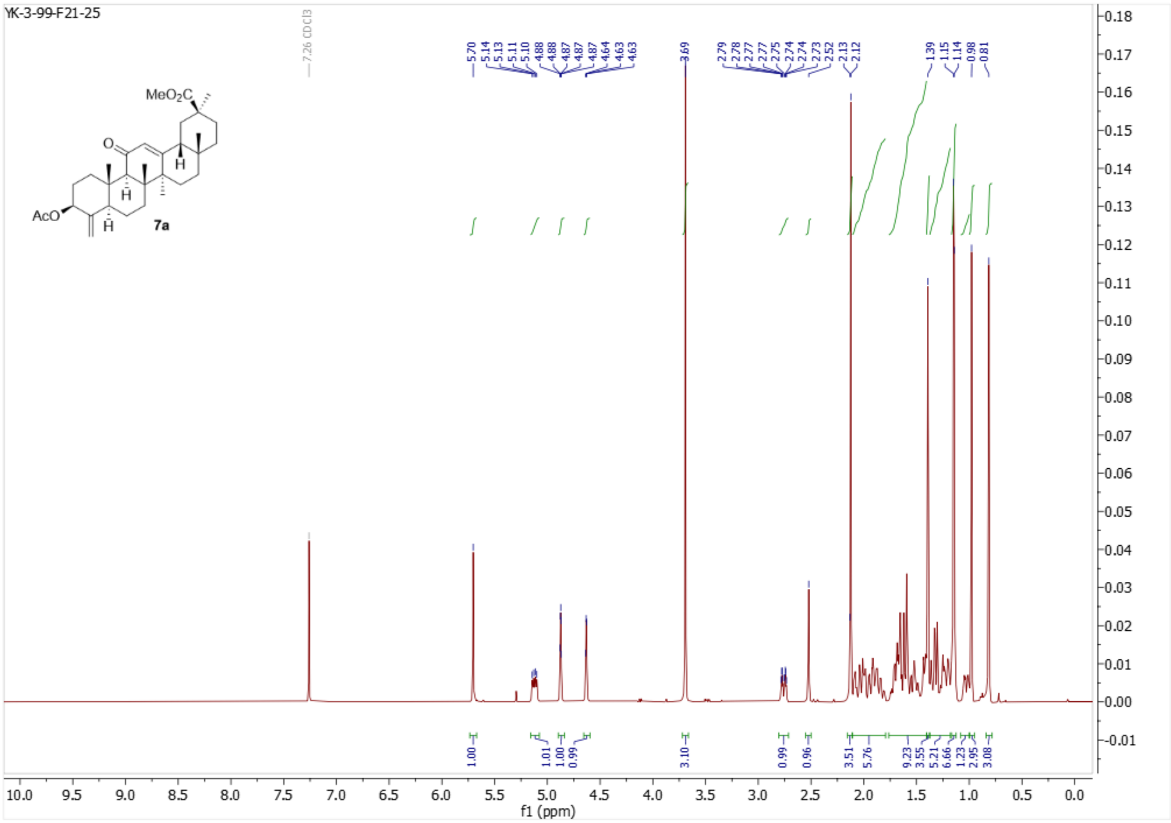

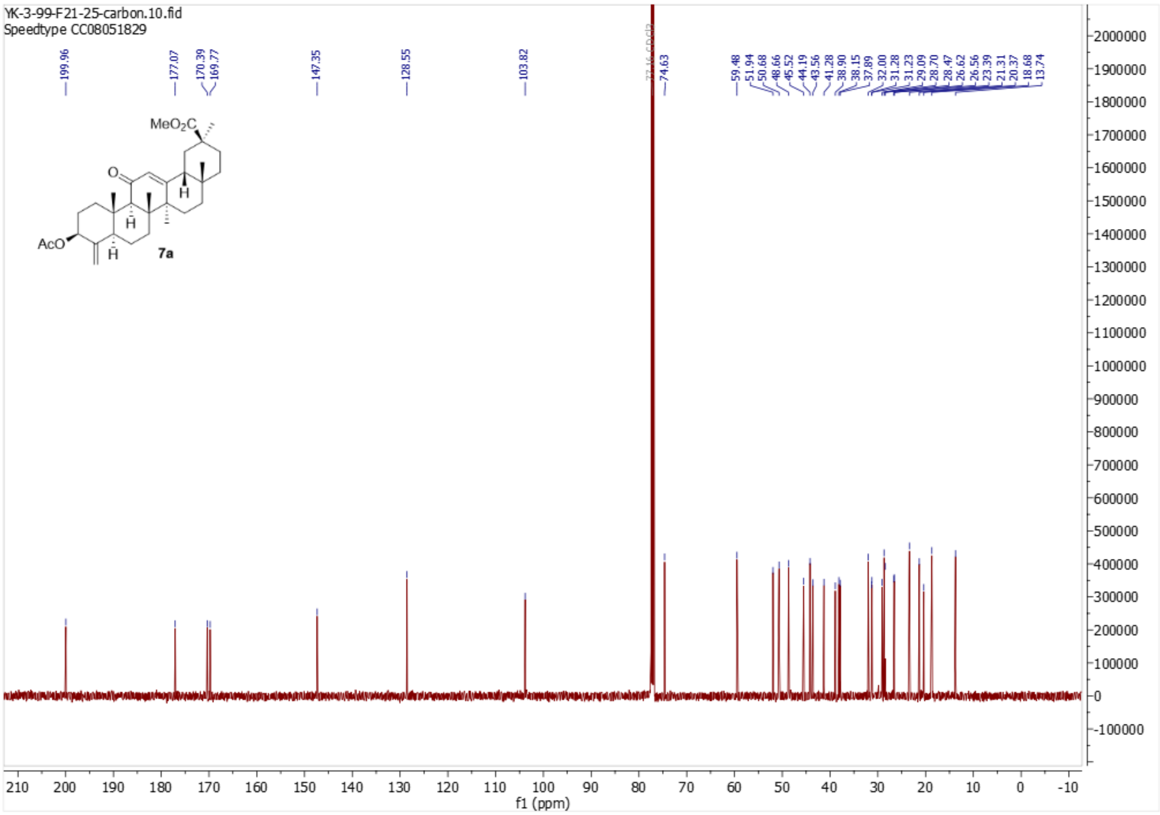

Compound 7b:

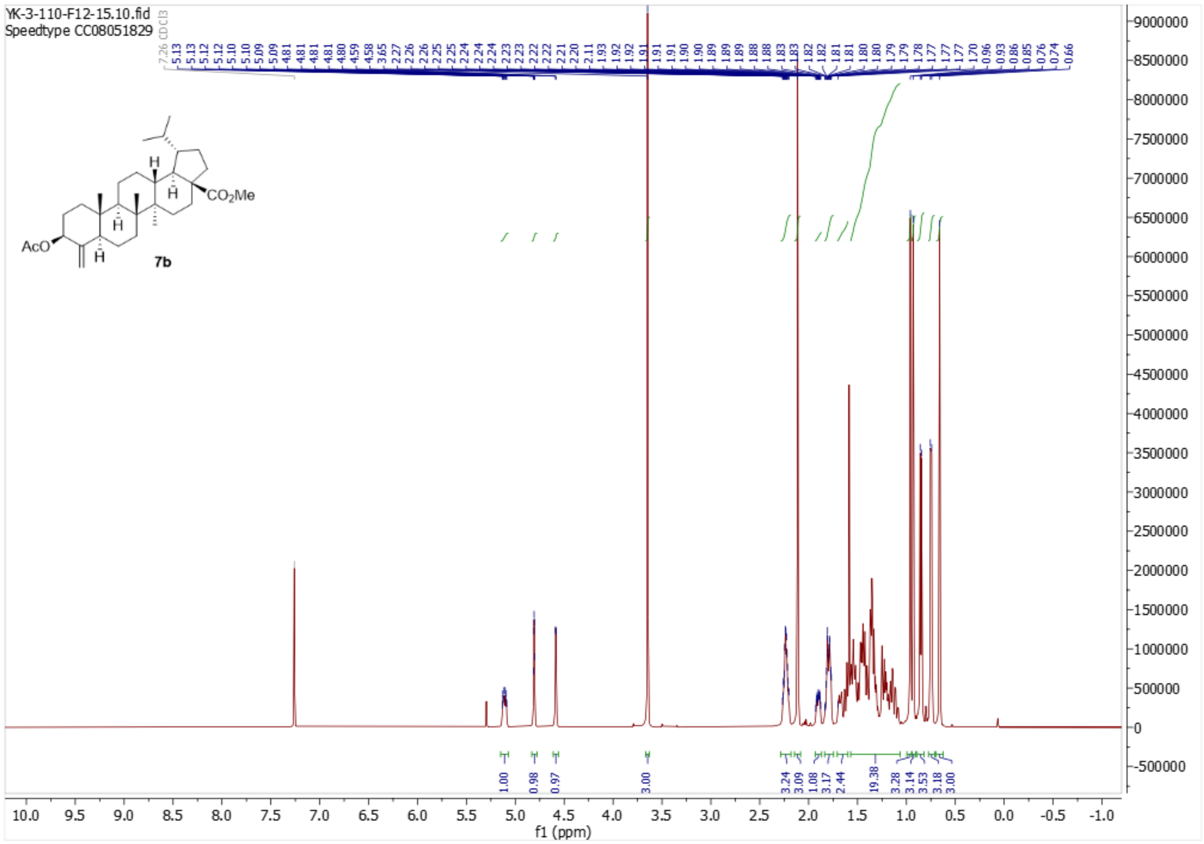

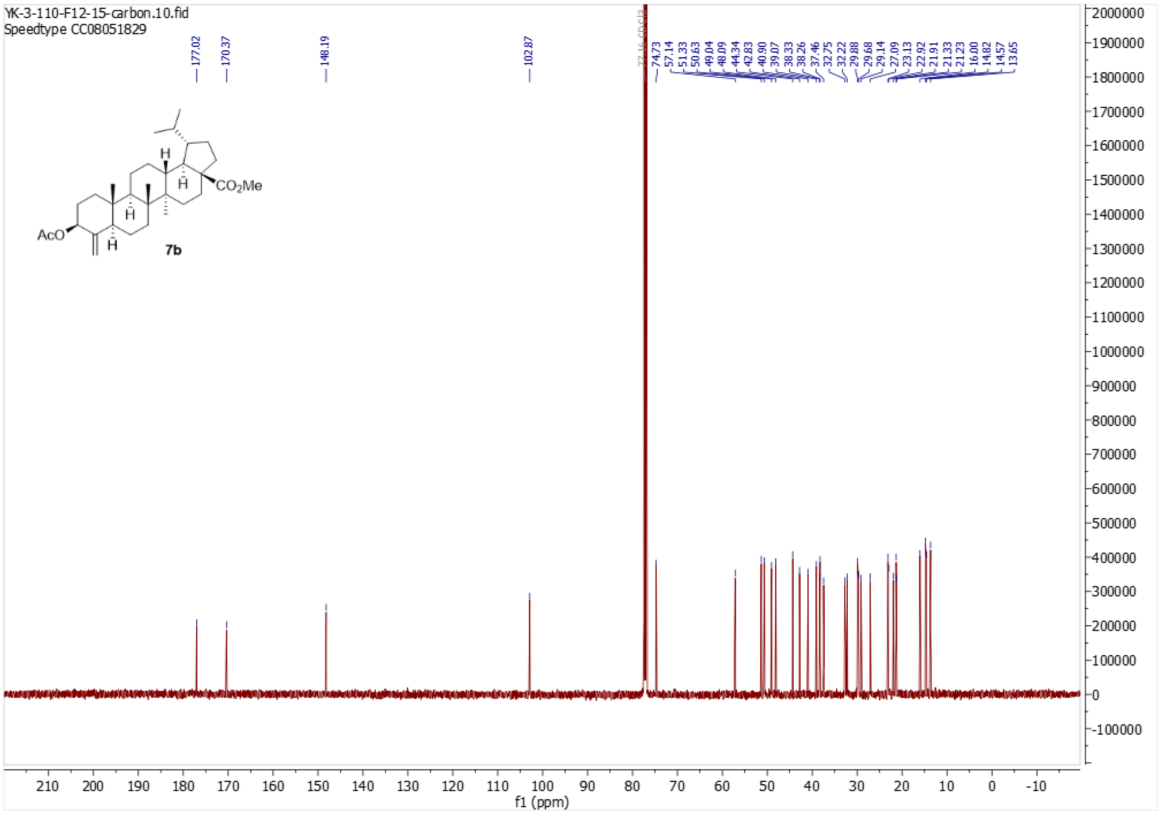

Compound 8b:

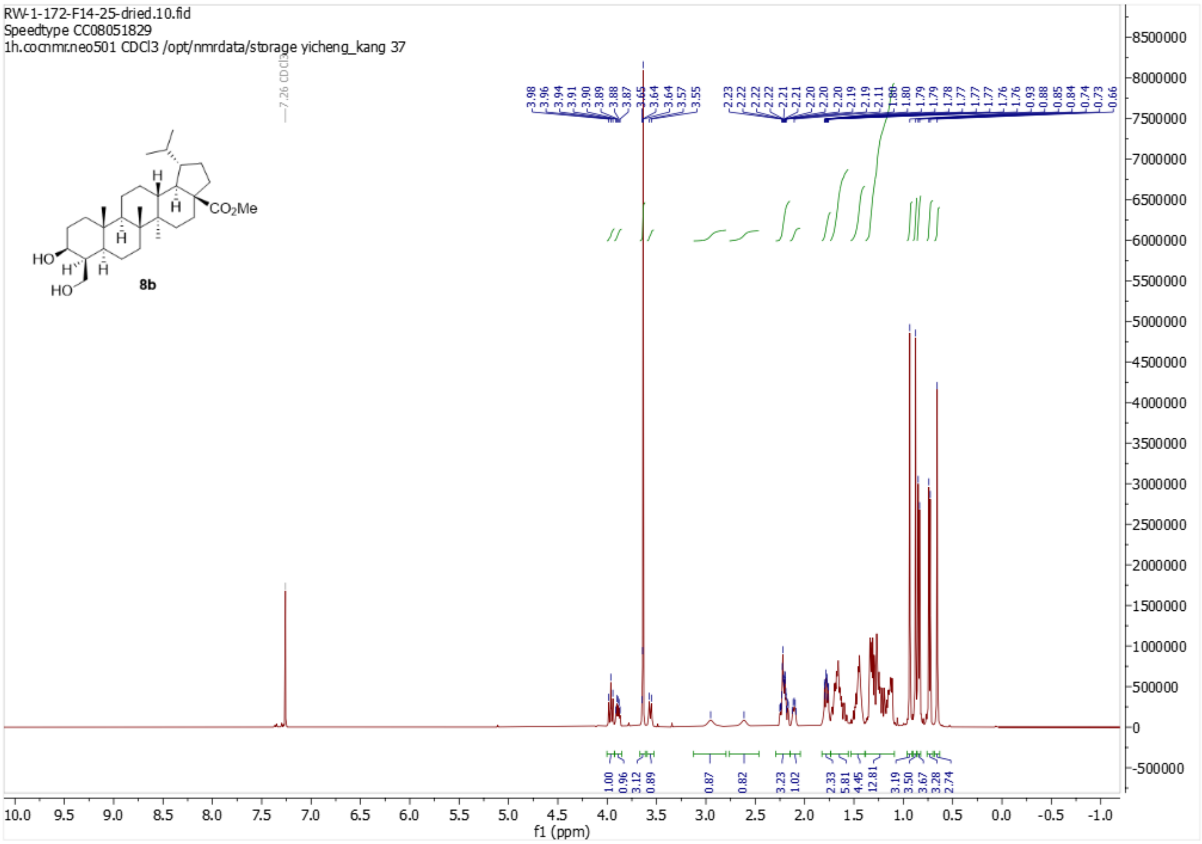

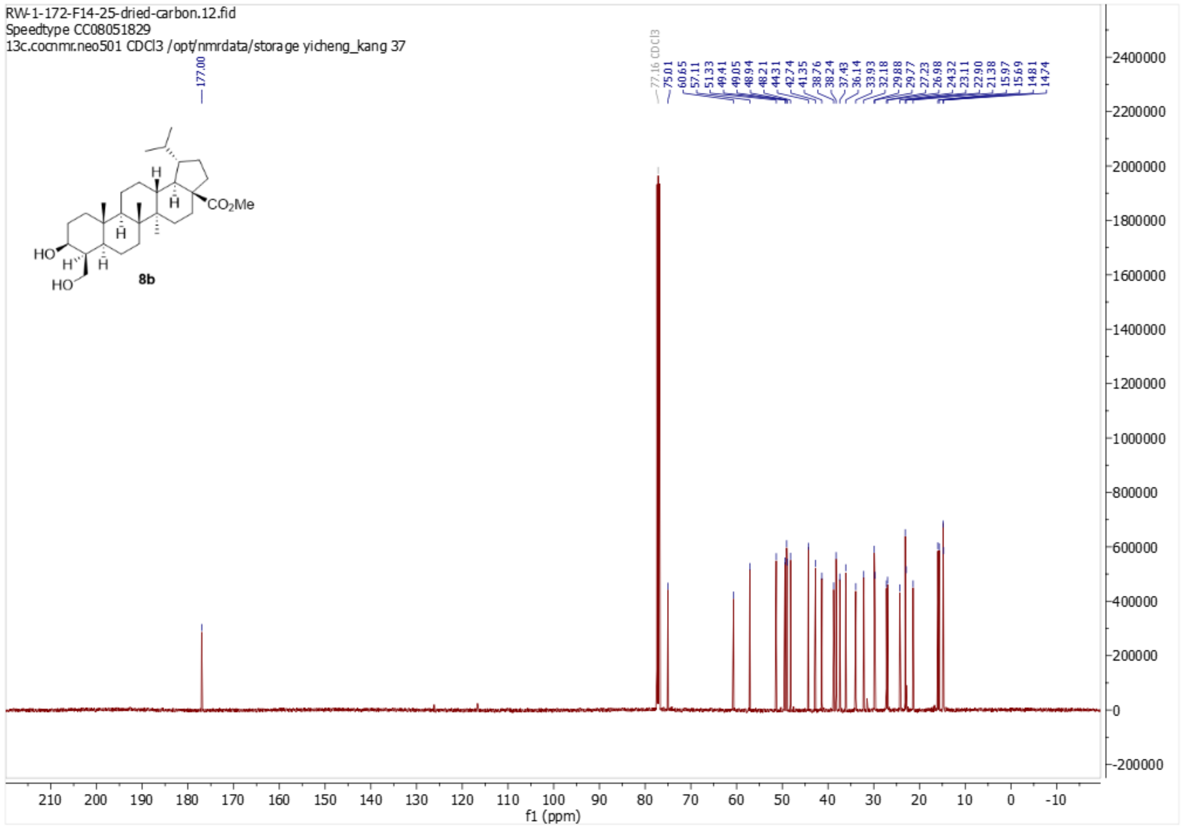

Compound 7c:

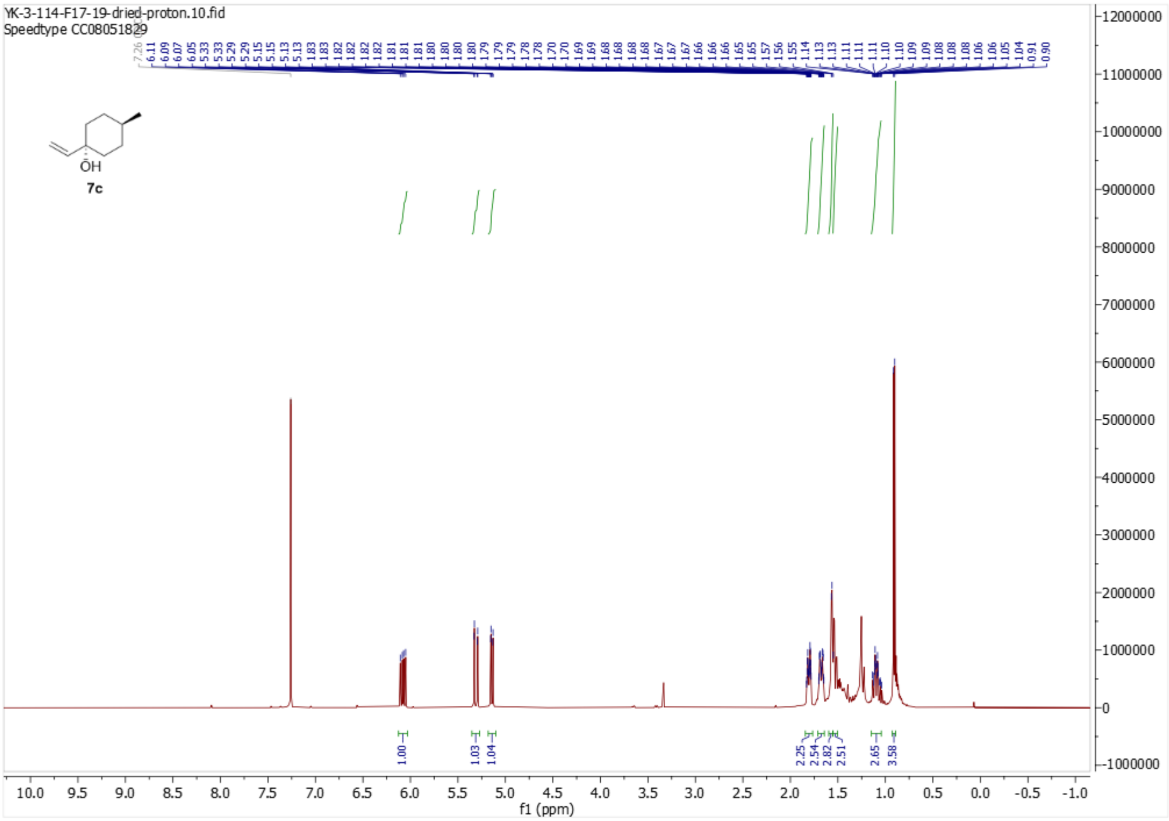
YK-3-114-F17-19-dried-proton.10.fid
Speedtype CC08051829
7c
OH
f1 (ppm)

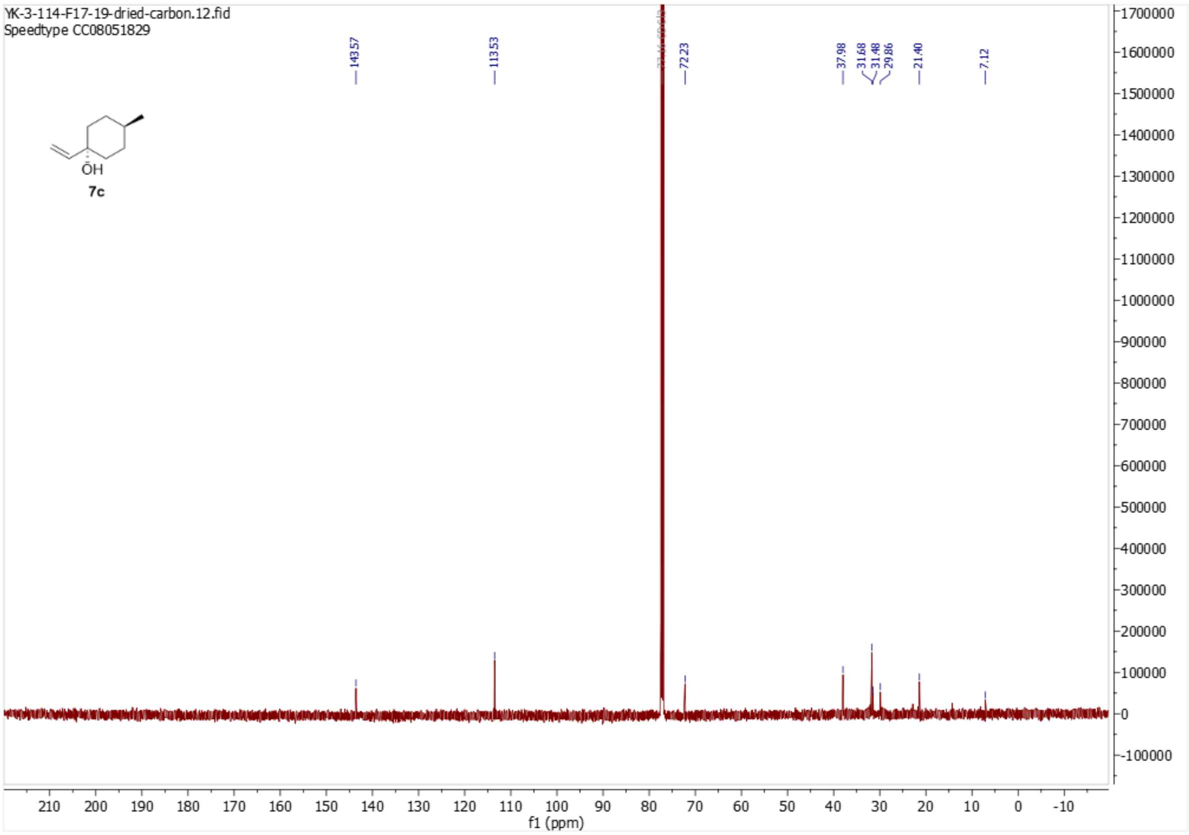
YK-3-114-F17-19-dried-carbon.12.fid
Speedtype CC08051829
143.57
113.53
72.23
37.98
31.68
31.48
29.86
21.40
7.12
7c
OH
f1 (ppm)

Note:

*Due to the volatility of this compound, only a small amount of material remained after removal of residual pentane (from column chromatography). A comparison of the peaks seen in the spectra before and after thorough removal of pentane confirms that these 6 peaks in the aliphatic region originate from compound **7c**.

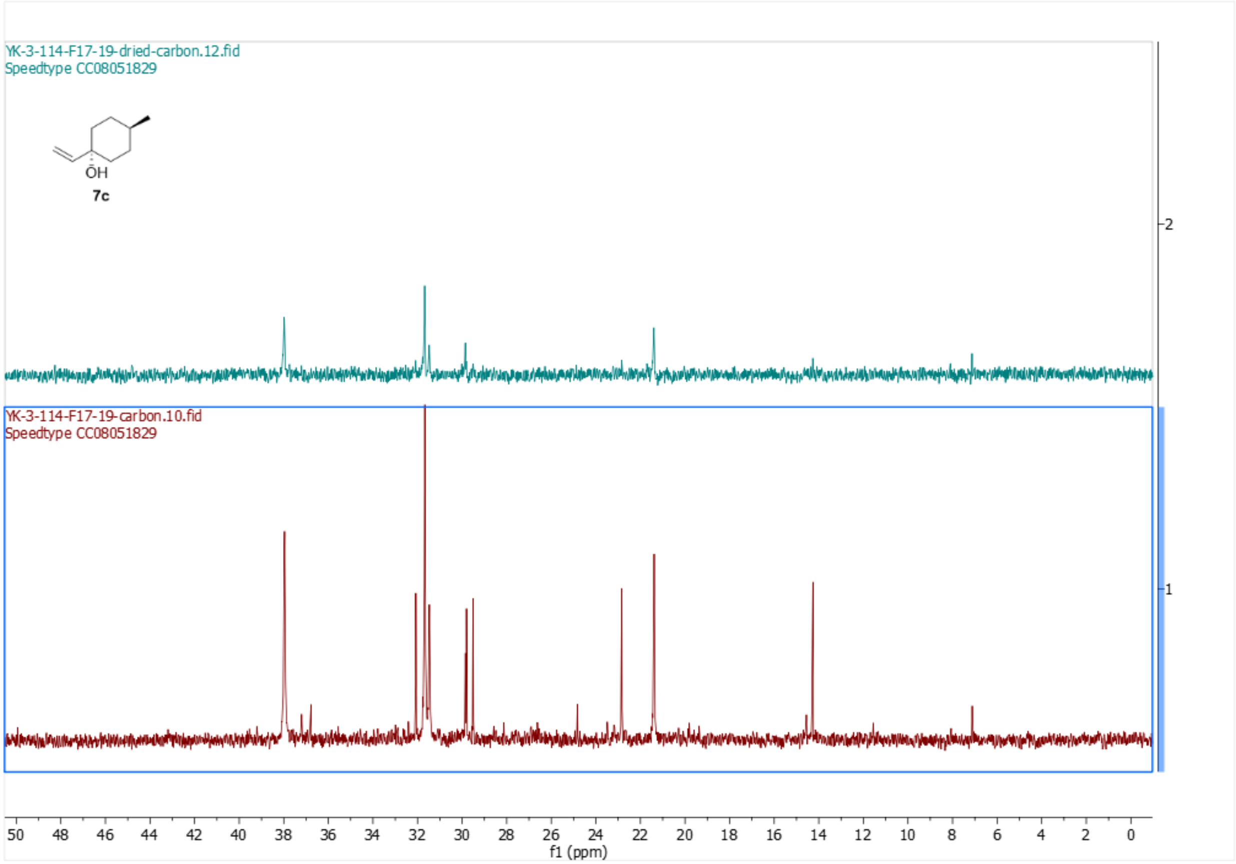

Compound 7d:

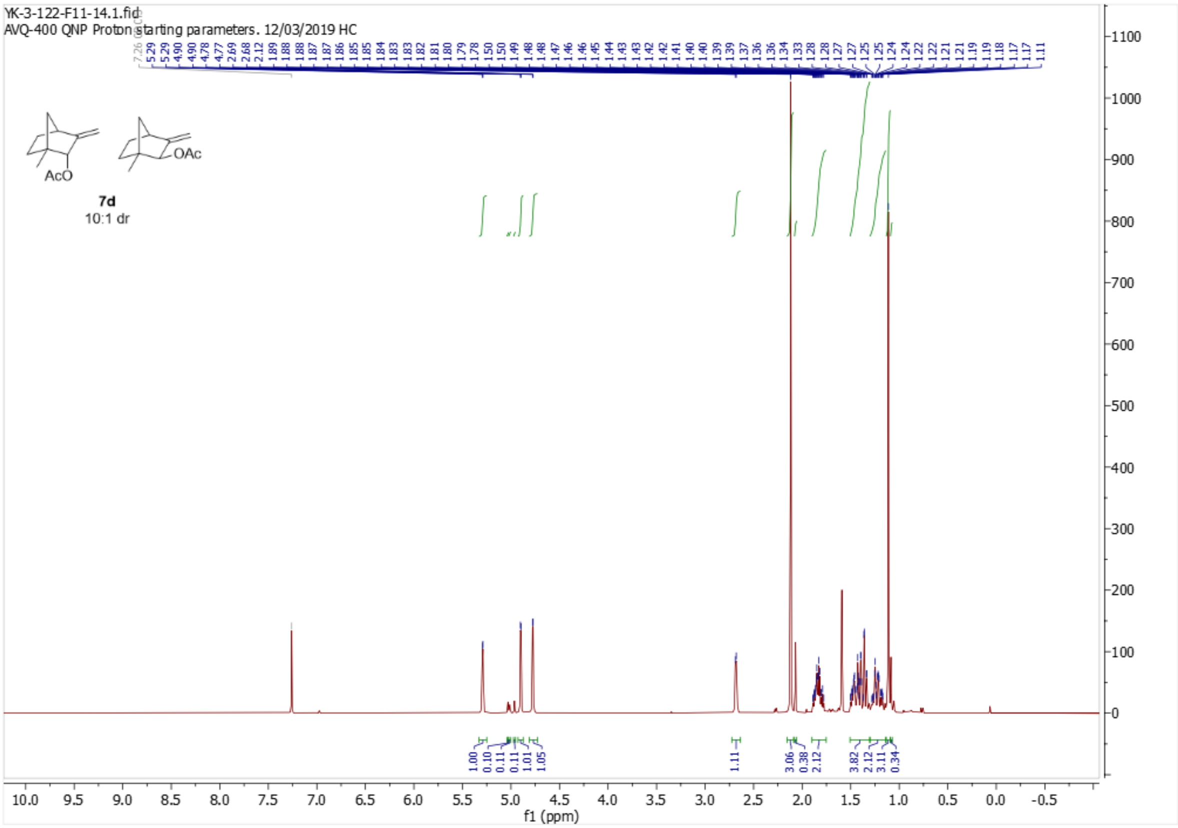

YK-3-122-F11-14.1.fid
AVQ-400 QNP Proton starting parameters. 12/03/2019 HC
7d
10:1 dr
f1 (ppm)

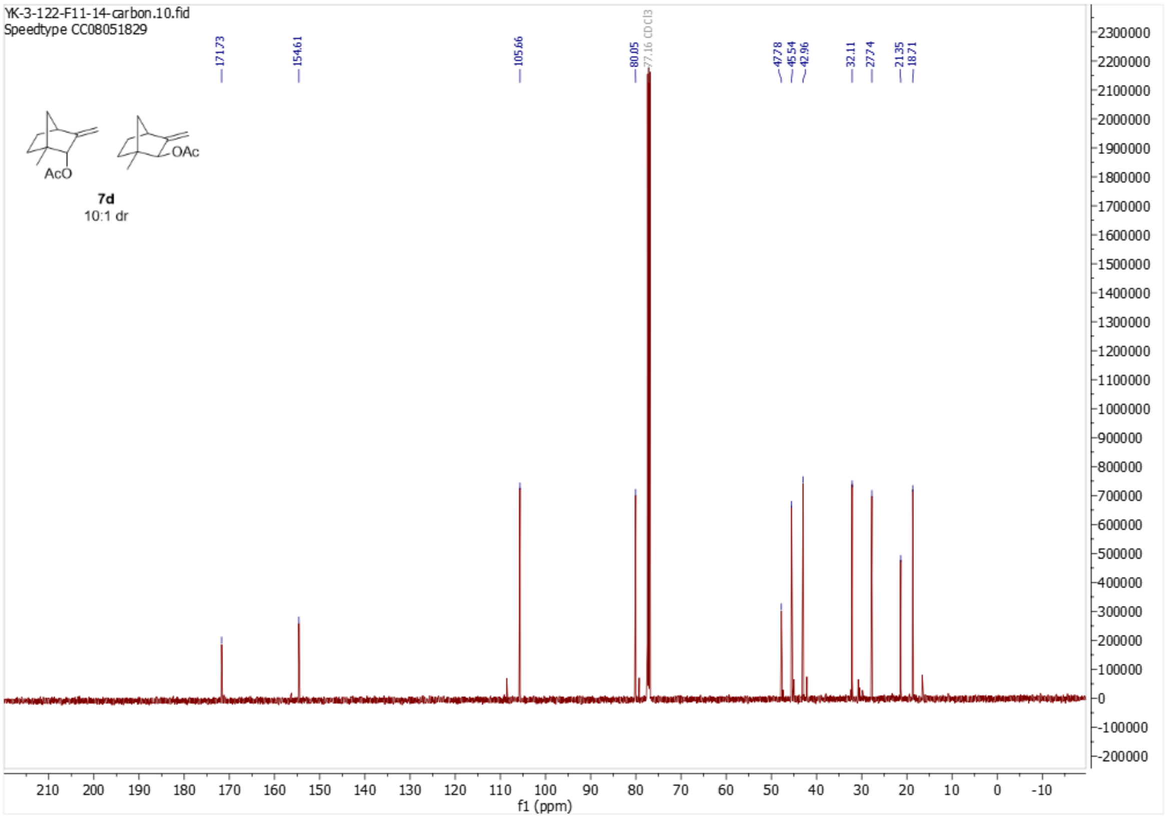

YK-3-122-F11-14-carbon.10.fid
Speedtype CC08051829
171.73
154.61
105.66
80.05
77.16 CDCl3
47.78
45.54
42.96
32.11
27.74
21.35
18.71
7d
10:1 dr
f1 (ppm)

Compound 9a:

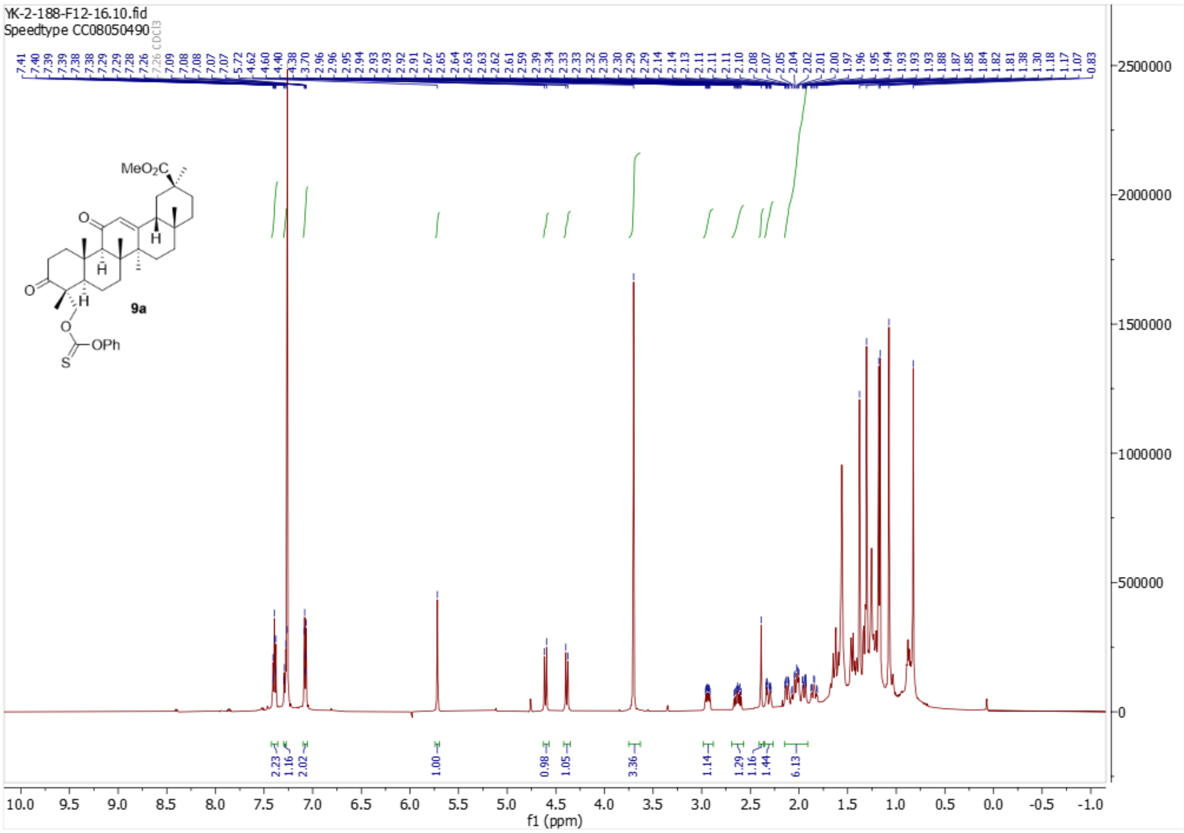

Compound 10a:

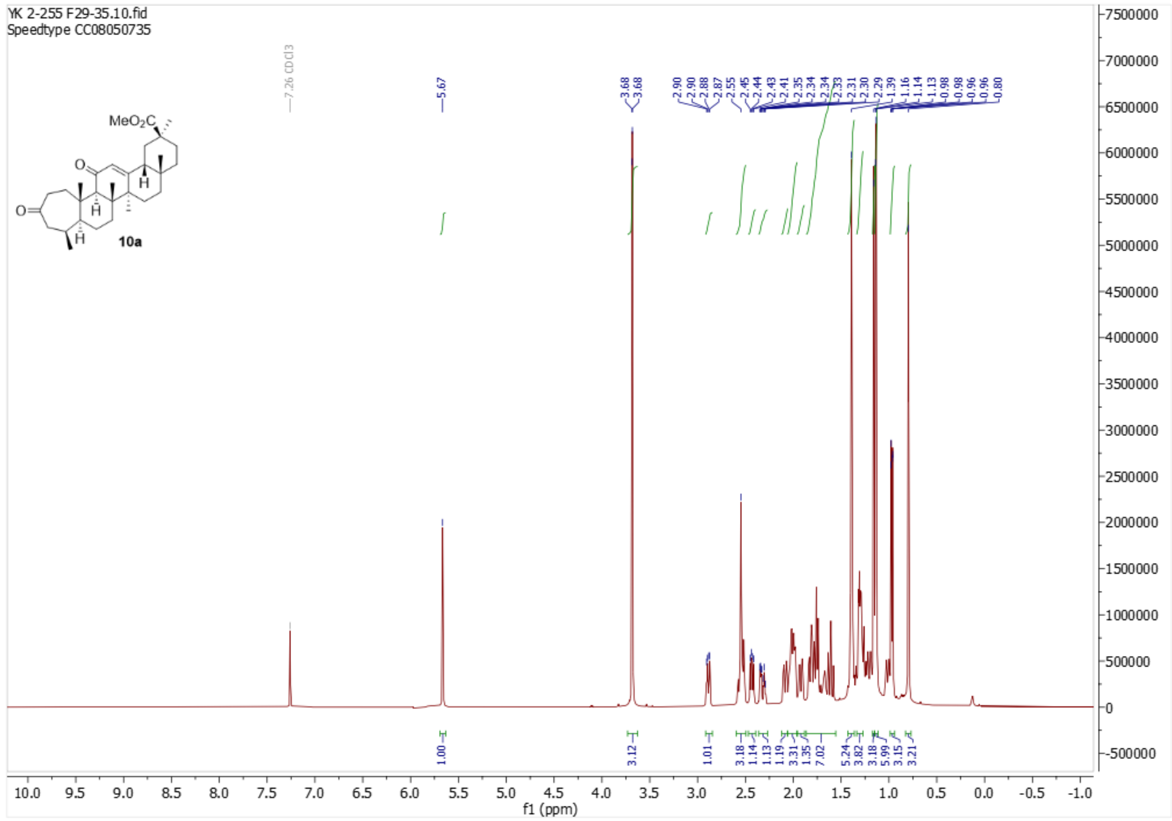

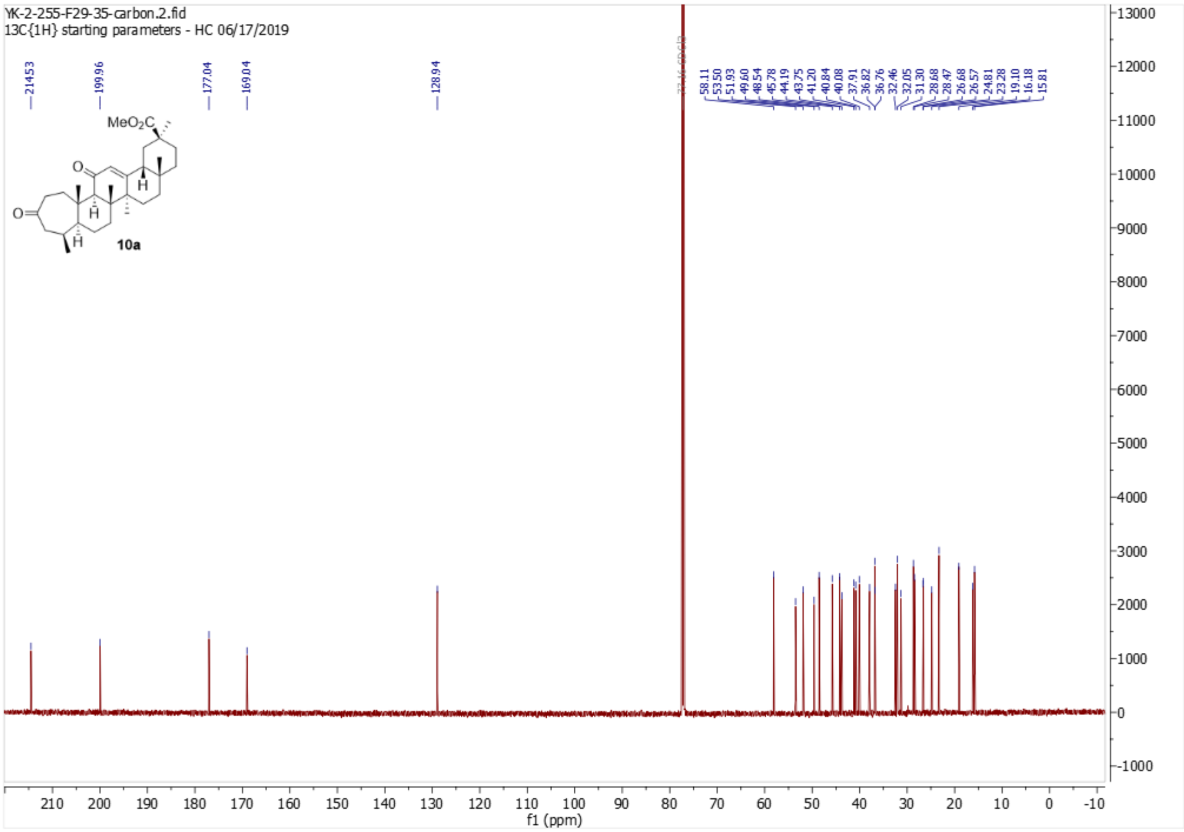

Compound S-int9b:

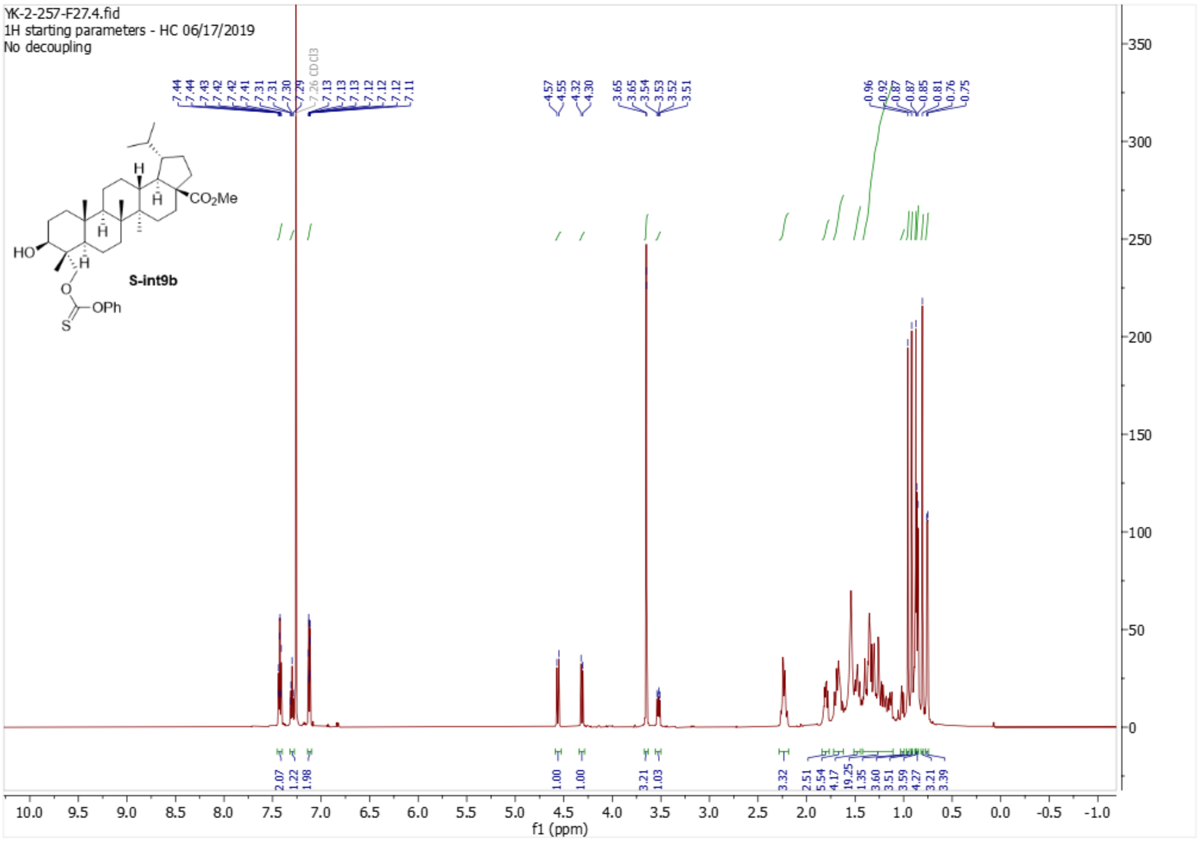

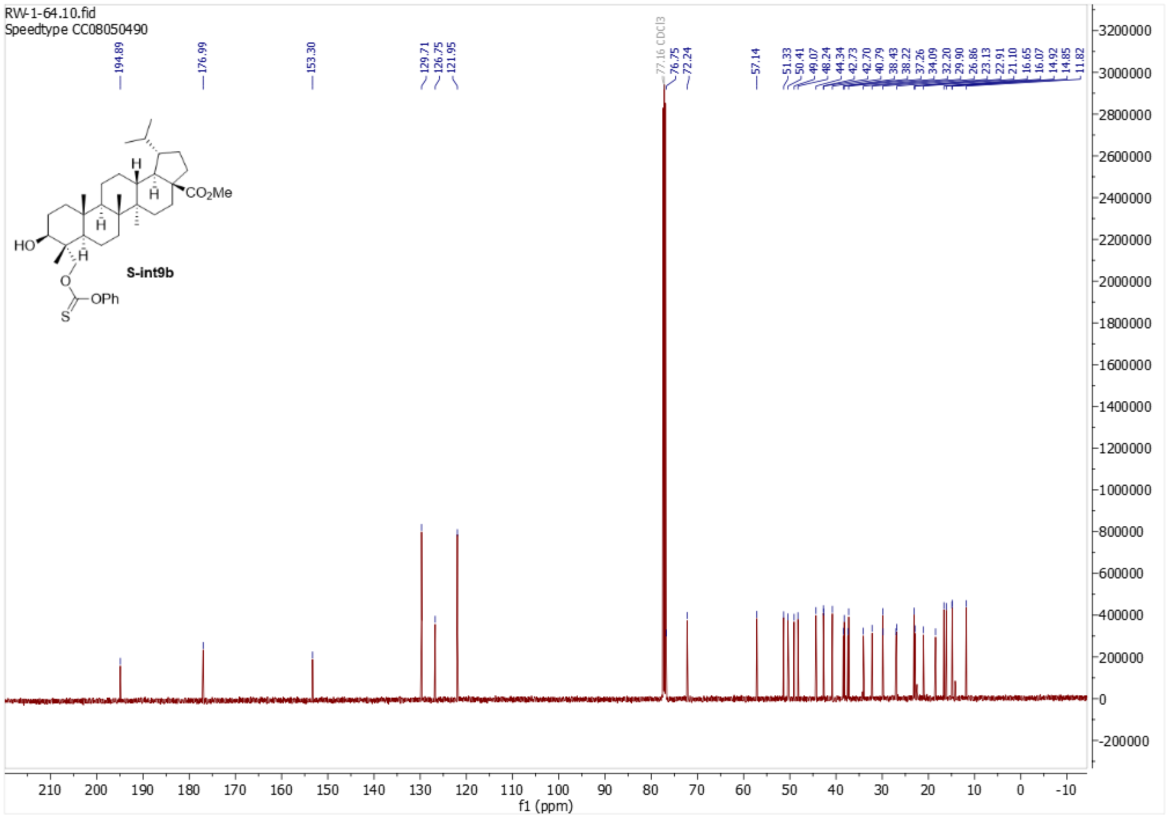

Compound 9b:

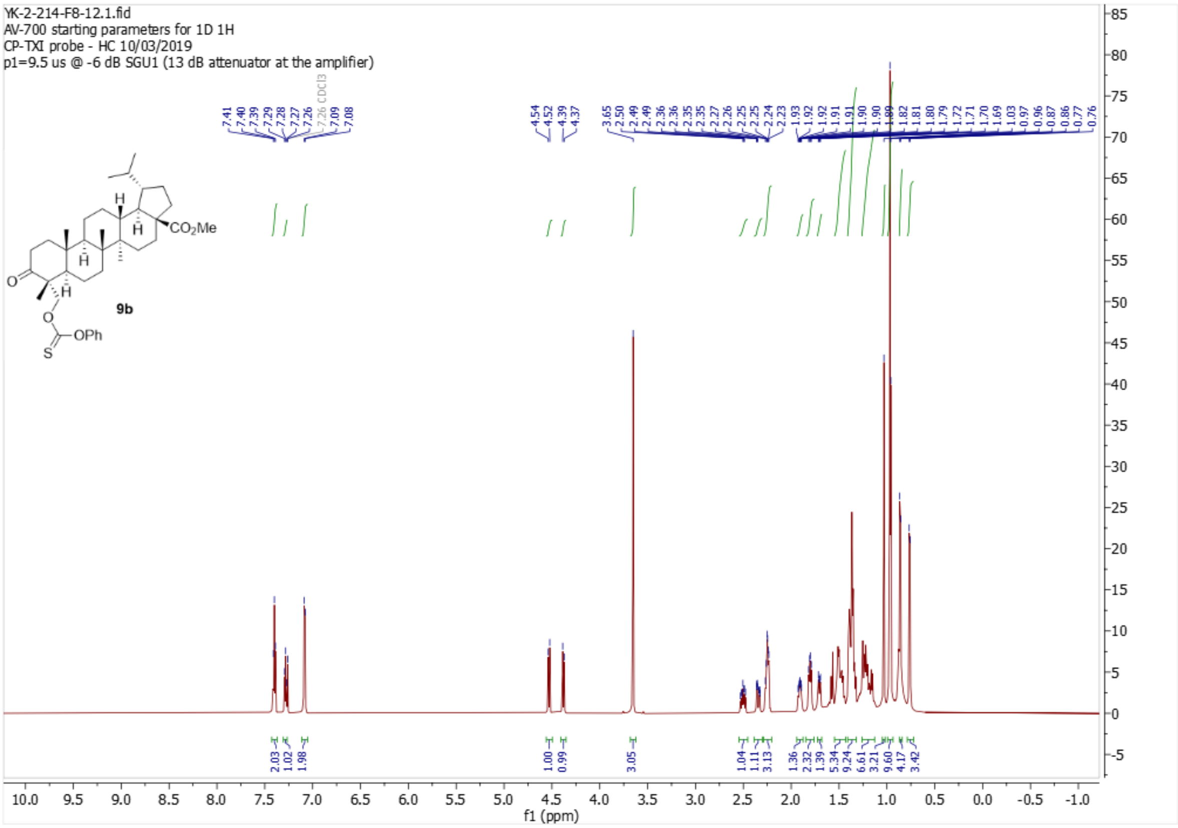

YK-2-214-F8-12.1.fid
AV-700 starting parameters for 1D 1H
CP-TXI probe - HC 10/03/2019
p1=9.5 us @ -6 dB SGU1 (13 dB attenuator at the amplifier)
9b
f1 (ppm)

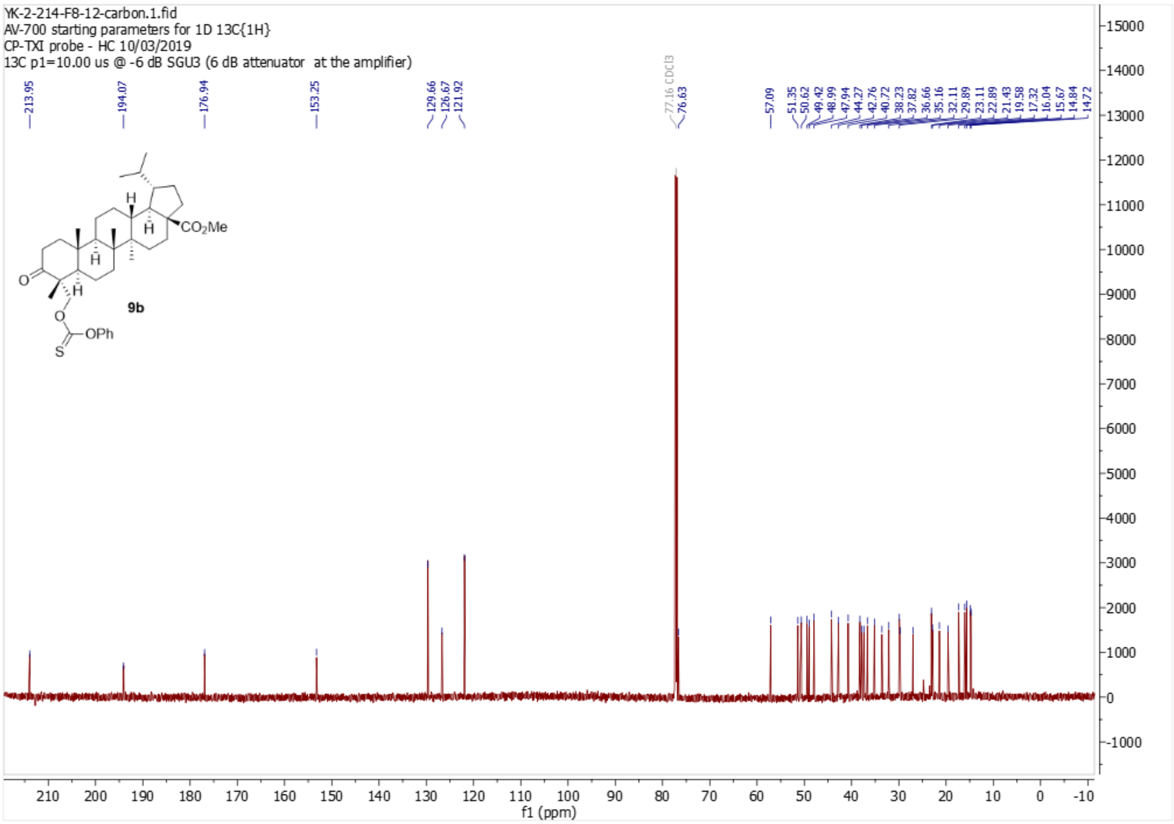

YK-2-214-F8-12-carbon.1.fid
AV-700 starting parameters for 1D 13C{1H}
CP-TXI probe - HC 10/03/2019
13C p1=10.00 us @ -6 dB SGU3 (6 dB attenuator at the amplifier)
9b
f1 (ppm)

Compound 10b:

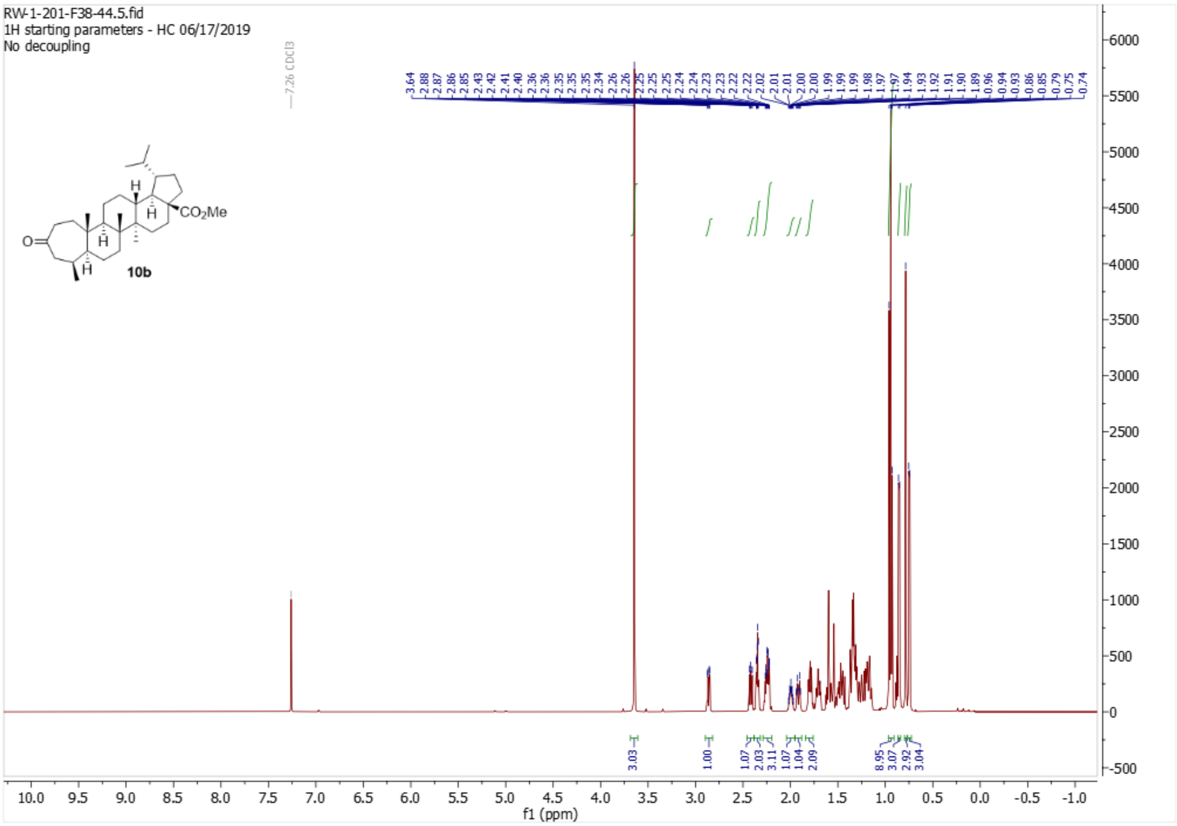

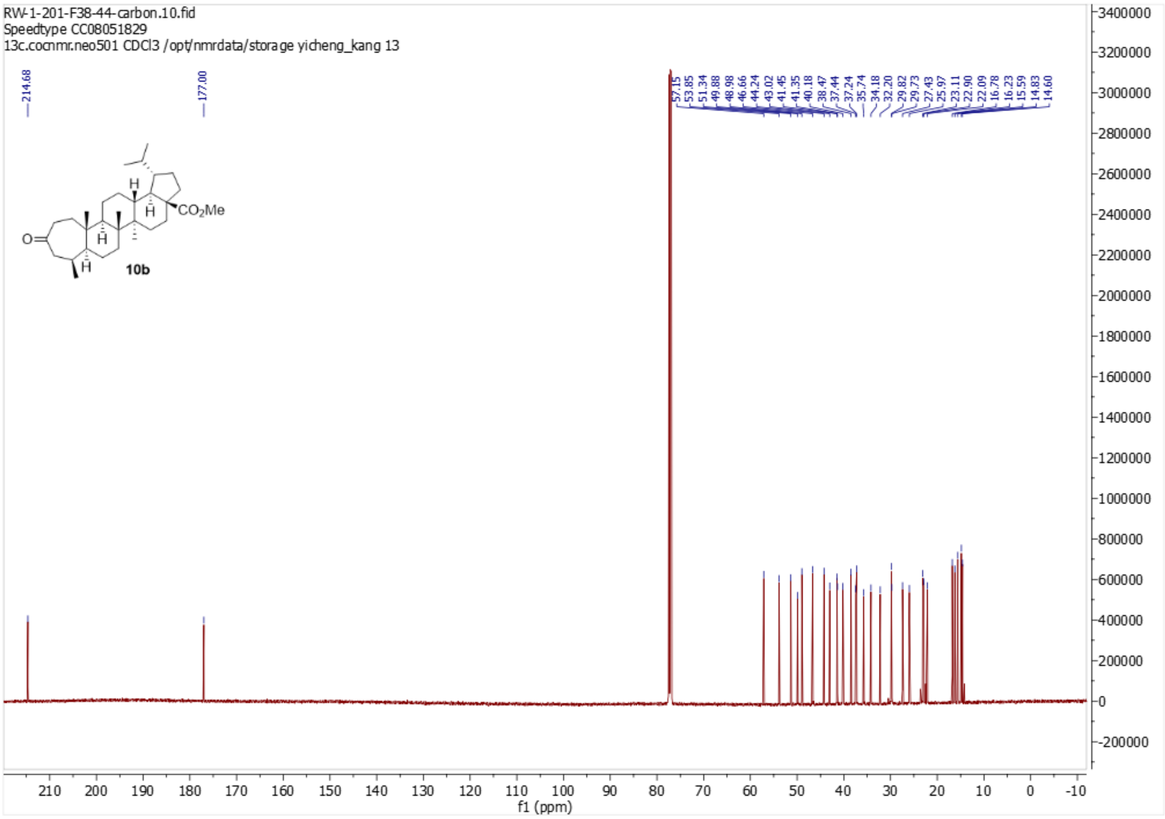

Compound 9c:

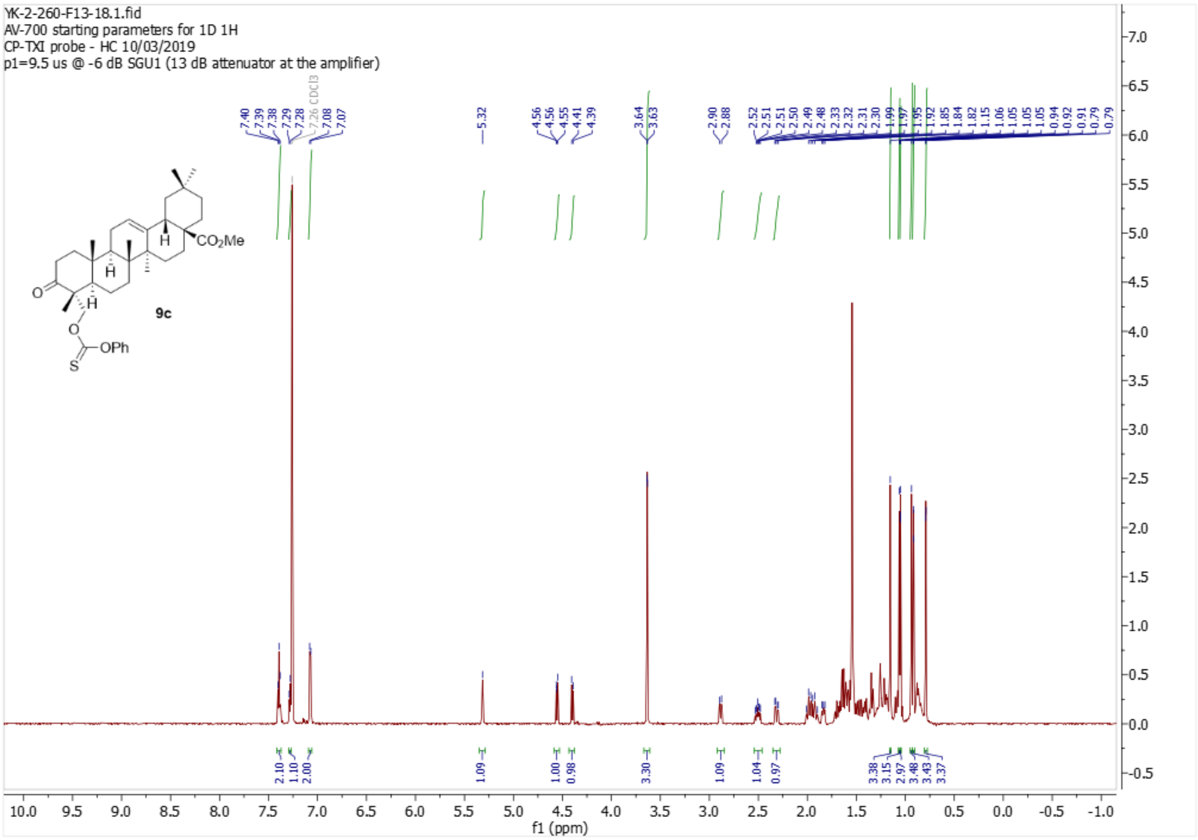

YK-2-260-F13-18.1.fid
AV-700 starting parameters for 1D 1H
CP-TXI probe - HC 10/03/2019
p1=9.5 us @ -6 dB SGU1 (13 dB attenuator at the amplifier)
9c
CO2Me
OPh
f1 (ppm)

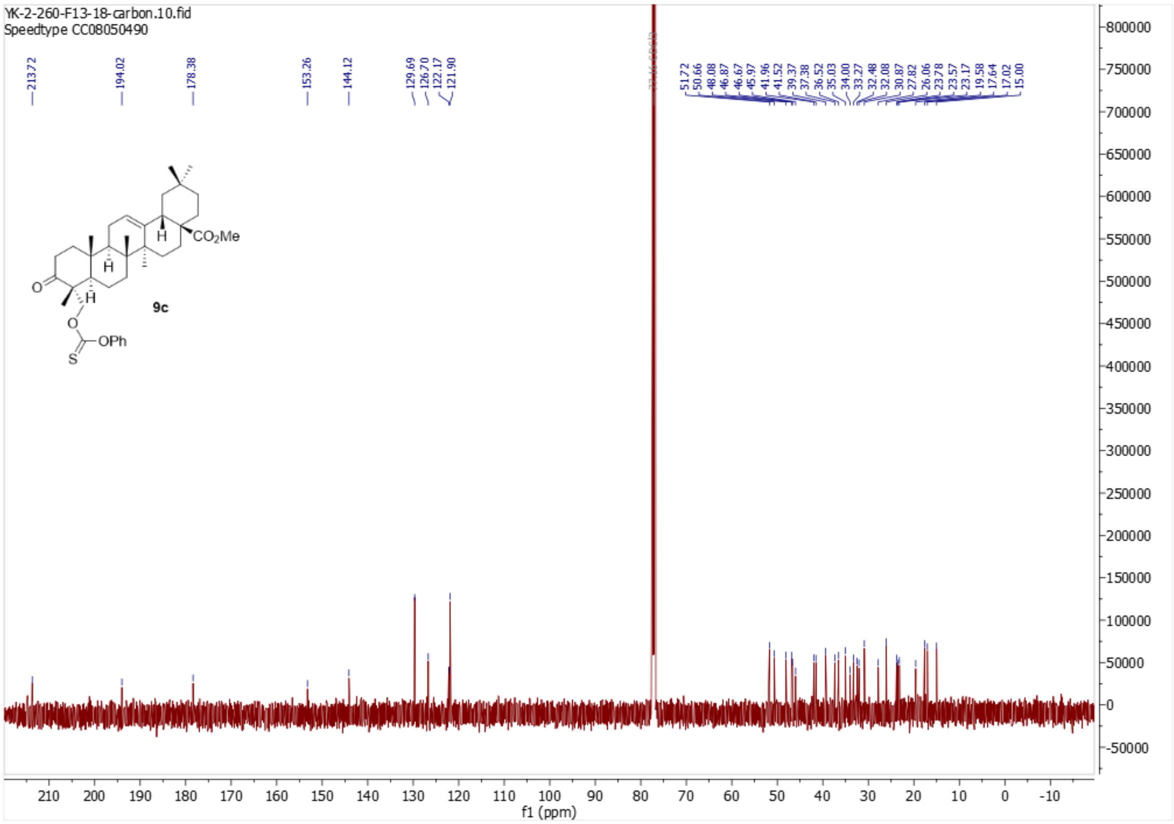

YK-2-260-F13-18-carbon.10.fid
Speedtype CC08050490
213.72
194.02
178.38
153.26
144.12
129.69
126.70
122.17
121.90
9c
CO2Me
OPh
f1 (ppm)

Compound 10c:

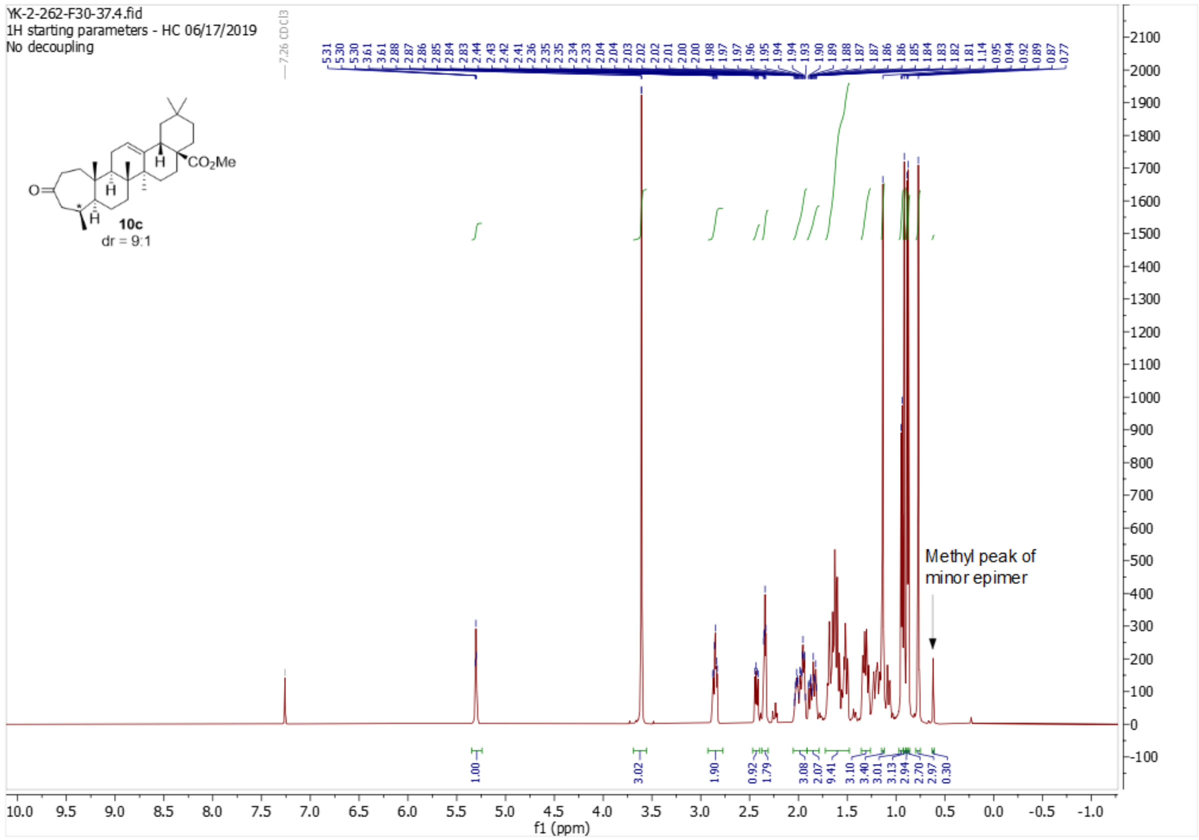

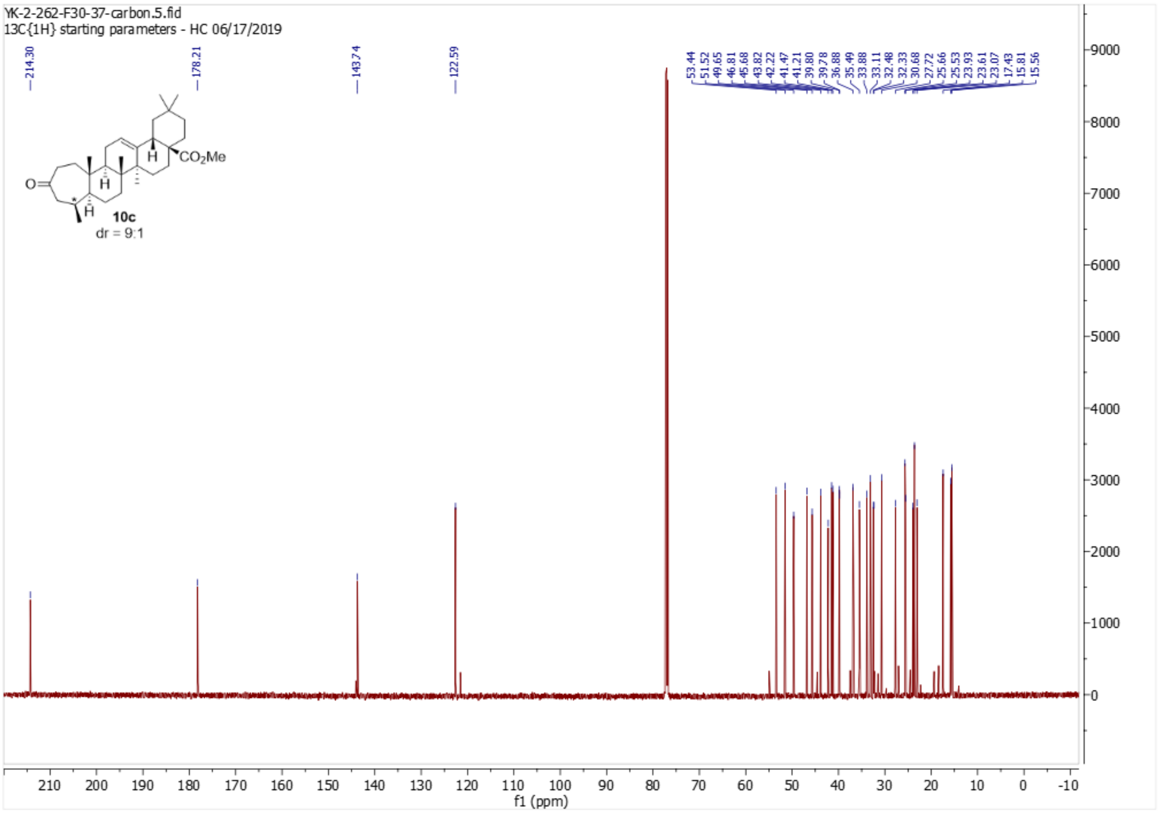

Compound 9d:

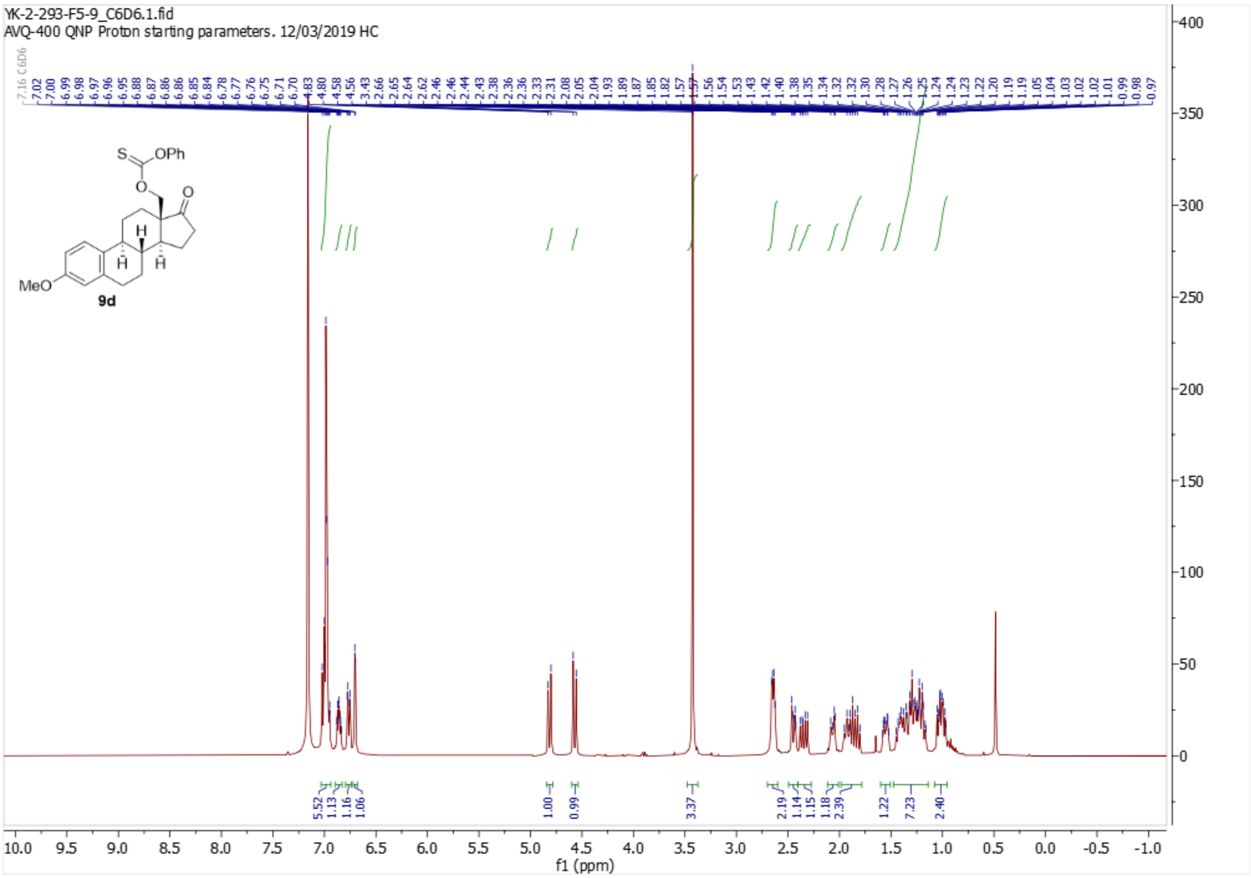
YK-2-293-F5-9_C6D6.1.fid
AVQ-400 QNP Proton starting parameters. 12/03/2019 HC
MeO
9d
OPh
f1 (ppm)

Compound 10d:

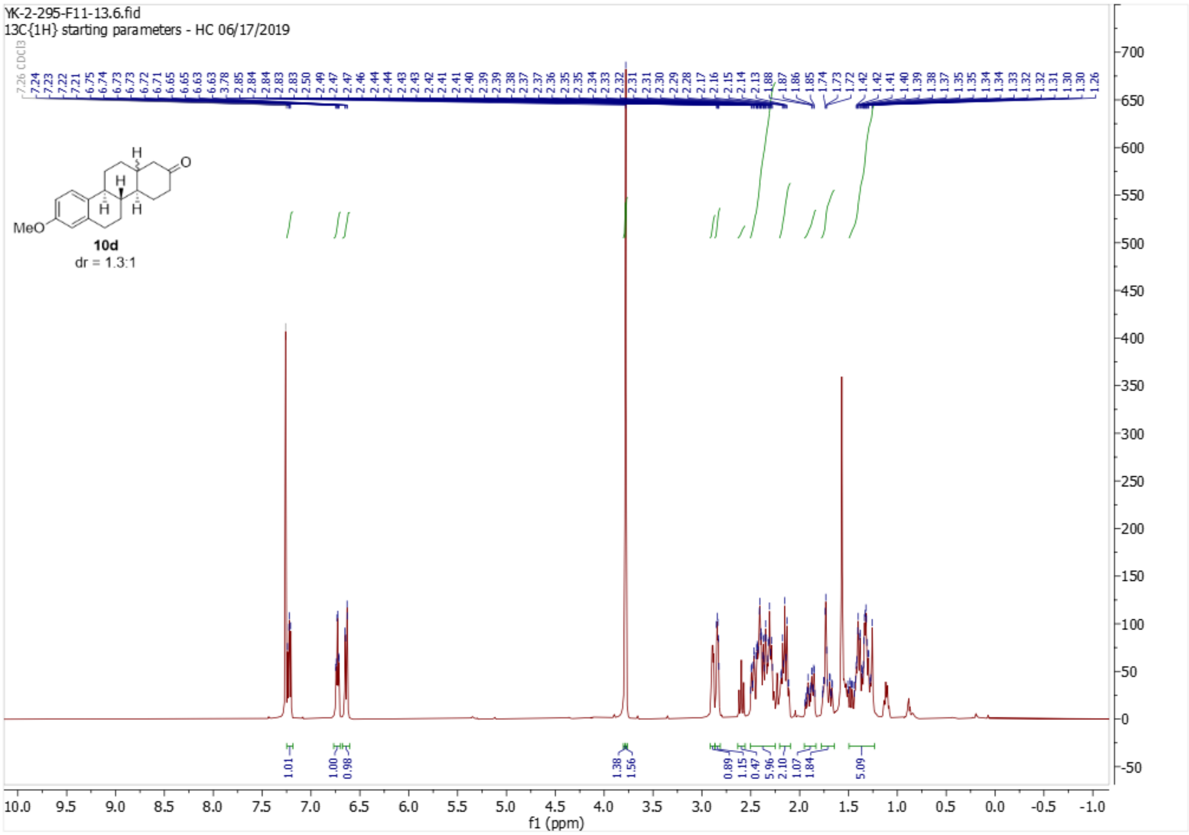

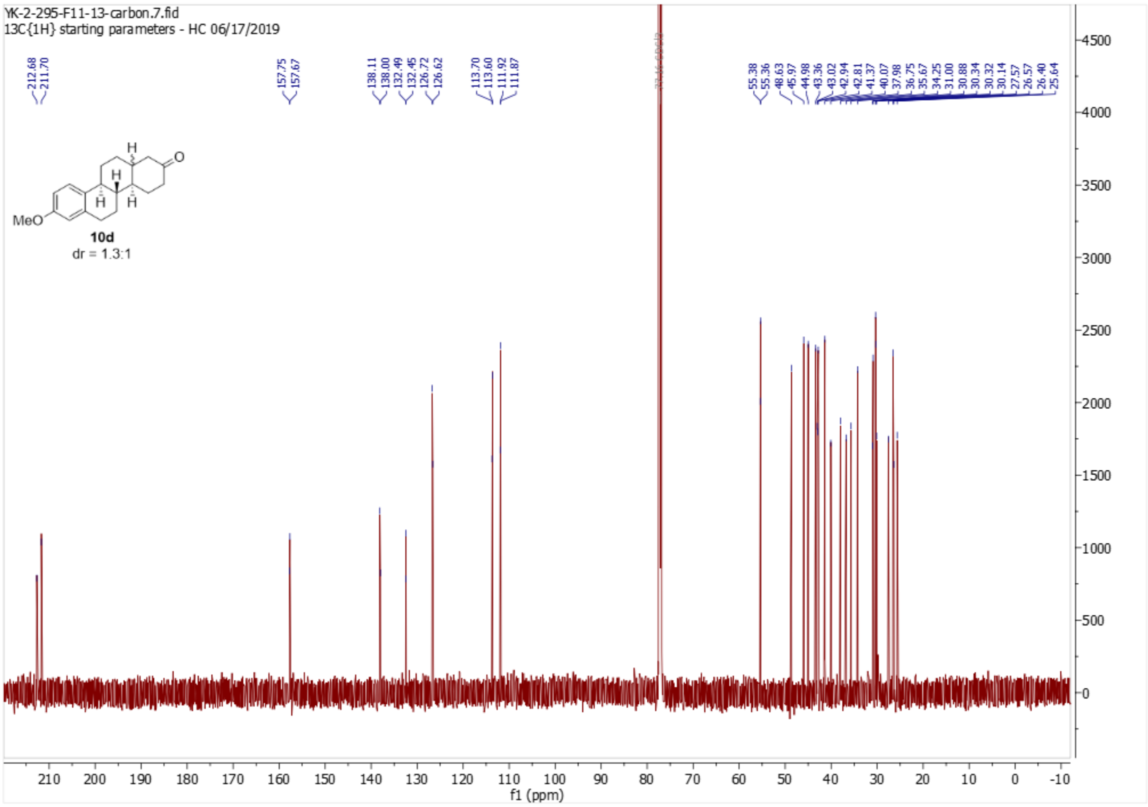

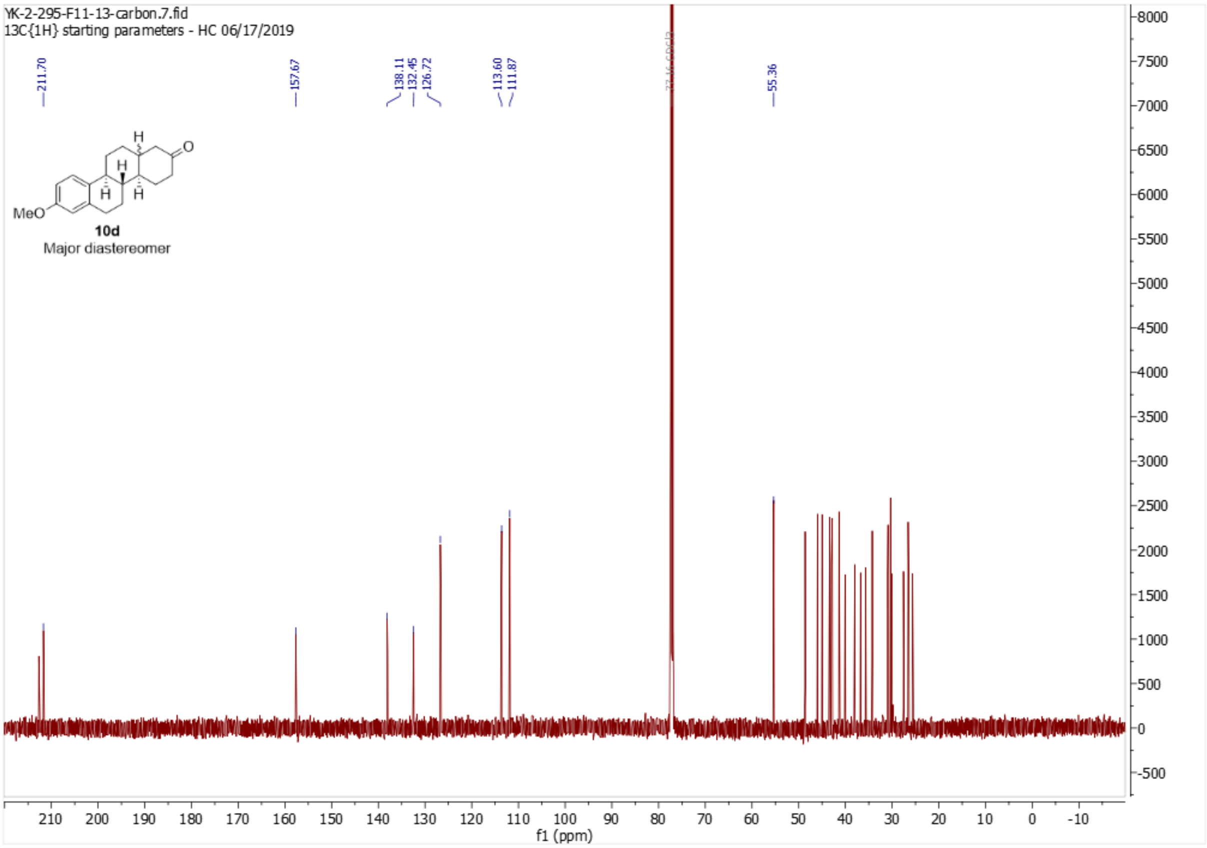
YK-2-295-F11-13-carbon.7.fid
13C{1H} starting parameters - HC 06/17/2019
211.70
157.67
138.11
132.45
126.72
113.60
111.87
55.36
MeO
10d
Major diastereomer
f1 (ppm)

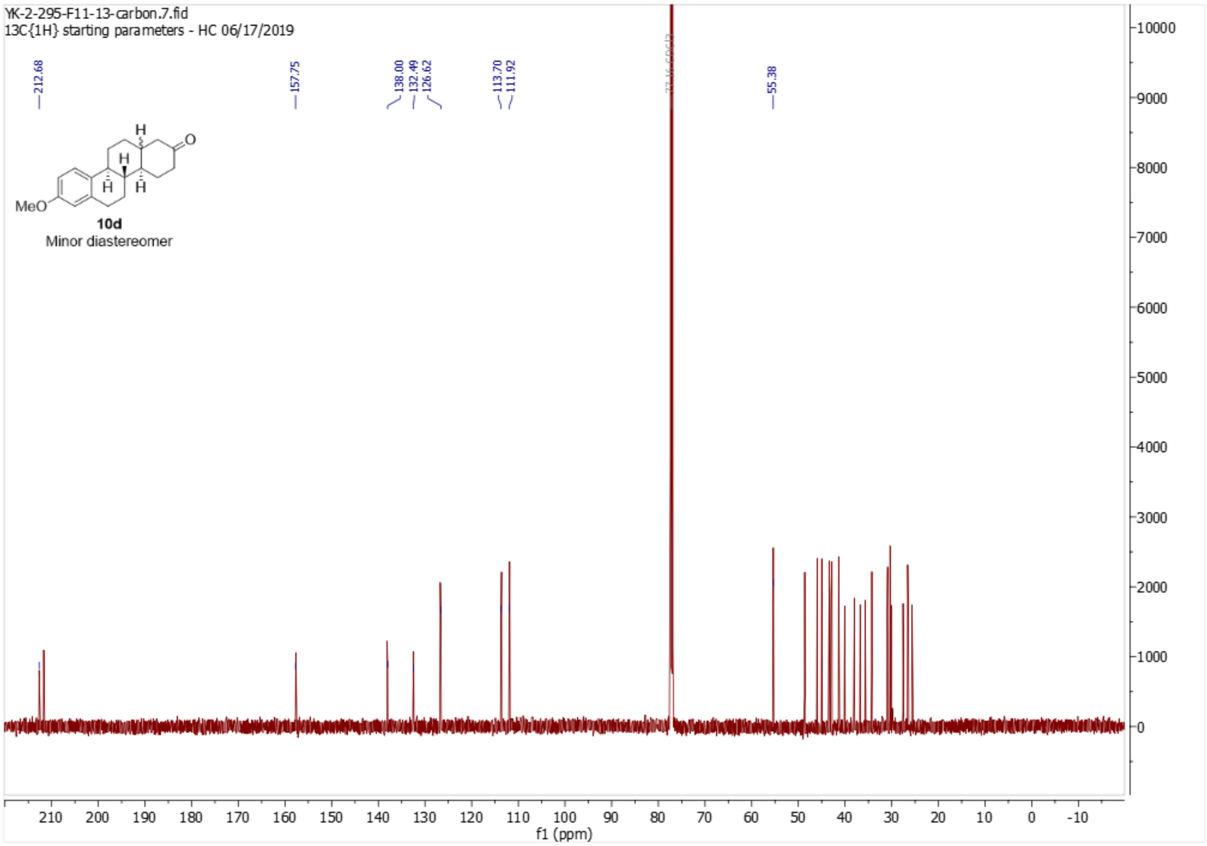
YK-2-295-F11-13-carbon.7.fid
13C{1H} starting parameters - HC 06/17/2019
212.68
157.75
138.00
132.49
126.62
113.70
111.92
55.38
MeO
10d
Minor diastereomer
f1 (ppm)
